# A Giese reaction for electron-rich alkenes†‡

**DOI:** 10.1039/d0sc06341j

**Published:** 2020-12-17

**Authors:** Qi Huang, Sankar Rao Suravarapu, Philippe Renaud

**Affiliations:** Department of Chemistry and Biochemistry, University of Bern Freiestrasse 3 CH-3012 Bern Switzerland philippe.renaud@dcb.unibe.ch

## Abstract

A general method for the hydroalkylation of electron-rich terminal and non-terminal alkenes such as enol esters, alkenyl sulfides, enol ethers, silyl enol ethers, enamides and enecarbamates has been developed. The reactions are carried out at room temperature under air initiation in the presence of triethylborane acting as a chain transfer reagent and 4-*tert*-butylcatechol (TBC) as a source of hydrogen atom. The efficacy of the reaction is best explained by very favorable polar effects supporting the chain process and minimizing undesired polar reactions. The stereoselective hydroalkylation of chiral *N*-(alk-1-en-1-yl)oxazolidin-2-ones takes place with good to excellent diastereocontrol.

## Abstract 2

Giese reaction not anymore limited to electron poor alkenes! A general method for the radical mediated hydroalkylation of electron rich alkenes including enol ethers, silylenolethers, enamides, and enecarbamates has been developed.

## Graphical abstract

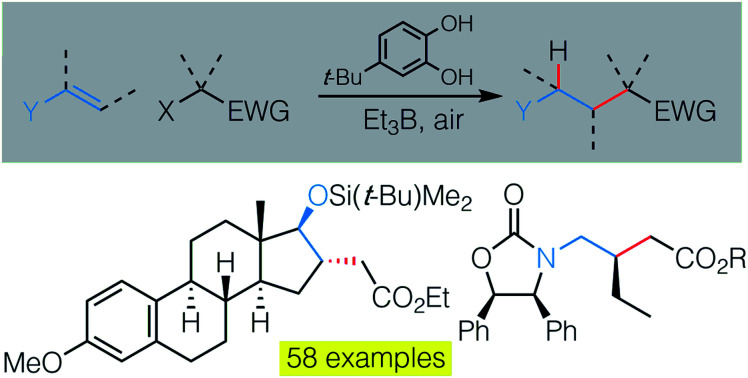

## Introduction

**Scheme 1:**
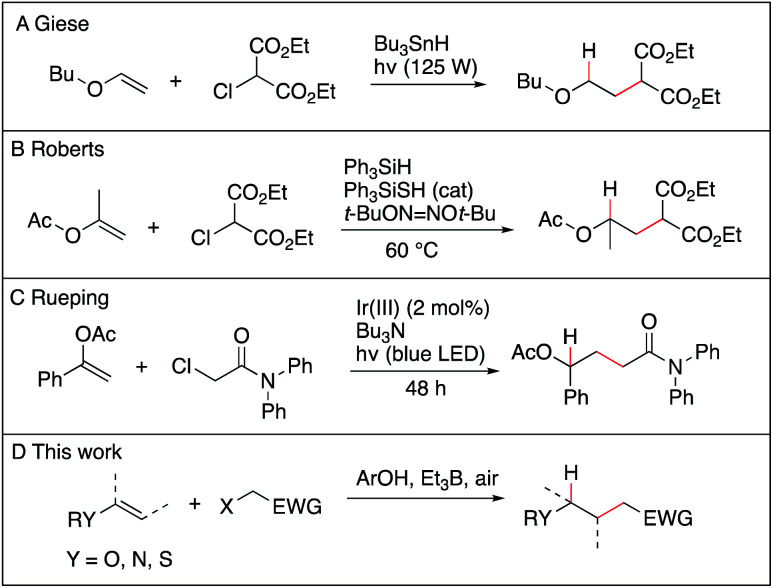
Hydroalkylation of electron-rich alkenes with anti-Markovnikov regioselectivity.

The anti-Markovnikov selective hydroalkylation of heteroatom-substituted electron-rich alkenes such as enol esters, enol ethers, thioenol ethers and enamides is an attractive process for the preparation of a variety of functionalized building blocks used for the synthesis of natural products and analogues. The well-established transition metal catalyzed hydroformylation reaction represents an effective approach to introduce one carbon atom^1^ and some promising results, such as the iridium catalyzed hydroalkylation of terminal alkenes with ureas,^2^ may emerge in the future. However, a general solution allowing to introduce a broad range of functionalized alkyl groups remains still greatly needed. Radical chemistry has been proved during the last 40 years to be one of the mildest method to achieve C–C bond formation.^3–7^ As for the hydroalkylation process, most of the reported methods described the addition of nucleophilic radicals to electron-poor olefins (the classical Giese reaction),^8–12^ the reversed process, *i.e.*, addition of electrophilic radical to electron-rich olefins, remains scarce. The addition of diethyl chloromalonate to vinyl ethers and silyl enol ethers using tributyltin hydride as the hydrogen source was reported by Giese *et al.* (Scheme 1A),^13^ followed a few years later by Renaud *et al.* who reported the hydroalkylation of enamines with sulfinylated and sulfonylated carbon-centered radicals in the presence of tributyltin hydride.^14–16^ Examples of two-step procedures involving a xanthate group transfer reaction followed by a reduction step have been reported by Zard.^17,18^ Roberts *et al.* reported triphenylsilane-mediated hydroalkylation of enol esters with electrophilic radicals in the presence of a thiol catalyst (Scheme 1B).^19^ Recently, Ryu *et al.* reported the hydroalkylation of butyl vinyl ether with ethyl 2-bromoacetate using *in situ* generated HBr as a source of hydrogen atom.^20^ Rueping *et al.* reported recently photoredox-catalyzed hydroalkylation of styrene derivatives and related olefins with α-halo amides (Scheme 1C)^21^ that was later extended to cyclization of enamides.^22^ These methods, however, suffer from serious limitations, such as limited scope, competing direct reduction of the halide, toxicity of reagents such as tin hydride, use of expensive catalyst, and long reaction time. The hydroalkylation of enol ethers, vinyl sulfides, and enamides with Markovnikov regioselectivity has been reported recently by Baran and Shenvi using an elegant metal-hydride hydrogen atom transfer process.^23–25^ Developing a general, mild and environmentally friendly method for the hydroalkylation of electron-rich alkenes with anti-Markovnikov regioselectivity remains to date an unsolved problem.

**Scheme 2:**
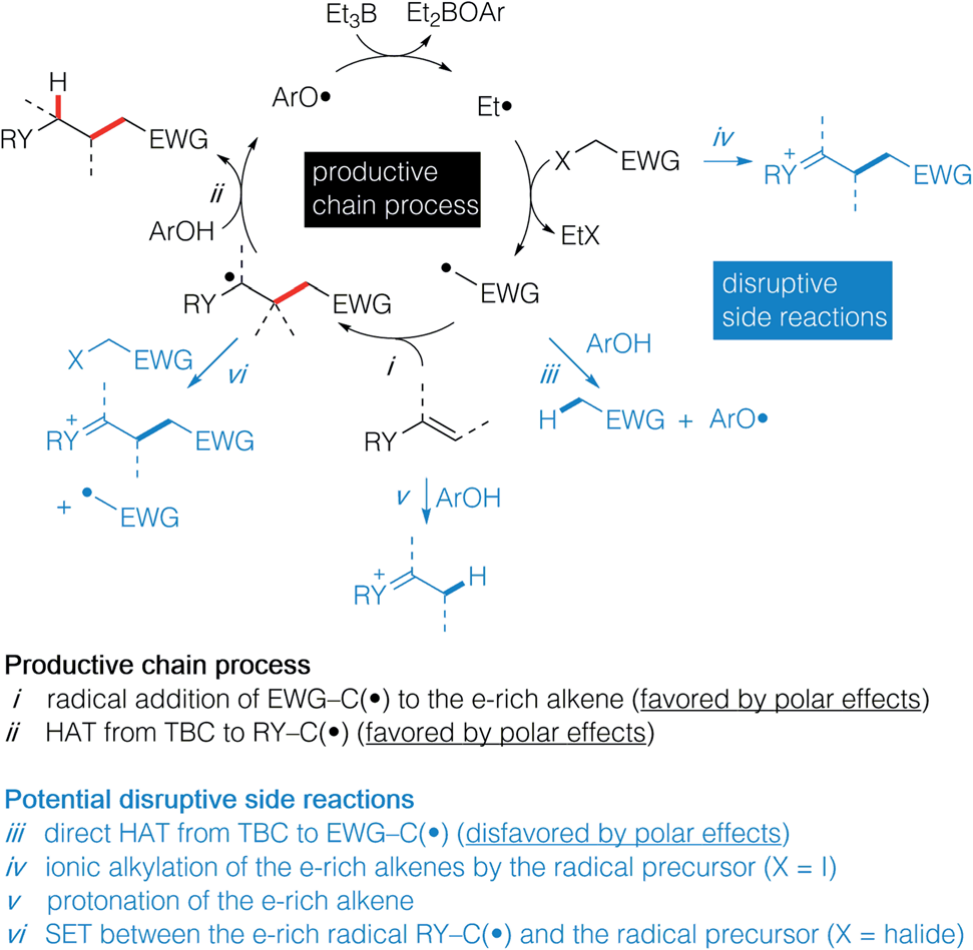
Proposed strategy for a general approach to hydroalkylated electron-rich alkenes showing the productive chain process (in black) and potential disruptive side reactions (in blue).

Recently, we have reported the hydroalkylation of mono- and polysubstituted unactivated alkenes with activated alkyl iodides by using 4-*tert*-butylcatechol (TBC) as the hydrogen source and triethylborane as an initiator and chain transfer reagent.^26,27^ The high efficiency of this reaction was attributed to strong polar effects, the catechol being a source of electrophilic hydrogen atoms, and to a unique repair mechanism, the system of catechol/Et_3_B being able to annihilate and repair undesired hydrogen atom transfer process involving the starting alkenes. Encouraged by these results, we decided to investigate the challenging hydroalkylation of electron-rich alkenes such as enol esters, enol ethers, enamides and related compounds. We described here a particularly general and simple approach to achieve this goal using TBC, a well-known biomimetic and non-toxic phenolic source of hydrogen atom (Scheme 1D). This reaction was expected to be strongly favored by polar effects since the electron-poor alkyl radicals add rapidly to the electron-rich alkenes (Scheme 2i). Moreover, the unique protic character of the OH group of TBC favors the fast reduction of the electron-rich radical adducts (Scheme 2ii) and disfavors the reduction of the initial electrophilic radicals (Scheme 2iii). Potential undesired chain disruptive side reactions such as ionic alkylations, protonation of the electron-rich alkenes (Scheme 2iv and v), and single electron transfer (SET) between the electron-rich radical adduct and the starting radical precursor (Scheme 2vi) do not take place under our reaction conditions.

## Results and discussion

### Hydroalkylation of enol esters and alkenyl sulfides

**Scheme 3:**
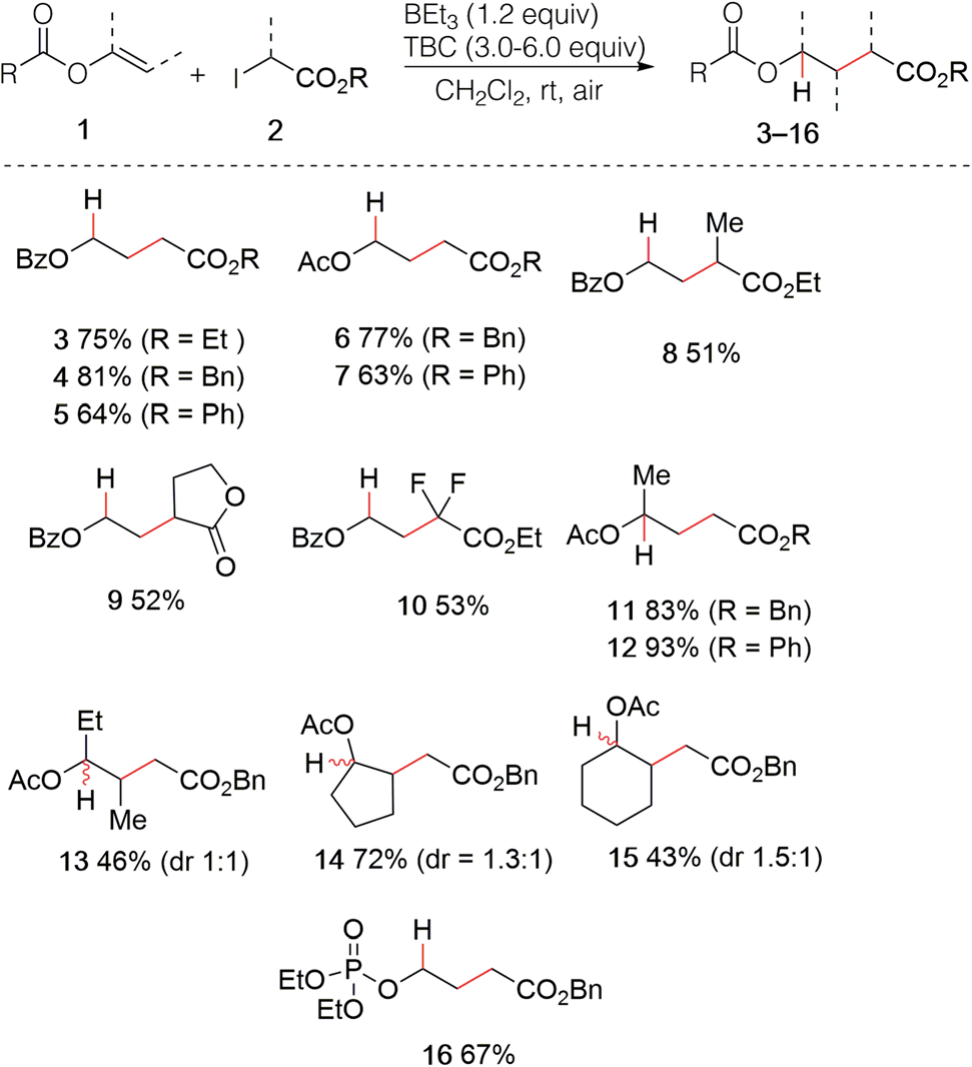
Radical hydroalkylation of enol esters and vinyl phosphate.

The use of enol esters was tested first. Simple mixing vinyl benzoate (5.0 equiv.), ethyl iodoacetate (1.0 equiv.), TBC (3.0 equiv.) and triethylborane (1 M in hexane, 1.2 equiv.) in dichloromethane under nitrogen atmosphere followed by stirring the reaction mixture open to air afforded the desired hydroalkylated product 3 in 75% yield (Scheme 3). Various di- and trisubstituted enol esters were tested using different electrophilic radical precursors. The method worked efficiently with terminal (3–12) as well as non-terminal enol esters (13–15) and can be also extended to the phosphate ester (16). A broad range of 2-iodoesters such as simple iodoacetates (3–7, 11–16), 2-iodopropionates (8), the iodolactone (9) and the difluoroiodoacetate (10) were all found to react cleanly under these reaction conditions.

**Scheme 4:**
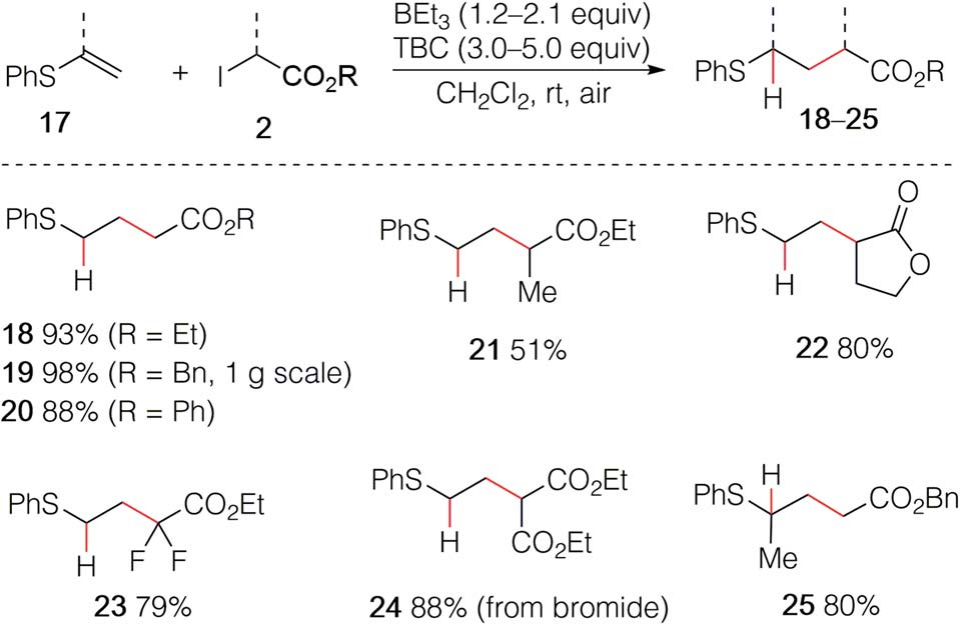
Hydroalkylation of alkenyl sulfides.

The reaction was then extended with success to alkenyl sulfides 17, affording the corresponding sulfides 18–25 in good to excellent yields (Scheme 4). The sulfide 19 was easily prepared by using this procedure on gram scale. Interestingly, the diethyl malonate derivative 24 was prepared in high yield using the corresponding bromomalonate radical precursor.

### Hydroalkylation of enol ethers

**Scheme 5:**
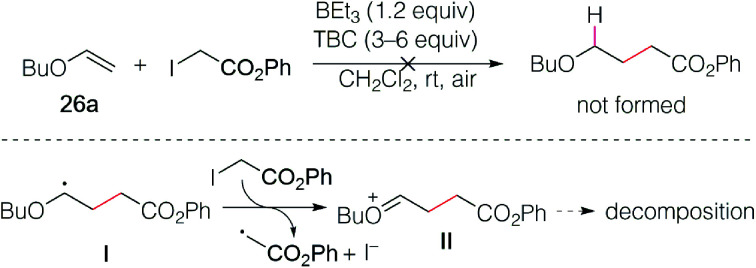
Reaction of phenyl iodoacetate with butyl vinyl ether.

The reaction of butyl vinyl ether (26a) and phenyl iodoacetate was then attempted but led to decomposition products (Scheme 5). This was attributed to a fast electron transfer between radical adduct I and the starting iodoester 2c, leading to the formation of the oxonium ion II that decomposes presumably by oligomerization processes involving the starting vinyl ether. Similar reactions have been reported by Giese in his seminal work.^13^

**Scheme 6:**
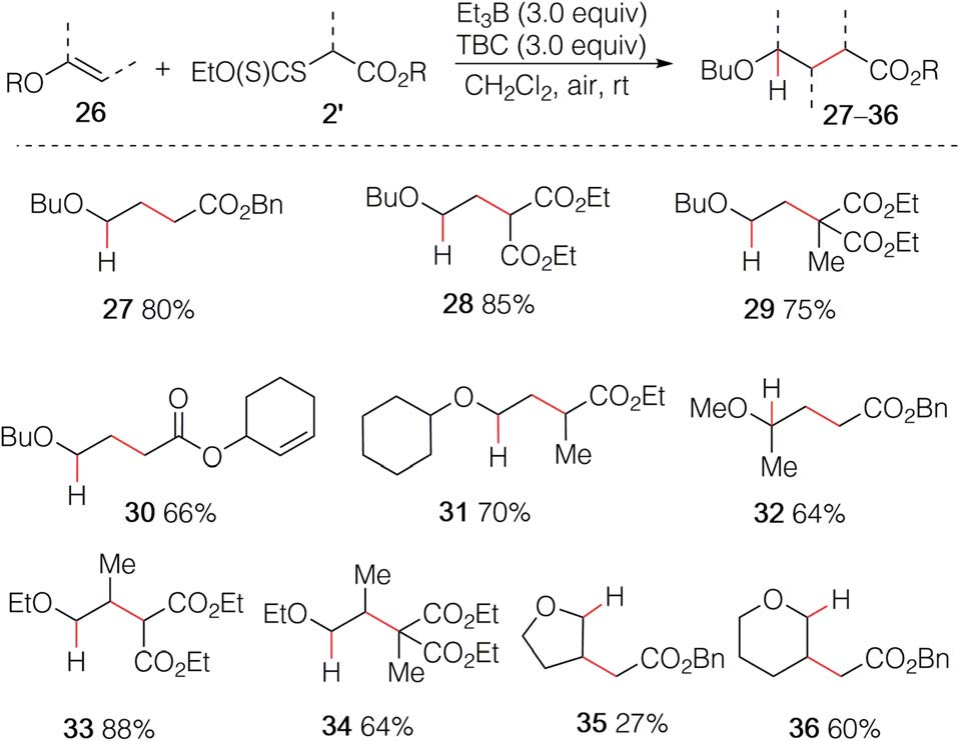
Radical hydroalkylation of enol ethers with xanthates.

By employing xanthate radical precursors 2′ that are less prone to single electron transfer reduction than iodides,^18,28–30^ the hydroalkylation of enol ethers 26 could be successfully performed (Scheme 6). For instance, reaction of vinyl ethers with various xanthates afforded the hydroalkylated products 27–31 in 70–85% yield. Noteworthy, the reaction between the unsaturated cyclohex-2-en-1-yl acetate xanthate and butyl vinyl ethers 26a led to product 30 resulting from intermolecular addition in 66% yield, while no cyclized product was detected.^31^ Similar result was obtained for 32 starting from 2-methoxypropene. Interestingly, non-terminal 1-ethoxypropene also reacted efficiently to deliver the corresponding adducts 33 and 34 in 88% and 64% yield, respectively. Cyclic enol ethers such as 2,3-dihydrofuran and 3,4-dihydro-2*H*-pyran did not react cleanly at room temperature and better results were obtained by running the reaction at 0 °C (35 27%, 36 60%).

**Scheme 7:**
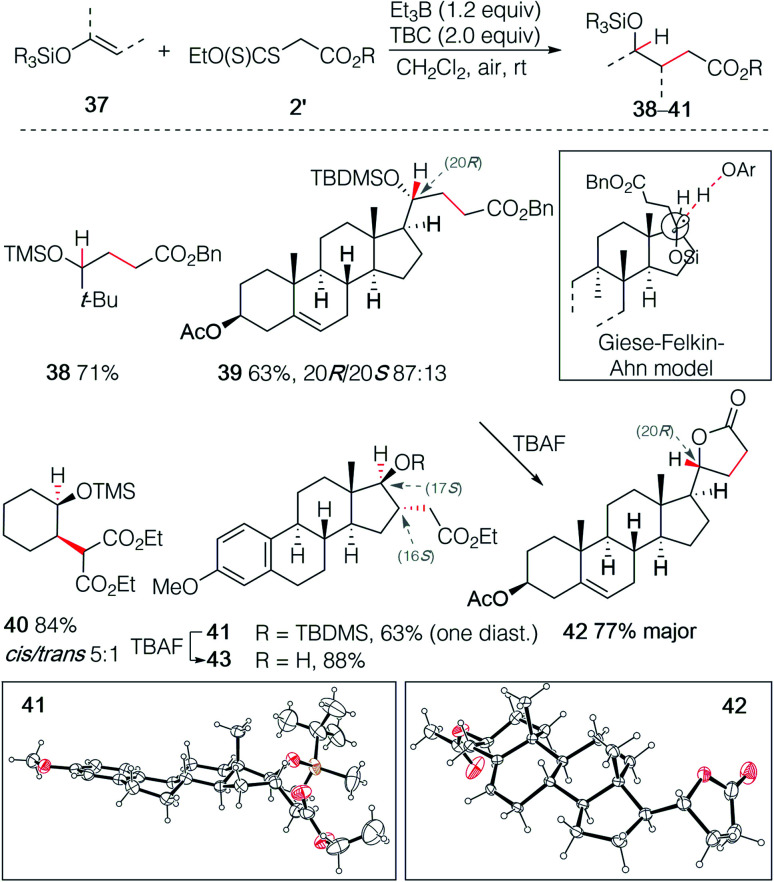
Radical hydroalkylation of silyl enol ethers with xanthates. Single crystal X-ray structures of 42 and 43 (ellipsoids drawn at 50% probability).

The hydroalkylation of terminal silyl enol ethers was examined next. Terminal silyl enol ethers derived from *tert*-butyl methyl ketone and pregnenolone acetate gave the desired γ-silyloxy esters 38 and 39 in 71% and 63% yield, respectively (Scheme 7). The silyl ether 39 was obtained with a good control of the stereochemistry (20*R*/20*S* 87 : 13). The stereochemical outcome is rationalized by the Felkin–Ahn type model introduced by Giese for 1-alkoxysubstituted radicals.^32^ This example illustrates also the high regioselectivity of this hydroalkylation process. Indeed, the double bond in ring B of pregnenolone that can be hydroalkylated in 65% yield under similar reaction conditions^26^ remains untouched, demonstrating further the critical importance of polar effects in this reaction. Upon deprotection of the *tert*-butyldimethylsilyl (TBDMS) ether with TBAF, spontaneous lactonization affording 42 was observed. The major diastereomer of 42 was obtained in 77% yield and its (*R*) configuration at C(20) was confirmed by single crystal X-ray crystallography (Scheme 7).^33–37^ Similar results were obtained with the non-terminal silyl enol ethers derived from cyclohexanone and estrone methyl ether that gave the γ-silyloxy esters 40 and 41 in 84% (*cis*/*trans* mixture 5 : 1) and 59% (single diastereomer) yield, respectively. The relative configuration at C(16) and C(17) of 41 was established by single crystal X-ray crystallography (Scheme 7),^33–37^ indicating that both the stereochemical outcome of the radical addition and of the hydrogen atom transfer are controlled by the axial C(18)-methyl group. Deprotection of the silyl ether of 41 gave the stable *trans*-γ-hydroxy ester 43 and no lactonization could be achieved even under acidic treatment. Interestingly, the hydroalkylation of Me_3_SiO-cyclohexene reported by Baran and co-workers^23,24^ using an iron catalyzed process and by Shenvi using a dual manganese/nickel catalyzed process delivered adducts with the opposite regioselectivity.^25^

### Hydroalkylation of enamides and enecarbamates

**Scheme 8:**
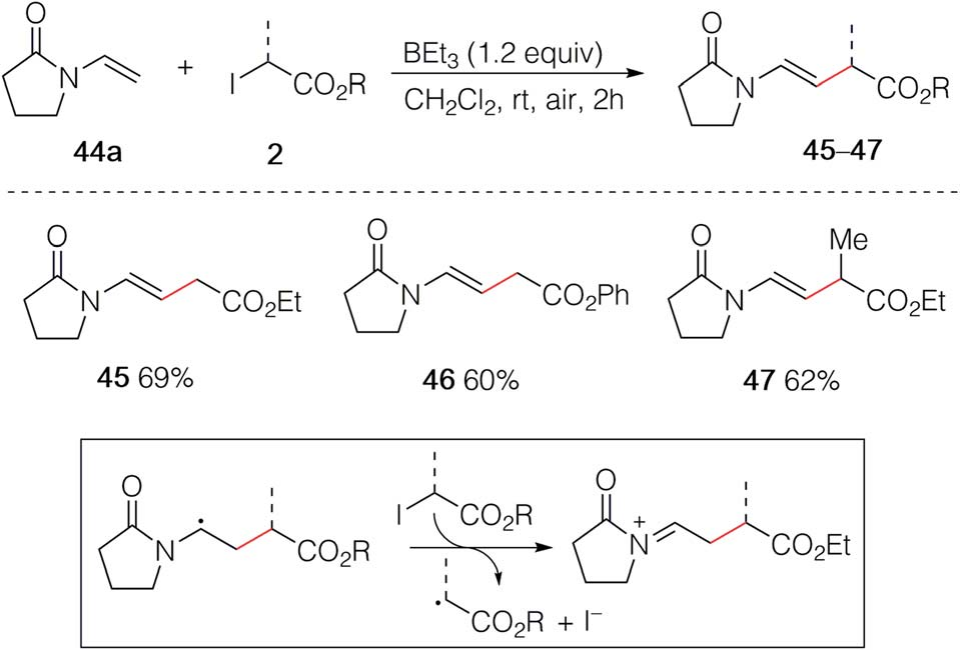
Non-reductive alkylation of 1-vinylpyrrolidin-2-one 44a with iodoesters 2.

In an early attempt to run the hydroalkylation of 1-vinylpyrrolidin-2-one (44a) using ethyl iodoacetate, no trace of the hydroalkylated product was observed. Instead, the alkylated enamide 45 resulting from a non-reductive process was isolated. Rapid optimization of this process showed, as expected, that TBC was not necessary for this transformation and good yields of 45, 46 and 47 were obtained upon simple treatment of 44a with the correspond α-iodoesters in the presence of triethylborane which is presumably acting as a radical initiator in the presence of air and as a scavenger for HI generated during the process (Scheme 8). A similar non-reductive alkylation has already been reported by Friestad and Wu but required the use of a stoichiometric amount of tin hydride and a tertiary amine base.^38^ The reaction proceeds *via* formation of an acyliminium ion resulting most probably from a single electron transfer process between the radical adduct and the starting iodide 2 followed by elimination of a proton (Scheme 8, frame).^39^

**Scheme 9:**
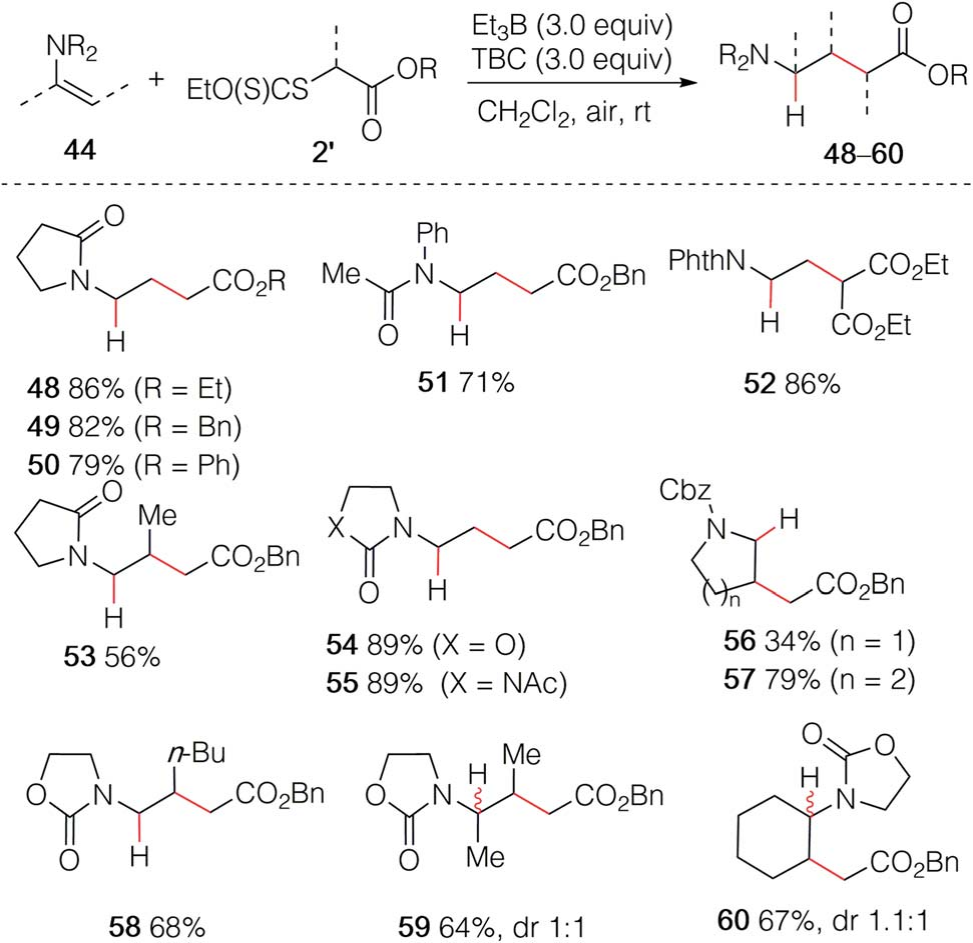
Hydroalkylation of enamides and enecarbamates.

As already demonstrated for the enol ethers, the use of xanthate radical precursors 2′ allows to suppress the single electron transfer step and favor the hydroalkylation process.^40–45^ The hydroalkylation of enamides was examined first. Terminal enamides afforded the desired hydroalkylated products 48–52 in excellent yields (Scheme 9). Reaction involving a non-terminal enamide led to the hydroalkylated products 53 in satisfactory yields. Similar results were obtained with terminal (54 and 55) and non-terminal enecarbamates (56–60). These results diverges from the one obtained by Gillaizeau *et al.* who have obtained the product of non-reductive alkylation by performing the reaction between xanthates and enamides in the presence of dilauroyl peroxide acting as a radical initiator and oxidant,^46^ demonstrating the mildness and non-oxidative character of the triethylborane-involved initiation process.

**Scheme 10:**
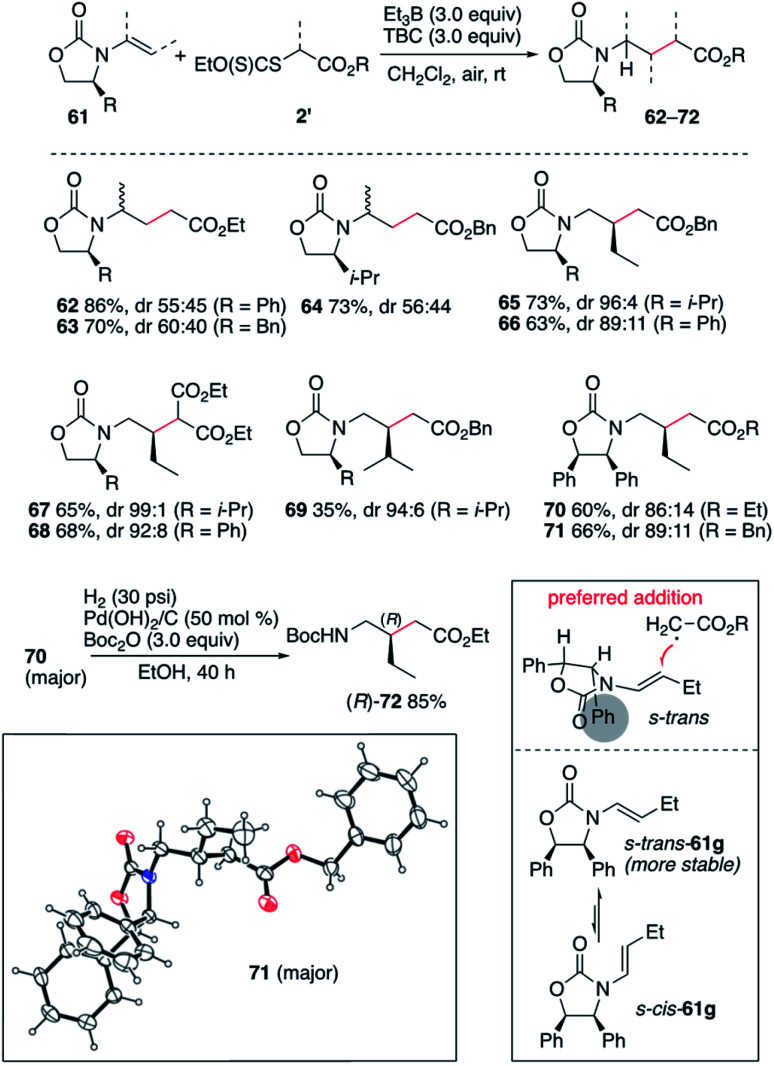
Stereoselective radical hydroalkylation of chiral enecarbamates. Single crystal X-ray structure of 71 (ellipsoids drawn at 50% probability).

The efficient hydroalkylation of *N*-alkenyloxazolidinones reported in Scheme 9 offers the possibility of controlling the stereoselectivity of the process by using enecarbamates 61 derived from chiral oxazolidinones.^47–51^ Reactions involving the terminal 1-substituted *N*-prop-1-en-2-yloxazolidinones provided compounds 62–64 in high yield but poor stereocontrol. Fortunately, reactions involving the non-terminal enecarbamates proceeded with good to high diastereocontrol as illustrated by the formation of compounds 65–71. These results are in agreement with results obtained for the hydroamination of similar enecarbamates.^48^ The highest diastereoselectivity being observed for the 4-isopropyloxazolidin-2-ones leading to 65, 67, and 69 with dr ranging from 94 : 6 to 99 : 1. Reactions involving 4-phenyloxazoldin-2-one provided 66, 68, 70 and 71 with slightly lower diastereoselectivities varying from 86 : 14 to 92 : 8. The relative configuration of 71 was confirmed by single crystal X-ray crystallography of the major diastereomer (Scheme 10).^33–37^ The stereochemical outcome of the process can be rationalized by radical addition from the less hindered face (anti to the 4-phenyl substituent) of the enecarbamate lying in its most stable s-*trans* conformation as proposed by Studer and coworkers for the related hydroamination process.^48,52,53^ Interestingly, the diphenyloxazolidinone derivative 70 was easily converted to the corresponding enantiomerically pure protected γ-amino acid (*R*)-72 under mild hydrogenolysis conditions.^54^

## Conclusions

We have developed a general and operationally simple radical chain process for the hydroalkylation of electron-rich terminal and non-terminal alkenes with α-iodo- and α-xanthylesters. The reaction is initiated with triethylborane and air while the inexpensive and non-toxic TBC is used as a source of hydrogen atom. Highly diastereoselective hydroalkylation was also achieved by using chiral enecarbamates, providing access to chiral γ-amino acid derivatives.

## Conflicts of interest

There are no conflicts to declare.

## Supplementary Material

SC-012-D0SC06341J-s001

SC-012-D0SC06341J-s002
