# Supplementary material for: A Giese reaction for electron-rich alkenes
Source: Chem Sci. 2020 Dec 17;12(6):2225–30. doi: 10.1039/d0sc06341j (PMC8179299; doi:10.1039/d0sc06341j)

Electronic Supplementary Material (ESI) for Chemical Science.
This journal is © The Royal Society of Chemistry 2020

Supporting Information

A Giese Reaction for Electron-Rich Alkenes

Qi Huang, Sankar Rao Suravarapu, and Philippe Renaud*

Table of Contents

1 General Information *3*

1.1 Instrumentation **3**

1.2 Materials **3**

2 List of Substrates *5*

2.1 List of Alkenes **5**

2.2 List of Radical Precursors **6**

3 General Procedures *7*

4 Experimental Procedures and Spectroscopic Data *8*

4.1 Preparation of Substrates **8**

4.2 Radical Addition Products **12**

4.3 Modification of Radical Adducts **45**

5 Spectra *48*

1 General Information

All the glassware was oven-dried or flame dried under vacuum, cooled under vacuum, and back-filled with nitrogen. Unless otherwise stated, all reactions were performed under nitrogen atmosphere. For flash column chromatography silica gel 60 Å (230–400 mesh particle size) was used. Thin layer chromatography (TLC) was performed using *Macherey-Nagel* ALUGRAM®Xtra SIL G/UV254, 0.2 mm silica gel; visualization under UV light (254 nm) and/or by dipping in a solution of $(NH_4)_2MoO_4$ (15.0 g), $Ce(SO_4)_2$ (0.5 g), H_2O (90 mL), conc. H_2SO_4 (10 mL); or $KMnO_4$ (3 g), K_2CO_3 (20 g) and NaOH 5% (3 mL) in H_2O (300 mL) and subsequent heating.

1.1 Instrumentation

^{1}H and ^{13}C NMR spectra were recorded on a Bruker Avance III HD-300 spectrometer operating at 300 MHz for ^{1}H and 75 MHz for ^{13}C at rt (24-25 °C) or on a Bruker Avance III HD-400 or a Bruker Avance II-400 spectrometer (^{1}H: 400 MHz; ^{13}C: 101 MHz) unless otherwise stated. Chemical shifts (δ) were reported in parts per million with the residual solvent peak used as an internal standard ($CHCl_3$: δ = 7.26 ppm and CD_3CN: δ = 1.94 ppm for ^{1}H NMR spectra and $CDCl_3$: δ = 77.0 ppm and CD_3CN: δ = 1.32 ppm for ^{13}C NMR spectra). The following abbreviations were used to explain the multiplicities: s (singlet), d (doublet), t (triplet), q (quadruplet), quint (quintet), sept (septuplet) m (multiplet), br (broad), the prefix app (apparent) was added when different coupling constants appeared accidentally equal. Coupling constants, *J*, are reported in Hz and with an accuracy of one unit of the last digit. HRMS analyses were recorded on an Applied Biosystems Sciex QSTAR Pulsar (hybrid quadrupole time-of-flight mass spectrometer) using positive electron spray. Infrared spectra were recorded on a Jasco FT-IR-460 plus spectrometer equipped with a Specac MKII Golden Gate Single Reflection Diamond ATR system and are reported in wave numbers (cm^{-1}). Gas chromatography (GC) analyses were performed on a spectrometer fitted with a *Macherey-Nagel* Optima delta-3-0.25 µm capillary column (20 m, 0.25 mm); gas carrier He 1.4 mL/min; injector: 220 °C split mode; detector: FID 280 °C, H_2 35 mL/min, air 350 mL/min.

1.2 Materials

Unless otherwise stated, all commercial reagents were used as received. Solvents for the reactions (CH_2Cl_2, THF and n-Hexane) were filtered over columns of dried alumina under a positive pressure of argon. Solvents for extractions and flash chromatography were of technical grade and

were distilled prior to use. Triethylborane solution (1 M in n-hexane) was prepared from pure triethylborane.

2 List of Substrates

2.1 List of Alkenes

enol ester

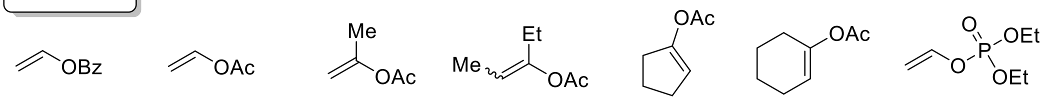

1a **1b** **1c** **1d** **1e** **1f** **1g**

alkenyl sulfide

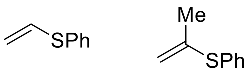

17a **17b**

enol ether

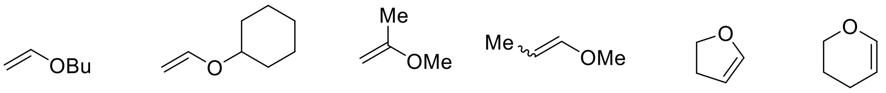

26a **26b** **26c** **26d** **26e** **26f**

silyl enol ether

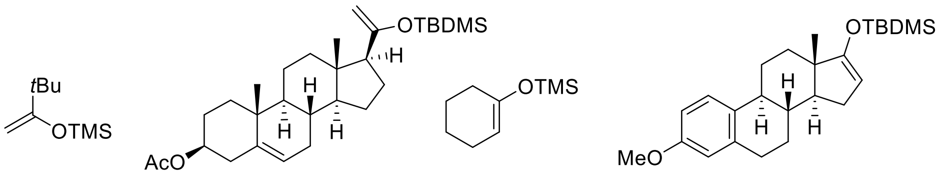

37a **37b** **37c** **37d**

enamide and enecarbamate

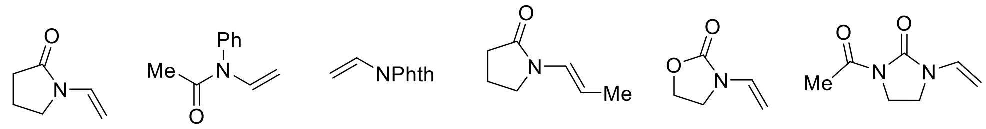

44a **44b** **44c** **44d** **44e** **44f**

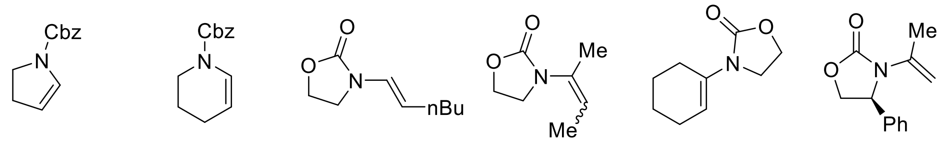

44g **44h** **44i** **44j** **44k** **61a**

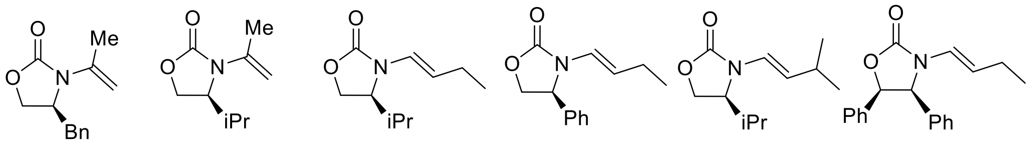

61b **61c** **61d** **61e** **61f** **61g**

2.2 List of Radical Precursors

halide	xanthate	halide	xanthate
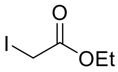 **2a**	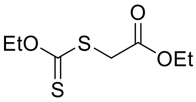 **2'a**	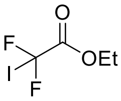 **2f**	*n.a.*
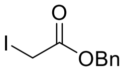 **2b**	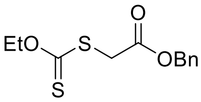 **2'b**	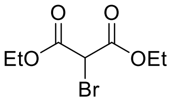 **2g**	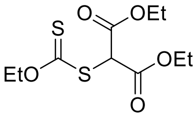 **2'g**
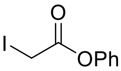 **2c**	*n.a.*	*n.a.*	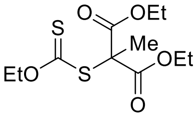 **2'h**
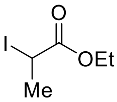 **2d**	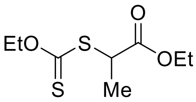 **2'd**	*n.a.*	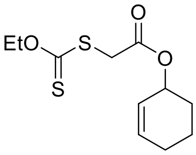 **2'i**
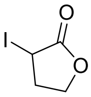 **2e**	*n.a.*		

3 General Procedures

General Procedure A

To a solution of α-iodoester (1.0 equiv), alkene (2.0-5.0 equiv) in CH_2Cl_2 (10 mL/0.5 mmol of iodide) was added 4-*tert*-butylcatechol (3.0 equiv) followed by Et_3B (1.2 equiv, 1 M solution in *n*-hexane) while the needle was immersed in the solution. The resulting solution was stirred at room temperature in the presence of air under $CaCl_2$ guard tube. Consumption of the starting material was monitored by GC or TLC. Upon completion, the reaction mixture was filtered over a short pad of neutral alumina and was washed with Et_2O to trap catechol derivatives and boron-containing side products. The resulting crude filtrate was concentrated under reduced pressure and flash column chromatography on silica gel gave the desired product.

General Procedure B

To a solution of α-xanthate ester (1.0 equiv), alkene (2.0-3.0 equiv) in CH_2Cl_2 (10 mL/1.0 mmol of xanthate) was added 4-*tert*-butylcatechol (3.0 equiv) followed by Et_3B (2.5-3.0 equiv, 1 M solution in *n*-hexane) while the needle was immersed in the solution. The resulting solution was stirred at room temperature in the presence of air under $CaCl_2$ guard tube. Consumption of the starting material was monitored by GC or TLC. Upon completion, the reaction mixture was filtered over a short pad of neutral alumina and was washed with Et_2O or EtOAc to trap catechol derivatives and boron-containing side products. The resulting crude filtrate was concentrated under reduced pressure and flash column chromatography on silica gel gave the desired product.

General Procedure C

To a solution of α-iodoester (1.0 mmol) and enamide (5.0 mmol) in dichloromethane was added triethylborane (1.2 mmol, 1.2 mL, 1M in hexane) while the needle was immersed in the solution. The resulting solution was stirred open to air under $CaCl_2$ guard tube for 3 h. The resulting solution was stirred at room temperature in presence of air under $CaCl_2$ guard tube. Consumption of starting material was monitored by TLC. Upon completion, reaction mixture was directly concentrated under reduced pressure and flash column chromatography on silica gel gave the desired product.

4 Experimental Procedures and Spectroscopic Data

4.1 Preparation of Substrates

Cyclohex-2-enyl 2-(ethoxycarbonothioylthio) acetate (2'i)

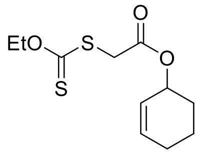

Chemical Formula: $C_{11}H_{16}O_3S_2$
Molecular Weight: 260,37

To a solution of 1-clohex-2-enyl 2-chloroacetate[1] (2.2 g, 12.6 mmol, 1.0 equiv) in acetone (12.6 mL) was added portionwise potassium ethyl xanthogenate (2.4 g, 15.1 mmol, 1.2 equiv) at room temperature. The resulting solution was stirred for 4 h and then water was added. The mixture was then extracted with Et_2O (3 x 100 mL), washed with brine, dried over Na_2SO_4, filtered and concentrated under reduced pressure. Purification by flash column chromatography on silica gel (pentane/Et_2O 95:05) gave the desired product as a pale yellowish green oil (2.8 g, 10.8 mmol, 85%). ^{1}H NMR (300 MHz, $CDCl_3$) δ (ppm) 5.96 – 5.87 (m, 1H), 5.65 (ddt, J = 10.0, 4.0, 2.1 Hz, 1H), 5.30 – 5.18 (m, 1H), 4.57 (q, J = 7.1 Hz, 2H), 3.84 (s, 2H), 2.13 – 1.86 (m, 2H), 1.84 – 1.63 (m, 3H), 1.64-1.5 (m, 1H), 1.35 (t, J = 7.1 Hz, 3H). ^{13}C NMR (75 MHz, $CDCl_3$) δ (ppm) 212.8, 167.6, 133.5, 125.1, 70.7, 69.9, 38.3, 28.2, 25.0, 18.8, 13.8. HRMS (ESI) Calcd. For $C_{11}H_{16}O_3NaS_2$: 283.0433 $[M+Na]^+$, Found: 283.0438.

1-Acetyl-3-vinylimidazolidin-2-one (44f)

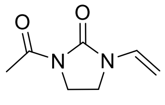

Chemical Formula: $C_7H_{10}N_2O_2$
Molecular Weight: 154,17

To an oven-dried screw-cap vial fitted with magnetic stir bar was added *N*-acetylimidazolidin-2-one[2] (641 mg, 5.00 mmol, 1.0 equiv), CuI (47 mg, 0.25 mmol, 0.05 equiv), *N,N'*-dimethylethylenediamine (55 µL, 0.50 mmol, 0.10 equiv), K_2CO_3 (1.38 g, 10.00 mmol, 2.0 equiv) and vinyl bromide (1 M in THF, 10.0 mL, 10.00 mmol, 2.0 equiv). The vial was tightly sealed using Teflon septum and heated to 90 °C for 24 h. The mixture was filtered through a pad of Celite and washed with EtOAc. The filtrate was concentrated under reduced pressure and the residue was purified by flash chromatography on silica gel (ethyl acetate/heptane = 2:3) to give the desired product **44f** as a white solid (600 mg, 3.89 mmol, 78% yield). ^{1}H NMR (300 MHz, CD_3CN) δ (ppm) 6.94 (dd, J = 15.9, 9.1 Hz, 1H), 4.46 – 4.32 (m, 2H), 3.84 – 3.74 (m, 2H), 3.56 – 3.46 (m, 2H), 2.40 (s, 3H). ^{13}C NMR (75 MHz, CD_3CN) δ (ppm) 171.1, 153.4, 130.6, 93.4, 40.3, 39.2, 23.8. IR (neat, cm^{-1}): 2972, 2911, 1722, 1677, 1630, 1474, 1430, 1393, 1360, 1302, 1274,

---

[1] Soulard, V.; Villa, G.; Vollmar, D. P.; Renaud, P. *J. Am. Chem. Soc.* **2018**, *140*, 155.

[2] Hall Jr., H. K.; Schneider, A. K. *J. Am. Chem. Soc.* **1958**, *80*, 6409.

1257, 1209, 1173, 1101, 1035, 1000, 975, 964, 924, 860, 842, 744, 609, 598, 587, 441. HRMS (ESI) Calcd for $C_7H_{11}O_2N_2$: 155.0815 $[M+H]^+$, Found: 155.0809. mp: 52.3-53.8 °C.

3-(Cyclohex-1-en-1-yl)oxazolidin-2-one (44k)

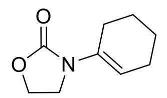

Chemical Formula: $C_9H_{13}NO_2$
Molecular Weight: 167,21

A mixture of oxazolidin-2-one (871 mg, 10.0 mmol, 1.0 equiv), cyclohexanone (1.96 g, 20.0 mmol, 2.0 equiv), and (*d,l*)-camphorsulfonic acid (232 mg, 1.0 mmol, 0.1 equiv) in toluene was refluxed for 15 h with Dean-Stark set up to remove water. The organic layer was successively washed with $NaHCO_3$ and dried with Na_2SO_4. Flash chromatography on silica gel (TBME/pentane = 3:2) gave the desired product **44k** as a white solid (121 mg, 0.72 mmol, 7% yield). The spectra data are in agreement with the literature report.[3] ^{1}H NMR (300 MHz, CD_3CN) δ (ppm) 5.33 – 5.29 (m, 1H), 4.30 – 4.19 (m, 2H), 3.76 – 3.65 (m, 2H), 2.47 – 2.41 (m, 2H), 2.13 – 2.05 (m, 2H), 1.73 – 1.62 (m, 2H), 1.62 – 1.51 (m, 2H). ^{13}C NMR (75 MHz, CD_3CN) δ (ppm) 156.2, 136.4, 112.3, 62.5, 463, 26.5, 24.8, 23.4, 22.7. IR (neat, cm^{-1}): 2931, 2909, 2852, 2833, 1724, 1651, 1478, 1405, 1345, 1321, 1286, 1258, 1223, 1171, 1111, 1078, 1046, 1001, 909, 845, 832, 791, 752, 705. HRMS (ESI) Calcd for $C_9H_{14}O_2N$: 168.1019 $[M+H]^+$, Found: 168.1015. mp: 42.8-44.7 °C.

(*S*)-4-Phenyl-3-(prop-1-en-2-yl) oxazolidin-2-one (61a)

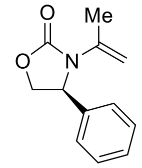

Chemical Formula: $C_{12}H_{13}NO_2$
Molecular Weight: 203,24

CuI (116 mg, 0.61 mmol, 0.05 equiv), (*S*)-4-phenyloxazolidin-2-one (2.00 g, 12.20 mmol, 1.0 equiv), potassium carbonate (3.37 mg, 24.4 mmol, 2.0 equiv) were charged successively into a two neck round bottom flask under nitrogen atmosphere. Then *N, N'*-dimethylethylenediamine (130 µL, 107.3 mg, 1.22 mmol, 0.1 equiv), 2-bromoprop-1-ene (2.94 g, 24.40 mmol, 2.0 equiv), toluene (12.0 mL) were added. Then the reaction mixture was heated to reflux for 2 days. Upon completion, the reaction mixture was cooled down to room temperature and filtered over a short pad of silica and washed with ether. The filtrate was concentrated under reduce pressure to give the crude mixture. Flash chromatography on silica gel with 10-35% ether in pentane afforded the desired product **61a** as a white solid (1.40 g, 6.89 mmol, 56%). ^{1}H NMR (300 MHz, $CDCl_3$) δ (ppm) 7.44 – 7.31 (m, 3H), 7.30 – 7.24 (m, 2H), 5.09 (dd, J = 8.8, 5.6 Hz, 1H), 4.65 (t, J = 8.7 Hz, 1H), 4.42 (d, J = 4.3 Hz, 2H), 4.07 (dd, J = 8.6, 5.6 Hz, 1H), 2.11 (d, J = 0.9 Hz, 3H). ^{13}C NMR (75 MHz, $CDCl_3$) δ (ppm) 155.5, 139.0, 138.9, 129.3, 128.7, 125.9, 101.9, 69.6, 60.6, 20.5. HRMS (ESI) Calcd. for $C_{12}H_{14}O_2N$: 204.1019 $[M+H]^+$, Found: 204.1018.

---

[3] Pan, X.; Cai, Q.; Ma, D. *Org. Lett.* **2004**, *6*, 1809.

(*S*)-4-Benzyl-3-(prop-1-en-2-yl) oxazolidin-2-one (61b)

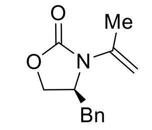

Chemical Formula: $C_{13}H_{15}NO_2$
Molecular Weight: 217,27

CuI (47.6 mg, 0.25 mmol, 0.05 equiv), (S)-4-benzyloxazolidin-2-one (885 mg, 5.0 mmol, 1.0 equiv), potassium carbonate (1350.0 mg, 10.0 mmol, 2.0 equiv) were charged successively into a two neck round bottom flask under nitrogen atmosphere. Then *N,N'*-dimethylethylenediamine (55.0 μL, 44.0 mg, 0.5 mmol, 0.1 equiv), 2-bromoprop-1-ene (900.0 mg, 7.5 mmol, 1.5 equiv), toluene (5.0 mL) were added. Then the reaction mixture was heated to reflux for 28 h. Upon completion, the reaction mixture was cooled down to room temperature and filtered over a short pad of silica and washed with ether. The filtrate was concentrated under reduce pressure to give the crude mixture. Flash chromatography on silica gel with 10-35% ether in pentane afforded the desired product **61b** as a white solid (920 mg, 4.23 mmol, 85%). ^{1}H NMR (300 MHz, $CDCl_3$) δ (ppm) 7.38 – 7.23 (m, 3H), 7.20 – 7.13 (m, 2H), 4.74 (s, 1H), 4.68 (d, J = 0.6 Hz, 1H), 4.36 – 4.25 (m, 1H), 4.19 (t, J = 8.2 Hz, 1H), 4.09 (dd, J = 8.7, 4.0 Hz, 1H), 3.21 (dd, J = 13.8, 3.2 Hz, 1H), 2.75 (dd, J = 13.8, 9.3 Hz, 1H), 2.20 (d, J = 0.9 Hz, 3H). ^{13}C NMR (75 MHz, $CDCl_3$) $\boldsymbol{\delta}$ (ppm) 154.9, 139.1, 135.6, 129.3, 129.0, 127.3, 101.3, 65.8, 57.0, 37.2, 20.6. HRMS (ESI) Calcd. for $C_{13}H_{16}O_2N$: 218.1176 $[M+H]^+$, Found: 218.1174.

(*S*)-4-Isopropyl-3-(prop-1-en-2-yl)oxazolidin-2-one (61c)

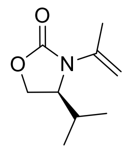

Chemical Formula: $C_9H_{15}NO_2$
Molecular Weight: 169,22

To an oven-dried screw-cap vial fitted with magnetic stirring bar was added (*S*)-4-isopropyloxazolidin-2-one (1.29 g, 10.00 mmol, 1.0 equiv), 2-bromopropene (1.78 mL, 20.00 mmol, 2.0 equiv), CuI (95 mg, 0.50 mmol, 0.05 equiv), *N,N*'-dimethylethylenediamine (110 μL, 1.00 mmol, 0.10 equiv), K_2CO_3 (2.76 g, 20.00 mmol, 2.0 equiv) and THF (10 mL). The vial was tightly sealed using Teflon septum and heated to 90 °C for 44 h. The mixture was filtered through a pad of Celite and washed with EtOAc. The filtrate was concentrated under reduced pressure and the residue was purified by flash chromatography on silica gel (ethyl acetate/heptane = 3:7) to give the desired product **61c** as a colorless oil (1.63 g, 9.63 mmol, 96% yield). ^{1}H NMR (300 MHz, CD_2Cl_2) δ 4.73 – 4.66 (m, 2H), 4.28 – 4.19 (m, 1H), 4.14 – 4.02 (m, 2H), 2.22 (ddp, J = 10.2, 7.0, 3.2 Hz, 1H), 2.08 (d, J = 1.3 Hz, 3H), 0.87 (t, J = 7.1 Hz, 6H). ^{13}C NMR (75 MHz, CD_2Cl_2) δ (ppm) 139.3, 103.3, 62.7, 60.3, 27.8, 20.5, 17.9, 14.5. IR (neat, cm^{-1}): 2963, 2876, 1743, 1363, 1402, 1321, 1215, 1182, 1160, 1119, 1060, 982, 859, 836, 768, 753, 728, 645, 633. HRMS (ESI) Calcd for $C_9H_{16}O_2N$: 170.1176 $[M+H]^+$, Found: 170.1169. $[\alpha]_D$ = -42.4 (c = 0.224, $CHCl_3$).

(*S,E*)-3-(But-1-en-1-yl)-4-phenyloxazolidin-2-one (61e)

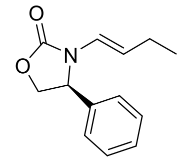

Chemical Formula: $C_{13}H_{15}NO_2$
Molecular Weight: 217,27

A catalytic amount of *p*-toluenesulfonic acid and (*S*)-4-phenyloxazolidin-2-one (1.63 g, 10.0 mmol, 1.0 equiv) was added to a stirred solution of butanal (793 mg, 11.0 mmol, 1.1 equiv) in benzene (50 mL). The reaction mixture was fitted with a Dean-Stark trap and condenser. The solution was heated to reflux over 20 h. The reaction mixture was cooled to room temperature and concentrated under reduced pressure. The crude material was purified by flash chromatography on silica gel (gradient of diethyl ether/pentane = 2:5 to 1:2) to afford the desired chiral product **61e** as a white solid (1.26 g, 5.8 mmol, 58% yield). ^{1}H NMR (300 MHz, $CDCl_3$) δ (ppm) 7.46 – 7.31 (m, 3H), 7.29 – 7.22 (m, 2H), 6.58 (dt, *J* = 14.5, 1.5 Hz, 1H), 5.00 (dd, *J* = 9.0, 5.3 Hz, 1H), 4.75 – 4.62 (m, 2H), 4.11 (dd, *J* = 8.6, 5.3 Hz, 1H), 1.99 – 1.84 (m, 2H), 0.86 (t, *J* = 7.4 Hz, 3H). ^{13}C NMR (75 MHz, $CDCl_3$) δ (ppm) 156.0, 138.5, 129.4, 128.8, 126.0, 122.1, 115.4, 70.6, 58.8, 23.3, 14.2. IR (neat, cm^{-1}): 2962, 2916, 2872, 1742, 1670, 1457, 1403, 1320, 1214, 1130, 1073, 1038, 942, 754, 698. HRMS (ESI) Calcd for $C_{13}H_{16}O_2N$: 218.1176 $[M+H]^+$, Found: 218.1175. mp: 38.6-38.8 °C. $[\alpha]_D$ = 106.2 (c = 0.416, $CHCl_3$).

(*4S*,*5R*)-3-((*E*)-But-1-en-1-yl)-4,5-diphenyloxazolidin-2-one (61g)

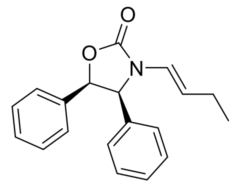

Chemical Formula: $C_{19}H_{19}NO_2$
Molecular Weight: 293,37

A catalytic amount of *p*-toluenesulfonic acid and (*4S, 5R*)-4,5-diphenyloxazolidin-2-one (622 mg, 2.60 mmol, 1.0 equiv) was added to a stirred solution of butanal (206 mg, 2.86 mmol, 1.1 equiv) in benzene (25 mL). The reaction mixture was fitted with a Dean-Stark trap and condenser. The solution was heated to reflux over 16 h. The reaction mixture was cooled to room temperature and concentrated under reduced pressure. The crude material was purified by flash chromatography on silica gel (gradient of diethyl ether/pentane = 1:4 to 1:3) to afford the desired chiral enamine **61g** as a white solid (358 mg, 1.22 mmol, 47% yield). ^{1}H NMR (300 MHz, $CDCl_3$) δ (ppm) 7.15 – 7.02 (m, 6H), 6.97 (tt, *J* = 4.7, 2.4 Hz, 2H), 6.82 (qd, *J* = 4.0, 1.7 Hz, 2H), 6.68 (dt, *J* = 14.5, 1.5 Hz, 1H), 5.89 (d, *J* = 8.1 Hz, 1H), 5.21 (d, *J* = 8.2 Hz, 1H), 4.67 (dt, *J* = 14.5, 6.7 Hz, 1H), 1.93 (pd, *J* = 7.4, 1.5 Hz, 2H), 0.86 (t, *J* = 7.4 Hz, 3H). ^{13}C NMR (75 MHz, $CDCl_3$) δ (ppm) 155.6, 134.0, 133.7, 128.34, 128.30, 128.2, 128.0, 127.2, 126.5, 122.0, 115.8, 80.5, 63.9, 23.2, 14.2. IR (neat, cm^{-1}): 2969, 2955, 2920, 2868, 1729, 1673, 1456, 1409, 1375, 1361, 1341, 1247, 1214, 1138, 1095, 1074, 1034, 1025, 1014, 940, 885, 828, 760, 716, 692, 568. HRMS (ESI) Calcd for $C_{19}H_{20}O_2N$: 294.1489 $[M+H]^+$, Found: 294.1482. mp: 155.1-157.3 °C. $[\alpha]_D$ = -20.0 (c = 0.416, dichloromethane).

4.2 Radical Addition Products

4-Ethoxy-4-oxobutyl benzoate (3)

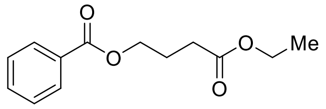

Chemical Formula: $C_{13}H_{16}O_4$
Molecular Weight: 236,27

Following general procedure A, the reaction was carried out with ethyl iodoacetate **2a** (107 mg, 0.50 mmol), vinyl benzoate **1a** (370 mg, 0.35 mL, 2.50 mmol), 4-*tert*-butylcatechol (498 mg, 3.00 mmol), Et_3B (0.6 mL, 0.60 mmol). Flash column chromatography on silica gel (pentane/Et_2O = 88:12) gave the desired product **3** as a colorless oil (75%). ^{1}H NMR (300 MHz, $CDCl_3$) δ (ppm) 8.10-7.96 (m, 2H), 7.60-7.50(m, 1H), 7.48-7.38 (m, 2H), 4.36 (t, J = 6.3 Hz, 2H), 4.12 (q, J = 7.11 Hz, 2H), 2.48 (t, J = 7.3 Hz, 2H), 2.11 (quin, 2H), 1.24 (t, J = 7.1 Hz, 3H). ^{13}C NMR (75 MHz, $CDCl_3$) δ (ppm) 172.9, 166.5, 133.0, 130.2, 129.6, 128.4, 64.0, 60.6, 31.0, 24.2, 14.2. IR (neat, cm^{-1}): 3066, 2980, 2898, 1716, 1270, 1174, 1111, 1070, 1025, 708. HRMS (ESI) Calcd. for $C_{13}H_{17}O_4$: 237.1121 $[M+H]^+$, Found: 237.1124.

4-(Benzyloxy)-4-oxobutyl benzoate (4)

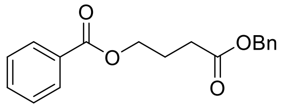

Chemical Formula: $C_{18}H_{18}O_4$
Molecular Weight: 298,34

Following general procedure A, the reaction was carried out with benzyl 2-iodoacetate **2b** (139 mg, 0.50 mmol), vinyl benzoate **1a** (370 mg, 0.35 mL, 2.50 mmol), 4-*tert*-butylcatechol (499 mg, 3.00 mmol), Et_3B (0.6 mL, 0.60 mmol). Flash column chromatography on silica gel (pentane/Et_2O = 90:10) gave the desired product **4** as a colorless oil (121 mg, 81% yield). Spectral data are in accordance with the literature report.[4] ^{1}H NMR (300 MHz, $CDCl_3$) δ (ppm) 7.98-7.91 (m, 2H), 7.51-7.43 (m, 1H), 7.39-7.31 (m, 2H), 7.29-7.22 (m, 5H), 5.03, (s, 2H), 4.29 (t, J = 6.3 Hz, 2H), 2.47 (t, J =7.4 Hz, 2H), 2.06 (quin, 2H). ^{13}C NMR (75 MHz, $CDCl_3$) δ (ppm) 172.7, 166.5, 135.8, 133.0, 130.2, 129.6, 128.6, 128.4, 128.3, 128.3 66.5, 63.9, 31.0, 24.2. HRMS (ESI) Calcd. for $C_{18}H_{19}O_4$: 299. 1278 $[M+H]^+$, Found: 299.1274.

4-Oxo-4-phenoxybutyl benzoate (5)

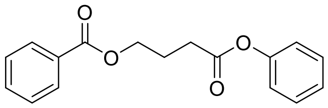

Chemical Formula: $C_{17}H_{16}O_4$
Molecular Weight: 284,31

Following general procedure A, the reaction was carried out with phenyl 2-iodoacetate **2c** (131 mg, 0.50 mmol), vinyl benzoate **1a** (370 mg, 0.35 mL, 2.50 mmol), 4-*tert*-butylcatechol (498 mg, 3.00 mmol), Et_3B (0.6 mL, 0.60 mmol). Flash column chromatography on silica gel (pentane/Et_2O = 90:10) gave the desired product **5** as a colorless oil (90 mg, 64% yield). ^{1}H NMR (300 MHz, $CDCl_3$) δ (ppm) 8.12 – 8.01 (m, 2H), 7.61 – 7.53 (m, 1H), 7.52 – 7.30 (m, 4H), 7.26 – 7.17 (m, 1H), 7.13 – 7.04 (m, 2H), 4.46 (t, J = 6.2 Hz, 2H), 2.76

---

[4] Studer, A.; Amrein, S. *Angew. Chem. Int. Ed.* **2000**, *39*, 3080.

(t, J = 7.3 Hz, 2H), 2.34 – 2.15 (m, 2H). ^{13}C NMR (75 MHz, $CDCl_3$) δ (ppm) 171.4, 166.6, 150.6, 133.1, 130.1, 129.7, 129.5, 128.4, 125.9, 121.6, 63.8, 31.1, 24.2. IR (neat, cm^{-1}): 2962, 1754, 1714, 1592, 1491, 1451, 1314, 1269, 1194, 1138, 1110, 1069, 1025, 936, 809, 751, 708, 687. HRMS (ESI) Calcd. for $C_{17}H_{17}O_4$: 285.1121 $[M+H]^+$, Found : 285.1116.

Benzyl 4-acetoxybutanoate (6)

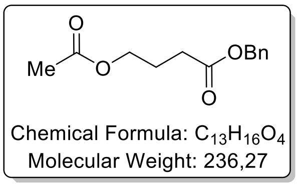

Following general procedure A, the reaction was carried out with benzyl 2-iodoacetate **2b (**139 mg, 0.50 mmol), vinyl acetate **1b** (215.2 mg, 0.23 mL, 2.50 mmol), 4-*tert*-butylcatechol (498 mg, 3.00 mmol), Et_3B (0.6 mL, 0.60 mmol). Flash column chromatography on silica gel (pentane/Et_2O = 80:20) gave the desired product **6** as a colorless oil (91 mg, 77% yield). ^{1}H NMR (300 MHz, $CDCl_3$) δ (ppm) 7.32-7.23 (m, 5H), 5.05 (s, 2H), 4.03 (t, J = 6.3 Hz, 2H), 2.38 (t, J = 7.4 Hz, 2H), 1.95 (s, 3H), 1.92 (quin, 2H). ^{13}C NMR (75 MHz, $CDCl_3$) δ (ppm) 172.7, 171.0, 135.9, 128.6, 128.3, 128.2, 66.4, 63.4, 30.8, 24.0, 20.9. IR (neat, cm^{-1}): 2956, 2360, 1731, 1455, 1366, 1230, 1162, 1042, 800, 737, 697, 631, 620, 605. HRMS (ESI) Calcd. for $C_{13}H_{17}O_4$: 237.1121 $[M+H]^+$, Found : 237.1123.

Phenyl 4-acetoxybutanoate (7)

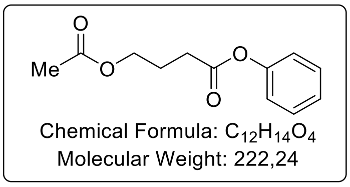

Following general procedure A, the reaction was carried out with phenyl 2-iodoacetate **2c (**131 mg, 0.50 mmol), vinyl acetate **1b** (215 mg, 0.23 mL, 2.50 mmol), 4-*tert*-butylcatechol (498 mg, 3.00 mmol), Et_3B (0.6 mL, 0.60 mmol). Flash column chromatography on silica gel (pentane/Et_2O = 80:20) gave the desired product **7** as a colorless oil (70 mg, 0.32 mmol, 63%). ^{1}H NMR (300 MHz, $CDCl_3$) δ (ppm) 7.44 – 7.30 (m, 2H), 7.30 – 7.17 (m, 1H), 7.09 (dt, J = 8.4, 1.2 Hz, 2H), 4.25 – 4.10 (m, 2H), 2.74 – 2.57 (m, 2H), 2.19 – 2.00 (m, 2H), 2.07 (s. 3H). ^{13}C NMR (75 MHz, $CDCl_3$) δ (ppm) 171.4, 171.1, 150.6, 129.5, 125.9, 121.5, 63.3, 31.0, 24.1, 20.9. IR (neat, cm^{-1}): 2962, 1734, 1592, 1493, 1364, 1231, 1192, 1137, 1041, 931, 810, 751, 689. HRMS (ESI) Calcd. for $C_{12}H_{15}O_4$: 223.0965 $[M+H]^+$, Found : 223.0961.

4-Ethoxy-3-methyl-4-oxobutyl benzoate (8)

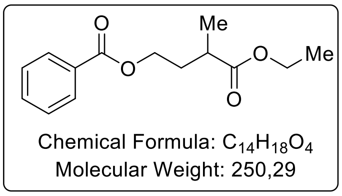

Following general procedure A, the reaction was carried out with ethyl 2-iodopropanoate **2d (**114 mg, 0.50 mmol), vinyl benzoate **1a** (370 mg, 0.35 mL, 2.50 mmol), 4-*tert*-butylcatechol (498 mg, 3.00 mmol), Et_3B (0.6 mL, 0.60 mmol). Flash column chromatography on silica gel (pentane/Et_2O = 90:10) gave the desired product **8** as a colorless oil (64 mg, 0.26 mmol, 51% yield). ^{1}H NMR (300 MHz, $CDCl_3$) δ (ppm) 8.03-7.92 (m, 2H),

7.54-7.44 (m, 1H), 7.43-7.32 (m, 2H), 4.29 (td, J = 6.4, 1.3 Hz, 2H), 4.04 (qd, J = 7.3, 1.38 Hz, 2H), 2.59 (sex, 1H), 2.21-2.05 (m, 1H), 1.90-1.74 (m, 1H), 1.15 (t, J = 7.1 Hz, 3H), 1.18 (d, J = 7.1 Hz, 3H). ^{13}C NMR (75 MHz, $CDCl_3$) δ (ppm) 175.9, 166.5, 132.9, 130.2, 129.6, 128.3, 62.8, 60.5, 36.7, 32.4, 17.2, 14.2. IR (neat, cm^{-1}): 2976, 1716, 1601, 1451, 1376, 1314, 1269, 1176, 1139, 1109, 1070, 1025, 858, 806, 708. HRMS (ESI) Calcd. for $C_{14}H_{19}O_4$: 251.1278 [M+H]$^+$, Found: 251.1281.

2-(2-Oxotetrahydrofuran-3-yl)ethyl benzoate (9)

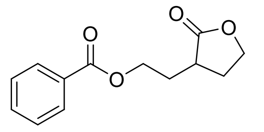

Chemical Formula: $C_{13}H_{14}O_4$
Molecular Weight: 234,25

Following general procedure A, the reaction was carried out with 3-iododihydrofuran-2(3*H*)-one **2e** **(**106 mg, 0.50 mmol), vinyl benzoate **1a** (370 mg, 0.35 mL, 2.50 mmol), 4-*tert*-butylcatechol (500 mg, 3.00 mmol), Et_3B (0.6 mL, 0.60 mmol). Flash column chromatography on silica gel (pentane/Et_2O = 65:35) gave the desired product **9** as a colorless oil (60 mg, 0.26 mmol, 52% yield). ^{1}H NMR (300 MHz, $CDCl_3$) δ (ppm) 7.99-7.93 (m, 2H), 7.55-7.47 (m, 1H), 7.43-7.34 (m, 2H), 4.40 (t, J = 6.4 Hz, 2H), 4.32 (td, J = 8.9, 2.3 Hz, 1H), 4.14 (ddd, J =10.1, 9.3, 6.5 Hz, 1H), 2.66 (dtd, J = 10.8, 8.9, 4.8 Hz, 1H), 2.51-2.28(m, 2H), 2.08-1.78 (m, 2H). ^{13}C NMR (75 MHz, $CDCl_3$) δ (ppm) 178.6, 166.4, 133.1, 129.9, 129.6, 128.5, 66.5, 62.6, 36.8, 29.6, 28.8. IR (neat, cm^{-1}): 2962, 2910, 1765, 1712, 1601, 1451, 1375, 1314, 1269, 1211, 1160, 1107, 1070, 1023, 938, 807, 709, 687. HRMS (ESI) Calcd. for $C_{13}H_{15}O_4$: 235.0965 [M+H]$^+$, Found : 235.0966.

4-Ethoxy-3,3-difluoro-4-oxobutyl benzoate (10)

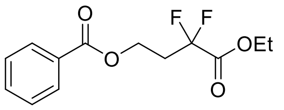

Chemical Formula: $C_{13}H_{14}F_2O_4$
Molecular Weight: 272,25

Following general procedure A, the reaction was carried out with ethyl 2,2-difluoro-2-iodoacetate **2f** **(**125 mg, 0.50 mmol), vinyl benzoate **1a** (370 mg, 0.35 mL, 2.50 mmol), 4-*tert*-butylcatechol (498 mg, 3.00 mmol), Et_3B (0.6 mL, 0.60 mmol). Flash column chromatography on silica gel (pentane/Et_2O = 90:10) gave the desired product **10** as a colorless oil (72 mg, 0.26 mmol, 53% yield). ^{1}H NMR (300 MHz, $CDCl_3$) δ (ppm) 7.97-7.90 (m, 2H), 7.54-7.45 (m, 1H), 7.41-7.32 (m, 2H), 4.46 (t, J = 6.18 Hz, 2H), 4.15 (q, J = 7.17, 2H), 2.53 (tt, J = 15.72, 6.18 Hz, 2H), 1.19 (t, J = 7.17 Hz, 3H). ^{13}C NMR (75 MHz, $CDCl_3$) δ (ppm) 166.1, 163.7, 133.2, 129.6, 128.4, 114.8 (t, J = 251.1 Hz), 63.1, 58.0 (t, J = 5.9 Hz), 34.08 (t, J = 23.9 Hz), 13.8. ^{19}F NMR (376 MHz, $CDCl_3$) δ (ppm) -105.2 (s). IR (neat, cm^{-1}): 2983, 1761, 1721, 1598, 1452, 1374, 1315, 1270, 1194, 1094, 1025, 841, 709, 687. HRMS (ESI) Calcd. for $C_{13}H_{15}O_4F_2$: 273.0933 [M+H]$^+$, Found : 273.0934.

Benzyl 4-acetoxypentanoate (11)

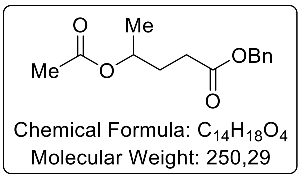

Following general procedure A, the reaction was carried out with benzyl 2-iodoacetate **2b (**139 mg, 0.50 mmol), isopropenyl acetate **1c** (250 mg, 0.27 mL, 2.50 mmol), 4-*tert*-butylcatechol (498 mg, 3.00 mmol), Et_3B (0.6 mL, 0.60 mmol). Flash column chromatography on silica gel (pentane/Et_2O = 85:15) gave the desired product **11** as a colorless oil (104 mg, 0.42 mmol, 83%). ^{1}H NMR (300 MHz, $CDCl_3$) δ (ppm) 7.33-7.23 (m, 5H), 5.04 (s, 2H), 4.85 (sex, 1H), 2.43-2.24 (m, 2H), 1.93 (d, J = 1.0 Hz, 3H), 1.89-1.79 (m, 2H), 1.15 (dd, J = 6.3, 1.0 Hz, 3H). ^{13}C NMR (75 MHz, $CDCl_3$) δ (ppm) 172.9, 170.7, 136.0, 128.7, 128.4, 70.1, 66.5, 30.9, 30.5, 21.3, 19.9. IR (neat, cm^{-1}): 2978, 2355, 1729, 1455, 1372, 1237, 1164, 1131, 1073, 961, 746, 697, 607. HRMS (ESI) Calcd. for $C_{14}H_{19}O_4$: 251.1278 [M+H]$^+$, Found : 251.1275.

Phenyl 4-acetoxypentanoate (12)

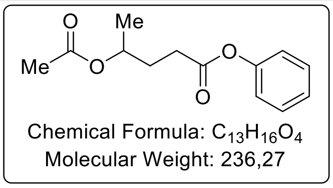

Following general procedure A, the reaction was carried out with phenyl 2-iodoacetate **2c (**131 mg, 0.50 mmol), isopropenyl acetate **1c** (250 mg, 0.27 mL, 2.50 mmol), 4-*tert*-butylcatechol (500 mg, 3.00 mmol), Et_3B (0.6 mL, 0.60 mmol). Flash column chromatography on silica gel (pentane/Et_2O = 85:15) gave the desired product **12** as a colorless oil (110 mg, 0.47 mmol, 93%). ^{1}H NMR (300 MHz, $CDCl_3$) δ (ppm) 7.35-7.25 (m, 2H), 7.21-7.10 (m, 1H), 7.04-6.95 (m, 2H), 4.93 (sex, 1H), 2.57-2.48 (m, 2H), 1.96 (d, J = 1.3 Hz, 3H), 1.95-1.87 (m, 2H), 1.20 (dd, J = 6.3, 1.3 Hz, 3H). ^{13}C NMR (75 MHz, $CDCl_3$) δ (ppm) 171.5, 170.6, 150.7, 129.4, 125.8, 121.5, 69.8, 30.9, 30.5, 21.2, 19.9. IR (neat, cm^{-1}): 2978, 2355, 1755, 1731, 1592, 1493, 1456, 1372, 1238, 1193, 1129, 1070, 1023, 934, 813, 752, 689, 627. HRMS (ESI) Calcd. for $C_{13}H_{17}O_4$: 237.1121 [M+H]$^+$, Found : 237.1124.

Benzyl 4-acetoxy-3-methylhexanoate (13)

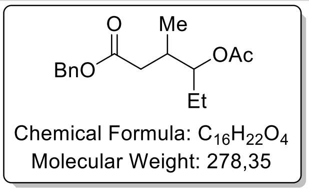

Following general procedure A, the reaction was carried out with olefin **1d**[5] (192 mg, 1.50 mmol), benzyl iodoacetate **2b** (138 mg, 0.5 mmol), *tert*-butylcatechol (249 mg, 1.50 mmol), Et_3B (0.6 mL, 0.60 mmol) in DCM (10 mL) and it took 1 h for the reaction to go to completion. Flash chromatography on silica gel (gradient of pentane/diethyl ether = 9:1 to 7:1) gave the desired product **13** as a colorless oil (63 mg, 0.23 mmol, 45% yield, dr = 1:1). ^{1}H NMR (300 MHz, $CDCl_3$) δ (ppm) 7.40 – 7.28 (m, 10H), 5.124 (s, 2H), 5.118 (s, 2H), 4.81 (ddd, J = 7.8, 5.7, 3.7 Hz, 1H), 4.74 (ddd, J = 7.6, 5.9, 4.8 Hz, 1H), 2.51 – 2.41 (m, 2H), 2.36 – 2.22 (m, 2H), 2.22 – 2.12 (m, 2H), 2.03 (s, 3H), 2.02 (s, 3H), 1.66 – 1.44 (m, 4H), 0.95 (d, J = 6.6, 3H), 0.94 (d,

---

[5] Onishi, Y.; Nishimoto, Y.; Yasuda, M.; Baba, A. *Org. Lett.* **2011**, *13*, 2762.

J = 6.6, 3H), 0.88 (t, J = 7.4, 3H), 0.87 (t, J = 7.4, 3H). ^{13}C NMR (75 MHz, $CDCl_3$) δ (ppm) 172.8, 172.6, 170.98, 170.97, 136.1, 128.7, 128.41, 128.40, 128.38, 78.4, 77.8, 66.44, 66.41, 38.2, 37.4, 33.4, 33.2, 24.5, 24.1, 21.2, 21.1, 16.7, 14.4, 10.2, 9.7. HRMS (ESI) Calcd for $C_{16}H_{23}O_4$: 279.1591 $[M+H]^+$, Found: 279.1594.

Benzyl 2-(2-acetoxycyclopentyl)acetate (14)

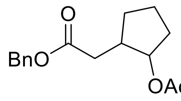

Chemical Formula: $C_{16}H_{20}O_4$
Molecular Weight: 276,33

Following general procedure A, the reaction was carried out with cyclopent-1-en-1-yl acetate **1e** (189 mg, 1.50 mmol), benzyl iodoacetate (135 mg, 0.50 mmol), *tert*-butylcatechol **2b** (249 mg, 1.50 mmol), Et_3B (0.6 mL, 0.60 mmol) in DCM (10 mL) and it took 2 h for the reaction to go to completion. Flash chromatography on silica gel (gradient of pentane/diethyl ether = 10:1 to 6:1) gave the desired product **14** as a colorless oil (100 mg, 0.36 mmol, 72% yield, dr = 1.3:1). ^{1}H NMR (300 MHz, $CDCl_3$) δ (ppm) 7.44 – 7.30 (m, 10H), 5.22 (td, J = 5.1, 1.9 Hz, 1H, minor dia), 5.134 (s, 2H, major dia), 5.129 (s, 2H, minor dia), 4.82 (dt, J = 6.7, 4.7 Hz, 1H, major dia), 2.68 – 2.50 (m, 2H), 2.48 – 2.27 (m, 4H), 2.02 (s, 3H, major dia), 2.00 (s, 3H, minor dia), 2.06 – 1.94 (m, 3H), 1.83 – 1.56 (m, 7H), 1.55 – 1.38 (m, 1H), 1.36 – 1.20 (m, 1H). ^{13}C NMR (75 MHz, $CDCl_3$) δ (ppm) 172.7 (minor dia), 172.4 (major dia), 171.1 (major dia), 170.7 (minor dia), 136.09 (minor dia), 136.07 (major dia), 128.7 (major and minor dia), 128.42 (major and minor dia), 128.37 (major and minor dia), 80.4 (major dia), 77.5 (minor dia), 66.4 (minor dia), 66.39 (major dia), 42.1 (major dia), 40.3 (minor dia), 38.1 (major dia), 34.5 (minor dia), 32.5 (minor dia), 31.7 (major dia), 30.2 (major dia), 29.7 (minor dia), 22.5 (major dia), 22.2 (minor dia), 21.3 (major dia), 21.2 (minor dia). HRMS (ESI) Calcd for $C_{16}H_{21}N_4$: 277.1434 $[M+H]^+$, Found: 277.1438.

Benzyl 2-(2-acetoxycyclohexyl)acetate (15)

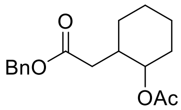

Chemical Formula: $C_{17}H_{22}O_4$
Molecular Weight: 290,36

Following general procedure A, the reaction was carried out with cyclohex-1-en-1-yl acetate **1f** (210 mg, 1.50 mmol), benzyl iodoacetate **2b** (135 mg, 0.50 mmol), *tert*-butylcatechol (249 mg, 1.50 mmol), Et_3B (0.6 mL, 0.60 mmol) in DCM (10 mL) and it took 4 h for the reaction to go to completion. Flash chromatography on silica gel (gradient of pentane/diethyl ether = 15:1 to 6:1) gave the desired product **15** as a colorless oil (67 mg, 0.23 mmol, 46% yield, dr = 1.5:1). ^{1}H NMR (300 MHz, $CDCl_3$) δ (ppm) 7.40 – 7.29 (m, 10H), 5.16 – 5.05 (m, 4H), 5.00 (p, J = 2.2 Hz, 1H of major dia), 4.51 – 4.43 (m, 1H of minor dia), 2.50 (dd, J = 15.2, 6.3 Hz, 1H of minor dia), 2.40 (dd, J = 15.2, 6.3 Hz, 1H of major dia), 2.27 (d, J = 7.8 Hz, 1H of minor dia), 2.24 – 2.06 (m, 3H), 2.06 – 2.00 (m, 1H), 2.02 (s, 3H of major dia), 1.97 (s, 3H of minor dia), 1.93 – 1.79 (m, 2H), 1.79 – 1.11 (m, 1H of minor dia), 1.70 – 1.61 (m, 2H), 1.58 –

1.41 (m, 6H), 1.40 – 1.23 (m, 3H), 1.23 – 1.01 (m, 1H of minor dia). ^{13}C NMR (75 MHz, $CDCl_3$) δ (ppm) 172.7 (minor dia), 172.6 (major dia), 170.8 (minor dia), 170.7 (major dia), 136.12 (minor dia), 136.08 (major dia), 128.7, 128.4, 128.37, 128.35, 76.6 (minor dia), 72.2 (major dia), 66.4 (major dia), 66.3 (minor dia), 39.4, 38.2, 37.1, 36.8, 31.9, 31.3, 29.7, 27.4, 25.2, 24.6, 21.3, 20.9. HRMS (ESI) Calcd for $C_{17}H_{23}O_4$: 291.1591 $[M+H]^+$, Found: 291.1583.

Benzyl 4-((diethoxyphosphoryl)oxy)butanoate (16)

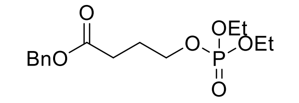

Chemical Formula: $C_{15}H_{23}O_6P$
Molecular Weight: 330,32

Following general procedure A, the reaction was carried out with olefin **1g**[6] (270 mg, 1.50 mmol), benzyl iodoacetate **2b** (138 mg, 0.5 mmol), *tert*-butylcatechol (249 mg, 1.50 mmol), Et_3B (0.5 mL, 0.60 mmol) in DCM (10 mL) and it took 3 h for the reaction to go to completion. Flash chromatography on silica gel (gradient of pentane/ethyl acetate = 1:1 to 1:3) gave the desired product **16** as a colorless oil (111 mg, 0.34 mmol, 67% yield). ^{1}H NMR (300 MHz, $CDCl_3$) δ (ppm) 7.34 (d, J = 3.4 Hz, 5H), 5.12 (s, 2H), 4.14 – 4.04 (m, 6H), 2.50 (t, J = 7.4 Hz, 2H), 2.02 (dddd, J = 13.6, 7.3, 6.1, 1.1 Hz, 2H), 1.32 (td, J = 7.1, 1.0 Hz, 6H). ^{13}C NMR (75 MHz, $CDCl_3$) δ (ppm) 172.7, 136.0, 128.7, 128.4, 128.3, 66.50, 66.48 (d, J = 5.9 Hz), 63.9 (d, J = 5.9 Hz), 30.3, 25.7 (d, J = 7.2 Hz), 16.3 (d, J = 6.7 Hz). IR (neat, cm^{-1}): 2981, 1733, 1260, 1164, 1019, 971, 740, 698. HRMS (ESI) Calcd for $C_{15}H_{24}O_6P$: 331.1305 $[M+H]^+$, Found: 331.1310.

Ethyl 4-(phenylthio) butanoate (18)

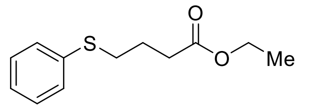

Chemical Formula: $C_{12}H_{16}O_2S$
Molecular Weight: 224,32

Following general procedure A, the reaction was carried out with ethyl 2-iodoacetate **2a (**107 mg, 0.50 mmol), phenyl vinyl sulfide **17a** (204 mg, 1.50 mmol), 4-*tert*-butylcatechol (250 mg, 1.50 mmol), Et_3B (0.6 mL, 0.6 mmol). Flash chromatography on silica gel (pentane/Et_2O = 96:4) gave the desired product **18** as a colorless oil (106 mg, 0.47 mmol, 95%). Physical and spectral data were in accordance with the literature.[7] ^{1}H NMR (300 MHz, $CDCl_3$) δ (ppm) 7.36-7.26 (m, 4H), 7.15-7.07 (m, 1H), 4.13 (q, J = 7.1 Hz, 2H), 2.97 (t, J = 7.1 Hz, 2H), 2.46 (t, J = 7.3 Hz, 2H), 1.96 (quin, 2H), 1.25 (t, J = 7.1 Hz, 3H). ^{13}C NMR (75 MHz, $CDCl_3$) δ (ppm) 172.9, 136.1, 129.4, 128.9, 126.0, 60.4, 33.0, 32.9, 24.4, 14.2. IR (Neat, cm^{-1}): 2963, 1728, 1583, 1480, 1438, 1373, 1278, 1202, 1135, 1024, 736, 690. HRMS (ESI) Calcd. for $C_{12}H_{16}O_2NaS$: 247.0763 $[M+Na]^+$, Found : 247.0767.

Benzyl 4-(phenylthio)butanoate (19)

---

[6] Kumpulainen, H.; Järvinen, T.; Saari, R.; Lehtonen, M.; Vepsäläinen, J. *J. Org. Chem.* **2005**, *70*, 9056.

[7] Giese, B.; Horler, H.; Leising, M. *Chem. Ber.* **1986**, *119*, 444.

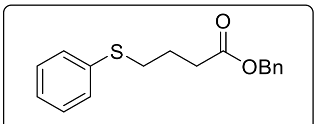

Gram scale synthesis:

Following general procedure A, the reaction was carried out with benzyl 2-iodoacetate **2b (**1.00 g, 3.62 mmol), phenyl vinyl sulfide **17a** (1.48 g 10.86 mmol), 4-*tert*-butylcatechol (1.80 g, 10.86 mmol), Et_3B (4.3 mL, 4.34 mmol). Flash chromatography on silica gel (pentane/Et_2O = 97:3) gave the desired product **19** as a colorless oil (1.02 g, 3.56 mmol, 98%). ^{1}H NMR (300 MHz, $CDCl_3$) δ (ppm) 7.32-7.21 (m, 9H), 7.17-7.12 (m, 1H), 5.08 (s, 2H), 2.93 (t, J = 7.05 Hz, 2H), 2.49 (t, J = 7.23 Hz, 2H), 1.95 (quin, 2H). ^{13}C NMR (75 MHz, $CDCl_3$) δ (ppm) 172.8, 136.1, 135.9, 129.4, 128.9, 128.6, 128.3, 128.2, 126.1, 66.4, 33.0, 32.9, 24.4. IR (neat, cm^{-1}): 2945, 2360, 1731, 1583, 1480, 1438, 1382, 1164, 1136, 1024, 1001, 735, 690. HRMS (ESI) Calcd. for $C_{17}H_{19}O_2S$: 287.1100 [M+H]$^+$, Found : 287.1103.

Phenyl 4-(phenylthio) butanoate (20)

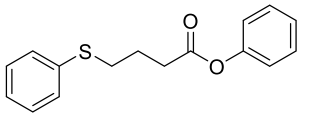

Following general procedure A, the reaction was carried out with phenyl 2-iodoacetate **2c (**131 mg, 0.50 mmol), phenyl vinyl sulfide **17a** (204 mg, 1.50 mmol), 4-*tert*-butylcatechol (249 mg, 1.50 mmol), Et_3B (0.6 mL, 0.60 mmol). Flash chromatography on silica gel (pentane/Et_2O = 97:3) gave the desired product **20** as a colorless oil (120 mg, 0.44 mmol, 88%). ^{1}H NMR (300 MHz, $CDCl_3$) δ (ppm) 7.37-7.31 (m, 4H), 7.29-7.22 (m, 2H), 7.22-7.13 (m, 2H), 7.06-7.02 (m, 2H), 3.02 (t, J = 7.0 Hz, 2H), 2.70 (t, J = 7.2 Hz, 2H), 2.04 (quin, 2H). ^{13}C NMR (75 MHz, $CDCl_3$) δ (ppm) 171.6, 150.7, 136.0, 129.6, 129.5, 129.1, 126.3, 125.9, 121.6, 33.1, 33.0, 24.4. IR (neat, cm^{-1}): 2957, 1755, 1591, 1491, 1480, 1438, 1360, 1191, 1161, 1121, 1069, 1024, 923, 810, 736, 687. HRMS (ESI) Calcd. For $C_{16}H_{16}O_2NaS$: 295.0763 [M+Na]$^+$, found : 295.0757.

Ethyl 2-methyl-4-(phenylthio)butanoate (21)

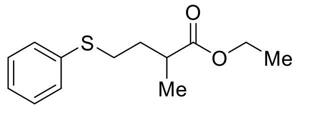

Following general procedure A, the reaction was carried out with ethyl 2-iodopropanoate **2d** (114mg, 0.50 mmol), phenyl vinyl sulfide **17a** (204 mg, 1.50 mmol), 4-*tert*-butylcatechol (249 mg, 1.50 mmol), Et_3B (0.6 mL, 0.60 mmol). Flash chromatography on silica gel (pentane/Et_2O = 96:4) gave the desired product **21** as a colorless oil (61 mg, 0.26 mmol, 51%). Physical and spectral data were in accordance with the literature.[8] ^{1}H NMR (300 MHz, $CDCl_3$) δ (ppm) 7.35-7.26 (m, 4H), 7.21-7.15 (m, 1H), 4.13 (q, J = 7.08 Hz, 2H), 3.00-2.85 (m,

---

[8] Curran, D. P.; Chen, M. H.; Spletzer, E.; Seong, C. M.; Chang, C. T. *J. Am. Chem. Soc.* **1989**, *111*, 8872.

2H), 2.68-2.57 (sex, 1H), 2.09-1.96 (m, 1H), 1.78-1.67 (m, 1H), 1.25 (t, *J* = 7.05, 3H), 1.17 (d, *J* = 7.02 Hz, 3H). ^{13}C NMR (75 MHz, $CDCl_3$) δ (ppm) 175.9, 136.2, 129.2, 128.9, 125.9, 60.4, 38.6, 33.0, 31.3, 17.0, 14.2. IR (neat, cm^{-1}): 2973, 2936, 1726, 1584, 1438, 1376, 1261, 1191, 1153, 1091, 1024, 858, 736, 690. HRMS (ESI) Calcd. for $C_{13}H_{18}O_2NaS$: 261.0920 $[M+Na]^+$, Found : 261.0921.

3-(2-(Phenylthio)ethyl)dihydrofuran-2(3*H*)-one (22)

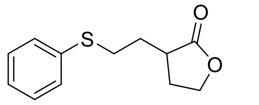

Chemical Formula: $C_{12}H_{14}O_2S$
Molecular Weight: 222,30

Following general procedure A, the reaction was carried out with 3-iododihydrofuran-2(3H)-one **2e** (106 mg, 0.50 mmol), phenyl vinyl sulfide **17a** (204 mg, 1.50 mmol), 4-*tert*-butylcatechol (250 mg, 1.50 mmol), Et_3B (0.6 mL, 0.60 mmol). Flash chromatography on silica gel (pentane/Et_2O = 80:20) gave the desired product **22** as a colorless oil (89 mg, 0.40 mmol, 80%). ^{1}H NMR (300 MHz, $CDCl_3$) δ (ppm) 7.31 – 7.25 (m, 2H), 7.25 – 7.17 (m, 2H), 7.16 – 7.08 (m, 1H), 4.25 (td, *J* = 8.8, 2.5 Hz, 1H), 4.09 (td, *J* = 9.5, 6.6 Hz, 1H), 3.09 – 2.89 (m, 2H), 2.76 – 2.61 (m, 1H), 2.39 – 2.26 (m, 1H), 2.18 – 2.02 (m, 1H), 1.94 – 1.76 (m, 1H), 1.68 (m, 1H). ^{13}C NMR (75 MHz, $CDCl_3$) δ (ppm) 178.9, 135.5, 129.6, 129.1, 126.4, 66.5, 38.0, 31.5, 29.9, 28.8. IR (neat, cm^{-1}): 2911, 1759, 1582, 1480, 1437, 1374, 1278, 1179, 1143, 1088, 1022, 965, 737, 690. HRMS (ESI) Calcd. for $C_{12}H_{15}O_2S$: 223.0787 $[M+H]^+$, Found : 223.0791.

Ethyl 2,2-difluoro-4-(phenylthio)butanoate (23)

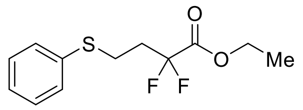

Chemical Formula: $C_{12}H_{14}F_2O_2S$
Molecular Weight: 260,30

Following general procedure A, the reaction was carried out with ethyl 2,2-difluoro-2-iodoacetate **2f (**125 mg, 0.50 mmol), phenyl vinyl sulfide **17a** (204 mg, 1.50 mmol), 4-*tert*-butylcatechol (250 mg, 1.50 mmol), Et_3B (0.6 mL, 0.60 mmol). Flash chromatography on silica gel (pentane/Et_2O = 80:20) gave the desired product **23** as a colorless oil (102 mg, 0.39 mmol, 79%). ^{1}H NMR (300 MHz, $CDCl_3$) δ (ppm) 7.32-7.27 (m, 2H), 7.27-7.20 (m, 2H), 7.20-7.13 (m, 1H), 4.24 (q, *J* = 7.1 Hz, 2H), 2.97 (m, 2H), 2.43-2.21 (m, 2H), 1.26 (t, *J* =7.1 Hz, 3H). ^{13}C NMR (75 MHz, $CDCl_3$) δ (ppm) 163.7, 134.8, 129.8, 129.2, 126.7, 115.1 (t, *J* = 249.7 Hz), 63.1, 34.8 (t, *J* = 23.2 Hz), 25.7 (t, *J* = 5.25 Hz), 13.9. ^{19}F NMR (376 MHz, $CDCl_3$) δ (ppm) -106.2 (s). IR (neat, cm^{-1}): 2983, 1759, 1584, 1481, 1439, 1374, 1305, 1184, 1083, 1024, 943, 850, 737, 690, 636. HRMS (ESI) Calcd. for $C_{12}H_{15}O_2F_2S$: 261.0755 $[M+H]^+$, Found : 261.0757.

Diethyl 2-(2-(phenylthio)ethyl) malonate (24)

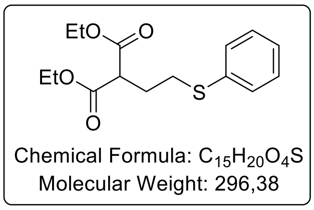

Following general procedure A, the reaction was carried out with diethyl 2-bromomalonate **2g (**146 mg, 0.59 mmol), phenyl vinyl sulfide **7a** (142 mg, 0.14 mL, 2.00 mmol), 4-*tert*-butylcatechol (260 mg, 3.00 mmol), Et_3B (1.3 mL, 1.30 mmol). Flash chromatography on silica gel (2-12% ether in pentane) gave the desired product **24** as a colorless oil (151 mg, 0.52 mmol, 88%). Spectral and physical data are in accordance with the literature.[9] ^{1}H NMR (300 MHz, $CDCl_3$) δ (ppm) 7.32 – 7.25 (m, 2H), 7.25 – 7.17 (m, 2H), 7.16 – 7.08 (m, 1H), 4.20 – 4.04 (m, 4H), 3.52 (t, J = 7.2 Hz, 1H), 2.94 – 2.85 (m, 2H), 2.18-2.08 (m, 2H), 1.17 (t, J = 7.1 Hz, 6H). ^{13}C NMR (75 MHz, $CDCl_3$) δ (ppm) 169.1, 135.7, 129.8, 129.1, 126.4, 61.7, 50.7, 31.5, 28.4, 14.2.

Benzyl 4-(phenylthio)pentanoate (25)

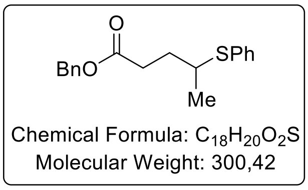

Following general procedure A, the reaction was carried out with olefin **7b**[10] (225 mg, 1.50 mmol), benzyl iodoacetate **2b** (138 mg, 0.5 mmol), *tert*-butylcatechol (249 mg, 1.50 mmol), Et_3B (0.6 mL, 0.60 mmol) in DCM (10 mL) and it took 2 h for the reaction to go to completion (dichloromethane/pentane = 1:5 for TLC plate). Flash chromatography on silica gel (gradient of dichloromethane/pentane = 1:4 to 1:2) gave the desired product **25** as a colorless oil (120 mg, 0.40 mmol, 80% yield). ^{1}H NMR (300 MHz, $CDCl_3$) δ (ppm) 7.42 – 7.31 (m, 7H), 7.31 – 7.22 (m, 3H), 5.12 (s, 2H), 3.23 (h, J = 6.7 Hz, 1H), 2.64 – 2.53 (m, 2H), 1.96 – 1.84 (m, 2H), 1.29 (d, J = 6.8 Hz, 3H). ^{13}C NMR (75 MHz, $CDCl_3$) δ (ppm) 173.2, 136.1, 134.7, 132.5, 129.0, 128.7, 128.4, 128.3, 127.1, 66.4, 43.0, 31.8, 31.6, 21.3. IR (neat, cm^{-1}): 2958, 2919, 1731, 1583, 1454, 1438, 1378, 1154, 1114, 1089, 1023, 738, 692. HRMS (ESI) Calcd for $C_{18}H_{21}O_2S$: 301.1257 $[M+H]^+$, Found: 301.1251.

Benzyl 4-butoxybutanoate (27)

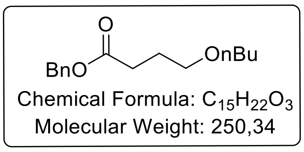

Following general procedure B, the reaction was carried out with benzyl 2-(ethoxycarbonothioylthio) acetate **2'b** (135 mg, 0.50 mmol, 1.0 equiv), butyl vinyl ether **26a (**150 mg, 1.50 mmol, 3.0 equiv), 4-*tert*-butylcatechol (249 mg, 1.50 mmol, 3.0 equiv), Et_3B (1.5 mL, 1.50 mmol, 3.0 equiv) in CH_2Cl_2 (5.0 mL). Flash chromatography on silica gel (pentane/Et_2O 92:8) gave the desired product **27** as a colorless oil (100 mg, 0.40 mmol, 80%). ^{1}H NMR (300 MHz, $CDCl_3$) δ (ppm) 7.39-7.31 (m, 5H), 5.12 (s, 2H), 3.43(t, J = 6.21 Hz, 2H), 3.38 (t, J = 6.54 Hz, 2H), 2.46 (t, J = 7.35 Hz, 2H), 1.97-1.85 (m, 2H), 1.58-1.45 (m, 2H), 1.42-1.25 (m, 2H), 0.93 (t, J = 7.32, 3H). ^{13}C

---

[9] Quiclet-Sire, B.; Revol, G.; Zard, S. Z. *Tetrahedron* **2010**, *66*, 6656.

[10] Harada, T.; Karasawa, A.; Oku, A. *J. Org. Chem.* **1986**, *51*, 842.

NMR (75 MHz, $CDCl_3$) δ (ppm) 173.4, 136.1, 128.5, 128.2, 70.7, 69.5, 66.1, 31.8, 31.1, 25.1, 19.3, 13.9. IR (neat, cm^{-1}): 2956, 2931, 2863, 2358, 2338, 2023, 1735, 1455, 1377, 1244, 1163, 1110, 1026, 973, 734, 696. Anal. Calcd for $C_{15}H_{22}O_3$: C, 71.97; H, 8.86. Found: C, 71.87; H, 8.96.

Diethyl 2-(2-butoxyethyl)malonate (28)

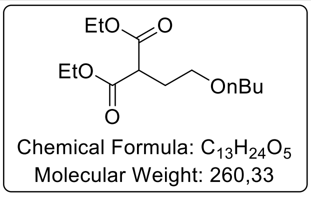

Following general procedure B, the reaction was carried out with diethyl 2-(ethoxycarbonothioylthio)malonate **2'g (**280 mg, 1.00 mmol), butyl vinyl ether **26a** (204 mg, 0.26 mL, 2.00 mmol), 4-*tert*-butylcatechol (498 mg, 3.00 mmol), Et_3B (2.5mL, 2.50 mmol). Flash chromatography on silica gel (pentane/Et_2O = 92:8) gave the desired product **28** as a pale yellow oil (225 mg, 0.86 mmol, 85%). Spectral data are in accordance with the literature report.[11] ^1H NMR (300 MHz, $CDCl_3$) δ (ppm) 4.20 (q, J = 7.2 Hz 2H), 4.20 (q, J = 7.2 Hz, 2H), 3.55 (t, J = 7.3 Hz, 1H), 3.45 (t, J = 6.0 Hz, 2H), 3.38 (t, J = 6.5 Hz, 2H), 2.17 (dd, J = 13.4, 6.1 Hz, 2H), 1.59 – 1.45 (m, 2H), 1.43 – 1.31 (m, 2H), 1.27 (t, J = 7.1 Hz, 6H), 0.91 (t, J = 7.3 Hz, 3H). ^{13}C NMR (75 Hz, $CDCl_3$) δ (ppm) 169.5, 70.8, 67.8, 61.3, 49.1, 31.7, 28.9, 19.3, 14.1, 13.9. IR (neat, cm^{-1}): 2960, 2936, 2908, 2868, 1748, 1730, 1465, 1369, 1332, 1298, 1256, 1231, 1174, 1151, 1111, 1026, 861. HRMS (ESI) Calcd. For $C_{13}H_{24}O_5Na$: 283.1516 $[M+Na]^+$, Found:. 283.1515.

Diethyl 2-(2-butoxyethyl)-2-methylmalonate (29)

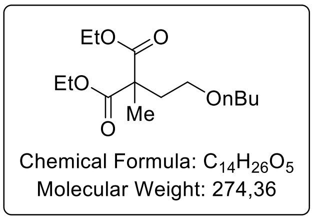

Following general procedure B, the reaction was carried out with diethyl 2-(ethoxycarbonothioylthio)-2-methylmalonate **2'h** (294 mg, 1.00 mmol), butyl vinyl ether **26a** (204 mg, 0.26 mL, 2.00 mmol), 4-*tert*-butylcatechol (498 mg, 3.00 mmol), Et_3B (2.5 mL, 2.50 mmol). Flash chromatography on silica gel (2-10% ether in pentane) gave the desired product **29** as a colorless oil (206 mg, 75%). ^1H NMR (300 MHz, $CDCl_3$) δ (ppm) 4.25 – 4.10 (m, 4H), 3.46 (t, J = 6.6 Hz, 2H), 3.35 (t, J = 6.6 Hz, 2H), 2.18 (t, J = 6.6 Hz, 2H), 1.57 – 1.45 (m, 2H), 1.44 (s, 3H), 1.40 – 1.29 (m, 2H), 1.25 (t, J = 7.1 Hz, 6H), 0.90 (t, J = 7.3 Hz, 3H). ^{13}C NMR (75 MHz, $CDCl_3$) δ (ppm) 172.2, 70.9, 66.6, 61.2, 52.1, 35.2, 31.8, 19.9, 19.3, 14.0, 13.9. IR (neat, cm^{-1}): 2980, 2960, 2936, 2869, 1729, 1463, 1377, 1300, 1238, 1202, 1107, 1024, 862. HRMS (ESI) Calcd. for $C_{14}H_{26}O_5Na$: 297.1672 $[M+Na]^+$, Found: 297.1669.

---

[11] Braun, M.-G.; Zard, S. Z. *Org. Lett.* **2011**, *13*, 776.

Cyclohex-2-enyl 4-butoxybutanoate (30)

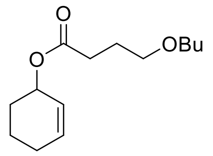

Chemical Formula: $C_{14}H_{24}O_3$
Molecular Weight: 240,34

Following general procedure B, the reaction was carried out with cyclohex-2-enyl 2-(ethoxycarbonothioylthio) acetate **6i (**260 mg, 1.0 mmol), butyl vinyl ether **26a** (300 mg, 3.0 mmol) in dichloromethane (10.0 mL), 4-*tert*-butylcatechol (500 mg, 3.0 mmol), Et_3B (1M in hexane) (3.0 mL, 3.0 mmol). Flash chromatography on silica gel (2-9% ether in pentane) gave the desired product **30** as a colorless oil (160 mg, 0.67 mmol, 67%). ^{1}H NMR (300 MHz, $CDCl_3$) δ (ppm) 5.99 – 5.89 (m, 1H), 5.70 (ddt, J = 10.0, 3.9, 2.1 Hz, 1H), 5.31 – 5.22 (m, 1H), 3.43 (t, J = 5.1 Hz, 2H), 3.39 (t, J = 5.4 Hz, 2H), 2.39 (t, J = 7.4 Hz, 2H), 2.18 – 1.97 (m, 2H), 1.96 – 1.81 (m, 3H), 1.80 – 1.60 (m, 3H), 1.57 – 1.48 (m, 2H), 1.45 – 1.31 (m, 2H), 0.91 (t, J = 7.3 Hz, 3H). ^{13}C NMR (75 MHz, $CDCl_3$) δ (ppm) 173.2, 132.6, 125.8, 70.7, 69.6, 67.9, 31.8, 31.4, 28.3, 25.2, 24.9, 19.3, 18.9, 13.9. IR (neat, cm^{-1}): 2933, 2862, 1726, 1453, 1367, 1244, 1159, 1113, 1010, 914, 728. HRMS (ESI) Calcd. for $C_{14}H_{24}O_3Na$: 263.1618 $[M+Na]^+$, Found: 263.1621.

Ethyl 4-(cyclohexyloxy)-2-methylbutanoate (31)

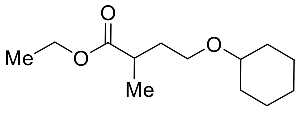

Chemical Formula: $C_{13}H_{24}O_3$
Molecular Weight: 228,33

Following general procedure B, the reaction was carried out with ethyl 2-(ethoxycarbonothioylthio)propanoate **2'd (**222 mg, 1.00 mmol, 1.0 equiv), cyclohexyl vinyl ether **26b** (315 mg, 2.50 mmol, 2.5 equiv), 4-*tert*-butylcatechol (500 mg, 3.00 mmol, 3.0 equiv), Et_3B (3.0 mL, 3.00 mmol, 1 M in hexane, 3.0 equiv) in dichloromethane (10.0 mL). Flash chromatography on silica gel (2-8% ether in pentane) gave the desired product **31** as a colorless oil (159 mg, 70%). ^{1}H NMR (300 MHz, $CDCl_3$) δ (ppm) 4.13 (q, J = 7.1 Hz, 2H), 3.52 – 3.39 (m, 2H), 3.25 – 3.13 (m, 1H), 2.68 – 2.53 (m, 1H), 2.03 – 1.80 (m, 3H), 1.79 – 1.58 (m, 3H), 1.57 – 1.42 (m, 1H), 1.32 – 1.13 (m, 11H). ^{13}C NMR (75 MHz, $CDCl_3$) δ (ppm) 176.7, 65.3, 60.1, 36.6, 34.0, 32.2, 32.1, 25.8, 24.1, 17.2, 14.2. HRMS (ESI) Calcd. for $C_{13}H_{24}O_3Na$: 251.1618 $[M+Na]^+$, Found: 251.1615.

Benzyl 4-methoxypentanoate (32)

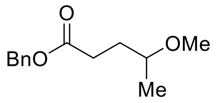

Chemical Formula: $C_{13}H_{18}O_3$
Molecular Weight: 222,28

Following general procedure B, the reaction was carried out with 2-methoxypropene **26c** (108 mg, 1.50 mmol, 3.0 equiv), xanthate **2'b** (135 mg, 0.50 mmol, 1.0 equiv), 4-*tert*-butylcatechol (249 mg, 1.50 mmol, 3.0 equiv), Et_3B (1.5 mL, 1.50 mmol, 3.0 equiv) in DCM (5.0 mL) and it took 3 h for the reaction to go to completion. Flash chromatography on silica gel (gradient of pentane/diethyl ether = 15:1 to 8:1) gave the desired product **7cb** as a colorless oil (71 mg, 0.32 mmol, 64% yield). ^{1}H NMR (300 MHz, $CDCl_3$) δ (ppm) 7.40 – 7.28 (m, 5H), 5.12 (s,

2H), 3.38 – 3.26 (m, 4H), 2.45 (td, J = 7.3, 1.2 Hz, 2H), 1.81 (td, J = 7.8, 6.2 Hz, 2H), 1.13 (d, J = 6.1 Hz, 3H). ^{13}C NMR (75 MHz, $CDCl_3$) δ (ppm) 173.7, 136.2, 128.7, 128.31, 128.30, 75.8, 66.3, 56.2, 31.5, 30.4, 19.0. IR (neat, cm^{-1}): 2970, 2930, 2822, 1733, 1456, 1374, 1341, 1262, 1160, 1132, 1085, 1028, 735, 696. HRMS (ESI) Calcd for $C_{13}H_{19}O_3$: 223.1329 $[M+H]^+$, Found: 223.1332.

Diethyl 2-(1-ethoxypropan-2-yl)malonate (33)

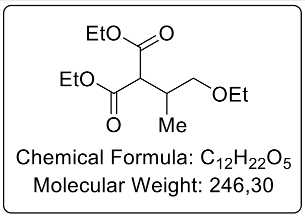

Following general procedure B, the reaction was carried out with diethyl 2-(ethoxycarbonothioylthio)malonate **2'g (**280 mg, 1.00 mmol, 1.0 equiv), (E/Z)-1-ethoxyprop-1-ene **26d** (223 mg, 0.29 mL, 2.50 mmol, 2.5 equiv), 4-*tert*-butylcatechol (498 mg, 3.00 mmol, 3.0 equiv), Et_3B (2.5mL, 2.50 mmol, 2.5 equiv). Flash chromatography on silica gel (2-10% ether in pentane) gave the desired product **33** as a pale yellow oil (225 mg, 0.91 mmol, 91%). ^{1}H NMR (300 MHz, $CDCl_3$) δ (ppm) 4.19 (q, J = 7.1 Hz, 2H), 4.19 (q, J = 7.1 Hz, 2H), 3.49 – 3.40 (m, 3H), 3.37 (d, J = 6.1 Hz, 2H), 2.61 – 2.45 (sept, 1H), 1.27 (t, J = 7.1 Hz, 3H), 1.27 (t, J = 7.1 Hz, 3H) 1.16 (t, J = 7.0 Hz, 3H), 1.02 (d, J = 6.9 Hz, 3H). ^{13}C NMR (75 Hz, $CDCl_3$) δ (ppm) 169.0, 168.8, 73.1, 66.3, 61.1 (2C), 54.4, 33.8, 15.1, 14.7, 14.1. IR (neat, cm^{-1}): 2977, 2936, 2871, 1751, 1728, 1462, 1369, 1303, 1263, 1244, 1221, 1174, 1151, 1106, 1029, 863. HRMS (ESI) Calcd. for $C_{12}H_{22}O_5Na$: 269.1359 $[M+Na]^+$, Found: 269.1366.

Diethyl 2-(1-ethoxypropan-2-yl)-2-methylmalonate (34)

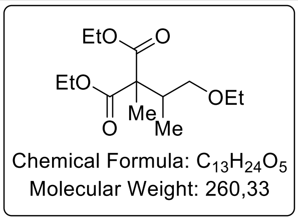

Following general procedure B, the reaction was carried out with diethyl 2-(ethoxycarbonothioylthio)-2-methylmalonate **2'h** (270 mg, 0.92 mmol, 1.0 equiv), (E/Z)-1-ethoxy-prop-1-ene **26d** (158 mg, 0.2 mL, 1.83 mmol, 2.0 equiv), 4-*tert*-butylcatechol (457 mg, 3.00 mmol, 3.0 equiv), Et_3B (2.5 mL, 2.50 mmol, 2.5 equiv). Flash chromatography on silica gel (2-14% ether in pentane) gave the desired product **34** as a colorless oil (152 mg, 0.58 mmol, 63%). ^{1}H NMR (300 MHz, $CDCl_3$) δ (ppm) 4.26 – 4.07 (m, 4H), 3.52 – 3.34 (m, 3H), 3.24 (dd, J = 9.6, 7.3 Hz, 1H), 2.70 – 2.56 (m, 1H), 1.36 (s, 3H), 1.25 (t, J = 7.1 Hz, 6H), 1.16 (t, J = 7.0 Hz, 3H), 0.97 (d, J = 6.9 Hz, 3H). ^{13}C NMR (75 MHz, $CDCl_3$) δ (ppm) 171.7, 171.5, 66.2, 61.1, 61.0, 56.0, 37.6, 16.4, 15.1, 14.0, 13.4. IR (neat, cm^{-1}): 2977, 2873, 1729, 1448, 1380, 1244, 1221, 1132, 1111, 1091, 1021, 861. HRMS (ESI) Calcd. for $C_{13}H_{24}O_5Na$: 283.1516 $[M+Na]^+$, Found:. 283.1515.

Benzyl 2-(tetrahydrofuran-3-yl)acetate (35)

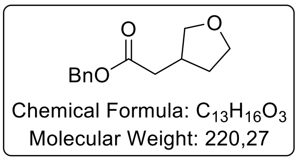

Under nitrogen, to a solution of 2,3-dihydrofuran **26e** (105 mg, 1.50 mmol, 3.0 equiv) and xanthate **2'b** (135 mg, 0.50 mmol, 1.0 equiv) in dry DCM (5.0 mL) was added 4-*tert*-butylcatechol (249 mg, 1.50 mmol, 3.0 equiv).The solution was the cooled down to 0 °C and Et_3B (1.5 mL, 1 M in n-hexane) was added slowly via syringe while the needle was immersed in the solution. The resulting reaction mixture was stirred open to air for 4 h at 0 °C with a $CaCl_2$ trap. The solution was filtered over a small pad of neutral alox (eluted with diethyl ether). The filtrate was then concentrated under reduced pressure and purification by flash chromatography on silica gel (gradient of diethyl ether/pentane = 1:3 to 3:7) gave the desired product **35** as a colorless oil (30 mg, 0.14 mmol, 27% yield). ^1H NMR (300 MHz, $CDCl_3$) δ (ppm) 7.44 – 7.31 (m, 5H), 5.15 (s, 2H), 3.96 (dd, J = 8.6, 7.0 Hz, 1H), 3.88 (td, J = 8.3, 5.2 Hz, 1H), 3.78 (dt, J = 8.6, 7.4 Hz, 1H), 3.44 (dd, J = 8.6, 6.4 Hz, 1H), 2.67 (dtd, J = 13.3, 6.7, 1.3 Hz, 1H), 2.50 (s, 1H), 2.48 (d, J = 1.2 Hz, 1H), 2.14 (dtd, J = 12.8, 7.7, 5.2 Hz, 1H), 1.72 – 1.52 (m, 1H). ^{13}C NMR (75 MHz, $CDCl_3$) δ (ppm) 172.4, 136.0, 128.7, 128.45, 128.37, 73.1, 67.8, 66.5, 38.1, 35.6, 32.2. IR (neat, cm^{-1}): 2940, 2860, 1730, 1268, 1212, 1157, 968, 904, 738, 696. HRMS (ESI) Calcd for $C_{14}H_{17}O_3$: 221.1172 $[M+H]^+$, Found: 221.1172.

Benzyl 2-(tetrahydro-2*H*-pyran-3-yl)acetate (36)

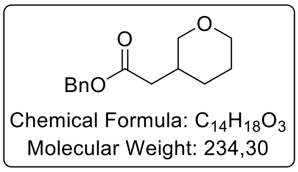

Under nitrogen, to a solution of 3,4-dihydropyran **26f** (126 mg, 1.50 mmol, 3.0 equiv) and xanthate **2'b** (135 mg, 0.50 mmol, 1.0 equiv) in dry DCM (5.0 mL) was added 4-tert-butylcatechol (249 mg, 1.50 mmol, 3.0 equiv).The solution was the cooled down to 0 °C and Et_3B (1.5 mL, 1 M in n-hexane) was added slowly via syringe while the needle was immersed in the solution. The resulting reaction mixture was stirred open to air for 4 h at 0 °C with a $CaCl_2$ trap. The solution was filtered over a small pad of neutral alox (eluted with diethyl ether). The filtrate was then concentrated under reduced pressure and purification by flash chromatography on silica gel (diethyl ether/pentane = 1:3) gave the desired product **36** as a colorless oil (69 mg, 0.29 mmol, 60% yield). ^1H NMR (300 MHz, $CDCl_3$) δ (ppm) 7.43 – 7.28 (m, 5H), 5.12 (s, 2H), 3.84 (tdd, J = 10.6, 3.9, 1.6 Hz, 2H), 3.45 – 3.33 (m, 1H), 3.14 (dd, J = 11.2, 9.1 Hz, 1H), 2.33 – 2.17 (m, 2H), 2.17 – 2.04 (m, 1H), 1.87 (dtt, J = 14.4, 3.7, 2.0 Hz, 1H), 1.62 (ddt, J = 11.2, 6.1, 4.0 Hz, 2H), 1.33 – 1.17 (m, 1H). ^{13}C NMR (75 MHz, $CDCl_3$) δ (ppm) 172.2, 136.0, 128.7, 128.40, 128.37, 72.6, 68.4, 66.4, 37.4, 33.1, 29.7, 25.4. IR (neat, cm^{-1}): 2934, 2845, 1731, 1455, 1267, 1182, 1165, 1092, 1032, 982, 935, 913, 857, 737, 696. HRMS (ESI) Calcd for $C_{14}H_{19}O_3$: 235.1329 $[M+H]^+$, Found: 235.1330.

Benzyl 5,5-dimethyl-4-((trimethylsilyl)oxy)hexanoate (38)

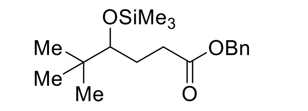

Chemical Formula: $C_{18}H_{30}O_3Si$
Molecular Weight: 322,52

Following general procedure B, the reaction was carried out with olefin **37a**[12] (260 mg, 1.50 mmol, 3.0 equiv), xanthate **2'b** (135 mg, 0.50 mmol, 1.0 equiv), 4-*tert*-butylcatechol (249 mg, 1.50 mmol, 3.0 equiv), Et_3B (1.5 mL, 1.50 mmol, 3.0 equiv) in DCM (5 mL) and it took 3 h for the reaction to go to completion. Flash chromatography on silica gel (pentane/diethyl ether = 25:1) gave the desired product **38** as a colorless oil (141 mg, 0.35 mmol, 71% yield). ^{1}H NMR (300 MHz, $CDCl_3$) δ (ppm) 7.41 – 7.28 (m, 5H), 5.12 (s, 2H), 3.28 (dd, J = 10.0, 2.3 Hz, 1H), 2.51 (ddd, J = 16.0, 9.2, 5.3 Hz, 1H), 2.32 (ddd, J = 16.0, 9.0, 7.1 Hz, 1H), 1.89 (dddd, J = 13.8, 9.3, 7.1, 2.3 Hz, 1H), 1.59 (dddd, J = 14.0, 10.0, 9.0, 5.3 Hz, 1H), 0.85 (s, 9H), 0.11 (s, 9H). ^{13}C NMR (75 MHz, $CDCl_3$) δ (ppm) 173.9, 136.2, 128.7, 128.4, 128.3, 80.4, 66.3, 35.6, 32.0, 27.6, 26.4, 1.0. IR (neat, cm^{-1}): 2956, 1736, 1250, 1152, 1084, 1029, 979, 882, 835, 748, 696. HRMS (ESI) Calcd for $C_{18}H_{31}O_3Si$: 323.2048 [M+H]$^+$, Found: 323.2046.

Benzyl 4-((3*S*,8*S*,9*S*,10*R*,13*S*,14*S*,17*S*)-3-acetoxy-10,13-dimethyl-2,3,4,7,8,9,10,11,12,13,14,15,16,17-tetradecahydro-1*H*-cyclopenta[*a*]phenanthren-17-yl)-4-((*tert*-butyldimethylsilyl)oxy)butanoate (39)

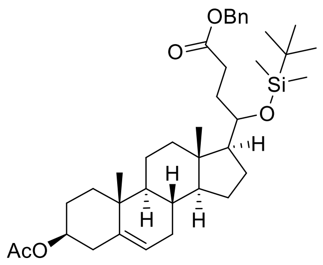

Chemical Formula: $C_{38}H_{58}O_5Si$
Molecular Weight: 622,96

Under nitrogen, to a solution of olefin **37b**[13] (473 mg, 1.00 mmol, 2.0 equiv) and xanthate **2'b** (135 mg, 0.50 mmol, 2.0 equiv) in dry DCM (5.0 mL) was added 4-*tert*-butylcatechol (249 mg, 1.50 mmol, 3.0 equiv), followed by slow addition of Et_3B (1.0 mL, 1 M in *n*-hexane, 2.0 equiv) while the needle was immersed in the solution. The resulting reaction mixture was stirred open to air for 1 h at rt with a $CaCl_2$ trap. The solution was filtered over a small pad of neutral alox (eluted with diethyl ether). The filtrate was then concentrated under reduced pressure and purification by flash chromatography on silica gel (gradient of diethyl ether/pentane = 1:20 to 1:10) gave the desired product **39** as a colorless oil (195 mg, 0.31 mmol, 63%, dr = 87:13). ^{1}H NMR (300 MHz, $CDCl_3$) δ (ppm) 7.40 – 7.28 (m, 5H), 5.44 – 5.30 (m, 1H), 5.15 – 5.07 (m, 2H), 4.65 – 4.55 (m, 1H), 3.83 (ddd, J = 9.2, 4.4, 2.5 Hz, 1H, major dia), 3.78 – 3.68 (m, 1H, minor dia), 2.61 – 2.50 (m, 1H), 2.43 – 2.33 (m, 1H), 2.33 – 2.30 (m, 2H), 2.15 – 1.67 (m, 9H), 1.64 – 1,35 (m, 7H), 1.28 – 0.82 (m, 19H), 0.69 (s, 3H, major dia), 0.66 (s, 3H, minor dia), 0.08 (s, 3H, major dia), 0.07 (s, 3H, major dia), 0.043 (s, 3H, minor dia),

---

[12] Vellekoop, A. S.; Smith, R. A. J. *J. Am. Chem. Soc.* **1994**, 116, 2902.

[13] Mander, L. N.; Sethi, S. P. *Tetrahedron Lett.* **1984**, *25*, 5953.

0.039 (s, 3H, minor dia). ^{13}C NMR (75 MHz, $CDCl_3$) δ (ppm) *major dia*: 174.3, 170.7, 139.9, 136.3, 128.7, 128.27, 128.25, 122.6, 74.1, 72.4, 66.3, 56.5, 53.6, 50.3, 42.0, 39.2, 38.3, 37.2, 36.8, 32.0, 31.9, 30.5, 27.9, 27.7, 26.3, 25.0, 24.4, 21.6, 20.9, 19.5, 18.3, 12.2, -3.4, -4.0. HRMS (ESI) Calcd for $C_{38}H_{59}O_5Si$: 623.4126 $[M+H]^+$, Found: 623.4105.

Diethyl 2-(2-(trimethylsilyloxy)cyclohexyl) malonate (40)

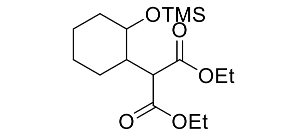

Chemical Formula: $C_{16}H_{30}O_5Si$
Molecular Weight: 330,50

Following general procedure B, the reaction was carried out with diethyl 2-(ethoxycarbonothioylthio)malonate **2'g (**280 mg, 1.00 mmol, 1.0 equiv), cyclohexenyloxytrimethylsilane **37c** (340.6 mg, 2.00 mmol, 2.0 equiv), 4-*tert*-butylcatechol (498 mg, 3.00 mmol, 3.0 equiv), Et_3B (2.5mL, 2.50 mmol, 2.5 equiv). Flash chromatography on silica gel (2-6% ether in pentane) gave the desired product **9cg** as a colorless oil (278 mg, 84%, *cis*:*trans* = 5:1). Spectral data are in accordance with the literature report.[7] *1st diastereomer (trans):* ^{1}H NMR (300 MHz, $CDCl_3$) δ (ppm) 3.58 – 3.50 (m, 5H), 3.40 (d, J = 11.0 Hz, 1H), 2.13-1.1 (m, 15H) 0.05 (s, 9H). ^{13}C NMR (75 MHz, $CDCl_3$) δ (ppm) 169.4, 168.9, 67.7, 61.3, 61.2, 54.9, 42.1, 33.5, 25.6, 24.1, 19.6, 14.3, 0.3. *2nd diastereomer (cis):* ^{1}H NMR (300 MHz, $CDCl_3$) δ (ppm) 4.22 – 4.11 (m, 5H), 3.70 (d, J = 4.3 Hz, 1H), 2.13-1.01 (m, 15 H), 0.10 (s, 9H). ^{13}C NMR (75 MHz, $CDCl_3$) δ (ppm) 169.8, 169.0, 72.9, 61.1, 60.9, 52.2, 45.8, 36.3, 27.2, 25.7, 24.9, 13.8, 14.2, 0.5. IR (neat, cm^{-1}): 2980, 2934, 2858, 1750, 1729, 1447, 1368, 1293, 1249, 1140, 1091, 1020, 910, 884, 836, 748, 684, 524. HRMS (ESI) Calcd. for $C_{16}H_{30}O_5NaSi$: 353.1755 $[M+Na]^+$, Found: 353.1754.

Ethyl 2-((8*R*,9*S*,13*S*,14*S*,16*S*,17*S*)-17-((*tert*-butyldimethylsilyl)oxy)-3-methoxy-13-methyl-7,8,9,11,12,13,14,15,16,17-decahydro-6*H*-cyclopenta[a]phenanthren-16-yl)acetate (41)

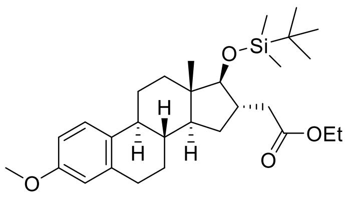

Chemical Formula: $C_{29}H_{46}O_4Si$
Exact Mass: 486,32

Under nitrogen, to a solution of olefin **37d**[13] (199 mg, 0.50 mmol, 1.0 equiv) and xanthate **2'a** (312 mg, 1.50 mmol, 3.0 equiv) in dry DCM (5.0 mL) was added 4-*tert*-butylcatechol (166 mg, 1.00 mmol, 2.0 equiv), followed by slow addition of Et_3B (0.6 mL, 1 M in *n*-hexane) while the needle was immersed in the solution. The resulting reaction mixture was stirred open to air for 1 h at rt with a $CaCl_2$ trap. The solution was filtered over a small pad of neutral alox (eluted with diethyl ether). The filtrate was then concentrated under reduced pressure and purification by flash chromatography on silica gel (gradient of diethyl ether/pentane = 1:40 to 1:20) gave the desired product **41** as a white solid (144 mg, 0.30 mmol, 59%). Suitable crystals were obtained upon slow diffusion of heptane in a solution of **41** in Et_2O in the fridge overnight. ^{1}H NMR (300 MHz, $CDCl_3$) δ (ppm) 7.19 (d, J = 7.7 Hz, 1H), 6.71 (dd, J = 8.6, 2.8 Hz, 1H), 6.62

(d, J = 2.7 Hz, 1H), 4.14 (q, J = 7.1 Hz, 2H), 3.77 (s, 3H), 3.31 (d, J = 7.1 Hz, 1H), 2.88 – 2.78 (m, 2H), 2.67 – 2.54 (m, 1H), 2.35 – 2.11 (m, 4H), 1.86 (dt, J = 12.5, 2.7 Hz, 2H), 1.75 – 1.62 (m, 1H), 1.53 – 1.35 (m, 3H), 1.39 – 1.17 (m, 3H), 1.27 (t, J = 7.1 Hz, 3H), 0.91 (s, 9H), 0.80 (s, 3H), 0.07 (s, 3H), 0.06 (s, 3H). ^{13}C NMR (75 MHz, $CDCl_3$) δ (ppm) 173.5, 157.6, 138.2, 132.8, 126.4, 113.9, 111.6, 86.9, 60.4, 55.4, 48.2, 44.3, 44.1, 40.6, 39.1, 38.8, 37.5, 30.0, 29.7, 27.3, 26.5, 26.0, 18.2, 14.4, 12.2, -3.8, -3.9. IR (neat, cm^{-1}): 2928, 2855, 1733, 1611, 1500, 1463, 1254, 1179, 1155, 1135, 1087, 1036, 1026, 882, 870, 836, 777, 769. HRMS (ESI) Calcd for $C_{29}H_{47}O_4Si$: 487.3238 $[M+H]^+$, Found: 487.3218. $[\alpha]_D$ = +10.5 (c = 0.396, $CHCl_3$). mp: 76.1-77.3 °C.

X-Ray crystal structure report of **41** (CCDC number: 2031380):

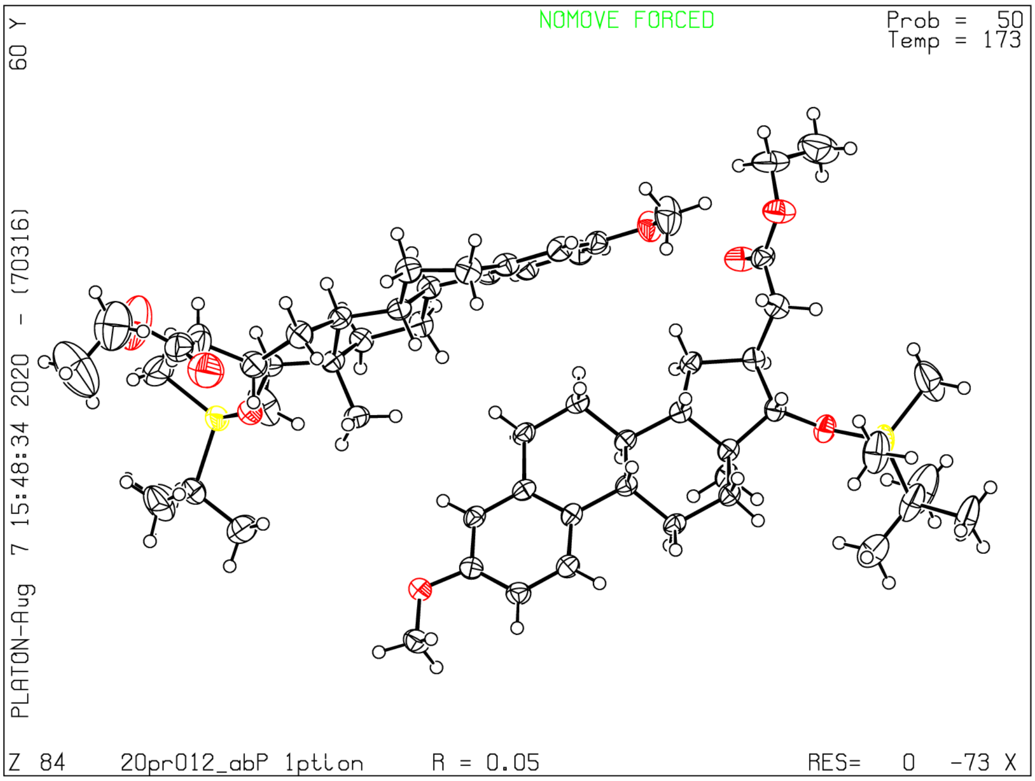

Table 1. Crystal data and structure refinement for **41**.

Empirical formula	$C_{29}H_{46}O_4Si$
Formula weight	486.75
Temperature/K	173.00(10)
Crystal system	triclinic
Space group	P1
a/Å	6.44725(4)
b/Å	10.18619(6)

c/Å	22.76512(12)
α/°	101.7853(5)
β/°	91.4615(5)
γ/°	98.5773(5)
Volume/Å^3	1444.746(15)
Z	2
ρ_{calc}g/cm^3	1.119
μ/mm^{-1}	0.945
F(000)	532.0
Crystal size/mm^3	0.251 × 0.074 × 0.045
Radiation	Cu Kα (λ = 1.54184)
2Θ range for data collection/°	7.948 to 149.002
Index ranges	-8 ≤ h ≤ 8, -12 ≤ k ≤ 11, -28 ≤ l ≤ 28
Reflections collected	29329
Independent reflections	9878 [R_{int} = 0.0301, R_{sigma} = 0.0277]
Data/restraints/parameters	9878/3/629
Goodness-of-fit on F^2	1.081
Final R indexes [I>=2σ (I)]	R_1 = 0.0501, wR_2 = 0.1380
Final R indexes [all data]	R_1 = 0.0528, wR_2 = 0.1512
Largest diff. peak/hole / e Å^{-3}	0.56/-0.29
Flack parameter	0.01(2)

(*E*)-Ethyl 4-(2-oxopyrrolidin-1-yl)but-3-enoate (45)

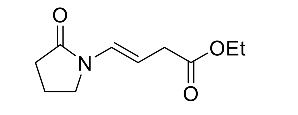

Chemical Formula: $C_{10}H_{15}NO_3$
Molecular Weight: 197,23

Following general procedure C, the reaction was carried out with ethyl iodoacetate **2a (**107 mg, 0.50 mmol, 1.0 equiv), enamide **44a** (277.8 mg, 2.50 mmol, 5.0 equiv) Et_3B (0.6 mL, 0.6 mmol, 1.2 equiv). Flash chromatography on silica gel (pentane:Et_2O = 1:1) gave the desired product **45** as a pale yellow oil (68 mg, 0.34 mmol, 69% yield). ^{1}H NMR (300 MHz, $CDCl_3$) δ (ppm) 6.97 (d, J = 14.5 Hz, 1H), 5.02 (dt, J = 14.5, 7.3 Hz, 1H), 4.14 (q, J = 7.1 Hz, 2H), 3.60 – 3.50 (m, 2H), 3.09 (dd, J = 7.3, 1.3 Hz, 2H), 2.48 (t, J = 8.1 Hz, 2H), 2.17 – 2.06 (m, 2H), 1.26 (t, J = 7.1 Hz, 3H). ^{13}C NMR (75 MHz, $CDCl_3$) δ (ppm) 173.2, 172.1, 126.7, 103.6,

60.9, 45.3, 35.7, 31.3, 17.6, 14.3. HRMS (ESI) Calcd. For $C_{10}H_{16}NO_3$: 198.1125 $[M+H]^+$, Found: 198.1122.

(*E*)-Phenyl 4-(2-oxopyrrolidin-1-yl) but-3-enoate (46)

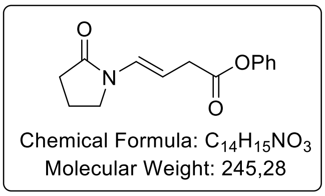

Following general procedure C, the reaction was carried out with phenyl iodoacetate **2c (**131 mg, 0.5 mmol, 1.0 equiv), enamide **44a** (278mg, 2.5 mmol, 5.0 equiv) Et_3B (0.6 mL, 0.6 mmol, 1.2 equiv). Flash chromatography on silica gel (pentane:Et_2O = 1:1) gave the desired product **46** as a pale yellow oil (74 mg, 0.30 mmol, 60% yield). ^1H NMR (300 MHz, $CDCl_3$) δ 7.38 (td, J = 7.8, 2.3 Hz, 2H), 7.30 – 7.19 (m, 1H), 7.14 – 7.01 (m, 3H), 5.11 (dtd, J = 14.5, 7.3, 2.4 Hz, 1H), 3.57 (dd, J = 8.4, 5.9 Hz, 2H), 3.36 (dt, J = 7.4, 1.8 Hz, 2H), 2.50 (td, J = 8.1, 2.4 Hz, 2H), 2.19 – 2.04 (m, 2H). ^{13}C NMR (75 MHz, $CDCl_3$) δ (ppm) 173.1, 170.6, 150.7, 129.5, 127.1, 125.9, 121.5, 102.7, 45.2, 35.6, 31.2, 17.5. IR (neat, cm^{-1}): 2948, 2899, 1754, 1690, 1662, 1591, 1485, 1460, 1415, 1285, 1191, 1161, 1129, 1069, 928, 817, 733, 688, 647. HRMS (ESI): calcd for $C_{14}H_{16}O_3N$ $[M+H]^+$: 246.1125, found: 246.1129.

(*E*)-Ethyl 2-methyl-4-(2-oxopyrrolidin-1-yl) but-3-enoate (47)

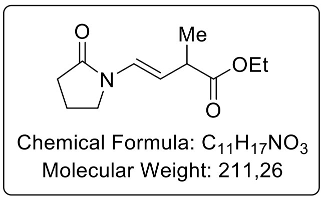

Following general procedure C, the reaction was carried out with ethyl 2-iodopropanoate **2d (**114 mg, 0.5 mmol, 1.0 equiv), enamide **44a** (278 mg, 2.5 mmol, 5.0 equiv), Et_3B (0.6 mL, 0.6 mmol, 1.2 equiv). Flash chromatography on silica gel (pentane:Et_2O = 1:1) gave the desired product **47** as a pale yellow oil (65 mg, 0.31 mmol, 62% yield). ^1H NMR (300 MHz, $CDCl_3$) δ (ppm) 6.97 (dd, J = 14.6, 1.0 Hz, 1H), 5.01 (dd, J = 14.5, 8.4 Hz, 1H), 4.12 (qd, J = 7.2, 1.0 Hz, 2H), 3.53 – 3.44 (m, 2H), 3.15 (dqd, J = 8.1, 7.0, 1.0 Hz, 1H), 2.47 (dd, J = 8.7, 7.5 Hz, 2H), 2.09 (td, J = 7.8, 1.3 Hz, 2H), 1.31 – 1.22 (m, 6H). ^{13}C NMR (75 MHz, $CDCl_3$) δ (ppm) 175.1, 173.3, 125.1, 111.0, 60.8, 45.3, 41.0, 31.3, 18.3, 17.6, 14.3. IR (neat, cm^{-1}): 2978, 2935, 2885, 1698, 1417, 1378, 1266, 1180, 1110, 733, 700. HRMS (ESI): calcd for $C_{11}H_{18}O_3N$ $[M+H]^+$: 212.1281, found: 212.1287.

Ethyl 4-(2-oxopyrrolidin-1-yl) butanoate (48)

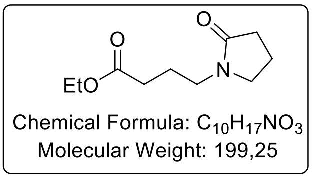

Following general procedure B, the reaction was carried out with ethyl 2-(ethoxycarbonothioylthio)acetate **2'a** (365 mg, 1.75 mmol), 1-vinylpyrrolidin-2-one **44a** (390 mg, 3.50 mmol), 4-*tert*-butylcatechol (874 mg, 5.26 mmol) and Et_3B (5.26 mL, 5.26 mmol, 1M solution in *n*-hexane) in DCM (17.5 mL). Flash chromatography on silica gel (5% MeOH in ether) gave the desired product **48** as yellowish green oil (300 mg, 1.51 mmol, 86%).

^{1}H NMR (300 MHz, $CDCl_3$) δ (ppm) 4.11 (q, J = 7.1 Hz, 2H), 3.38 (t, J = 7.0 Hz, 2H), 3.29 (t, J = 7.2 Hz, 2H), 2.36 (t, J = 8.1 Hz, 2H), 2.30 (t, J = 7.5 Hz, 2H), 2.07 – 1.93 (m, 2H), 1.83 (p, J = 7.5 Hz, 2H), 1.23 (t, J = 7.1 Hz, 3H). ^{13}C NMR (75 MHz, $CDCl_3$) δ (ppm) 175.2, 173.1, 60.6, 47.2, 42.0, 31.7, 31.1, 22.7, 18.0, 14.3. HRMS (ESI) Calcd. For $C_{10}H_{18}O_3N$: 200.1281 $[M+H]^+$, Found: 200.1283.

Benzyl 4-(2-oxopyrrolidin-1-yl)butanoate (49)

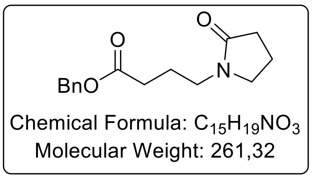

Following general procedure B, the reaction was carried out with benzyl 2-(ethoxycarbonothioylthio) acetate **2’b** (128 mg, 0.50 mmol, 1.0 equiv), 1-vinylpyrrolidin-2-one **44a** (111 mg, 1.00 mmol, 2.0 equiv), 4-*tert*-butylcatechol (249 mg, 1.50 mmol, 3.0 equiv) and Et_3B (1.5 mL, 1.50 mmol, 3.0 equiv, 1 M solution in *n*-hexane) in DCM (5.0 mL). Flash chromatography on silica gel (EtOAc/pentane = 90:10) gave the desired product **49** as a pale yellow oil (107 mg, 0.41 mmol, 82% yield). ^{1}H NMR (300 MHz, $CDCl_3$) δ (ppm) 7.4–7.28 (m, 5H), 5.11 (s, 2H), 3.36 (t, J = 7.0 Hz, 2H), 3.30 (t, J = 7.1 Hz, 2H), 2.37 (t, J = 7.4 Hz, 2H), 2.36 (t, J = 7.9 Hz, 2H), 1.99 (quin, 2H), 1.86 (quin, 2H). ^{13}C NMR (75 MHz, $CDCl_3$) δ (ppm) 175.3, 172.9, 136.0, 128.7, 128.4, 128.3, 66.5, 47.3, 42.0, 31.7, 31.1, 22.7, 18.0. IR (Neat, cm^{-1}): 2934, 2875, 2358, 2161, 1730, 1673, 1495, 1455, 1424, 1286, 1264, 1149, 1089, 971, 738, 698**.** HRMS (ESI) Calcd. for $C_{15}H_{20}NO_3$: 262.1438 $[M+H]^+$, Found: 262.1438.

Phenyl 4-(2-oxopyrrolidin-1-yl)butanoate (50)

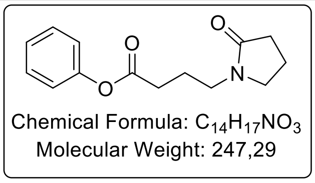

Following general procedure B, the reaction was carried out with phenyl 2-(ethoxycarbonothioylthio) acetate **2’c** (128 mg, 0.50 mmol, 1.0 equiv), 1-vinylpyrrolidin-2-one **44a** (111 mg, 1.00 mmol, 2.0 equiv), 4-*tert*-butylcatechol (249 mg, 1.50 mmol, 3.0 equiv) and Et_3B (1.5 mL, 1.50 mmol, 3.0 equiv, 1 M solution in *n*-hexane) in DCM (5.0 mL). Flash chromatography on silica gel (EtOAc/pentane = 90:10) gave the desired product **50** as a pale yellow oil (97 mg, 0.39 mmol, 79% yield). ^{1}H NMR (300 MHz, $CDCl_3$) δ (ppm) 7.36 (ddd, J = 7.8, 4.6, 2.1 Hz, 2H), 7.25- 7.18 (m, 1H), 7.10-7.05 (m, 2H), 3.40 (dd, J = 13.3, 6.8 Hz, 4H), 2.59 (t, J = 7.4 Hz, 2H), 2.39 (t, J = 8.1 Hz, 2H), 2.08-1.90 (m, 4H). ^{13}C NMR (75 MHz, $CDCl_3$) δ (ppm) 175.4, 171.7, 150.8, 129.5, 125.9, 121.6,

47.3, 42.0, 31.7, 31.1, 22.6, 18.1. IR (Neat, cm^{-1}): 2934, 2875, 2358, 2158, 2031, 1754, 1673, 1591, 1492, 1462, 1424, 1365, 1286, 1192, 1161, 1131, 932, 752, 690. HRMS (ESI) Calcd. for $C_{14}H_{18}NO_3$: 248.1281 $[M+H]^+$, Found: 248.1281.

Benzyl 4-(*N*-phenylacetamido)butanoate (51)

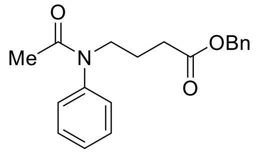

Chemical Formula: $C_{19}H_{21}NO_3$
Molecular Weight: 311,38

Following general procedure B, the reaction was carried out with olefin **44b**[14] (161 mg, 1.00 mmol, 2.0 equiv), xanthate **2'b** (135 mg, 0.50 mmol, 1.0 equiv), 4-*tert*-butylcatechol (249 mg, 1.50 mmol), Et_3B (1.5 mL, 1.50 mmol) in DCM (5.0 mL) and it took 1 h for the reaction to go to completion. Flash chromatography on silica gel (TBME/heptane = 7:3) gave the desired product **51** as a colorless oil (110 mg, 0.35 mmol, 71% yield). ^{1}H NMR (300 MHz, $CDCl_3$) δ (ppm) 7.45 – 7.27 (m, 8H), 7.19 – 7.12 (m, 2H), 5.08 (s, 2H), 3.80 – 3.67 (m, 2H), 2.40 (t, J = 7.6 Hz, 2H), 1.92 – 1.83 (m, 2H), 1.82 (s, 3H). ^{13}C NMR (75 MHz, $CDCl_3$) δ (ppm) 172.9, 170.5, 143.0, 136.1, 129.9, 128.7, 128.3, 128.2, 128.1, 66.4, 48.3, 31.7, 23.3, 22.9. IR (neat, cm^{-1}): 2931, 1731, 1653, 1595, 1495, 1454, 1393, 1295, 1276, 1210, 1157, 1075, 1026, 976, 768, 741, 697, 564. HRMS (ESI) Calcd for $C_{19}H_{22}O_3N$: 312.1594 $[M+H]^+$, Found: 312.1579.

Diethyl 2-(2-(1,3-dioxoisoindolin-2-yl)ethyl)malonate (52)

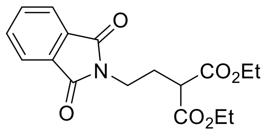

Chemical Formula: $C_{17}H_{19}NO_6$
Molecular Weight: 333,34

Under nitrogen, to a solution of *N*-vinylphthamilide **44c** (87 mg, 0.50 mmol, 1.0 equiv) and xanthate **2'g** (280 mg, 1.00 mmol, 2.0 equiv) in dry DCM (5.0 mL) was added 4-*tert*-butylcatechol (249 mg, 1.50 mmol, 3.0 equiv), followed by slow addition of Et_3B (1.50 mmol, 1.5 mL, 1 M in *n*-hexane) while the needle was immersed in the solution. The resulting reaction mixture was stirred open to air for 2 h at rt with a $CaCl_2$ trap. The solution was filtered over a small pad of neutral alox (eluted with diethyl ether). The filtrate was then concentrated under reduced pressure and purification by flash chromatography on silica gel (gradient of diethyl ether/pentane = 1:2 to 2:3) gave the desired product **52** as a colorless oil (143 mg, 0.43 mmol, 86% yield). ^{1}H NMR (300 MHz, $CDCl_3$) δ (ppm) 7.83 (dt, J = 6.9, 3.5 Hz, 2H), 7.78 – 7.66 (m, 2H), 4.17 (qd, J = 7.1, 2.2 Hz, 4H), 3.79 (t, J = 6.8 Hz, 2H), 3.38 (t, J = 7.3 Hz, 1H), 2.28 (q, J = 7.0 Hz, 2H), 1.25 (t, J = 7.1 Hz, 6H). ^{13}C NMR (75 MHz, $CDCl_3$) δ (ppm) 168.8, 168.3, 134.1, 132.2, 123.4, 61.8, 49.8, 35.9, 27.7, 14.1. IR (neat, cm^{-1}): 2983, 2937, 1773,

---

[14] Feltenberger, J. B.; Hayashi, R.; Tang, Y.; Babiash, E. S. C.; Hsung, R. P. *Org. Lett.* **2009**, *11*, 3666.

1706, 1467, 1439, 1395, 1368, 1336, 1272, 1245, 1174, 1153, 1121, 1011, 857, 718. HRMS (ESI) Calcd for $C_{17}H_{20}O_6N$: 334.1285 $[M+H]^+$, Found: 334.1290.

Benzyl 3-methyl-4-(2-oxopyrrolidin-1-yl)butanoate (53)

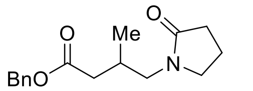

Chemical Formula: $C_{16}H_{21}NO_3$
Molecular Weight: 275,35

Following general procedure B, the reaction was carried out with olefin **44d**[15] (125 mg, 1.00 mmol), xanthate **2'b** (135 mg, 0.5 mmol), 4-*tert*-butylcatechol (249 mg, 1.50 mmol), Et_3B (1.5 mL, 1.50 mmol) in DCM (5 mL) and it took 2 h for the reaction to go to completion. Flash chromatography on silica gel (ethyl acetate/heptane = 2:1) gave the desired product **53** as a colorless oil (77 mg, 0.28 mmol, 56% yield). ^1H NMR (300 MHz, $CDCl_3$) δ (ppm) 7.40 – 7.28 (m, 5H), 5.11 (s, 2H), 3.43 – 3.28 (m, 2H), 3.21 (dd, J = 13.5, 7.2 Hz, 1H), 3.11 (dd, J = 13.5, 6.8 Hz, 1H), 2.42 – 2.26 (m, 4H), 2.25 – 2.16 (m, 1H), 2.07 – 1.90 (m, 2H), 0.94 (d, J = 6.5 Hz, 3H). ^{13}C NMR (75 MHz, $CDCl_3$) δ (ppm) 175.5, 172.7, 136.0, 128.7, 128.4, 128.3, 66.4, 48.3, 47.6, 39.2, 31.0, 29.0, 18.1, 17.9. IR (neat, cm^{-1}): 2961, 2916, 2874, 1729, 1678, 1456, 1424, 1385, 1285, 1267, 1205, 1153, 1109, 1082, 981, 740, 698. HRMS (ESI) Calcd for $C_{16}H_{22}O_3N$: 276.1594 $[M+H]^+$, Found: 276.1598.

Benzyl 4-(2-oxooxazolidin-3-yl)butanoate (54)

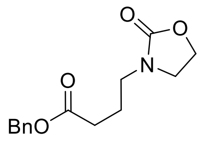

Chemical Formula: $C_{14}H_{17}NO_4$
Molecular Weight: 263,29

Following general procedure B, the reaction was carried out with olefin **44e**[16] (113 mg, 1.00 mmol), xanthate **2'b** (135 mg, 0.50 mmol), 4-*tert*-butylcatechol (249 mg, 1.50 mmol), Et_3B (1.5 mL, 1.50 mmol) in DCM (5.0 mL) and it took 1.5 h for the reaction to go to completion. Flash chromatography on silica gel (gradient of ethyl acetate/heptane = 1:1 to 4:1) gave the desired product **54** as a colorless oil (117 mg, 0.44 mmol, 89% yield). ^1H NMR (300 MHz, $CDCl_3$) δ (ppm) 7.41 – 7.27 (m, 5H), 5.11 (s, 2H), 4.32 – 4.22 (m, 2H), 3.58 – 3.47 (m, 2H), 3.29 (t, J = 7.1 Hz, 2H), 2.42 (t, J = 7.3 Hz, 2H), 1.90 (p, J = 7.2 Hz, 2H). ^{13}C NMR (75 MHz, $CDCl_3$) δ (ppm) 172.8, 158.6, 135.9, 128.7, 128.4, 128.3, 66.5, 61.8, 44.7, 43.8, 31.4, 22.7. IR (neat, cm^{-1}): 2921, 1728, 1483, 1427, 1261, 1153, 1096, 1046, 968, 760, 752, 740, 697. HRMS (ESI) Calcd for $C_{14}H_{18}O_4N$: 264.1230 $[M+H]^+$, Found: 264.1234.

---

[15] Xu, H.; Zi, Y.; Xu, X.; Wang, S.; Ji, S. *Tetrahedron* **2013**, *69*, 1600.

[16] Gaulon, C. ; Gizecki, P. ; Dhal, R. ; Dujardin, G. *Synlett* **2002**, 952.

Benzyl 4-(3-acetyl-2-oxoimidazolidin-1-yl)butanoate (55)

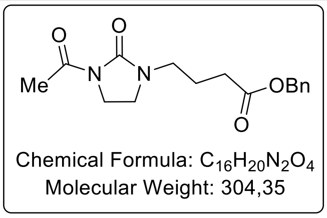

Following general procedure B, the reaction was carried out with olefin **44f** (154 mg, 1.00 mmol, 2.0 equiv), xanthate **2'b** (135 mg, 0.50 mmol, 1.0 equiv), 4-*tert*-butylcatechol (249 mg, 1.50 mmol), Et_3B (1.5 mL, 1.50 mmol) in DCM (5.0 mL) and it took 1 h for the reaction to go to completion. Flash chromatography on silica gel (ethyl acetate/heptane = 1:1 to 3:2) gave the desired product **55** as a light yellow oil (135 mg, 0.44 mmol, 89% yield). ^{1}H NMR (300 MHz, $CDCl_3$) δ (ppm) 7.39 – 7.29 (m, 5H), 5.11 (s, 2H), 3.80 (t, J = 8.1 Hz, 2H), 3.43 – 3.34 (m, 2H), 3.31 (t, J = 7.0 Hz, 2H), 2.47 (s, 3H), 2.41 (t, J = 7.3 Hz, 2H), 1.90 (p, J = 7.2 Hz, 2H). ^{13}C NMR (75 MHz, $CDCl_3$) δ (ppm) 172.8, 170.9, 155.0, 135.9, 128.7, 128.5, 128.4, 66.6, 43.3, 40.9, 39.6, 31.5, 23.4, 22.5. IR (neat, cm^{-1}): 2934, 1719, 1675, 1482, 1431, 1379, 1356, 1335, 1259, 1150, 1107, 743, 698, 610. HRMS (ESI) Calcd for $C_{16}H_{21}O_4N_2$: 305.1496 [M+H]$^+$, Found: 305.1488.

Benzyl 3-(2-(benzyloxy)-2-oxoethyl)pyrrolidine-1-carboxylate (56)

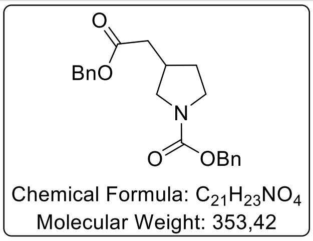

Following general procedure B, the reaction was carried out with olefin **44g** (203 mg, 1.00 mmol, 2.0 equiv), xanthate **2'b** (135 mg, 0.50 mmol, 1.0 equiv), 4-*tert*-butylcatechol (249 mg, 1.50 mmol), Et_3B (1.5 mL, 1.50 mmol) in DCM (5.0 mL) and it took 2 h for the reaction to go to completion. Flash chromatography on silica gel (TBME/pentane = 3:7) gave the desired product **56** as a colorless oil (60 mg, 0.17 mmol, 34% yield). ^{1}H NMR (300 MHz, $CDCl_3$) δ (ppm) 7.49 – 7.30 (m, 10H), 5.15 (s, 4H), 3.71 (dt, J = 10.5, 7.0 Hz, 1H), 3.56 (dp, J = 11.2, 3.8 Hz, 1H), 3.40 (q, J = 9.5, 8.9 Hz, 1H), 3.06 (td, J = 11.3, 7.7 Hz, 1H), 2.62 (dq, J = 15.1, 7.5 Hz, 1H), 2.47 (d, J = 7.9 Hz, 2H), 2.21 – 2.03 (m, 1H), 1.60 (dt, J = 12.3, 8.2 Hz, 1H). ^{13}C NMR (75 MHz, $CDCl_3$) δ (ppm) 171.9, 154.9, 137.1, 135.8, 128.7, 128.6, 128.5, 128.4, 128.05, 127.99, 66.8, 66.6, 51.5, 51.1, 45.7, 45.4, 37.7, 35.4, 34.6, 31.6, 30.9. IR (neat, cm^{-1}): 2950, 2875, 1732, 1697, 1415, 1358, 1154, 1117, 966, 767, 736, 695, 603. HRMS (ESI) Calcd for $C_{21}H_{24}O_4N$: 354.1700 [M+H]$^+$, Found: 354.1700.

Benzyl 3-(2-(benzyloxy)-2-oxoethyl)piperidine-1-carboxylate (57)

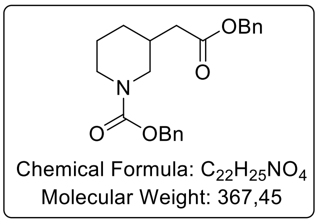

Following general procedure B, the reaction was carried out with olefin **44h**[17] (217 mg, 1.00 mmol, 2.0 equiv), xanthate **2'b** (135 mg, 0.50 mmol, 1.0 equiv), 4-*tert*-butylcatechol (249 mg, 1.50 mmol),

[17] De Simone, F.; Saget, T.; Benfatti, F.; Almeida, S.; Waser, J. *Chem. Eur. J.* **2011**, *17*, 14527.

Et_3B (1.5 mL, 1.50 mmol) in DCM (5.0 mL) and it took 2 h for the reaction to go to completion. Flash chromatography on silica gel (diethyl ether/pentane = 1:2) gave the desired product **57** as a colorless oil (146 mg, 0.40 mmol, 79% yield). ^{1}H NMR (300 MHz, $CDCl_3$) δ (ppm) 7.43 – 7.27 (m, 10H), 5.13 (s, 2H), 5.11 (s, 2H), 4.15 – 3.87 (m, 2H), 2.90 (ddd, *J* = 13.6, 10.8, 3.3 Hz, 1H), 2.70 (br, 1H), 2.38 – 2.20 (m, 2H), 2.04 (ddp, *J* = 10.3, 7.1, 3.5 Hz, 1H), 1.85 (dt, *J* = 13.1, 4.3 Hz, 1H), 1.65 (ddd, *J* = 17.1, 8.1, 4.2 Hz, 1H), 1.49 (h, *J* = 10.9, 9.3 Hz, 1H), 1.29 – 1.10 (m, 1H). ^{13}C NMR (75 MHz, $CDCl_3$) δ (ppm) 171.9, 155.4, 137.0, 136.0, 128.7, 128.6, 128.38, 128.35, 128.0, 127.9, 67.1, 66.4, 49.3, 44.5, 38.3, 32.9, 30.5 (a CH_2 group was detected at 24.4 ppm as broad line). IR (neat, cm^{-1}): 3031, 2934, 2853, 1732, 1693, 1429, 1259, 1234, 1151, 1130, 1083, 1028, 977, 735, 695. HRMS (ESI) Calcd for $C_{22}H_{26}O_4N$: 368.1856 $[M+H]^+$, Found: 368.1846.

Benzyl 3-((2-oxooxazolidin-3-yl)methyl)heptanoate (58)

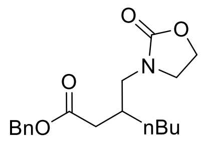

Chemical Formula: $C_{18}H_{25}NO_4$
Molecular Weight: 319,40

Following general procedure B, the reaction was carried out with olefin **44i**[18] (169 mg, 1.00 mmol), xanthate **2'b** (135 mg, 0.50 mmol), *tert*-butylcatechol (249 mg, 1.50 mmol), Et_3B (0.75 mL, 0.75 mmol) in DCM (5.0 mL) and it took 3 h for the reaction to go to completion. Flash chromatography on silica gel (ethyl acetate/heptane = 1:2) gave the desired product **58** as a colorless oil (108 mg, 0.34 mmol, 68% yield). ^{1}H NMR (300 MHz, $CDCl_3$) δ (ppm) 7.43 – 7.27 (m, 5H), 5.13 (d, *J* = 12.2 Hz, 1H), 5.08 (d, *J* = 12.3 Hz, 1H), 4.33 – 4.14 (m, 2H), 3.57 (td, *J* = 8.7, 6.5 Hz, 1H), 3.47 (ddd, *J* = 9.2, 8.3, 7.3 Hz, 1H), 3.23 (dd, *J* = 13.9, 8.4 Hz, 1H), 3.13 (dd, *J* = 13.9, 6.1 Hz, 1H), 2.35 (dd, *J* = 6.5, 2.1 Hz, 2H), 2.19 (tdd, *J* = 8.7, 5.0, 3.4 Hz, 1H), 1.36 – 1.21 (m, 6H), 0.97 – 0.77 (m, 3H). ^{13}C NMR (75 MHz, $CDCl_3$) δ (ppm) 173.0, 159.0, 135.9, 128.7, 128.43, 128.41, 66.5, 61.8, 48.9, 45.2, 37.1, 33.8, 32.0, 28.8, 22.9, 14.1. IR (neat, cm^{-1}): 2955, 2926, 2858, 1730, 1483, 1428, 1380, 1260, 1157, 1112, 1041, 970, 760, 737, 696. HRMS (ESI) Calcd for $C_{18}H_{26}O_4N$: 320.1856 $[M+H]^+$, Found: 320.1862.

Benzyl 3-methyl-4-(2-oxooxazolidin-3-yl)pentanoate (59)

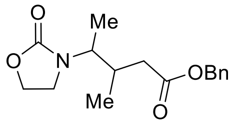

Chemical Formula: $C_{16}H_{21}NO_4$
Molecular Weight: 291,35

Under nitrogen, to a solution of olefin **44j**[3] (71 mg, 0.50 mmol, 1.0 equiv) and xanthate **2'b** (270 mg, 1.00 mmol, 2.0 equiv) in dry DCM (5.0 mL) was added 4-*tert*-butylcatechol (249 mg, 1.50 mmol, 3.0 equiv), followed by slow addition of Et_3B (1.5 mL, 1 M in *n*-hexane) while the needle was immersed in the solution. The resulting reaction mixture was stirred open to air for 1 h at rt with a $CaCl_2$ trap. The solution was filtered over a small pad of

---

[18] Koleoso, O. K.; Elsegood, M. R. J.; Teat, S. J.; Kimber, M. C. *Org. Lett.* **2018**, *20*, 1003.

neutral alox (eluted with diethyl ether). The filtrate was then concentrated under reduced pressure and purification by flash chromatography on silica gel (gradient of diethyl ether/pentane = 1:1 to 1:0) gave the first diastereoisomer as a colorless oil (46 mg, 0.16 mmol, 32% yield) and the second diastereoisomer as a colorless oil (45 mg, 0.16 mmol, 32% yield). *1st diastereoisomer:* ^{1}H NMR (300 MHz, $CDCl_3$) δ (ppm) 7.42 – 7.29 (m, 5H), 5.11 (d, J = 12.3Hz, 1H), 5.10 (d, J = 12.3Hz, 1H), 4.29 (td, J = 8.7, 5.8 Hz, 1H), 4.23 – 4.13 (m, 1H), 3.67 (dq, J = 9.7, 6.7 Hz, 1H), 3.55 – 3.40 (m, 2H), 2.55 – 2.43 (m, 1H), 2.25 – 2.04 (m, 2H), 1.19 (d, J = 6.8 Hz, 3H), 0.99 (d, J = 6.5 Hz, 3H). ^{13}C NMR (75 MHz, $CDCl_3$) δ (ppm) 173.1, 158.4, 136.0, 128.7, 128.41, 128.36, 66.5, 62.1, 54.0, 40.2, 39.3, 34.2, 17.7, 16.0. IR (neat, cm^{-1}): 2972, 2930, 1731, 1483, 1455, 1420, 1383, 1252, 1173, 1061, 1031, 1005, 975, 761, 697. HRMS (ESI) Calcd for $C_{16}H_{22}O_4N$: 292.1543 $[M+H]^+$, Found: 292.1537. *2nd diastereoisomer:* ^{1}H NMR (300 MHz, $CDCl_3$) δ (ppm) 7.42 – 7.28 (m, 5H), 5.13 (s, 2H), 4.40 – 4.23 (m, 2H), 3.76 (dq, J = 8.7, 6.8 Hz, 1H), 3.60 – 3.36 (m, 2H), 2.57 – 2.45 (m, 1H), 2.27 – 2.05 (m, 2H), 1.18 (d, J = 6.9 Hz, 3H), 0.96 (d, J = 6.4 Hz, 3H). ^{13}C NMR (75 MHz, $CDCl_3$) δ (ppm) 172.5, 158.4, 135.9, 128.7, 128.5, 66.6, 62.0, 53.3, 40.6, 38.8, 34.4, 16.9, 15.9. IR (neat, cm^{-1}): 2971, 2925, 1728, 1483, 1455, 1421, 1382, 1253, 1180, 1145, 1058, 1031, 979, 760, 697. HRMS (ESI) Calcd for $C_{16}H_{22}O_4N$: 292.1543 $[M+H]^+$, Found: 292.1534.

Benzyl 2-(2-(2-oxooxazolidin-3-yl)cyclohexyl)acetate (60)

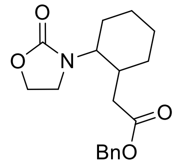

Chemical Formula: $C_{18}H_{23}NO_4$
Molecular Weight: 317,39

Under nitrogen, to a solution of olefin **44k** (84 mg, 0.50 mmol, 1.0 equiv) and xanthate **2'b** (270 mg, 1.00 mmol, 2.0 equiv) in dry DCM (5.0 mL) was added 4-*tert*-butylcatechol (249 mg, 1.50 mmol, 3.0 equiv), followed by slow addition of Et_3B (1.5 mL, 1 M in *n*-hexane) while the needle was immersed in the solution. The resulting reaction mixture was stirred open to air for 3 h at rt with a $CaCl_2$ trap. The solution was filtered over a small pad of neutral alox (eluted with diethyl ether). The filtrate was then concentrated under reduced pressure and purification by flash chromatography on silica gel (ethyl acetate/heptane = 1:1) gave the first diastereoisomer as a colorless oil (29 mg, 0.09 mmol, 18% yield), the second diastereoisomer as a colorless oil (40 mg, 0.13 mmol, 25% yield) and a mixture of the two diastereoisomers (38 mg, 0.12 mmol, 24% yield). *1st diastereoisomer:* ^{1}H NMR (300 MHz, $CDCl_3$) δ (ppm) 7.40 – 7.27 (m, 5H), 5.12 (d, J = 12.3 Hz, 1H), 5.06 (d, J = 12.3 Hz, 1H), 4.33 – 4.22 (m, 1H), 4.15 (q, J = 8.6 Hz, 1H), 3.54 – 3.41 (m, 3H), 2.48 (dd, J = 15.8, 5.4 Hz, 1H), 2.16 (dd, J = 15.9, 7.3 Hz, 1H), 2.06 – 1.90 (m, 1H), 1.90 – 1.65 (m, 4H), 1.52 – 1.31 (m, 2H), 1.31 – 1.06 (m, 2H). ^{13}C NMR (75 MHz, $CDCl_3$) δ (ppm) 173.1, 158.4, 136.0, 128.7, 128.4, 128.3, 66.5, 62.2, 56.8, 40.3, 38.6, 37.3, 32.4, 29.9, 25.5, 25.4. IR (neat, cm^{-1}): 2926, 2855, 1728, 1482,

1450, 1420, 1384, 1249, 1230, 1159, 1111, 1062, 1031, 975, 759, 739, 696. HRMS (ESI) Calcd for $C_{18}H_{24}O_4N$: 318.1700 $[M+H]^+$, Found: 318.1693. *2nd diastereoisomer:* ^1H NMR (300 MHz, $CDCl_3$) δ (ppm) 7.40 – 7.27 (m, 5H), 5.13 (d, J = 12.2 Hz, 1H), 5.07 (d, J = 12.2 Hz, 1H), 4.31 – 4.22 (m, 1H), 4.18 (q, J = 8.5 Hz, 1H), 3.85 (td, J = 7.8, 7.3, 4.6 Hz, 1H), 3.72 – 3.60 (m, 1H), 3.55 (td, J = 8.6, 5.0 Hz, 1H), 2.69 (ddq, J = 9.0, 6.1, 4.5 Hz, 1H), 2.51 (dd, J = 15.1, 6.0 Hz, 1H), 2.39 (dd, J = 15.1, 8.7 Hz, 1H), 1.84 – 1.66 (m, 3H), 1.58 (q, J = 4.9 Hz, 2H), 1.50 – 1.33 (m, 3H). ^{13}C NMR (75 MHz, $CDCl_3$) δ (ppm) 172.8, 158.3, 135.9, 128.7, 128.5, 128.4, 66.6, 62.0, 54.6, 44.2, 35.5, 34.0, 29.3, 25.8, 25.0, 21.0. IR (neat, cm^{-1}): 2929, 2859, 1728, 1482, 1454, 1415, 1252, 1157, 1077, 1058, 1035, 1000, 976, 741, 697. HRMS (ESI) Calcd for $C_{18}H_{24}O_4N$: 318.1700 $[M+H]^+$, Found: 318.1694.

Ethyl 4-((*S*)-2-oxo-4-phenyloxazolidin-3-yl)pentanoate (62)

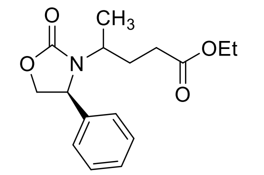

Chemical Formula: $C_{16}H_{21}NO_4$
Molecular Weight: 291,35

Following general procedure B, the reaction was carried out with ethyl 2-(ethoxy-carbonothioylthio)acetate **2'a (**208 mg, 1.00 mmol, 1.0 equiv), (*S*)-4-phenyl-3-(prop-1-en-2-yl)-oxazolidin-2-one **61a** (406 mg, 2.00 mmol, 2.0 equiv) in DCM (10.0 mL), 4-*tert*-butylcatechol (498 mg, 3.00 mmol), Et_3B (3.0 mL, 3.00 mmol, 1 M in *n*-hexane). Flash chromatography on silica gel (20-60% ether in pentane) gave the desired product **62** as a colorless oil (250 mg, 0.86 mmol, 86%, dr = 55:45). *1st diastereoisomer:* ^1H NMR (300 MHz, $CDCl_3$) δ (ppm) 7.43 – 7.29 (m, 5H), 4.74 (dd, J = 8.8, 6.4 Hz, 1H), 4.57 (t, J = 8.8 Hz, 1H), 4.13 (dd, J = 8.6, 6.4 Hz, 1H), 4.1-4.01 (m, 2H), 3.69 – 3.54 (m, 1H), 2.34 – 2.20 (m, 2H), 2.19 – 2.05 (m, 1H), 1.89 – 1.73 (m, 1H), 1.22 (t, J = 7.1 Hz, 3H), 0.91 (d, J = 6.9 Hz, 3H). ^{13}C NMR (75 MHz, $CDCl_3$) δ (ppm) 173.1, 158.1, 139.7, 129.3, 129.2, 127.4, 70.4, 60.6, 58.9, 50.3, 31.5, 28.6, 18.7, 14.3. *2nd diastereoisomer:* ^1H NMR (300 MHz, $CDCl_3$) δ (ppm) 7.43 – 7.31 (m, 5H), 4.79 (dd, J = 9.0, 6.7 Hz, 1H), 4.58 (t, J = 8.9 Hz, 1H), 4.15 (dd, J = 8.7, 6.8 Hz, 1H), 4.06 (q, J = 7.1 Hz, 2H), 3.64 – 3.47 (m, 1H), 2.36 – 2.12 (m, 2H), 1.72 – 1.47 (m, 2H), 1.22 (d, J = 6.9 Hz, 3H), 1.20 (t, J = 7.1 Hz, 3H). ^{13}C NMR (75 MHz, $CDCl_3$) δ (ppm) 173.0, 157.9, 139.4, 129.3 (2C), 127.4, 70.2, 60.5, 60.0, 50.3, 31.5, 29.6, 18.0, 14.3. HRMS (ESI) Calcd. For $C_{16}H_{21}NO_4Na$: 314.1363 $[M+Na]^+$, Found: 314.1364.

Ethyl 4-((*S*)-4-benzyl-2-oxooxazolidin-3-yl)pentanoate (63)

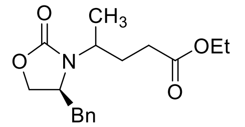

Chemical Formula: $C_{17}H_{23}NO_4$
Molecular Weight: 305,37

Following general procedure B, the reaction was carried out with ethyl 2-(ethoxycarbonothioylthio)acetate **2'a (**208 mg, 1.00 mmol, 1.0 equiv), (*S*)-4-benzyl-3-(prop-1-en-2-yl)-oxazolidin-2-one **61b** (436 mg, 2.00 mmol, 2.0 equiv) in DCM (10.0 mL), 4-*tert*-butylcatechol (500 mg, 3.00 mmol), Et_3B (3.0 mL, 3.00 mmol, 1M in *n*-hexane). Flash

chromatography on silica gel (20-60% ether in pentane) gave the desired product **63** as a colorless oil (220 mg, 72%, dr = 60:40). *1st diastereoisomer*: ^{1}H NMR (300 MHz, $CDCl_3$) δ (ppm) 7.39 – 7.23 (m, 3H), 7.20 – 7.12 (m, 2H), 4.14 (q, *J* = 7.1 Hz, 2H), 4.08 – 3.93 (m, 3H), 3.82 – 3.66 (m, 1H), 3.23 (dd, *J* = 13.1, 3.1 Hz, 1H), 2.62 (dd, *J* = 13.2, 9.5 Hz, 1H), 2.48 – 2.33 (m, 2H), 2.34 – 2.16 (m, 1H), 2.08 – 1.94 (m, 1H), 1.37 (d, *J* = 6.8 Hz, 3H), 1.26 (t, *J* = 7.1 Hz, 3H). ^{13}C NMR (75 MHz, $CDCl_3$) δ (ppm) 173.0, 157.6, 135.6, 129.0, 127.3, 66.9, 60.6, 56.1, 49.9, 40.3, 31.4, 28.7, 19.0, 14.2. *2nd diastereomer:* ^{1}H NMR (300 MHz, $CDCl_3$) δ (ppm) 7.39 – 7.23 (m, 3H), 7.20 – 7.12 (m, 2H), 4.14 (q, *J* = 7.1 Hz, 2H), 4.08 – 3.93 (m, 3H), 3.82 – 3.66 (m, 1H), 3.23 (dd, *J* = 13.1, 3.1 Hz, 1H), 2.72 – 2.61 (m, 1H) , 2.47 – 2.34 (m, 2H), 2.32 – 2.10 (m, 1H), 2.01 – 1.85 (m, 1H), 1.37 (d, *J* = 6.8 Hz, 3H), 1.26 (t, *J* = 7.1 Hz, 3H). ^{13}C NMR (75 MHz, $CDCl_3$) δ (ppm) 173.1, 157.5, 135.6, 129.0 (2C), 127.3, 66.7, 60.5, 57.1, 49.9, 40.3, 31.5, 30.4, 18.3, 14.2. HRMS (ESI) Calcd. For $C_{17}H_{23}NO_4Na$: 328.1519 $[M+Na]^+$, Found: 328.1518.

Benzyl 4-((*S*)-4-isopropyl-2-oxooxazolidin-3-yl)pentanoate (64)

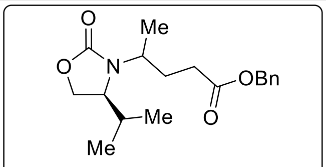

Chemical Formula: $C_{18}H_{25}NO_4$
Molecular Weight: 319,40

Following general procedure B, the reaction was carried out with olefin **61c** (169 mg, 1.00 mmol, 2.0 equiv), xanthate **2'b** (135 mg, 0.50 mmol, 1.0 equiv), 4-*tert*-butylcatechol (249 mg, 1.50 mmol), Et_3B (1.5 mL, 1.50 mmol) in DCM (5.0 mL) and it took 2 h for the reaction to go to completion. Flash chromatography on silica gel (gradient of ethyl acetate/heptane = 3:7 to 1:2) gave the desired product **64** as a light yellow oil (117 mg, 0.37 mmol, 73% yield, dr = 56:44). ^{1}H NMR (300 MHz, $CDCl_3$) δ (ppm) 7.38 (d, *J* = 4.0 Hz, 10H), 5.14 (s, 4H), 4.21 – 4.01 (m, 4H), 3.72 – 3.61 (m,3H), 3.50 (dp, *J* = 8.7, 6.8 Hz, 1H), 2.53 – 2.33 (m, 4H), 2.28 – 2.09 (m, 2H), 2.09 – 1.84 (m, 4H), 1.37 (d, *J* = 6.9 Hz, 3H), 1.29 (d, *J* = 6.8 Hz, 3H), 0.92 (d, *J* = 6.8 Hz, 3H), 0.91 (d, *J* = 6.8 Hz, 3H), 0.88 (d, *J* = 7.0 Hz, 3H), 0.86 (d, *J* = 7.0 Hz, 3H). ^{13}C NMR (75 MHz, $CDCl_3$) δ (ppm) 173.0, 172.9, 158.2, 157.6, 136.0, 135.9, 128.7, 128.43, 128.42, 66.55, 66.46, 62.7, 62.6, 61.2, 59.3, 50.0, 49.9, 31.6, 31.5, 29.8, 29.3, 28.6, 18.8, 18.4, 18.2, 18.1, 14.2, 14.1. HRMS (ESI) Calcd for $C_{18}H_{26}O_4N$: 320.1856 $[M+H]^+$, Found: 320.1845.

Benzyl 3-(((S)-4-isopropyl-2-oxooxazolidin-3-yl)methyl)pentanoate (65)

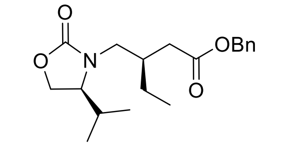

Chemical Formula: $C_{19}H_{27}NO_4$
Molecular Weight: 333,43

Following general procedure B, the reaction was carried out with olefin **61d**[19] (183 mg, 1.00 mmol, 2.0 equiv), xanthate **2'b** (135 mg, 0.50 mmol, 1.0 equiv), 4-*tert*-butylcatechol (249 mg, 1.50 mmol), Et_3B (1.5 mL, 1.50 mmol) in DCM (5.0 mL) and it took 1

[19] Guin, J.; Fröhlich, R.; Studer, A. *Angew. Chem. Int. Ed.* **2008**, *47*, 779.

h for the reaction to go to completion. Flash chromatography on silica gel (gradient of diethyl ether/pentane = 2:3 to 1:1) gave the desired product **65** as a colorless oil containing the other diastereoisomer in a 96:4 ratio (diastereomeric ratio was determined by GC analysis of the crude reaction mixture) (97 mg, 0.29 mmol, 58% yield of combined diastereoisomers). ^{1}H NMR (300 MHz, $CDCl_3$) δ (ppm) 7.40 – 7.28 (m, 5H), 5.14 (d, J = 12.2 Hz, 1H), 5.06 (d, J = 12.3 Hz, 1H), 4.16 – 3.99 (m, 2H), 3.76 (ddd, J = 8.6, 4.8, 3.4 Hz, 1H), 3.42 (dd, J = 14.3, 10.4 Hz, 1H), 2.96 (dd, J = 14.3, 4.5 Hz, 1H), 2.45 – 2.27 (m, 2H), 2.22 – 2.10 (m, 1H), 2.04 (ddq, J = 10.3, 6.9, 3.6 Hz, 1H), 1.50 – 1.28 (m, 2H), 0.93 (t, J = 7.5 Hz, 3H), 0.88 (d, J = 7.0 Hz, 3H), 0.83 (d, J = 6.9 Hz, 3H). ^{13}C NMR (75 MHz, $CDCl_3$) δ (ppm) 173.2, 158.8, 135.9, 128.7, 128.40, 128.38, 66.5, 62.7, 58.7, 45.5, 36.9, 34.8, 27.1, 25.5, 17.8, 14.2, 11.1. IR (neat, cm^{-1}): 2960, 2931, 2876, 1734, 1422, 1384, 1242, 1192, 1157, 1117, 1049, 1005, 975, 739, 697. HRMS (ESI) Calcd for $C_{19}H_{28}O_4N$: 334.2013 [M+H]$^+$, Found: 334.2005. $[\alpha]_D$ = 11.4 (c = 0.212, DCM).

Chromatogram of compound **65** and other diastereoisomer:

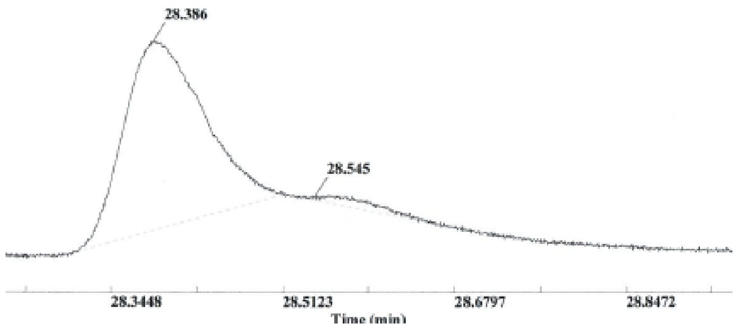

Peak Number #	Ret. Time	Area %
1	28.39	96.3991
2	28.54	3.6009

Benzyl 3-(((*S*)-2-oxo-4-phenyloxazolidin-3-yl)methyl)pentanoate (66)

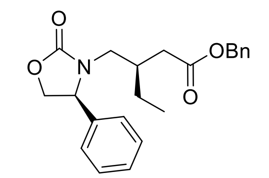

Chemical Formula: $C_{22}H_{25}NO_4$
Molecular Weight: 367,4450

Following general procedure B, the reaction was carried out with olefin **61e** (217 mg, 1.00 mmol, 2.0 equiv), xanthate **2’b** (135 mg, 0.50 mmol, 1.0 equiv), 4-*tert*-butylcatechol (249 mg, 1.50 mmol), Et_3B (1.5 mL, 1.50 mmol) in DCM (5.0 mL) and it took 3 h for the reaction to go to completion (diastereomeric ratio of 89:11 was determined by ^{1}H NMR analysis of the crude reaction mixture). Flash chromatography on silica

gel (gradient of diethyl ether/pentane = 2:3 to 1:1) gave the major diastereoisomer as a colorless oil (103 mg, 0.28 mmol, 56% yield) and the minor diastereoisomer as a colorless oil (12 mg, 0.03 mmol, 7% yield). *Major diastereoisomer:* ^{1}H NMR (300 MHz, $CDCl_3$) δ (ppm) 7.45 – 7.29 (m, 8H), 7.28 – 7.21 (m, 2H), 5.15 (d, J = 12.3 Hz, 1H), 5.08 (d, J = 12.2 Hz, 1H), 4.80 (dd, J = 8.9, 5.4 Hz, 1H), 4.51 (t, J = 8.8 Hz, 1H), 4.12 (dd, J = 8.7, 5.4 Hz, 1H), 3.35 (dd, J = 14.2, 10.5 Hz, 1H), 2.69 (dd, J = 14.2, 4.3 Hz, 1H), 2.48 – 2.23 (m, 2H), 2.17 – 1.96 (m, 1H), 1.24 (p, J = 7.3 Hz, 2H), 0.74 (t, J = 7.4 Hz, 3H). ^{13}C NMR (75 MHz, $CDCl_3$) δ (ppm) 173.3, 158.6, 138.2, 136.0, 129.5, 129.2, 128.7, 128.4, 127.2, 69.9, 66.6, 59.4, 46.0, 37.1, 34.5, 25.4, 11.0. IR (neat, cm^{-1}): 2962, 2926, 1743, 1457, 1415, 1383, 1235, 1184, 1154, 755, 697. HRMS (ESI) Calcd for $C_{22}H_{26}O_4N$: 368.1856 $[M+H]^+$, Found: 368.1846. $[\alpha]_D$ = 32.0 (c = 0.212, $CHCl_3$). *Minor diastereoisomer:* ^{1}H NMR (300 MHz, $CDCl_3$) δ (ppm) 7.44 – 7.30 (m, 6H), 7.30 – 7.23 (m, 4H), 5.03 (d, J = 12.3 Hz, 1H), 4.97 (d, J = 12.3 Hz, 1H), 4.75 (dd, J = 8.8, 5.4 Hz, 1H), 4.60 (t, J = 8.7 Hz, 1H), 4.16 (dd, J = 8.7, 5.4 Hz, 1H), 3.37 (dd, J = 14.2, 8.2 Hz, 1H), 2.70 (dd, J = 14.2, 6.0 Hz, 1H), 2.35 (dd, J = 15.5, 6.5 Hz, 1H), 2.22 (dd, J = 15.5, 6.8 Hz, 1H), 2.06 (dq, J = 8.4, 6.5 Hz, 1H), 1.50 – 1.33 (m, 1H), 1.27 (dq, J = 14.3, 7.2 Hz, 1H), 0.86 (t, J = 7.4 Hz, 3H). ^{13}C NMR (75 MHz, $CDCl_3$) δ (ppm) 172.4, 158.7, 138.1, 135.9, 129.4, 129.2, 128.7, 128.4, 128.3, 127.2, 69.9, 66.4, 60.5, 45.6, 36.4, 35.2, 24.4, 10.6. HRMS (ESI) Calcd for $C_{22}H_{26}O_4N$: 368.1856 $[M+H]^+$, Found: 368.1848.

Diethyl 2-(1-((*S*)-4-isopropyl-2-oxooxazolidin-3-yl)butan-2-yl)malonate (67)

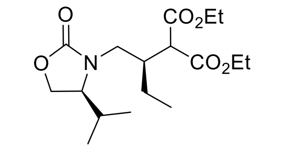

Chemical Formula: $C_{17}H_{29}NO_6$
Molecular Weight: 343,42

Under nitrogen, to a solution of olefin **61d** (92 mg, 0.50 mmol, 1.0 equiv) and xanthate **2'g** (280 mg, 1.00 mmol, 2.0 equiv) in dry DCM (5 mL) was added 4-*tert*-butylcatechol (249 mg, 1.50 mmol, 3.0 equiv), followed by slow addition of Et_3B (1.5 mL, 1 M in n-hexane) while the needle was immersed in the solution. The resulting reaction mixture was stirred open to air for 3 h at rt with a $CaCl_2$ trap. The solution was filtered over a small pad of neutral alox (eluted with diethyl ether). The filtrate was then concentrated under reduced pressure and purification by flash chromatography on silica gel (gradient of diethyl ether/pentane = 2:3 to 1:1) gave the desired product **67** as a colorless oil containing the other diastereoisomer in a 99:1 ratio (diastereomeric ratio was determined by GC analysis of the crude reaction mixture) (104 mg, 0.30 mmol, 61% yield of combined diastereoisomers). ^{1}H NMR (300 MHz, $CDCl_3$) δ (ppm) 4.27 – 4.02 (m, 6H), 3.82 – 3.72 (m, 1H), 3.58 (dd, J = 14.6, 9.1 Hz, 1H), 3.46 (d, J = 7.6 Hz, 1H), 3.09 (dd, J = 14.6, 5.1 Hz, 1H), 2.47 – 2.31 (m, 1H), 2.08 (ddq, J = 10.3, 6.9, 3.5 Hz, 1H), 1.48 (pd, J = 7.5, 1.8 Hz, 2H), 1.27 (t, J = 7.1 Hz, 6H), 0.96 (t, J = 7.5 Hz, 3H), 0.90 (d, J = 7.0 Hz, 3H), 0.84 (d, J = 6.8 Hz, 3H). ^{13}C NMR (75

MHz, $CDCl_3$) δ (ppm) 169.0, 168.7, 158.6, 62.7, 61.74, 61.67, 59.3, 54.0, 43.9, 37.9, 27.3, 23.3, 17.9, 14.24, 14.18, 14.1, 11.4. IR (neat, cm^{-1}): 2965, 2936, 2877, 1744, 1727, 1464, 1422, 1369, 1242, 1173, 1158, 1117, 1095, 1048, 1031, 977, 852, 771, 701. HRMS (ESI) Calcd for $C_{17}H_{30}O_6N$: 344.2068 $[M+H]^+$, Found: 344.2056. $[\alpha]_D$ = 23.7 (c = 0.214, DCM).

Chromatogram of compound **67** and other diastereoisomer:

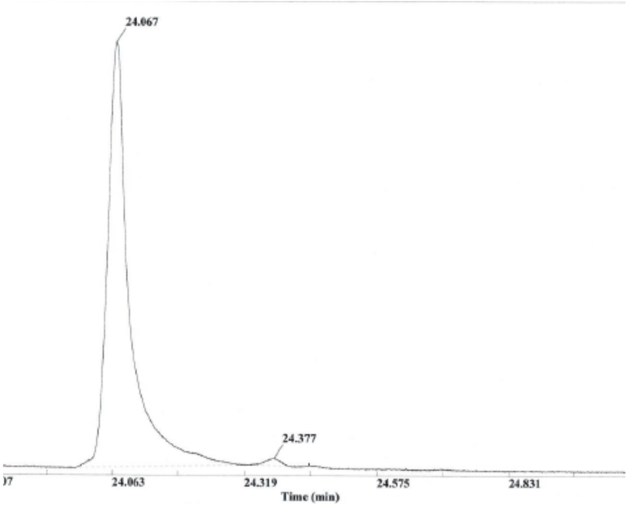

Peak Number #	Ret. Time	Area %
1	24.07	98.8264
2	24.38	1.1736

Diethyl 2-(1-((*S*)-2-oxo-4-phenyloxazolidin-3-yl)butan-2-yl)malonate (68)

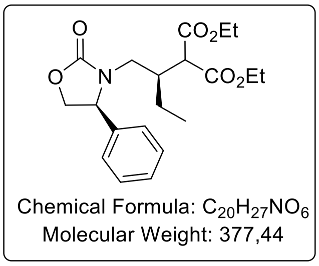

Following general procedure B, the reaction was carried out with olefin **61e** (217 mg, 1.00 mmol, 2.0 equiv), xanthate **2'g** (140 mg, 0.50 mmol, 1.0 equiv), 4-*tert*-butylcatechol (249 mg, 1.50 mmol), Et_3B (1.5 mL, 1.50 mmol) in DCM (5.0 mL) and it took 2 h for the reaction to go to completion. Flash chromatography on silica gel (gradient of diethyl ether/pentane = 1:2 to 1:1) gave the desired product **68** as a colorless oil containing the other diastereoisomer in a 92:8 ratio (diastereomeric ratio was determined by ^1H NMR of the crude reaction mixture) (129 mg, 0.34 mmol, 68% yield of combined diastereoisomers). ^1H NMR (300 MHz, $CDCl_3$) δ (ppm) 7.45 – 7.32 (m, 3H), 7.32 – 7.24 (m, 2H), 4.82 (dd, *J* = 8.8, 4.9 Hz, 1H), 4.58 (t, *J* = 8.7 Hz, 1H), 4.28 – 4.04 (m, 5H), 3.54 (dd, *J* = 14.5, 9.6 Hz, 1H), 3.43 (d, *J* = 7.8 Hz, 1H), 2.80 (dd, *J* = 14.5, 4.5 Hz, 1H), 2.33 – 2.21 (m, 1H), 1.41 – 1.13 (m, 8H), 0.68 (t, *J* = 7.4 Hz, 3H). ^{13}C NMR (75 MHz, $CDCl_3$) δ (ppm) 169.1, 168.6, 158.4, 138.3,

129.5, 129.3, 127.2, 70.0, 61.8, 61.6, 59.5, 54.3, 44.3, 37.4, 23.1, 14.2, 14.1, 11.1. IR (neat, cm^{-1}): 2968, 2935, 2877, 1746, 1725, 1459, 1415, 1368, 1224, 1172, 1155, 1116, 1092, 1065, 1028, 858, 760, 701. HRMS (ESI) Calcd for $C_{20}H_{28}O_6N$: 378.1911 $[M+H]^+$, Found: 378.1902. $[\alpha]_D$ = 33.6 (c = 0.200, $CHCl_3$).

Benzyl 3-(((*S*)-4-isopropyl-2-oxooxazolidin-3-yl)methyl)-4-methylpentanoate (69)

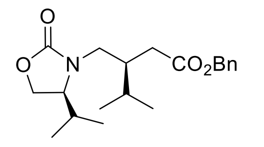

Chemical Formula: $C_{20}H_{29}NO_4$
Molecular Weight: 347,46

Following general procedure B, the reaction was carried out with olefin **61f**[19] (197 mg, 1.00 mmol, 2.0 equiv), xanthate **2'b** (135 mg, 0.50 mmol, 1.0 equiv), 4-*tert*-butylcatechol (249 mg, 1.50 mmol), Et_3B (1.5 mL, 1.50 mmol) in DCM (5.0 mL) and it took 3 h for the reaction to go to completion. Flash chromatography on silica gel (gradient of diethyl ether/pentane = 2:3 to 1:1) gave the desired product **69** as a colorless oil containing the other diastereoisomer in a 94:6 ratio (diastereomeric ratio was determined by GC analysis of the crude reaction mixture) (60 mg, 0.17 mmol, 35% yield of combined diastereoisomers). ^1H NMR (300 MHz, $CDCl_3$) δ (ppm) 7.41 – 7.28 (m, 5H), 5.13 (d, J = 12.2 Hz, 1H), 5.04 (d, J = 12.2 Hz, 1H), 4.10 – 3.99 (m, 2H), 3.78 (ddd, J = 8.5, 5.0, 3.4 Hz, 1H), 3.48 (dd, J = 14.2, 11.3 Hz, 1H), 2.94 (dd, J = 14.2, 3.8 Hz, 1H), 2.43 – 2.25 (m, 2H), 2.22 – 2.10 (m, 1H), 2.04 (ddp, J = 10.3, 6.9, 3.4 Hz, 1H), 1.75 (pd, J = 6.9, 4.0 Hz, 1H), 0.93 (d, J = 2.1 Hz, 3H), 0.90 (d, J = 2.1 Hz, 3H), 0.88 (d, J = 7.0 Hz, 3H), 0.83 (d, J = 6.9 Hz, 3H). ^{13}C NMR (75 MHz, $CDCl_3$) δ (ppm) 173.7, 158.8, 135.9, 128.7, 128.42, 128.39, 66.6, 62.7, 58.2, 43.8, 38.3, 34.5, 30.0, 27.1, 19.3, 19.0, 17.8, 14.2. IR (neat, cm^{-1}): 2959, 2875, 1739, 1425, 1389, 1370, 1333, 1245, 1156, 1111, 1049, 976, 739, 696. HRMS (ESI) Calcd for $C_{20}H_{30}O_4N$: 348.2169 $[M+H]^+$, Found: 348.2162. $[\alpha]_D$ = 6.8 (c = 0.096, DCM).

Chromatogram of compound **69** and other diastereoisomer:

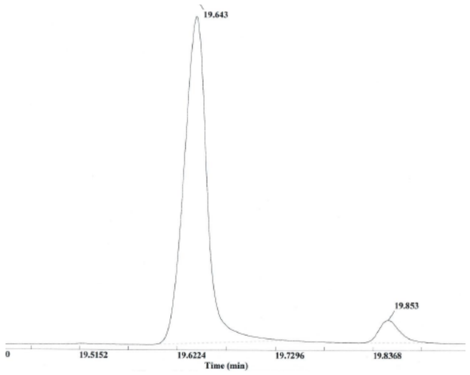

Peak Number #	Ret. Time	Area %

1	19.64	94.1017
2	19.85	5.8983

Ethyl (*R*)-3-(((*4S*,*5R*)-2-oxo-4,5-diphenyloxazolidin-3-yl)methyl)pentanoate (70)

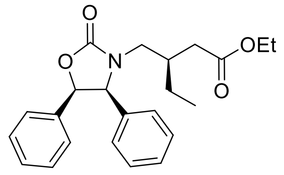

Chemical Formula: $C_{23}H_{27}NO_4$
Molecular Weight: 381,47

Under nitrogen, to a solution of olefin **61g** (147 mg, 0.50 mmol, 1.0 equiv) and xanthate **2'a** (208 mg, 1.00 mmol, 2.0 equiv) in dry DCM (5 mL) was added 4-*tert*-butylcatechol (249 mg, 1.50 mmol, 3.0 equiv), followed by slow addition of Et_3B (1.5 mL, 1 M in *n*-hexane) while the needle was immersed in the solution. The resulting reaction mixture was stirred open to air for 3 h at rt with a $CaCl_2$ trap. The solution was filtered over a small pad of neutral alox (eluted with ethyl acetate). The filtrate was then concentrated under reduced pressure and purification by flash chromatography on silica gel (gradient of diethyl ether/pentane = 1:3 to 3:7) gave the desired product **70** as a white solid containing the other diastereoisomer in a 86:14 ratio (diastereomeric ratio was determined by ^{1}H NMR of the crude reaction mixture) (115 mg, 0.30 mmol, 60% yield of combined diastereoisomers). ^{1}H NMR (300 MHz, $CDCl_3$) δ (ppm) 7.13 – 6.98 (m, 6H), 7.02 – 6.95 (m, 2H), 6.90 – 6.77 (m, 2H), 5.79 (d, J = 8.1 Hz, 1H), 5.08 (d, J = 8.1 Hz, 1H), 4.18 (qd, J = 7.1, 4.0 Hz, 2H), 3.56 (dd, J = 14.2, 10.7 Hz, 1H), 2.73 (dd, J = 14.2, 4.0 Hz, 1H), 2.51 – 2.26 (m, 2H), 2.15 (dtd, J = 13.9, 6.4, 4.2 Hz, 1H), 1.36 – 1.20 (m, 2H), 1.29 (t, J = 7.1 Hz, 3H), 0.80 (t, J = 7.4 Hz, 3H). ^{13}C NMR (75 MHz, $CDCl_3$) δ (ppm) 173.8, 158.7, 134.7, 134.0, 128.5, 128.0, 127.9, 126.1, 79.8, 64.8, 60.8, 46.9, 37.4, 35.0, 25.5, 14.4, 11.0. IR (neat, cm^{-1}): 2961, 2929, 1747, 1733, 1456, 1413, 1374, 1330, 1293, 1229, 1189, 1162, 1035, 1024, 760, 699. HRMS (ESI) Calcd for $C_{23}H_{28}O_4N$: 382.2013 $[M+H]^+$, Found: 382.2019. $[\alpha]_D$ = -18.61 (c = 0.202, $CHCl_3$). mp: 44.7-45.4 °C.

Benzyl 3-(((4*S*,5*R*)-2-oxo-4,5-diphenyloxazolidin-3-yl)methyl)pentanoate (71)

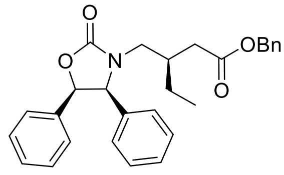

Chemical Formula: $C_{28}H_{29}NO_4$
Molecular Weight: 443,54

Under nitrogen, to a solution of olefin **61g** (293 mg, 1.00 mmol, 1.0 equiv) and xanthate **2'b** (541 mg, 2.00 mmol, 2.0 equiv) in dry DCM (10 mL) was added 4-*tert*-butylcatechol (499 mg, 3.00 mmol, 3.0 equiv), followed by slow addition of Et_3B (3.0 mL, 1 M in *n*-hexane) while the needle was immersed in the solution. The resulting reaction mixture was stirred open to air for 2 h at rt with a $CaCl_2$ trap. The solution was filtered over a small pad of neutral alox (eluted with ethyl acetate). The filtrate was then concentrated under reduced pressure and purification by flash chromatography on silica gel

(gradient of diethyl ether/pentane = 1:4 to 1:2) gave the desired product **71** as a white solid containing the other diastereoisomer in a 89:11 ratio (diastereomeric ratio was determined by ^{1}H NMR of the crude reaction mixture) (292 mg, 0.66 mmol, 66% yield of combined diastereoisomers). Suitable crystals were obtained upon slow diffusion of pentane in a solution of **71** in ethyl acetate at room temperature. ^{1}H NMR (300 MHz, $CDCl_3$) δ (ppm) 7.44 – 7.27 (m, 5H), 7.16 – 7.02 (m, 6H), 6.96 (dd, J = 6.8, 2.9 Hz, 2H), 6.83 (dd, J = 6.7, 2.9 Hz, 2H), 5.67 (d, J = 8.1 Hz, 1H), 5.20 (d, J = 12.3 Hz, 1H), 5.13 (d, J = 12.2 Hz, 1H), 5.04 (d, J = 8.1 Hz, 1H), 3.56 (dd, J = 14.2, 10.7 Hz, 1H), 2.73 (dd, J = 14.2, 4.1 Hz, 1H), 2.52 (dd, J = 16.2, 7.1 Hz, 1H), 2.41 (dd, J = 16.2, 5.8 Hz, 1H), 2.24 – 2.07 (m, 1H), 1.29 (dq, J = 8.4, 6.3 Hz, 2H), 0.79 (t, J = 7.4 Hz, 3H). ^{13}C NMR (75 MHz, $CDCl_3$) δ (ppm) 173.5, 158.8, 136.0, 134.7, 134.0, 128.8, 128.5, 128.45, 128.41, 128.0, 127.9, 126.1, 79.7, 66.7, 64.8, 46.9, 37.3, 35.1, 25.5, 11.0. IR (neat, cm^{-1}): 2960, 2907, 2872, 1726, 1543, 1454, 1423, 1343, 1277, 1240, 1193, 1166, 1116, 1066, 1037, 1026, 759, 731, 720, 698. HRMS (ESI) Calcd for $C_{28}H_{30}O_4N$: 444.2169 $[M+H]^+$, Found: 444.2149. $[\alpha]_D$ = -12.09 (c = 0.402, $CHCl_3$). mp: 68.6-72.0 °C.

X-Ray crystal structure report of **71** (CCDC number: 2031378):

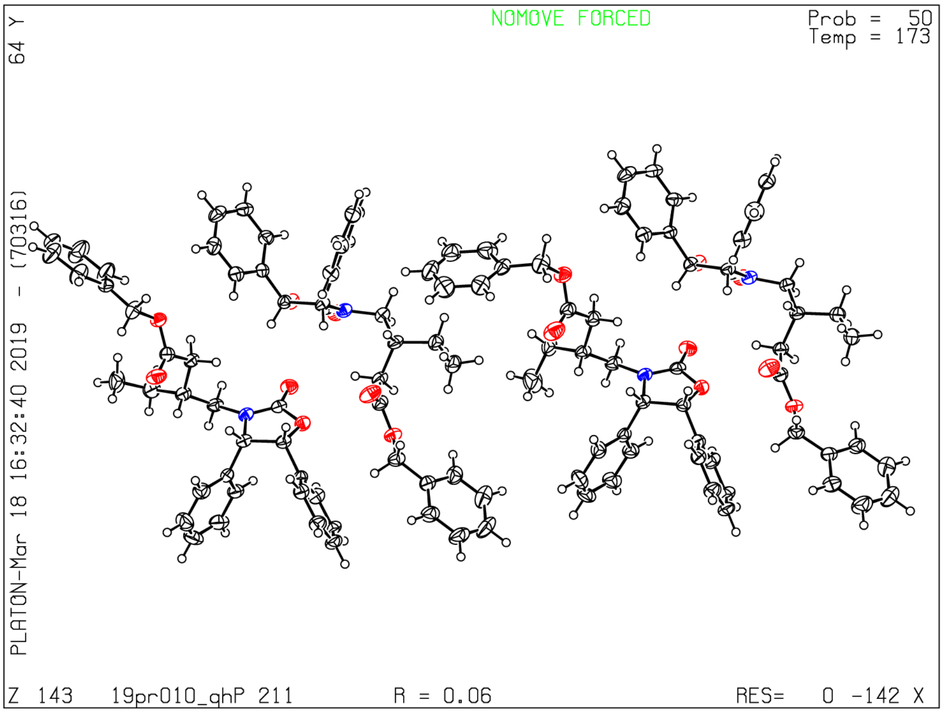

Table 2. Crystal data and structure refinement for **71**.

Empirical formula	$C_{112}H_{116}N_4O_{16}$
Formula weight	1774.08

Temperature	173(2) K	
Wavelength	1.54184 Å	
Crystal system	Monoclinic	
Space group	P 21	
Unit cell dimensions	a = 6.1281(2) Å	α= 90°.
	b = 27.1638(5) Å	β= 91.472(2)°.
	c = 28.2117(7) Å	γ = 90°.
Volume	4694.6(2) $Å^3$	
Z	2	
Density (calculated)	1.255 Mg/m^3	
Absorption coefficient	0.669 mm^{-1}	
F(000)	1888	
Crystal size	0.353 x 0.076 x 0.043 mm^3	
Theta range for data collection	2.258 to 77.191°.	
Index ranges	-7<=h<=7, -18<=k<=33, -34<=l<=35	
Reflections collected	36979	
Independent reflections	13824 [R(int) = 0.0716]	
Completeness to theta = 67.684°	99.9 %	
Absorption correction	Semi-empirical from equivalents	
Max. and min. transmission	1 and 0.32761	
Refinement method	Full-matrix least-squares on F^2	
Data / restraints / parameters	13824 / 1 / 1193	
Goodness-of-fit on F^2	1.037	
Final R indices [I>2sigma(I)]	R1 = 0.0553, wR2 = 0.1294	
R indices (all data)	R1 = 0.0692, wR2 = 0.1370	
Absolute structure parameter	-0.31(19)	
Extinction coefficient	n/a	
Largest diff. peak and hole	0.248 and -0.238 e.$Å^{-3}$	

4.3 Modification of Radical Adducts

(3*S*,8*S*,9*S*,10*R*,13*S*,14*S*,17*S*)-10,13-Dimethyl-17-((*R*)-5-oxotetrahydrofuran-2-yl)-2,3,4,7,8,9,10,11,12,13,14,15,16,17-tetradecahydro-1*H*-cyclopenta[*a*]phenanthren-3-yl acetate (42)

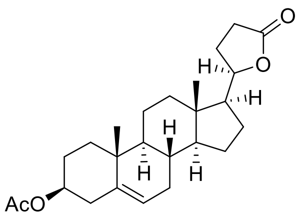

Chemical Formula: $C_{25}H_{36}O_4$
Molecular Weight: 400,56

To an oven-dried round bottom flask was added under nitrogen **39** (125 mg, 0.20 mmol, 1.0 equiv) and dry THF (10.0 mL). To the solution was added via syringe TBAF (1 M in THF, 1.0 mL, 1.00 mmol, 5.0 equiv). After stirring at rt for 24 h, the reaction was quenched with water, followed by extraction with ethyl acetate for 3 times. The combined organic phase was then washed with brine, dried over anhydrous Na_2SO_4, filtered and concentrated under reduced pressure. Purification by flash chromatography on silica gel (gradient of ethyl acetate:heptane = 1:4 to 1:3) gave the major diastereoisomer **42** as a white solid (62 mg, 0.15 mmol, 77%). Suitable crystals were obtained upon slow diffusion of heptane in a solution of **42** in Et_2O at room temperature. ^1H NMR (300 MHz, $CDCl_3$) δ (ppm) 5.36 (d, J = 5.1 Hz, 1H), 4.60 (tdd, J = 10.9, 6.6, 4.3 Hz, 1H), 4.41 (ddd, J = 10.3, 8.3, 6.2 Hz, 1H), 2.55 – 2.41 (m, 2H), 2.36 – 2.18 (m, 3H), 2.14 (dt, J = 12.8, 3.5 Hz, 1H), 2.03 (s, 3H), 2.00 – 1.91 (m, 1H), 1.91 – 1.80 (m, 2H), 1.79 – 1.67 (m, 2H), 1.66 – 1.34 (m, 7H), 1.33 – 0.90 (m, 6H), 1.03 (s, 3H), 0.77 (s, 3H). ^{13}C NMR (75 MHz, $CDCl_3$) δ (ppm) 177.7, 170.7, 140.0, 122.4, 82.5, 74.1, 56.1, 55.6, 50.2, 42.8, 38.8, 38.3, 37.1, 36.8, 32.1, 31.8, 28.6, 28.2, 27.9, 24.7, 23.9, 21.6, 20.7, 19.5, 12.5. IR (neat, cm^{-1}): 2964, 2937, 2904, 2884, 2868, 2820, 1761, 1729, 1248, 1177, 1038, 975. HRMS (ESI) Calcd for $C_{25}H_{37}O_4$: 401.2686 $[M+H]^+$, Found: 401.2683. mp: 211.4-212.6 °C. $[\alpha]_D$ = -81.0 (c = 0.204, $CHCl_3$).

X-Ray crystal structure report of **42** (CCDC number: 2031383):

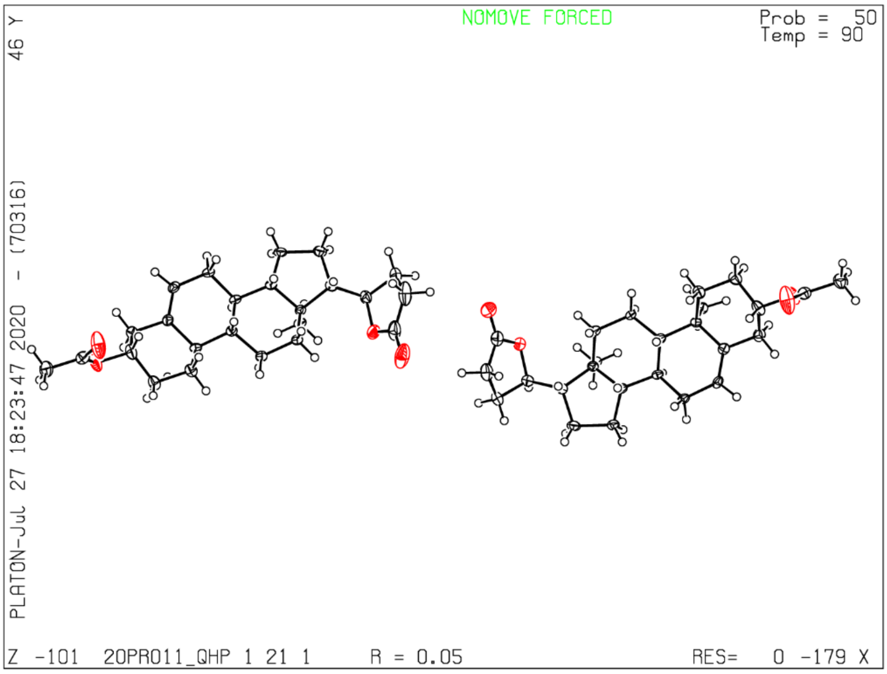

Table 3. Crystal data and structure refinement for **42**.

Empirical formula	$C_{25}H_{36}O_4$
Formula weight	400.54
Temperature/K	90.0(9)
Crystal system	monoclinic
Space group	$P2_1$
a/Å	6.15180(6)
b/Å	60.5855(6)
c/Å	6.15597(7)
α/°	90
β/°	106.5319(11)
γ/°	90
Volume/Å^3	2199.55(4)
Z	4
ρ_{calc}g/cm^3	1.210

μ/mm^{-1}	0.634
F(000)	872.0
Crystal size/mm^3	0.23 × 0.21 × 0.06
Radiation	CuKα (λ = 1.54184)
2Θ range for data collection/°	4.376 to 148.612
Index ranges	-7 ≤ h ≤ 7, -74 ≤ k ≤ 75, -7 ≤ l ≤ 7
Reflections collected	41608
Independent reflections	8784 [R_{int} = 0.0512, R_{sigma} = 0.0327]
Data/restraints/parameters	8784/1/530
Goodness-of-fit on F^2	1.035
Final R indexes [I>=2σ (I)]	R_1 = 0.0455, wR_2 = 0.1155
Final R indexes [all data]	R_1 = 0.0486, wR_2 = 0.1192
Largest diff. peak/hole / e Å^{-3}	0.26/-0.30
Flack parameter	-0.01(8)

Ethyl 2-((8*R*,9*S*,13*S*,14*S*,16*S*,17*S*)-17-hydroxy-3-methoxy-13-methyl-7,8,9,11,12,13,14,15,16,17-decahydro-6*H*-cyclopenta[*a*]phenanthren-16-yl)acetate (43)

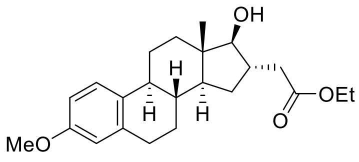

Chemical Formula: $C_{23}H_{32}O_4$
Molecular Weight: 372,51

To an oven-dried round bottom flask was added under nitrogen **41** (247 mg, 0.51 mmol, 1.0 equiv) and dry THF (10.0 mL). To the solution was added via syringe TBAF (1 M in THF, 2.54 mL, 2.54 mmol, 5.0 equiv). After stirring at rt for 7 h, the reaction was quenched with water, followed by extraction with diethyl ether for 3 times. The combined organic phase was then washed with brine, dried over anhydrous Na_2SO_4, filtered and concentrated under reduced pressure. Purification by flash chromatography on silica gel (ethyl acetate:heptane = 1:4) gave the desired product **43** as a white solid (167 mg, 0.45 mmol, 88%). ^{1}H NMR (300 MHz, $CDCl_3$) δ (ppm) 7.20 (dd, J = 8.7, 1.0 Hz, 1H), 6.71 (dd, J = 8.6, 2.9 Hz, 1H), 6.62 (d, J = 2.8 Hz, 1H), 4.16 (q, J = 7.2 Hz, 2H), 3.77 (s, 3H), 3.38 (d, J = 7.3 Hz, 1H), 2.85 (q, J = 5.4, 4.9 Hz, 2H), 2.54 (d, J = 7.7 Hz, 2H), 2.33 – 2.16 (m, 3H), 1.98 (dt, J = 12.5, 3.3 Hz, 1H), 1.89 – 1.67 (m, 2H), 1.58 – 1.21 (m, 7H), 1.27 (t, J = 7.2 Hz, 3H), 0.85 (s, 3H). ^{13}C NMR (75 MHz, $CDCl_3$) δ (ppm) 174.5, 157.6, 138.0, 132.7, 126.5, 114.0, 111.6, 87.7, 60.8, 55.3, 48.5, 44.6, 44.1, 40.5, 40.1, 38.6, 37.0, 30.9, 29.9, 27.4, 26.3, 14.4, 12.1.

IR (neat, cm^{-1}): 3454, 2926, 2868, 1733, 1714, 1608, 1498, 1254, 1236, 1034. HRMS (ESI) Calcd for $C_{23}H_{33}O_4$: 373.2373 $[M+H]^+$, Found: 373.2367. mp: 78.2-80.6 °C. $[\alpha]_D$ = +67.4 (c = 0.202, $CHCl_3$).

Ethyl (*R*)-3-(((tert-butoxycarbonyl)amino)methyl)pentanoate (72)

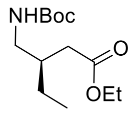

Chemical Formula: $C_{13}H_{25}NO_4$
Molecular Weight: 259,35

Pearlman's catalyst (20 wt% $Pd(OH)_2$ on carbon, 35 mg, 0.05 mmol, 50 mol%) was added to a solution of **70** (38 mg, 0.10 mmol, 1.0 equiv) and di-*tert*-butyldicarbonate (66 mg, 0.30 mmol, 3.0 equiv) in absolute ethanol (10.0 mL). The mixture was then purged with H_2 twice followed by pressurizing to 30 psi of H_2. The reaction was stirred at rt for 40 h, subsequently filtered through Celite and rinsed with ethyl acetate. The filtrate was evaporated under reduced pressure. Purification by flash chromatography on silica gel (ethyl acetate:heptane = 1:6) gave the desired product **72** as a colorless oil (22 mg, 0.085 mmol, 85%). ^1H NMR (300 MHz, $CDCl_3$) δ (ppm) 4.66 (br, 1H), 4.13 (q, J = 7.1 Hz, 2H), 3.19 (dt, J = 11.7, 5.6 Hz, 1H), 3.04 (dt, J = 13.7, 6.9 Hz, 1H), 2.28 (dd, J = 6.7, 2.0 Hz, 2H), 1.95 (hept, J = 6.7 Hz, 1H), 1.43 (s, 9H), 1.41 – 1.29 (m, 2H), 1.26 (t, J = 7.1 Hz, 3H), 0.92 (t, J = 7.4 Hz, 3H). ^{13}C NMR (101 MHz, $CDCl_3$) δ (ppm) 173.4, 156.2, 79.3, 60.5, 43.9, 37.6, 37.0, 28.5, 25.0, 14.4, 11.2. IR (neat, cm^{-1}): 3371, 2968, 2933, 2878, 1714, 1695, 1516, 1365, 1248, 1164, 1035. HRMS (ESI) Calcd for $C_{13}H_{26}O_4N$: 260.1856 $[M+H]^+$, Found: 260.1860. $[\alpha]_D$ = -1.8 (c = 0.250, $CHCl_3$).

5 Spectra

Cyclohex-2-enyl 2-(ethoxycarbonothioylthio) acetate (**2’i**) ^{1}H NMR (300 MHz, $CDCl_3$)

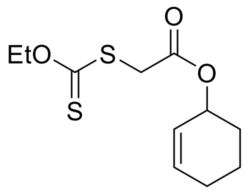

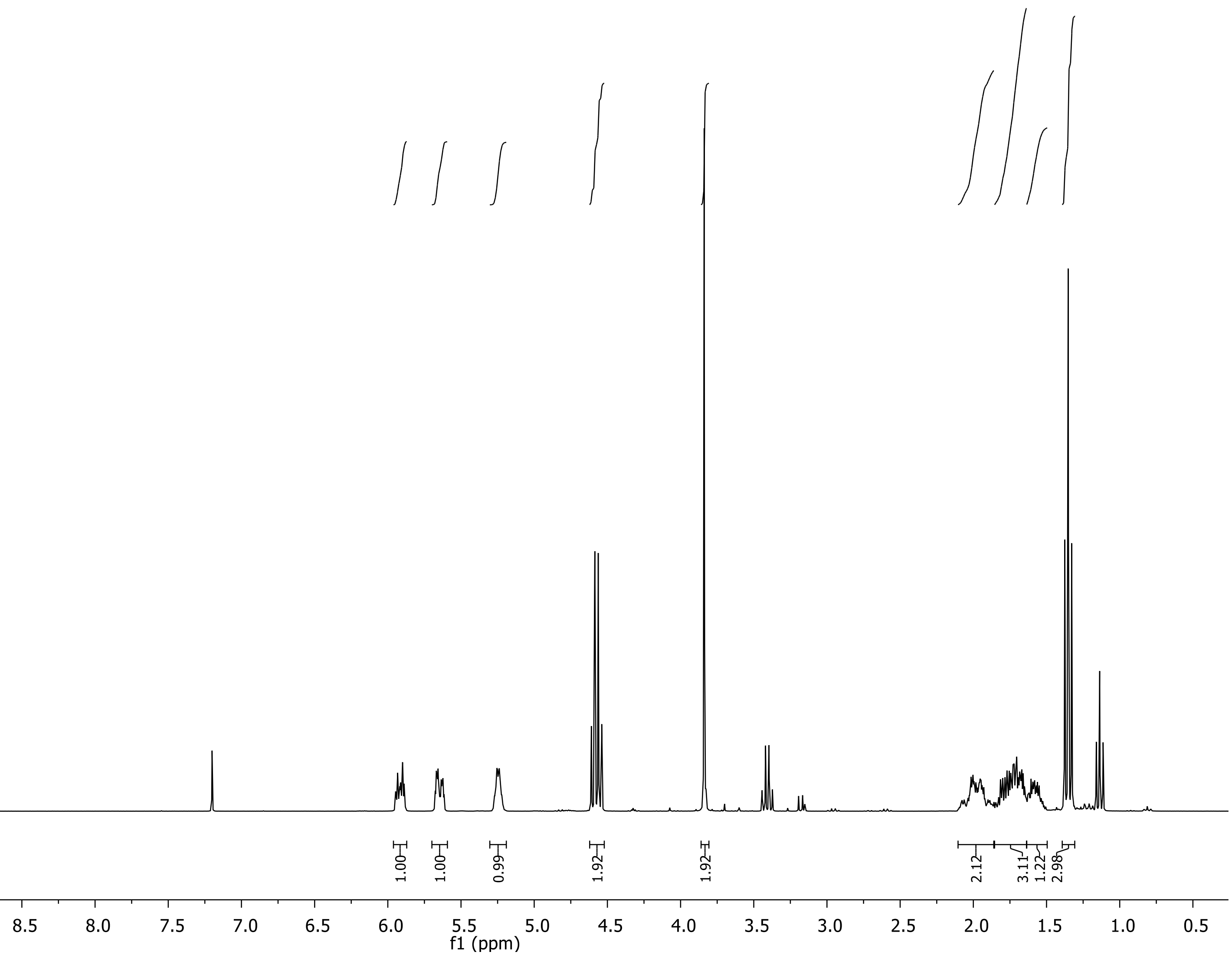

Cyclohex-2-enyl 2-(ethoxycarbonothioylthio) acetate (**2’i**)

^{13}C NMR (75 MHz, $CDCl_3$)

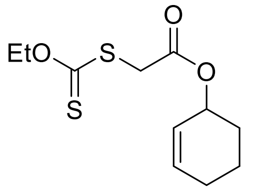

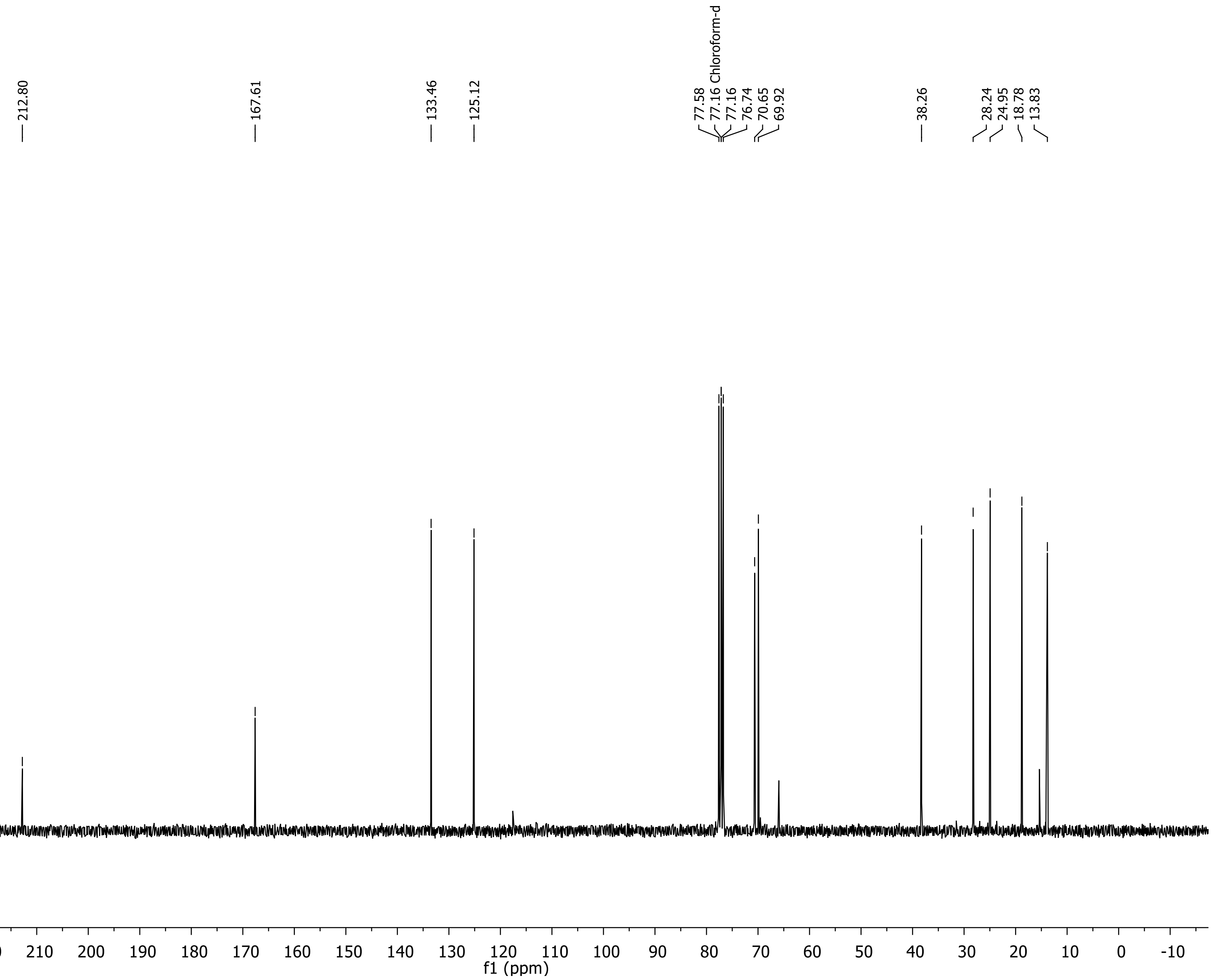

1-Acetyl-3-vinylimidazolidin-2-one (**44f**)

^{1}H NMR (300 MHz, CD_3CN)

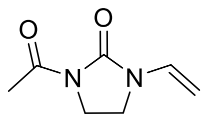

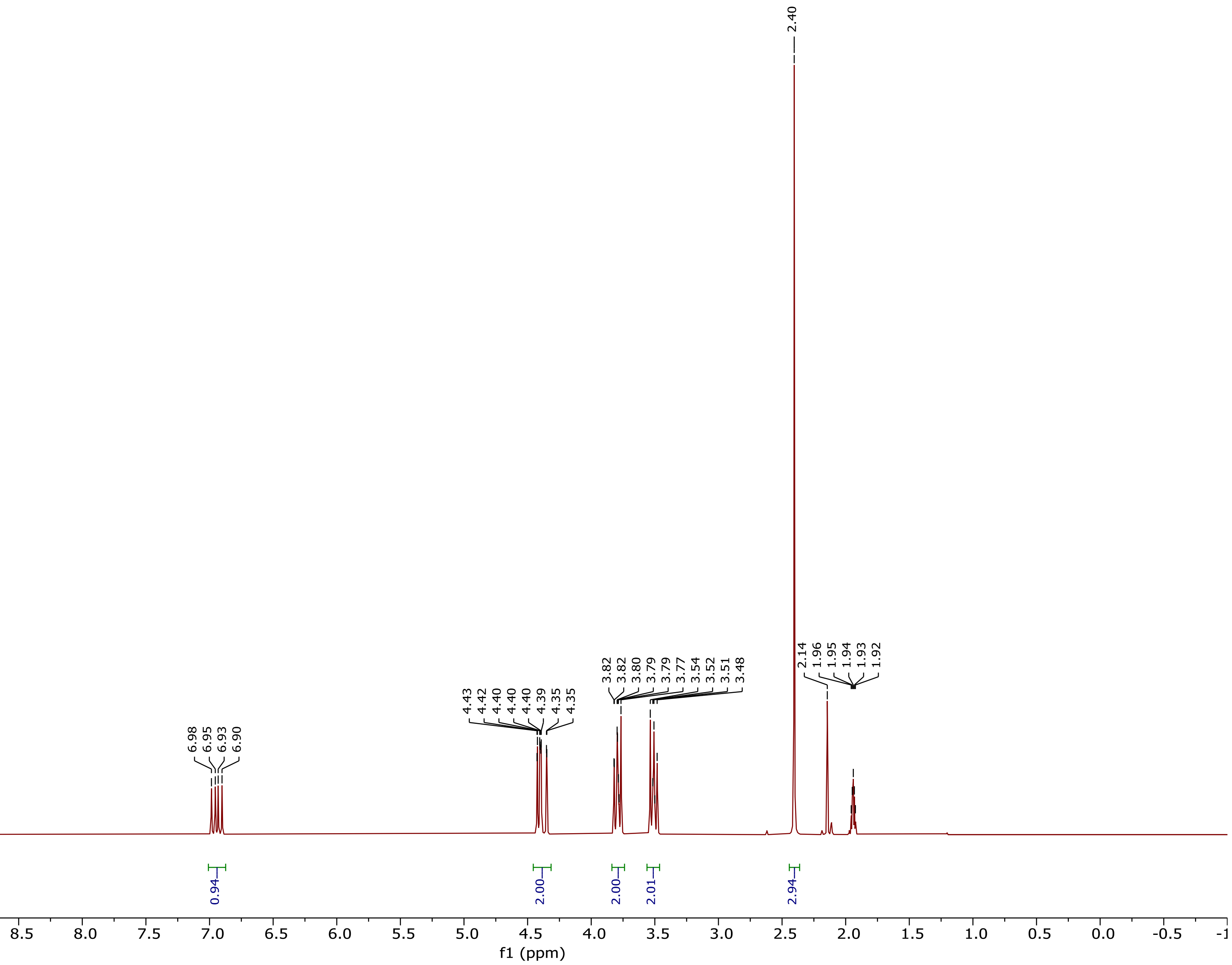

1-Acetyl-3-vinylimidazolidin-2-one (**44f**)

^{13}C NMR (75 MHz, CD_3CN)

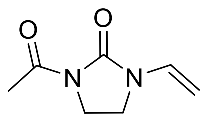

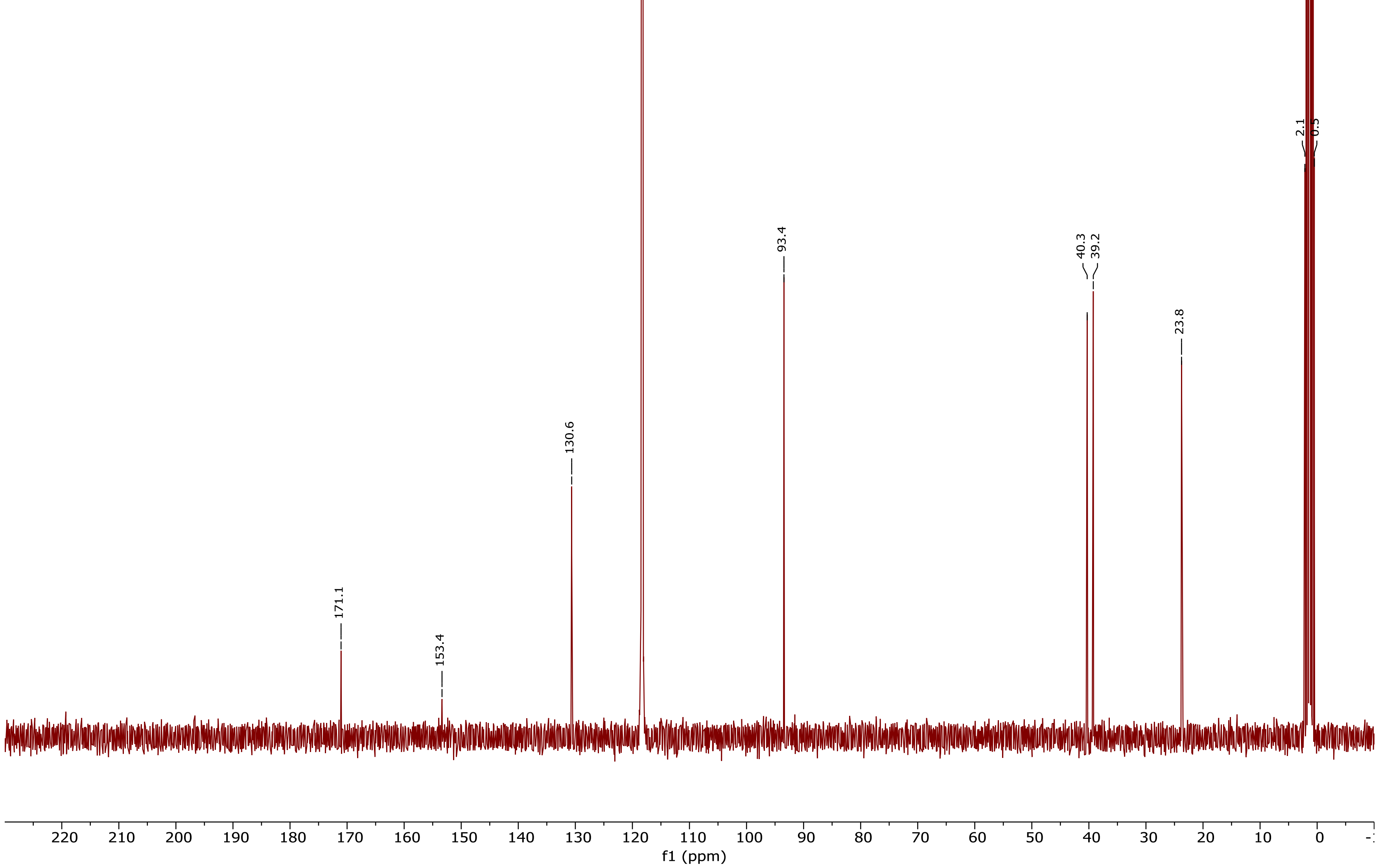

3-(Cyclohex-1-en-1-yl)oxazolidin-2-one (**44k**)

^{1}H NMR (300 MHz, CD_3CN)

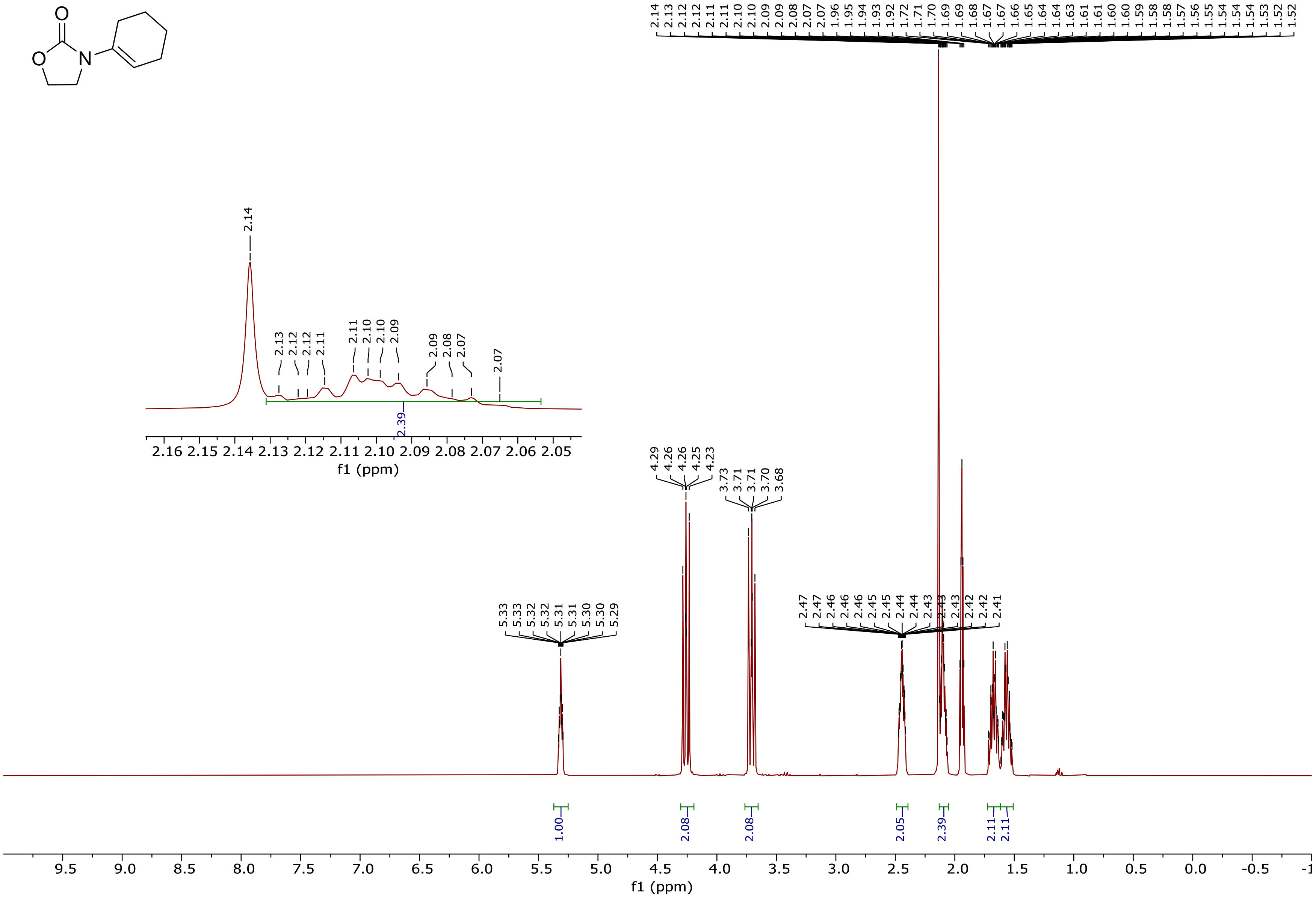

3-(Cyclohex-1-en-1-yl)oxazolidin-2-one (**44k**)

^{13}C NMR (75 MHz, CD_3CN)

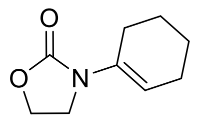

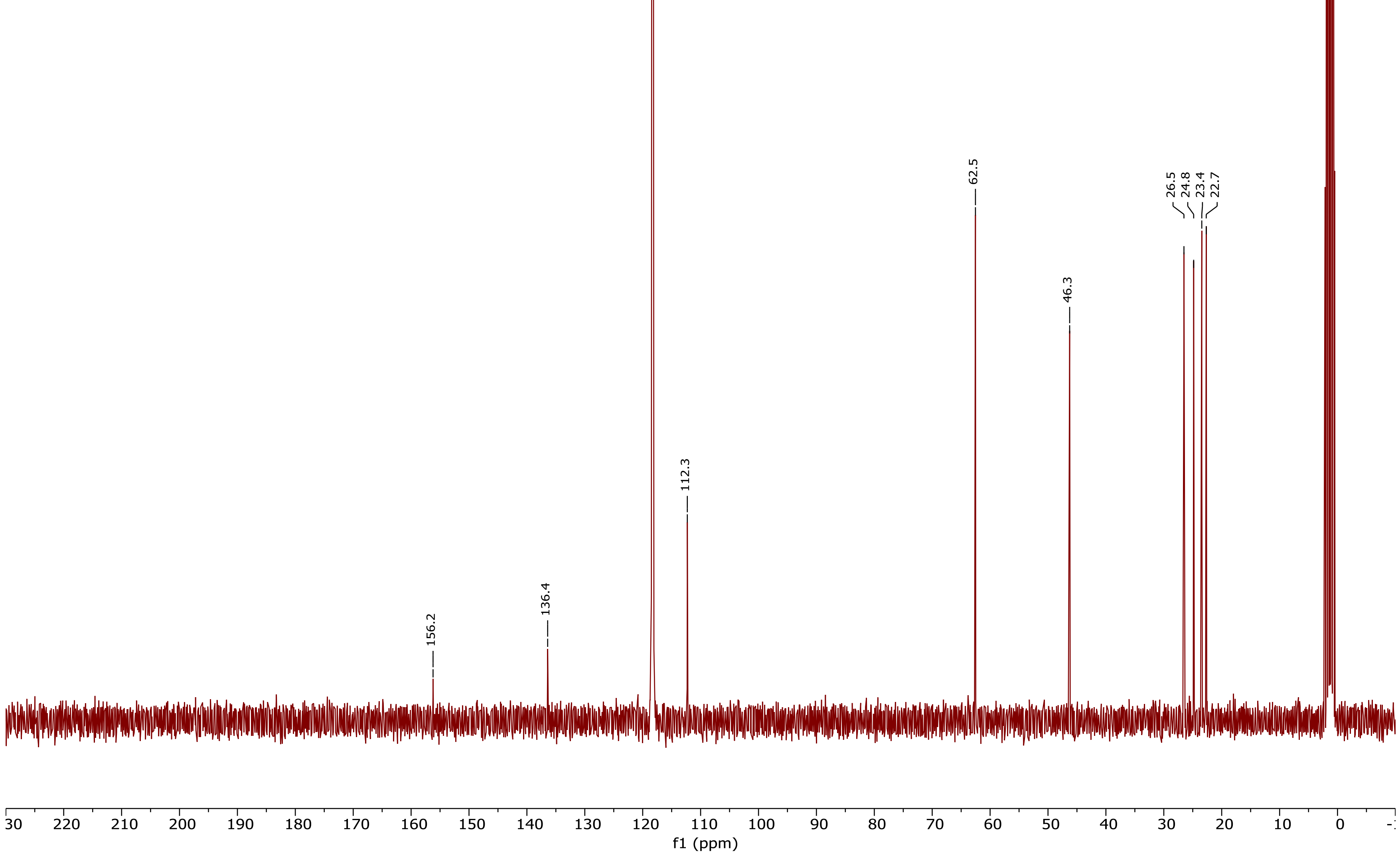

(*S*)-4-Phenyl-3-(prop-1-en-2-yl) oxazolidin-2-one (**61a**)

^{1}H NMR (300 MHz, $CDCl_3$)

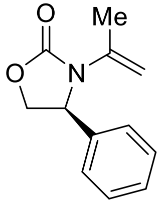

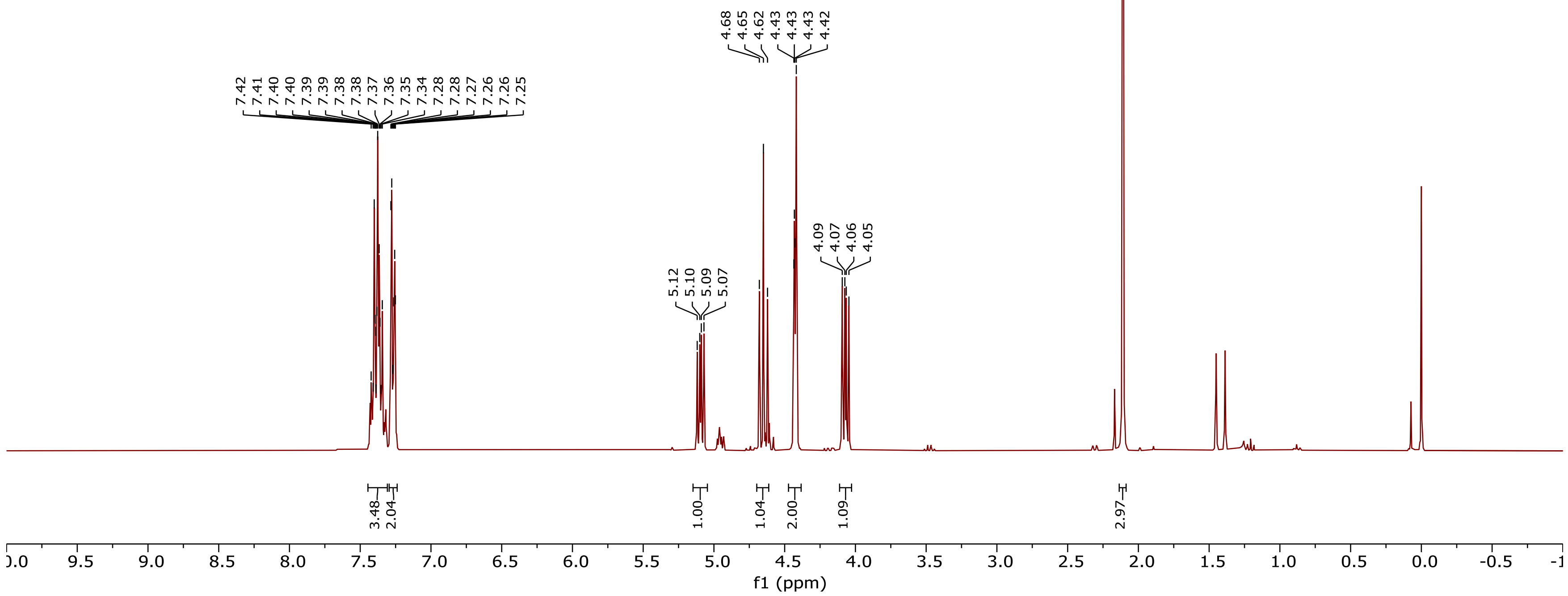

(*S*)-4-Phenyl-3-(prop-1-en-2-yl) oxazolidin-2-one (**61a**)

^{13}C NMR (75 MHz, $CDCl_3$)

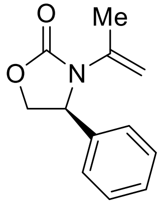

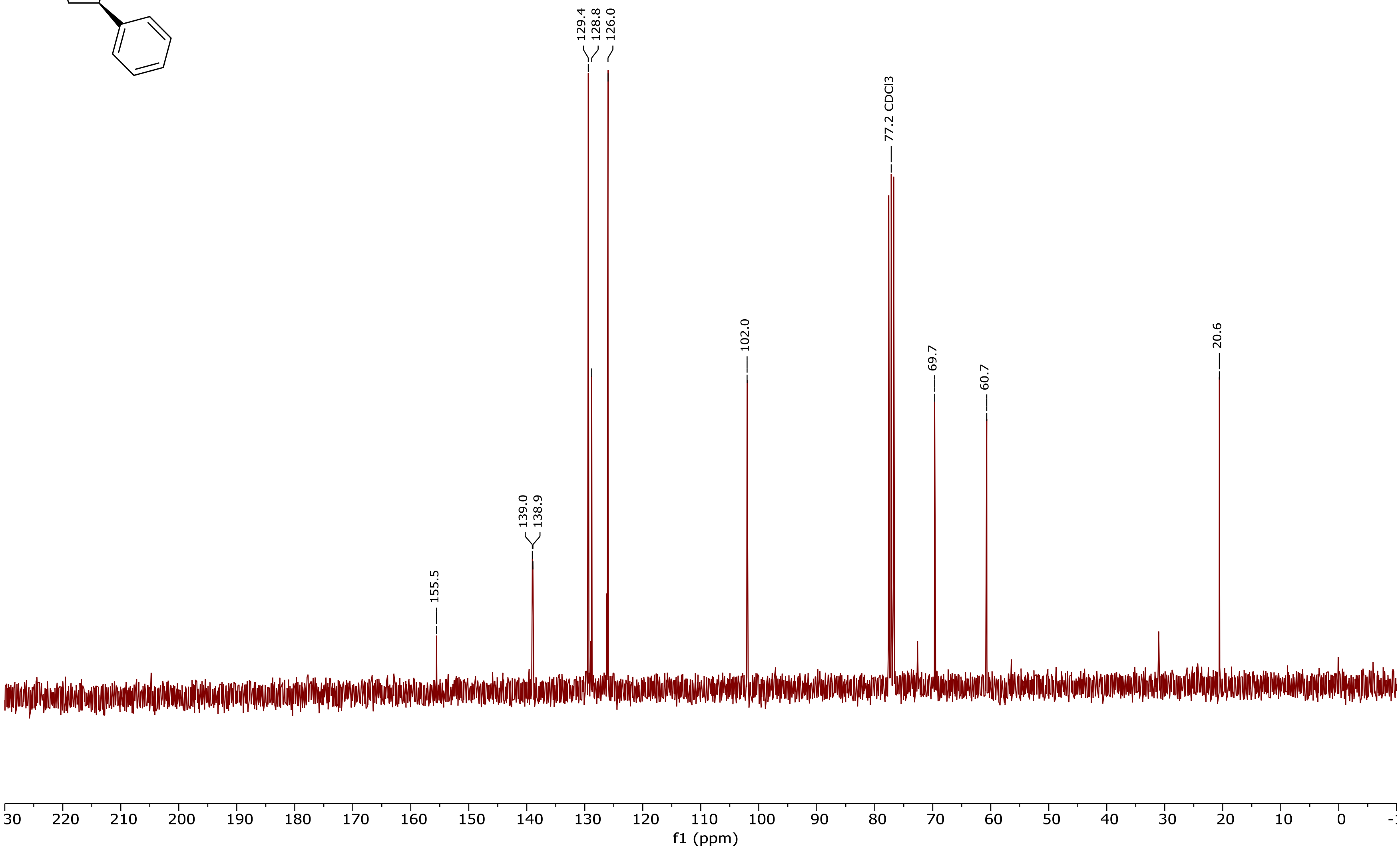

(*S*)-4-Benzyl-3-(prop-1-en-2-yl) oxazolidin-2-one (**61b**)

^{1}H NMR (300 MHz, $CDCl_3$)

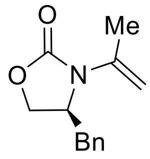

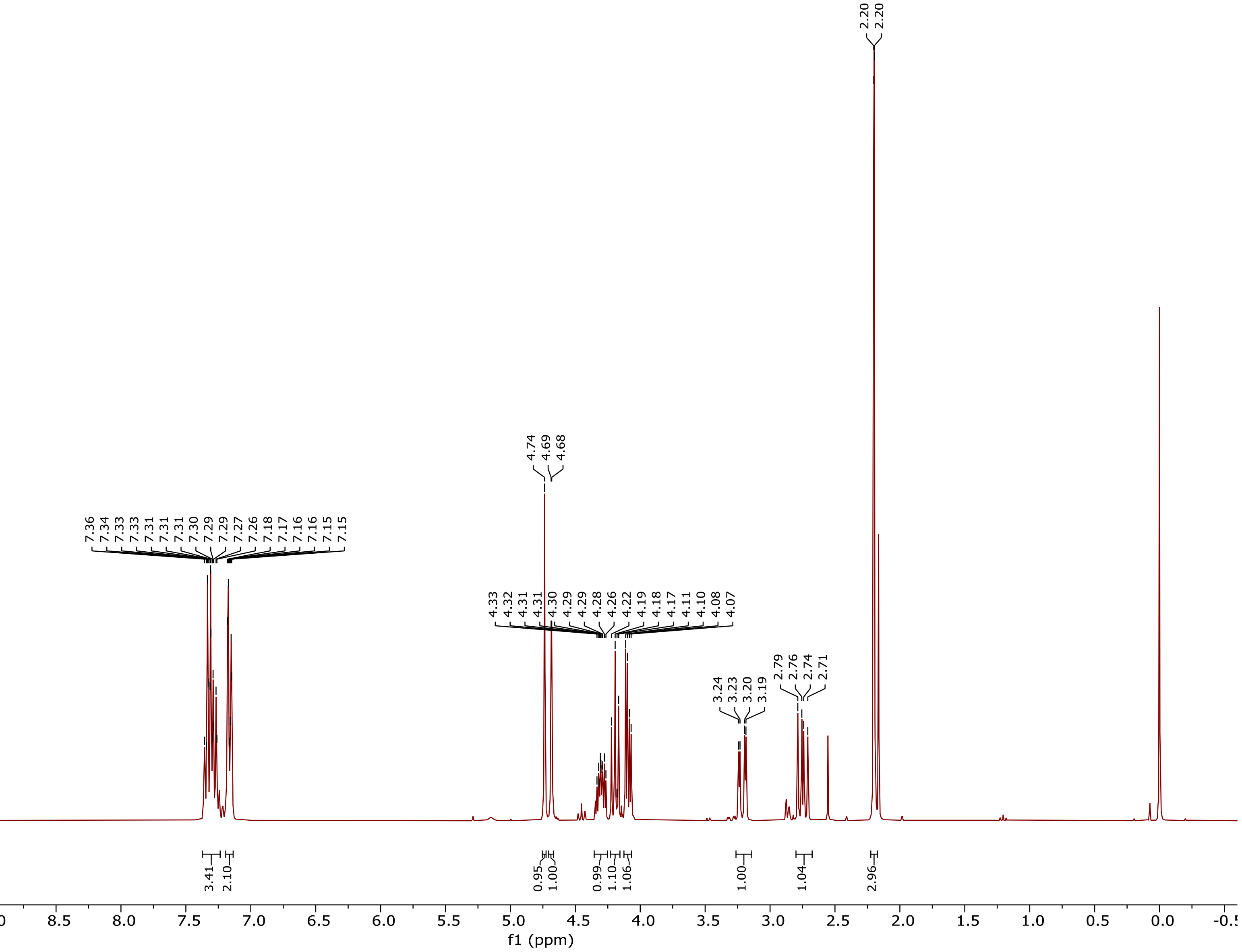

(*S*)-4-Benzyl-3-(prop-1-en-2-yl) oxazolidin-2-one (**61b**)

^{13}C NMR (75 MHz, $CDCl_3$)

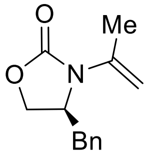

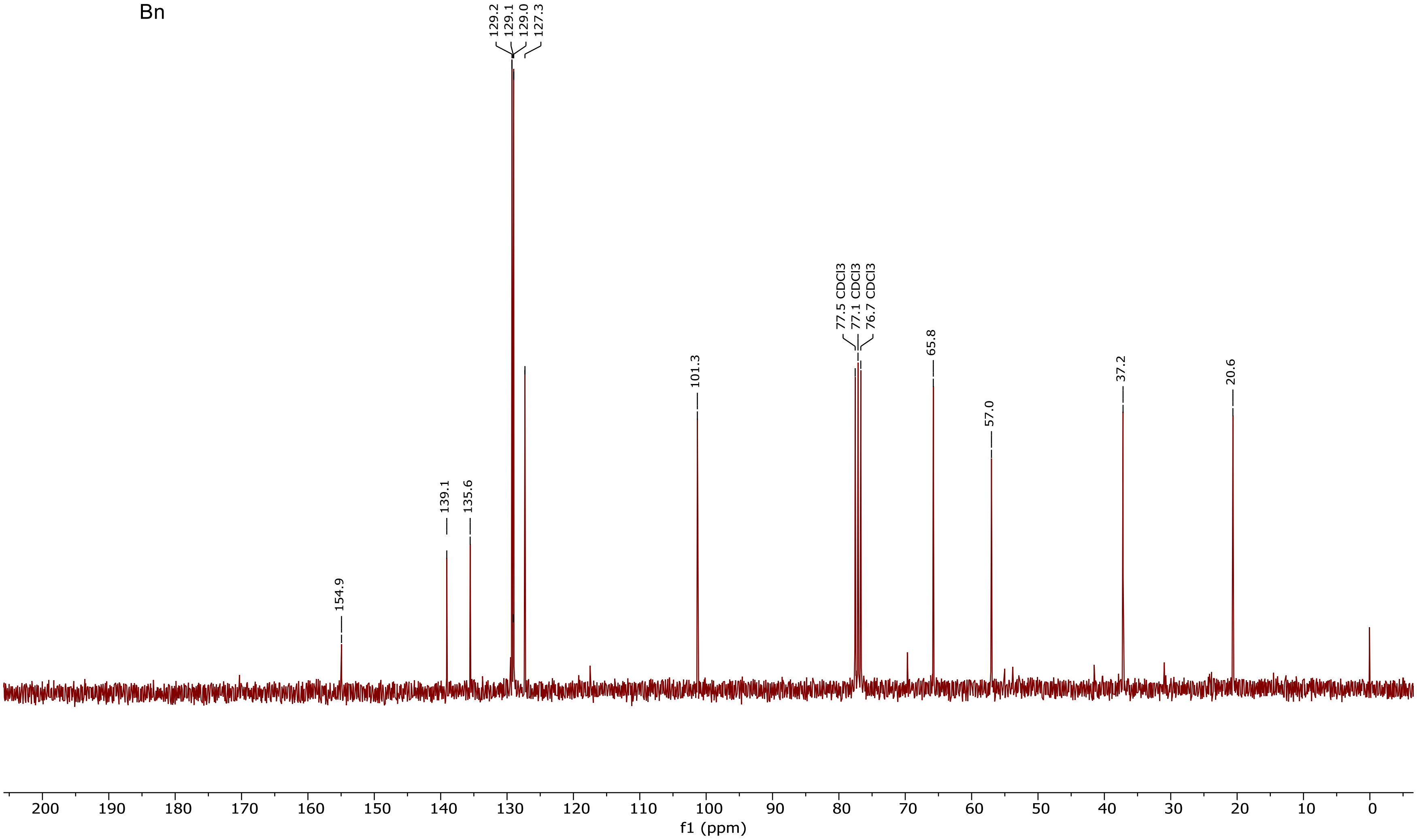

(*S*)-4-Isopropyl-3-(prop-1-en-2-yl)oxazolidin-2-one (**61c**)

^{1}H NMR (300 MHz, CD_2Cl_2)

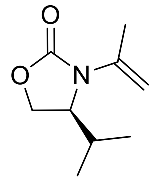

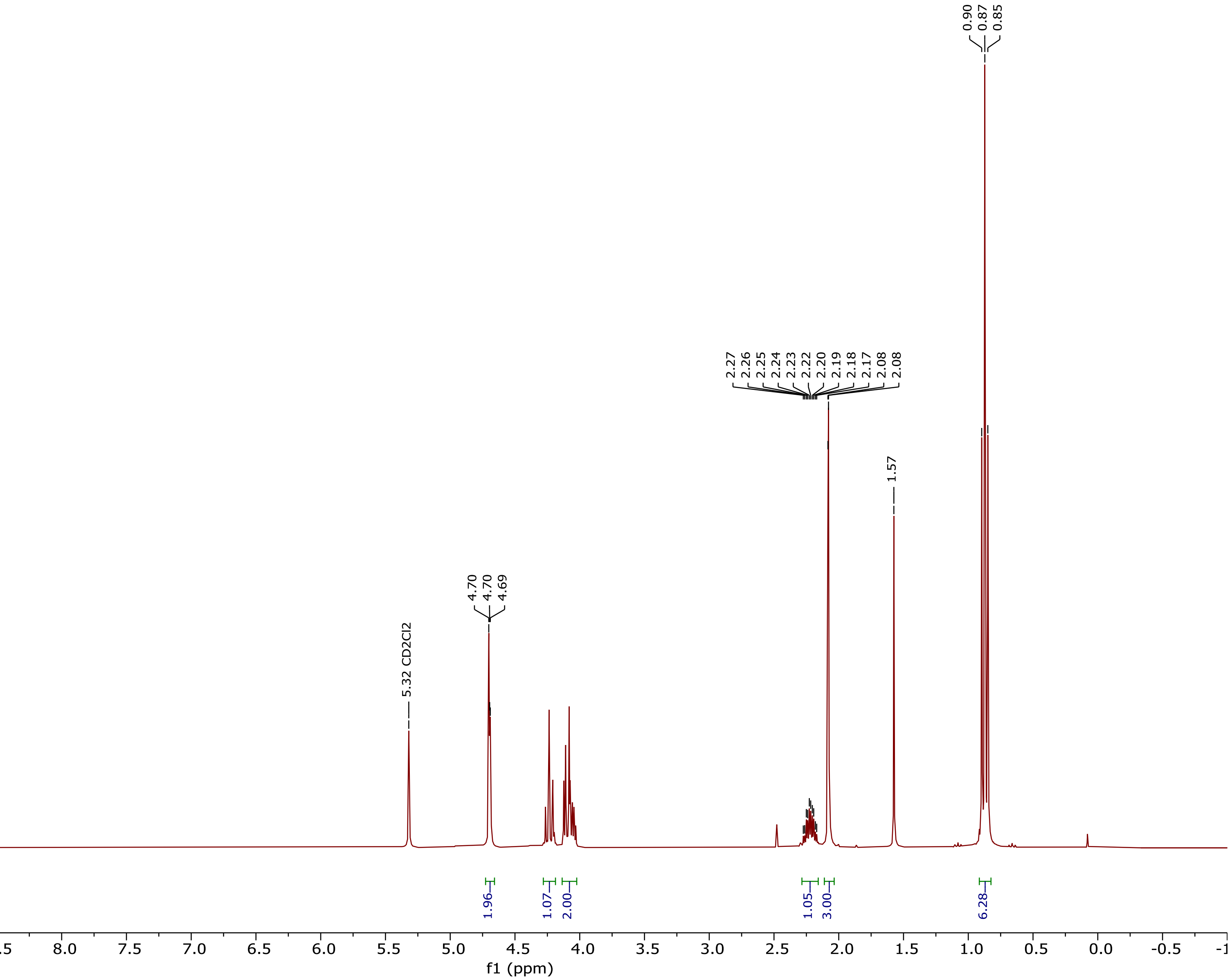

(*S*)-4-Isopropyl-3-(prop-1-en-2-yl)oxazolidin-2-one (**61c**)

^{13}C NMR (75 MHz, CD_2Cl_2)

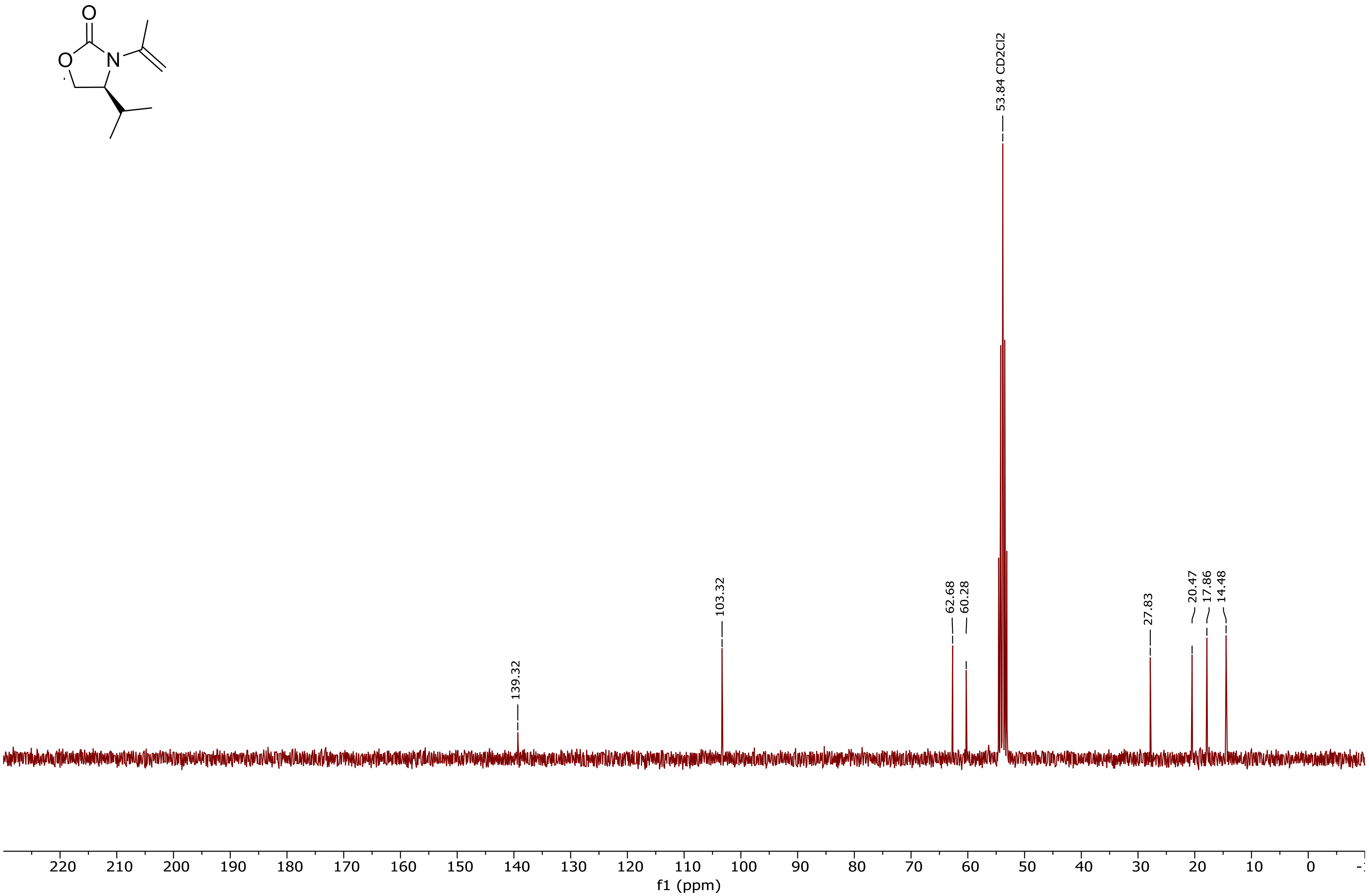

(*S*,*E*)-3-(But-1-en-1-yl)-4-phenyloxazolidin-2-one (**61e**)

^{1}H NMR (300 MHz, $CDCl_3$)

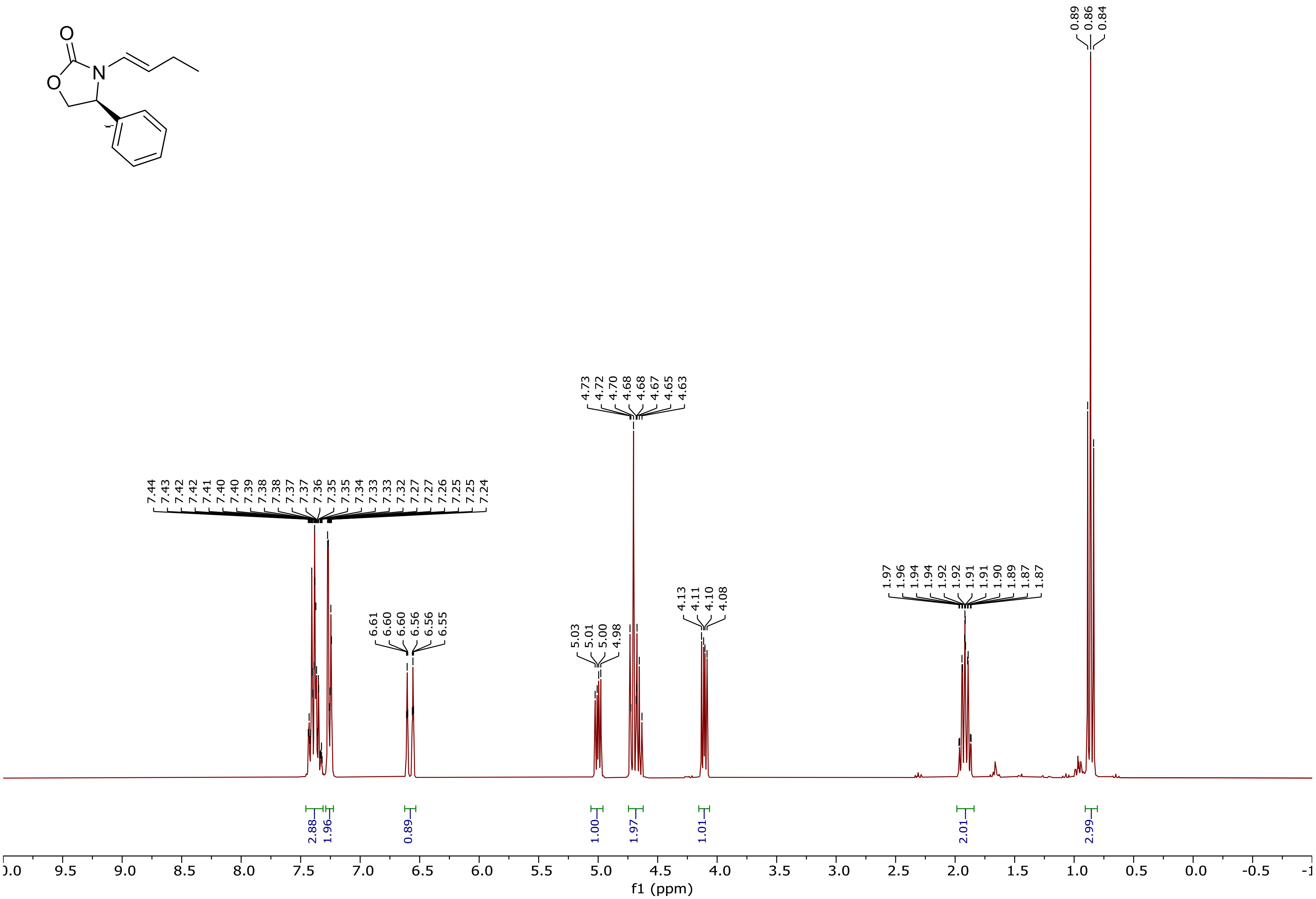

(*S*,*E*)-3-(But-1-en-1-yl)-4-phenyloxazolidin-2-one (**61e**)

^{13}C NMR (75 MHz, $CDCl_3$)

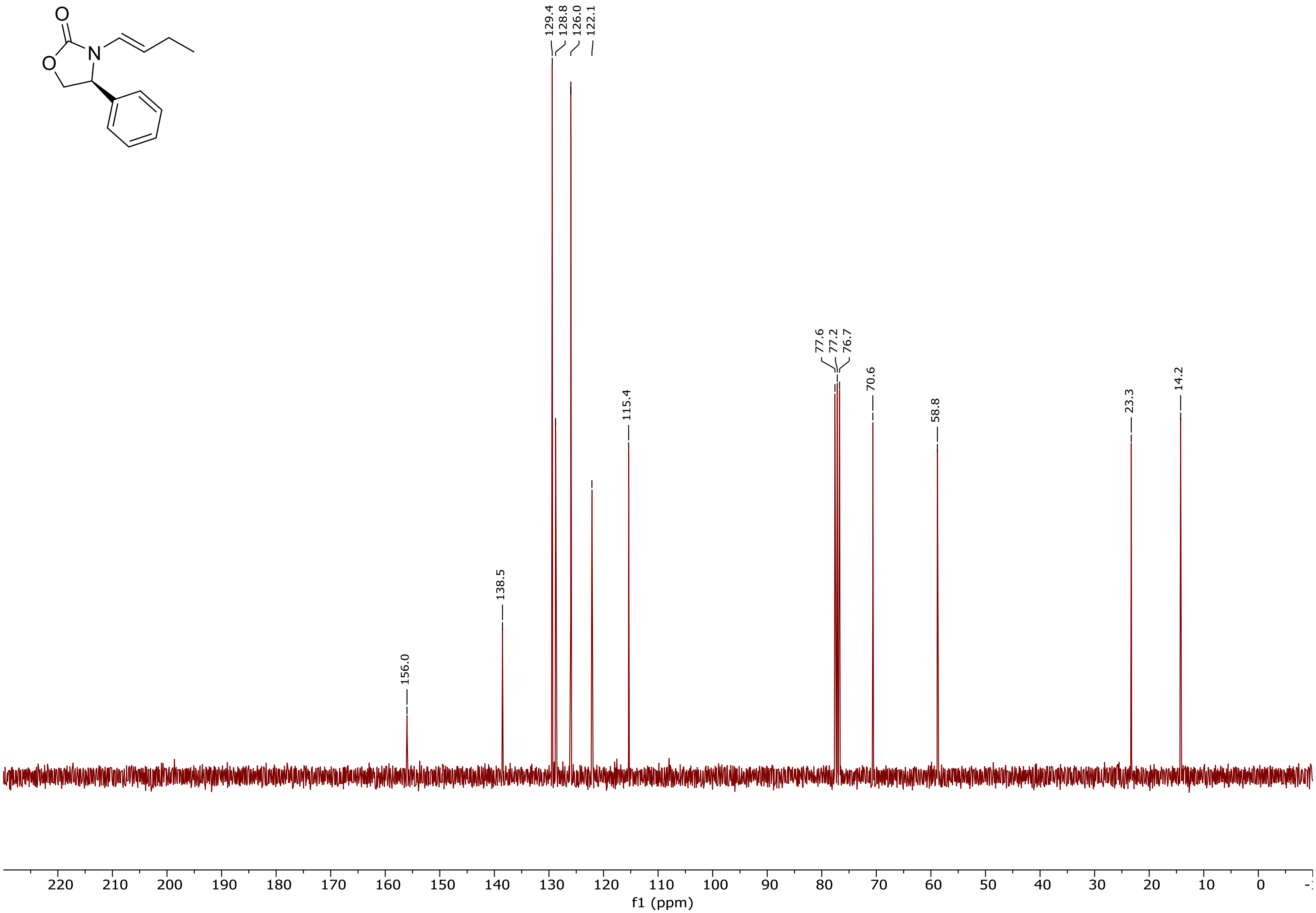

(*4S*,*5R*)-3-((*E*)-But-1-en-1-yl)-4,5-diphenyloxazolidin-2-one (**61g**) ^{1}H NMR (300 MHz, $CDCl_3$)

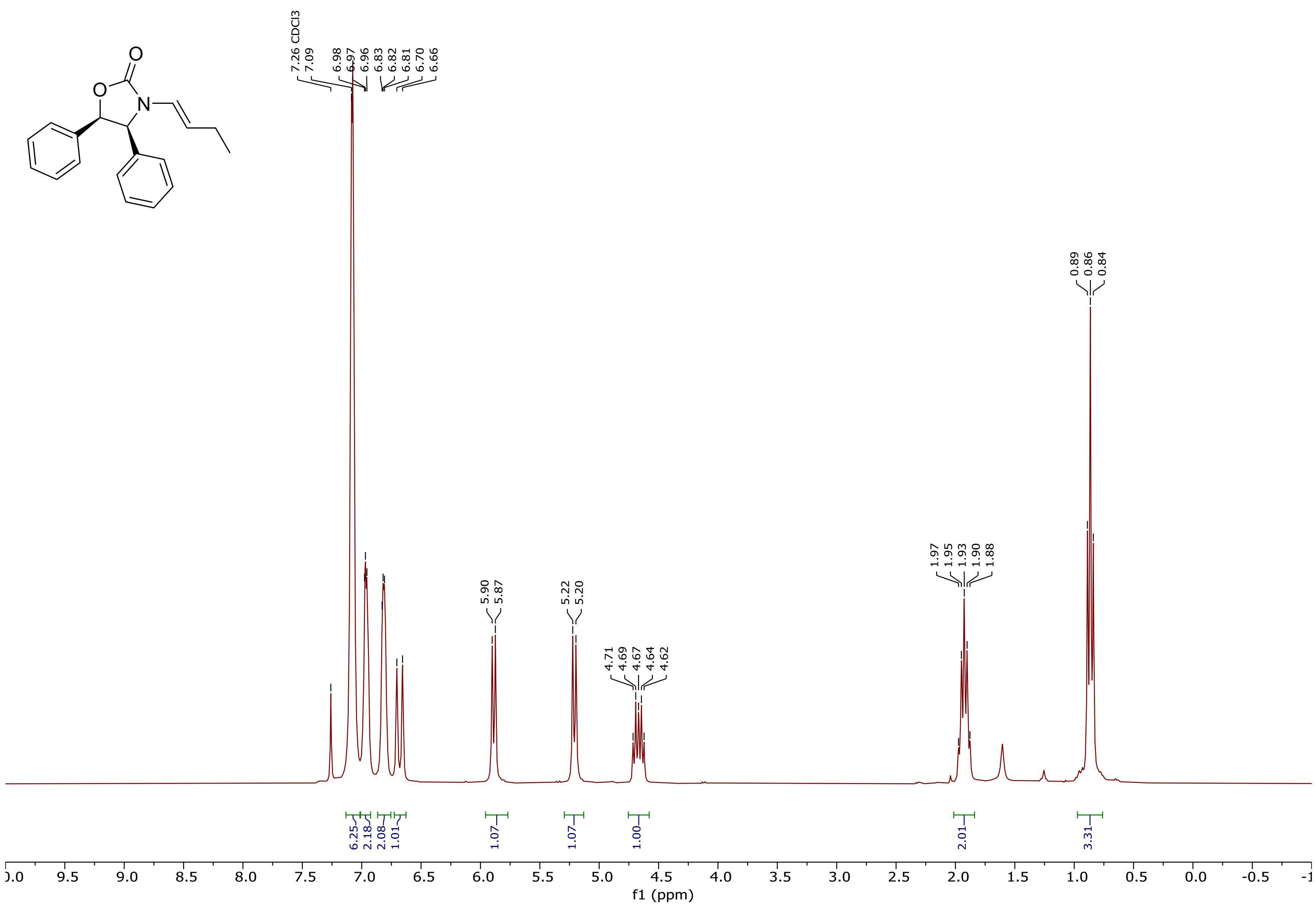

(4*S*,5*R*)-3-((*E*)-But-1-en-1-yl)-4,5-diphenyloxazolidin-2-one (**61g**) ^{13}C NMR (75 MHz, $CDCl_3$)

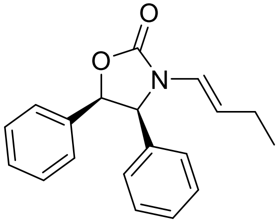

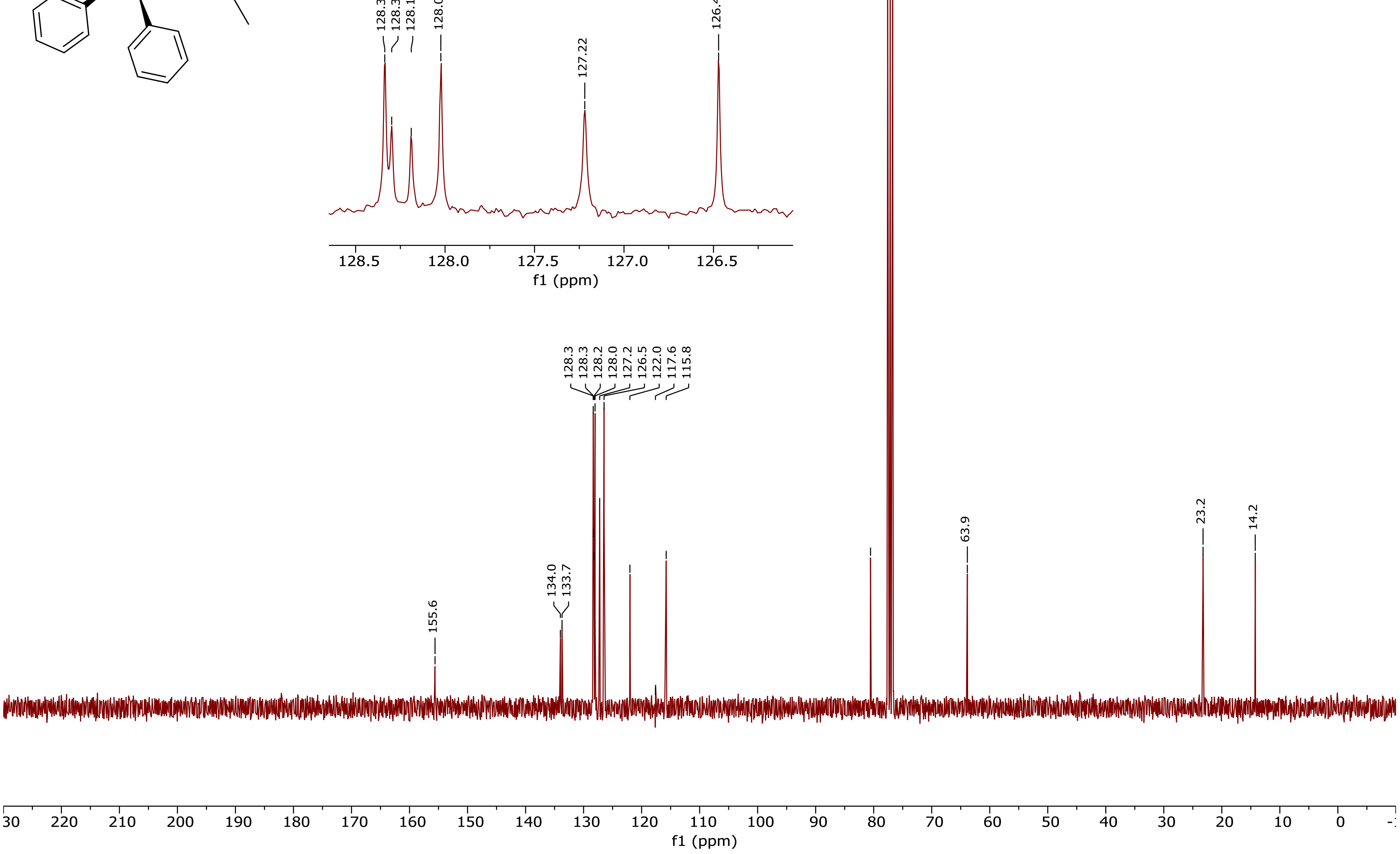

4-Ethoxy-4-oxobutyl benzoate (**3**)

^{1}H NMR (300 MHz, $CDCl_3$)

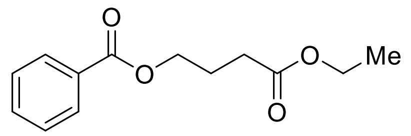

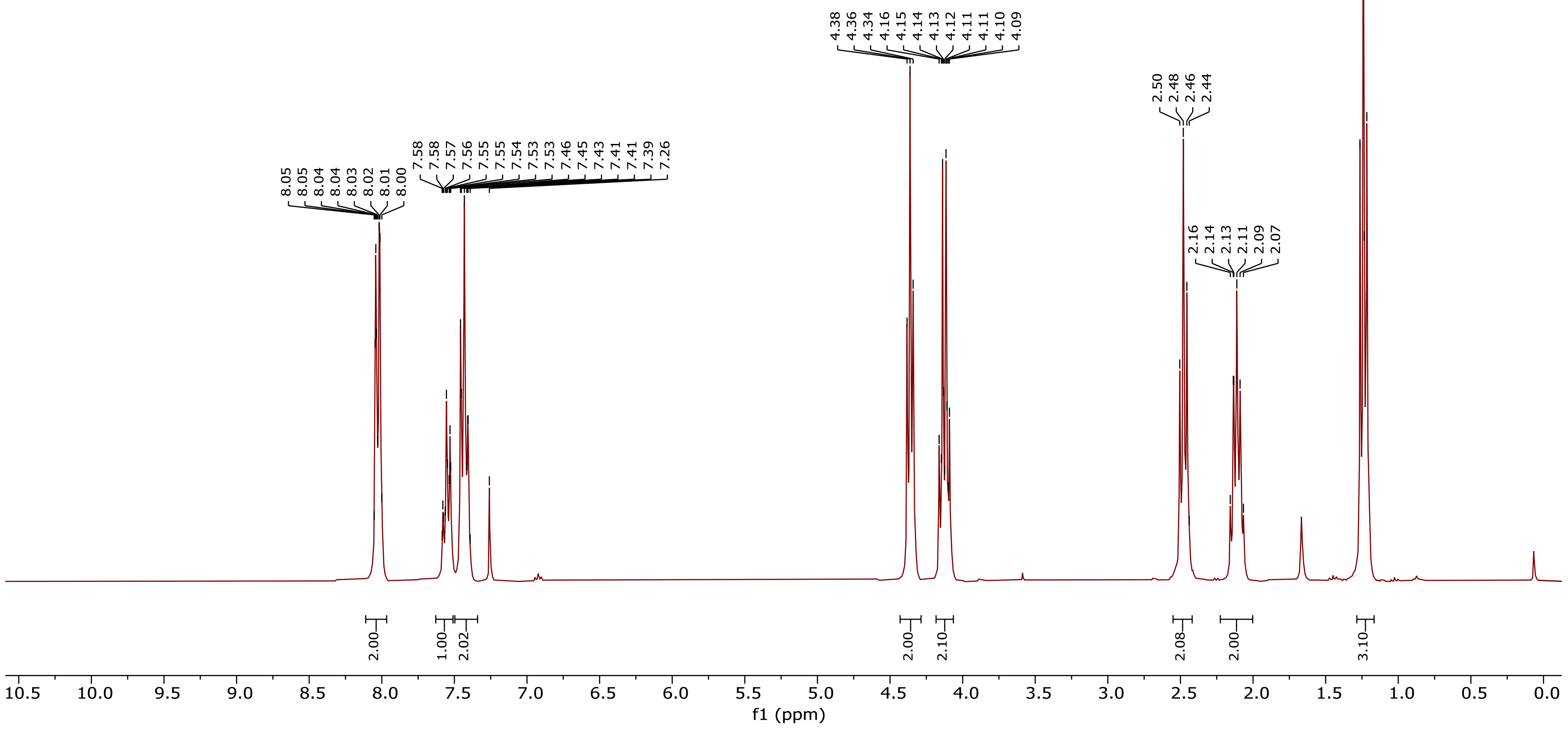

4-Ethoxy-4-oxobutyl benzoate (**3**)

^{13}C NMR (75 MHz, $CDCl_3$)

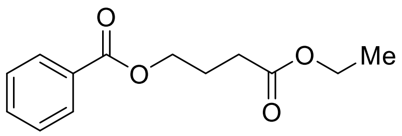

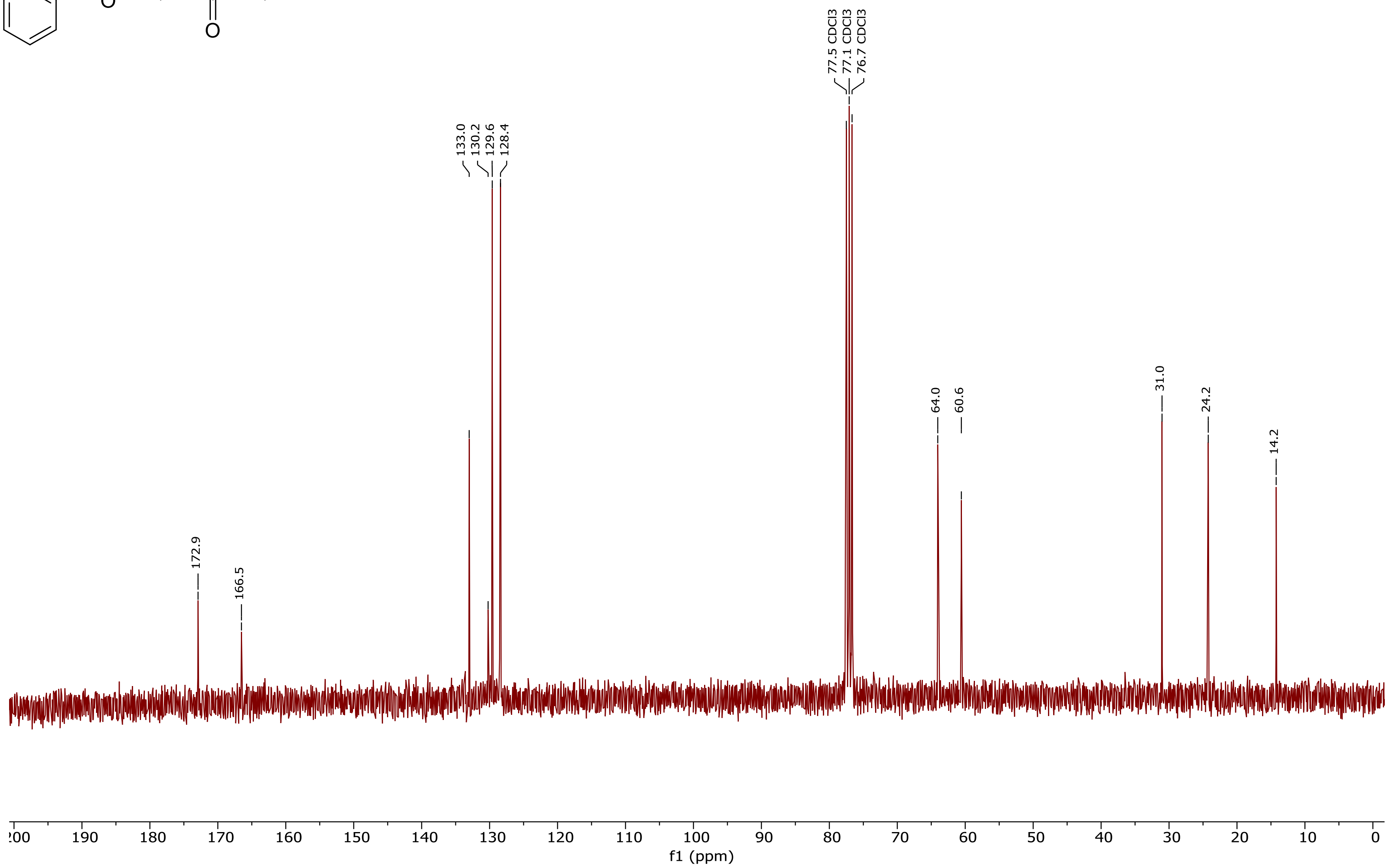

4-(Benzyloxy)-4-oxobutyl benzoate (**4**) ^{1}H NMR (300 MHz, $CDCl_3$)

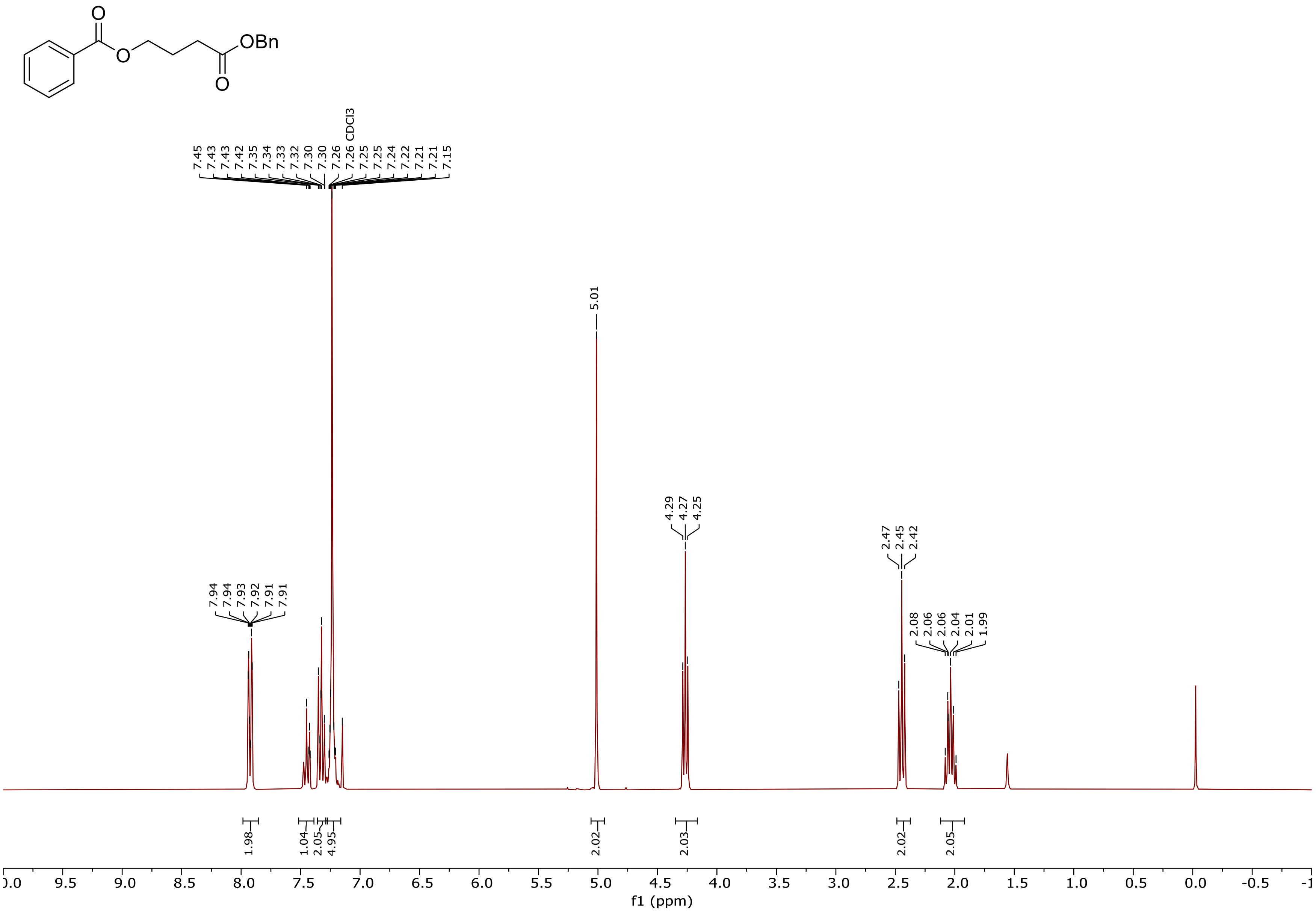

4-(Benzyloxy)-4-oxobutyl benzoate (**4**)

^{13}C NMR (75 MHz, $CDCl_3$)

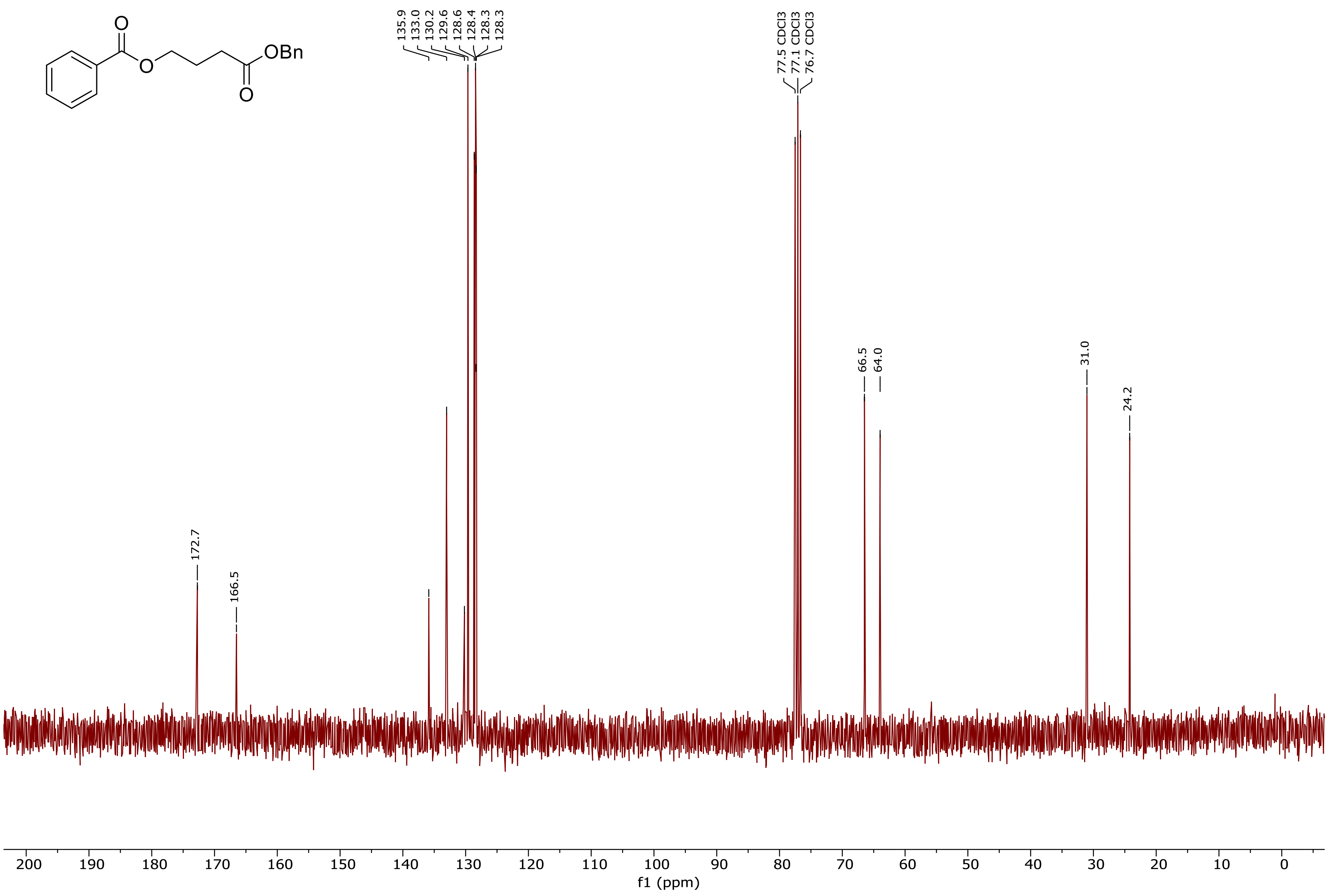

4-Oxo-4-phenoxybutyl benzoate (**5**)

^{1}H NMR (300 MHz, $CDCl_3$)

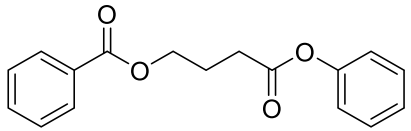

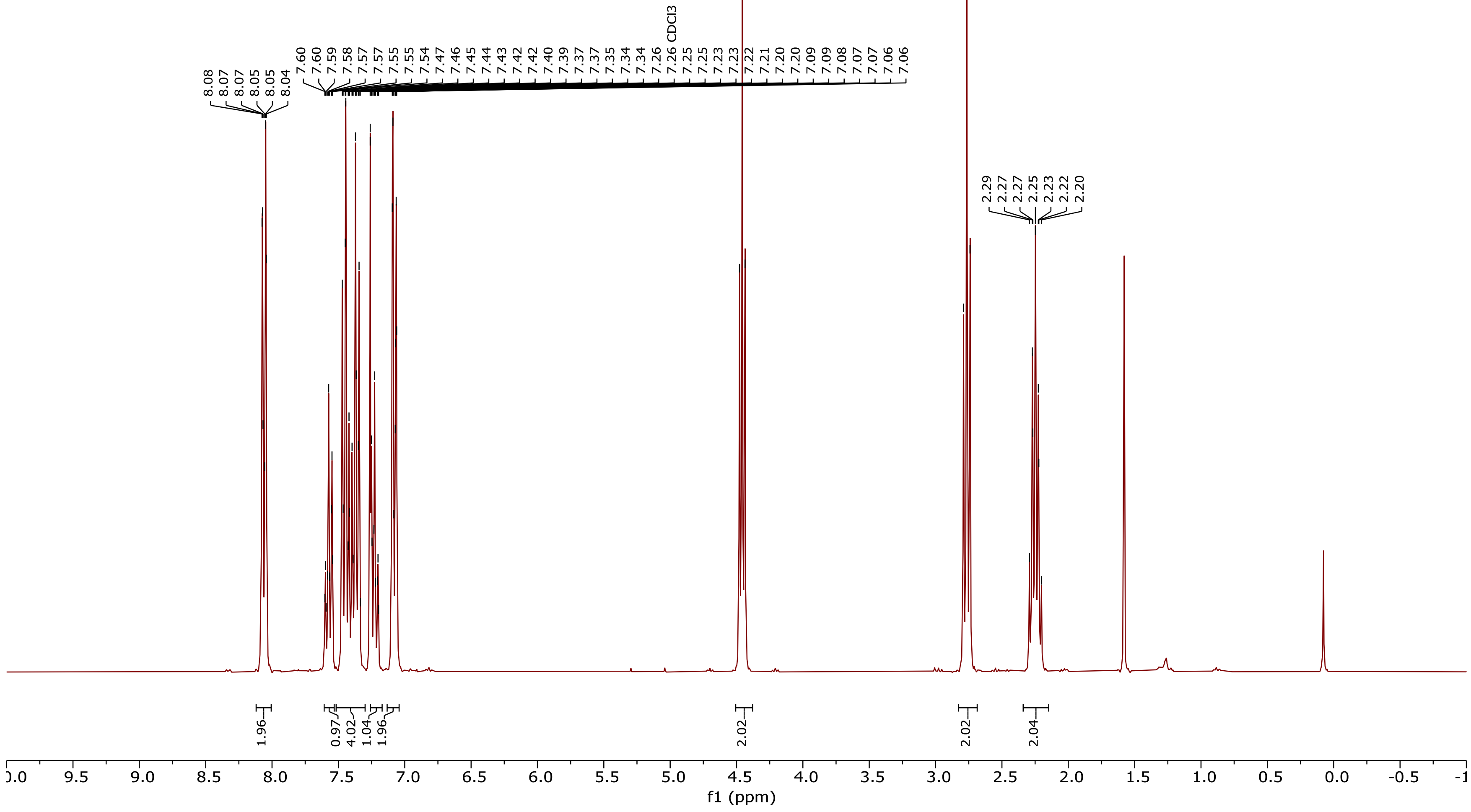

4-Oxo-4-phenoxybutyl benzoate (**5**)

^{13}C NMR (75 MHz, $CDCl_3$)

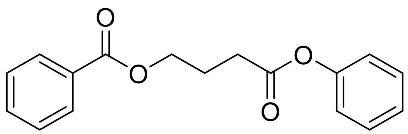

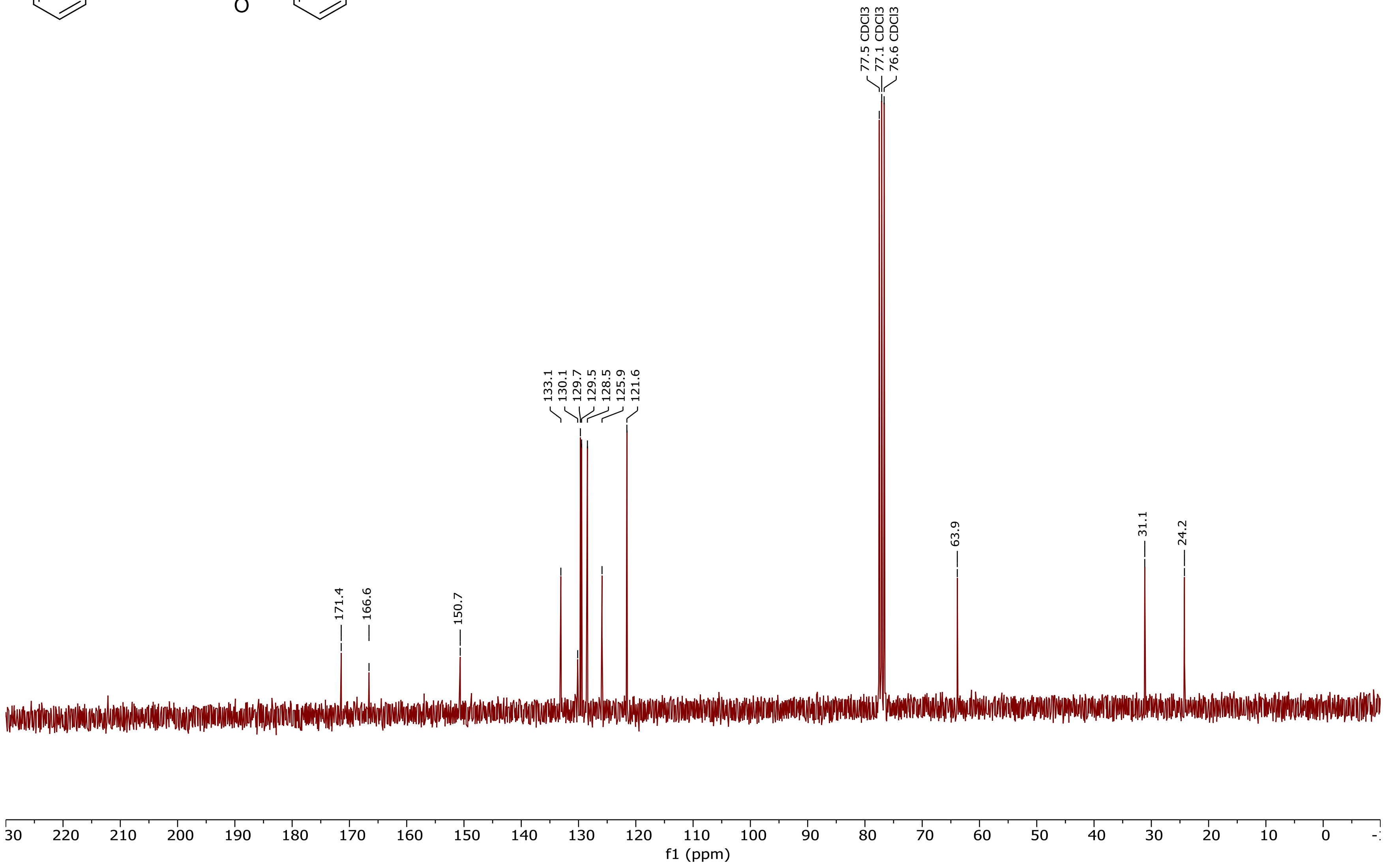

Benzyl 4-acetoxybutanoate (**6**)

^{1}H NMR (300 MHz, $CDCl_3$)

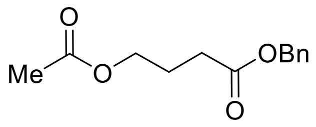

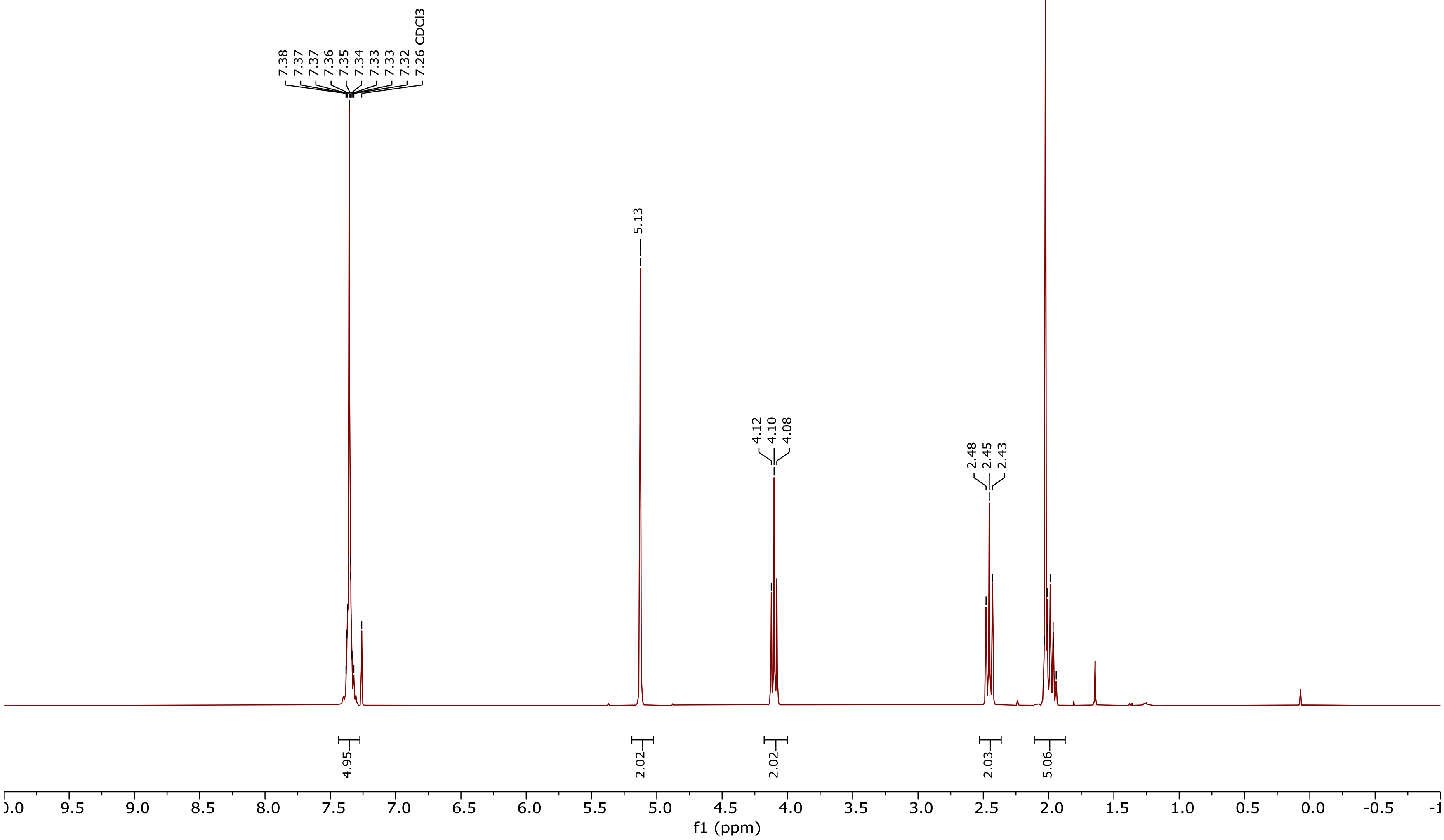

Benzyl 4-acetoxybutanoate (**6**)

^{13}C NMR (75 MHz, $CDCl_3$)

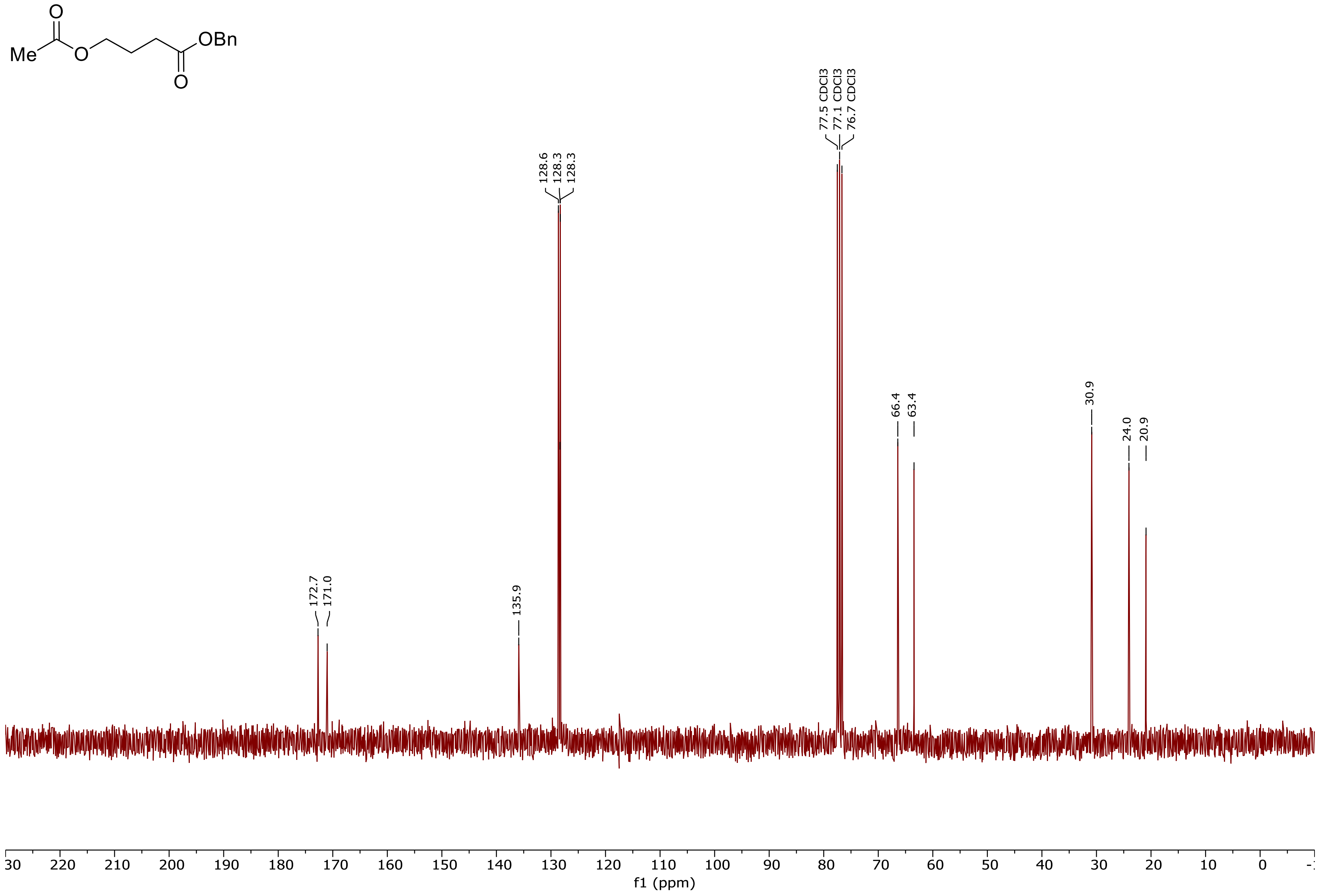

Phenyl 4-acetoxybutanoate (**7**) ^{1}H NMR (300 MHz, $CDCl_3$)

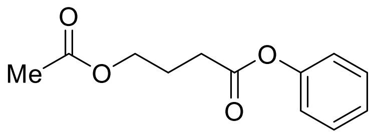

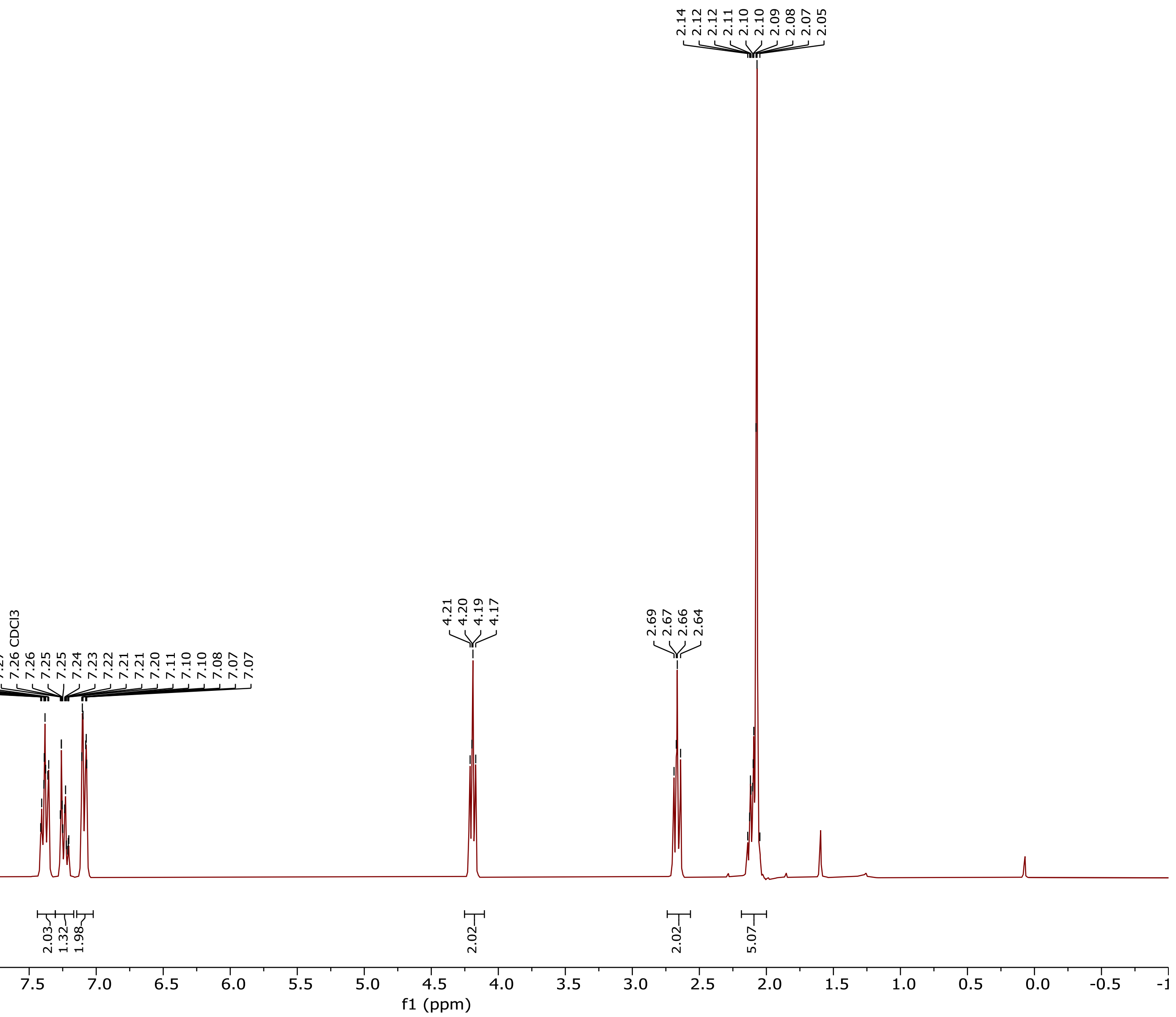

Phenyl 4-acetoxybutanoate (**7**)

^{13}C NMR (75 MHz, $CDCl_3$)

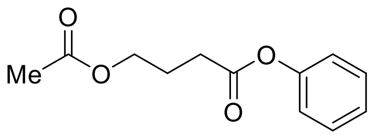

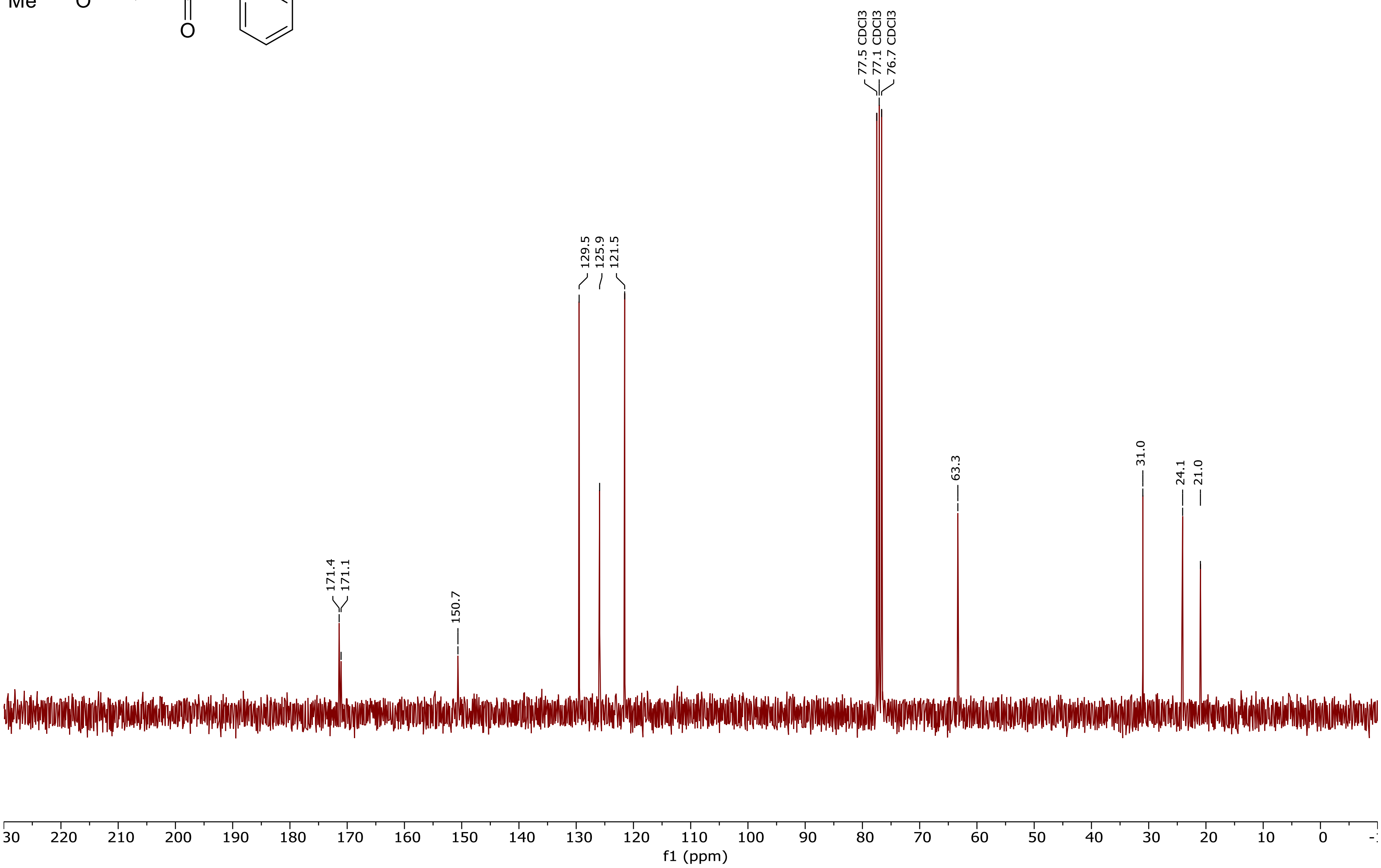

4-Ethoxy-3-methyl-4-oxobutyl benzoate (**8**)

^{1}H NMR (300 MHz, $CDCl_3$)

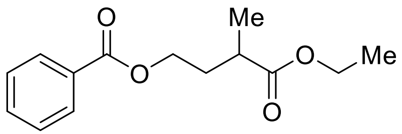

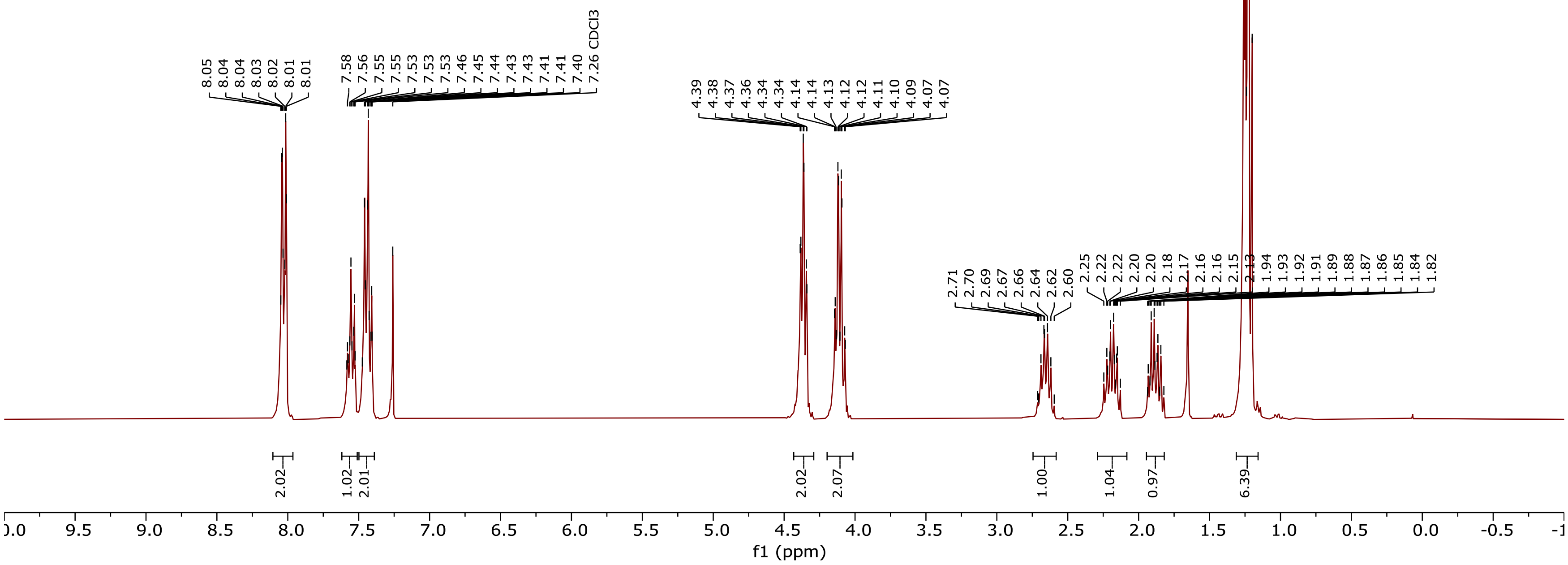

4-Ethoxy-3-methyl-4-oxobutyl benzoate (**8**)

^{13}C NMR (75 MHz, $CDCl_3$)

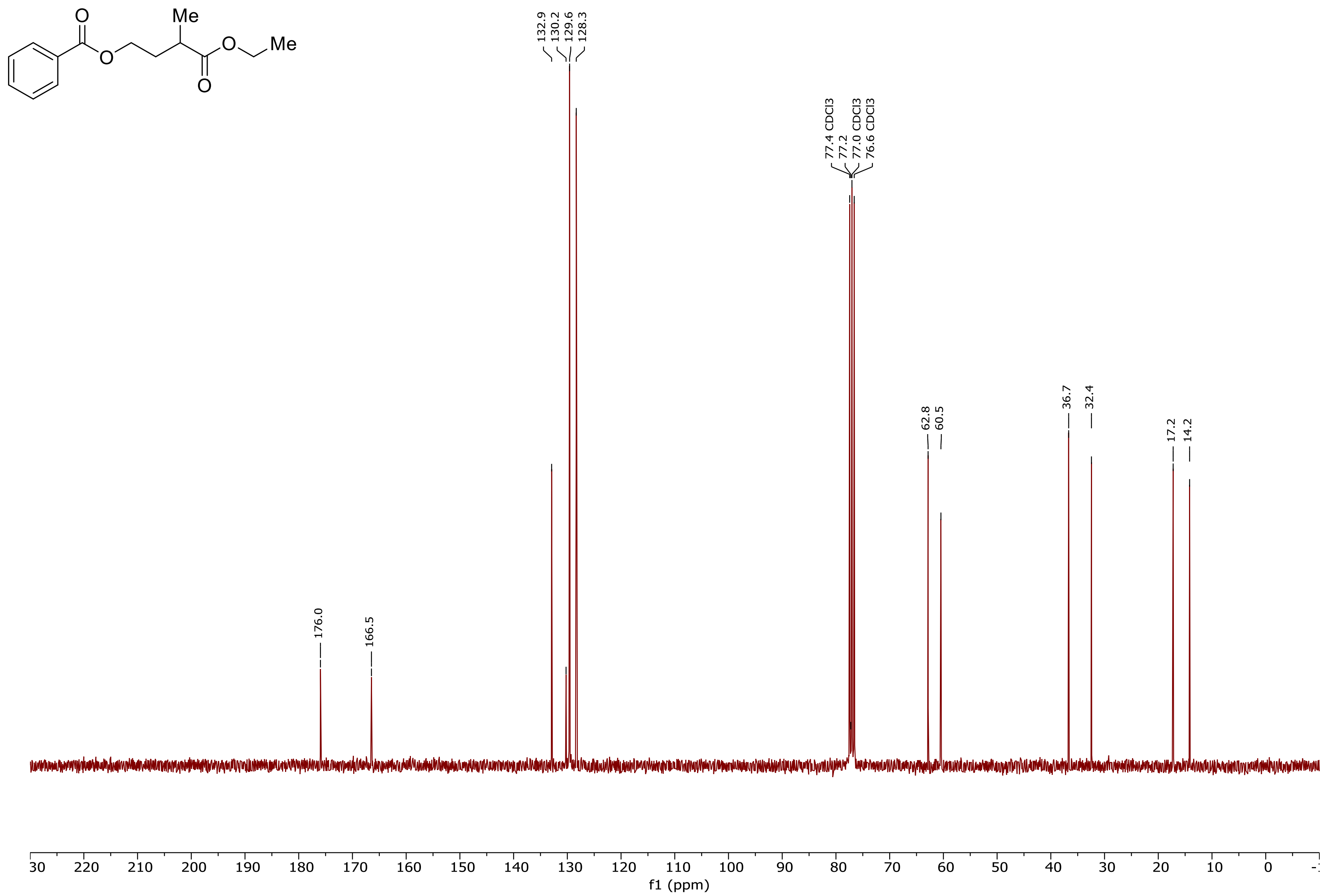

2-(2-Oxotetrahydrofuran-3-yl)ethyl benzoate (**9**)

^{1}H NMR (300 MHz, $CDCl_3$)

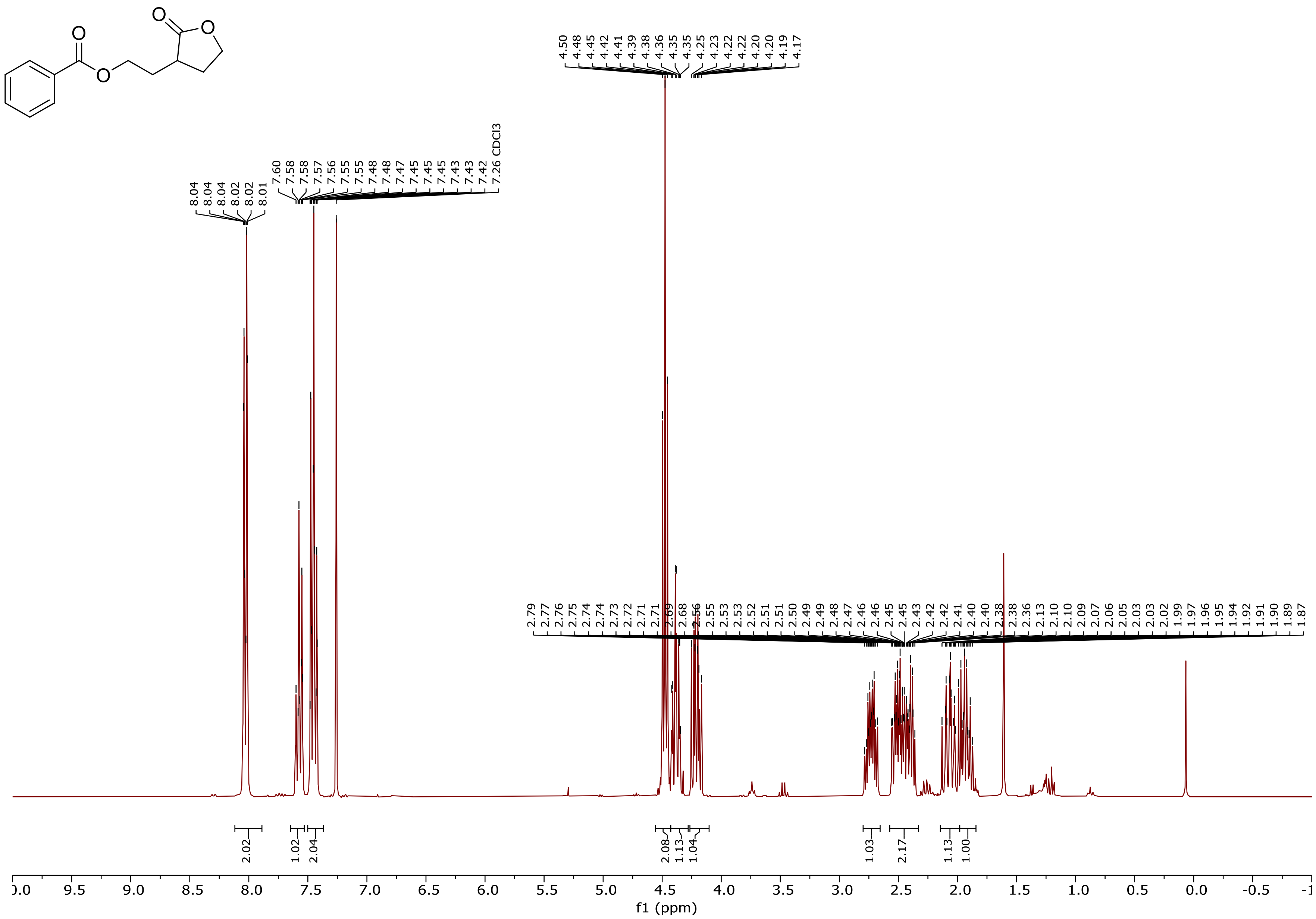

2-(2-Oxotetrahydrofuran-3-yl)ethyl benzoate (**9**)

^{13}C NMR (75 MHz, $CDCl_3$)

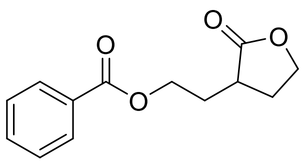

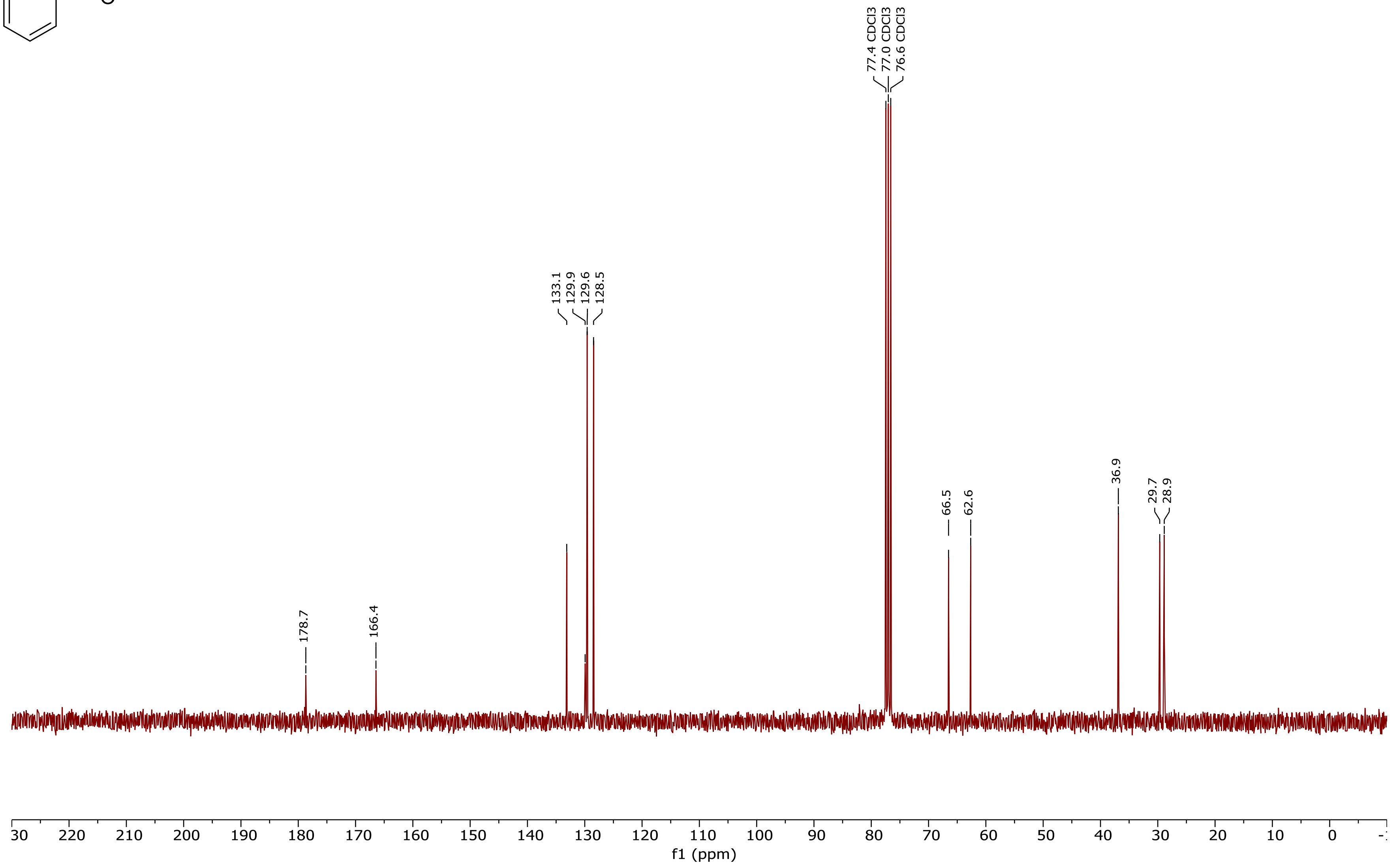

4-Ethoxy-3,3-difluoro-4-oxobutyl benzoate (**10**)

^1H NMR (300 MHz, $CDCl_3$)

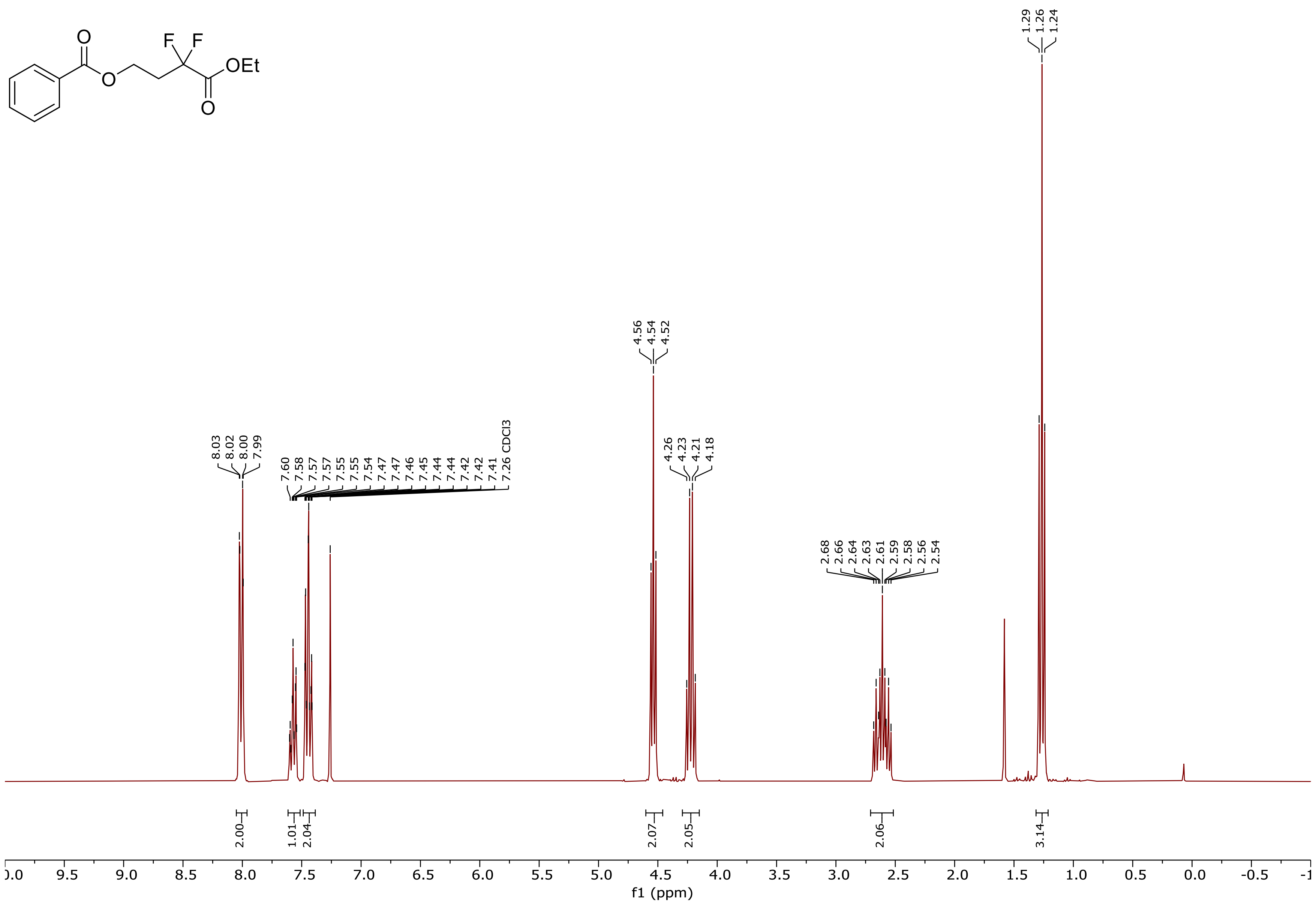

4-Ethoxy-3,3-difluoro-4-oxobutyl benzoate (**10**)

^{13}C NMR (75 MHz, $CDCl_3$)

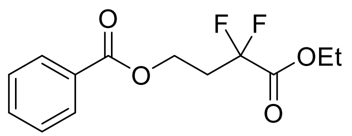

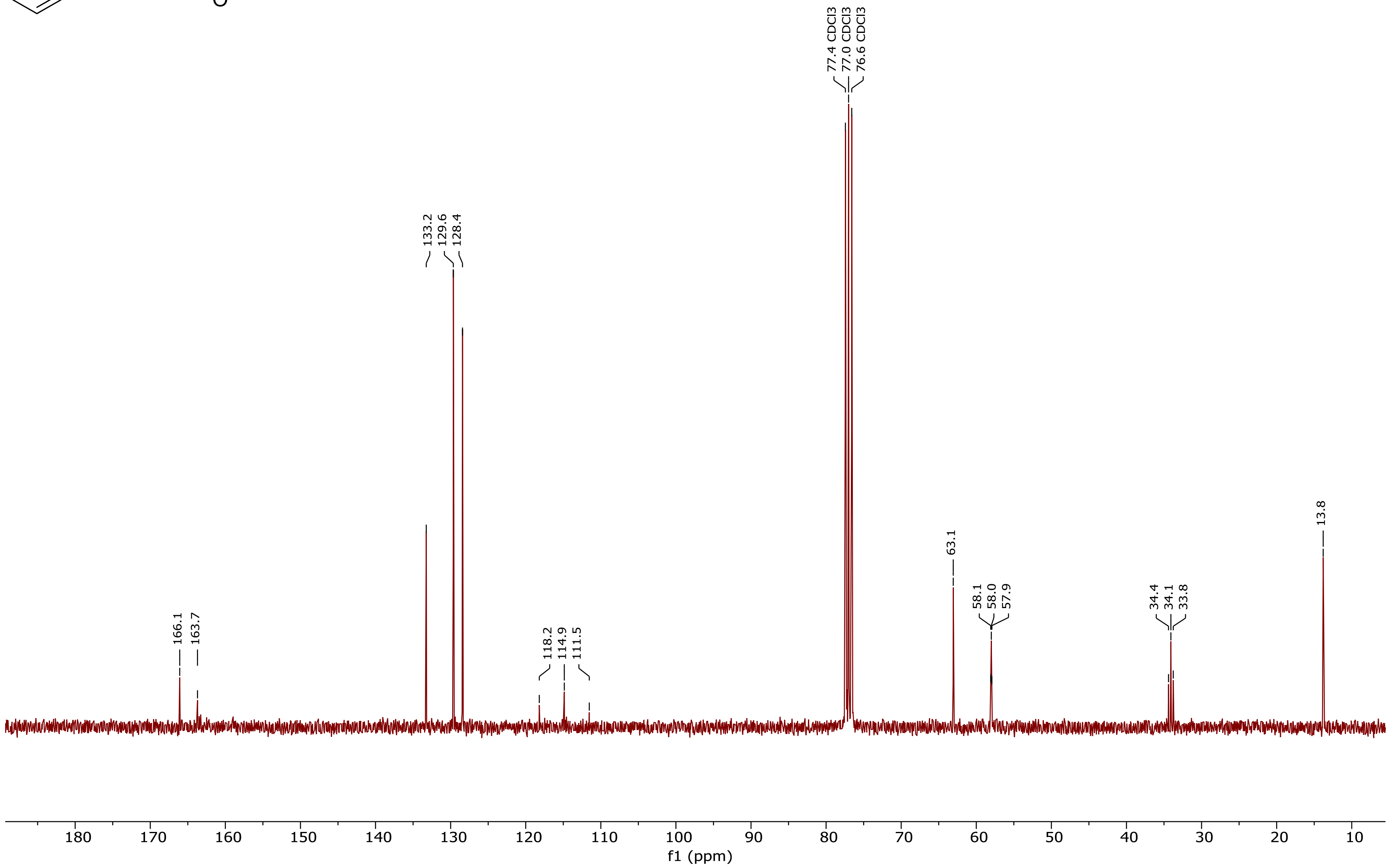

Benzyl 4-acetoxypentanoate (**11**)

^{1}H NMR (300 MHz, $CDCl_3$)

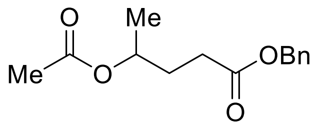

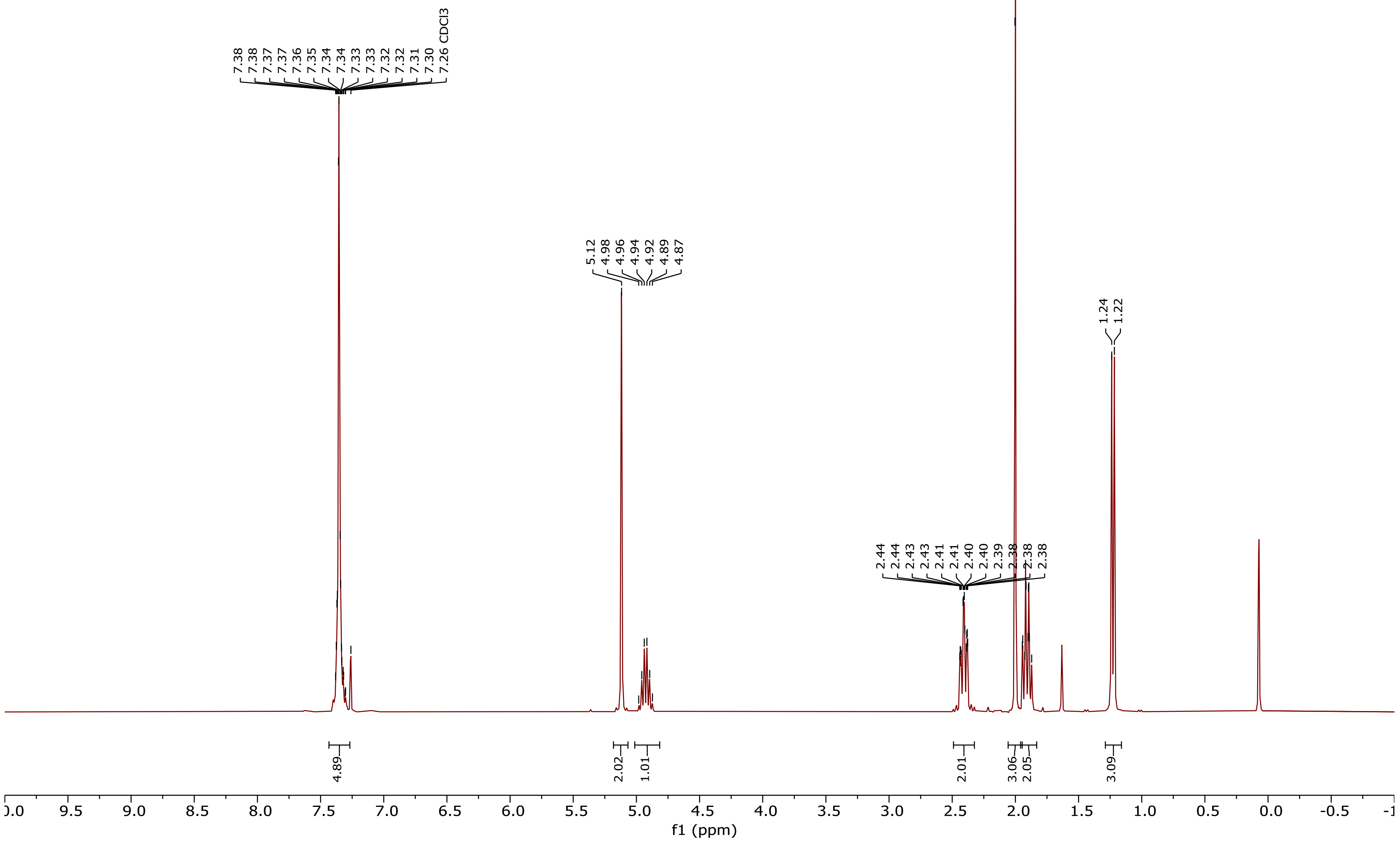

Benzyl 4-acetoxypentanoate (**11**)

^{13}C NMR (75 MHz, $CDCl_3$)

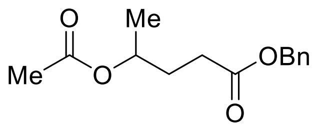

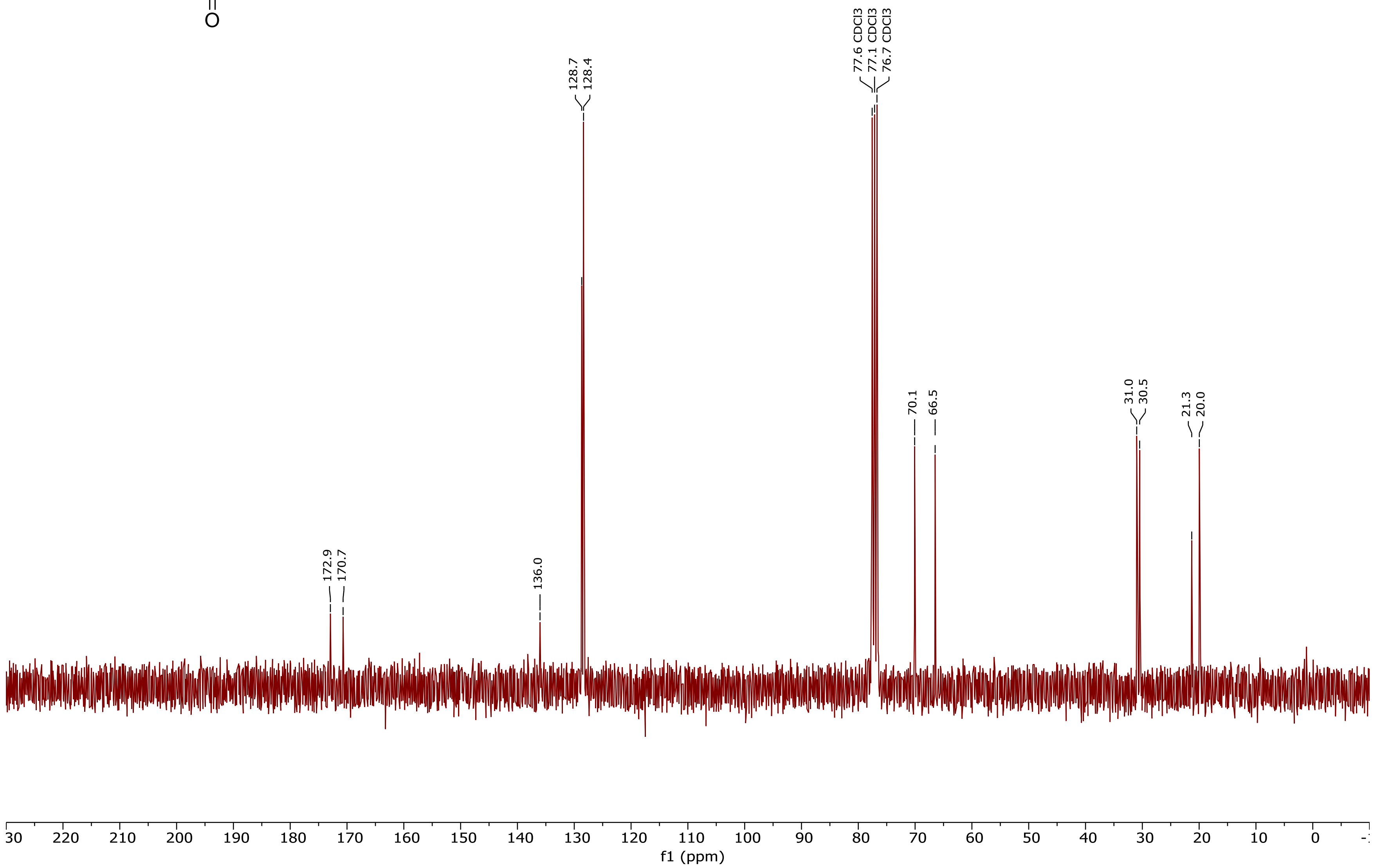

Phenyl 4-acetoxypentanoate (**12**)

^{1}H NMR (300 MHz, $CDCl_3$)

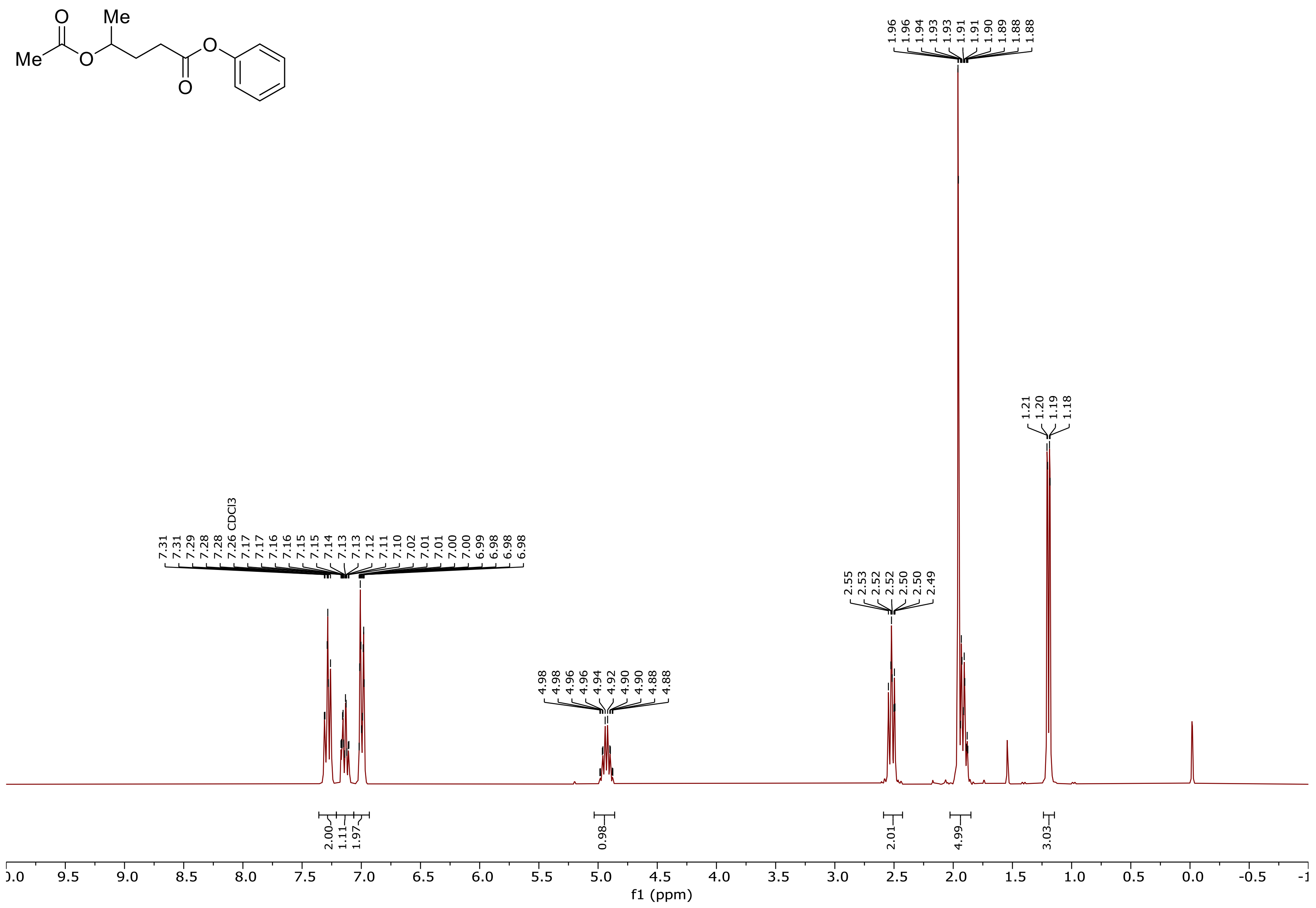

Phenyl 4-acetoxypentanoate (**12**)

^{13}C NMR (75 MHz, $CDCl_3$)

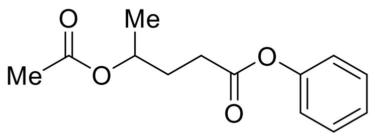

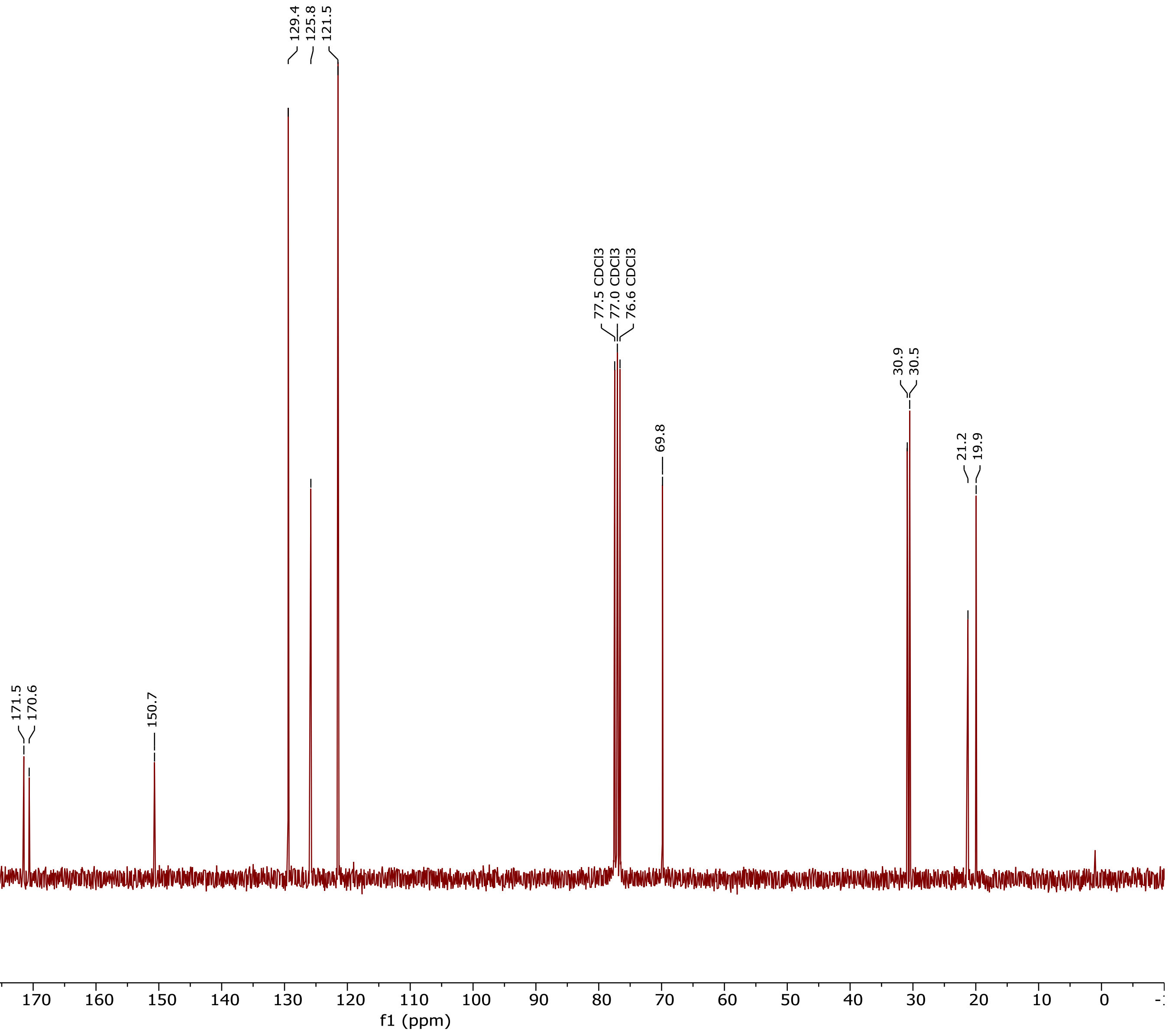

Benzyl 4-acetoxy-3-methylhexanoate (**13**)

^{1}H NMR (300 MHz, $CDCl_3$)

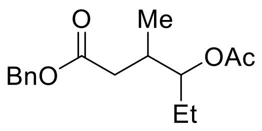

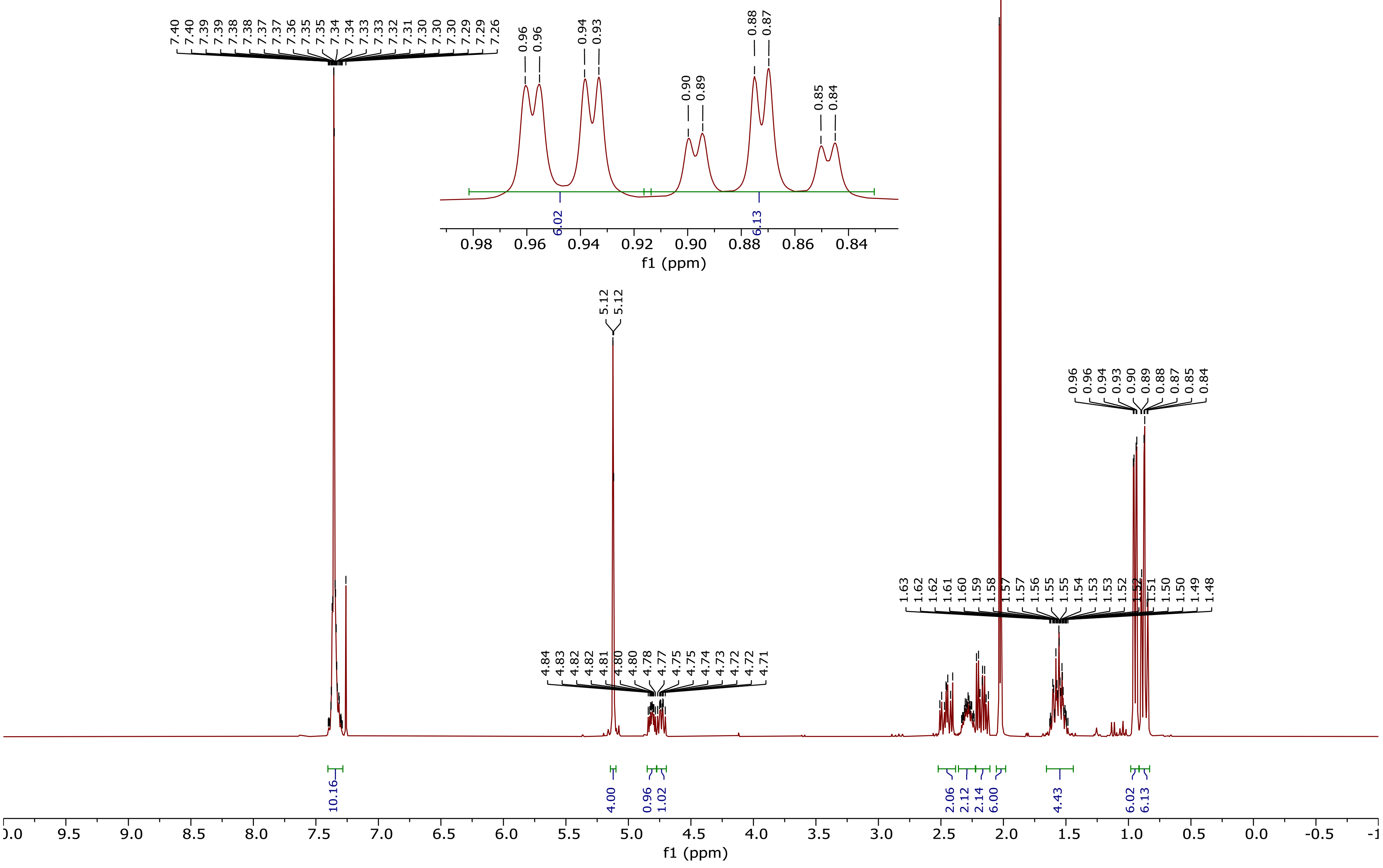

Benzyl 4-acetoxy-3-methylhexanoate (**13**)

^{13}C NMR (75 MHz, $CDCl_3$)

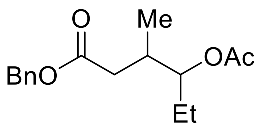

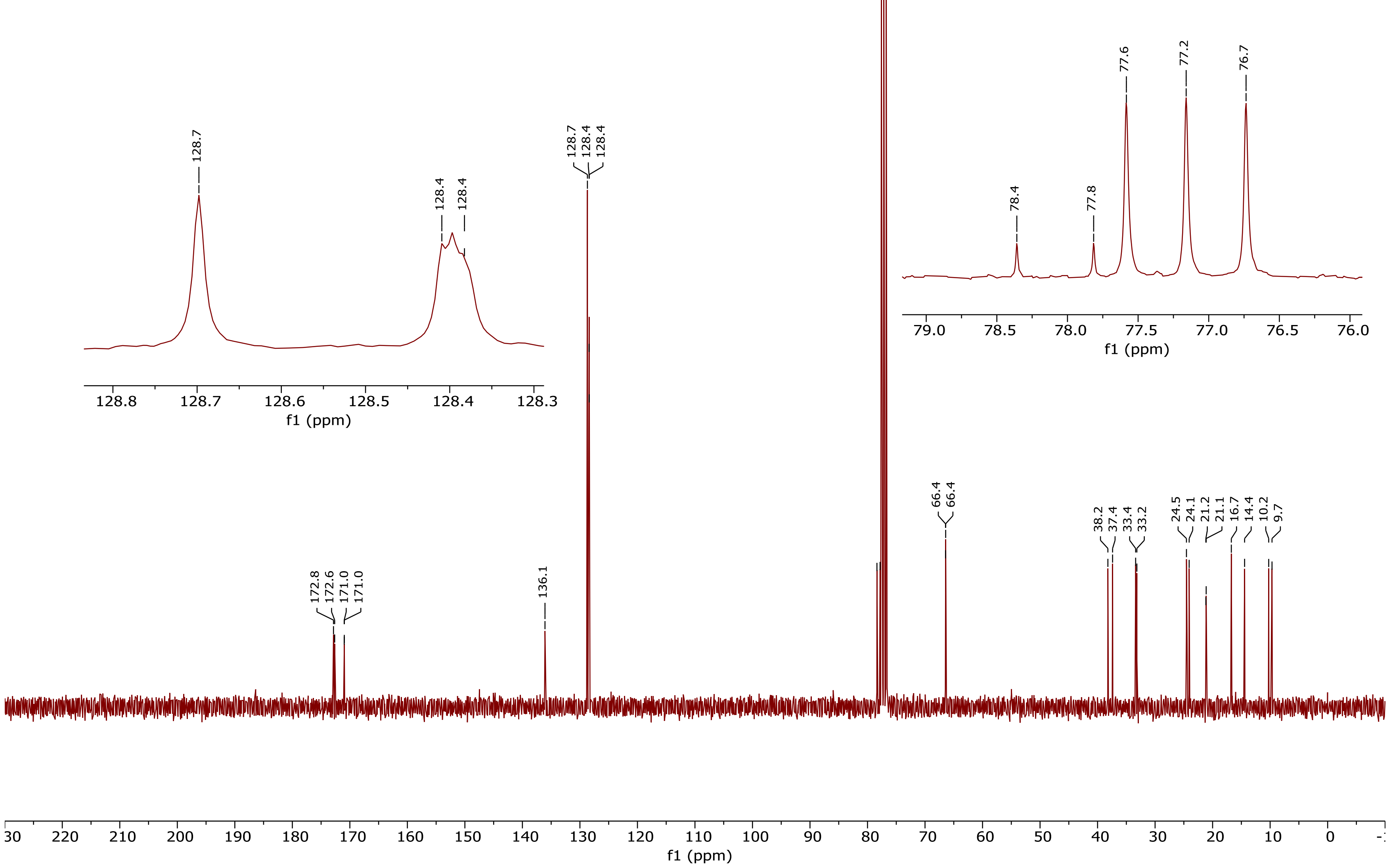

Benzyl 2-(2-acetoxycyclopentyl)acetate (**14**)

^{1}H NMR (300 MHz, $CDCl_3$)

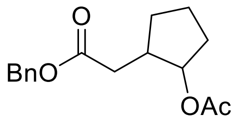

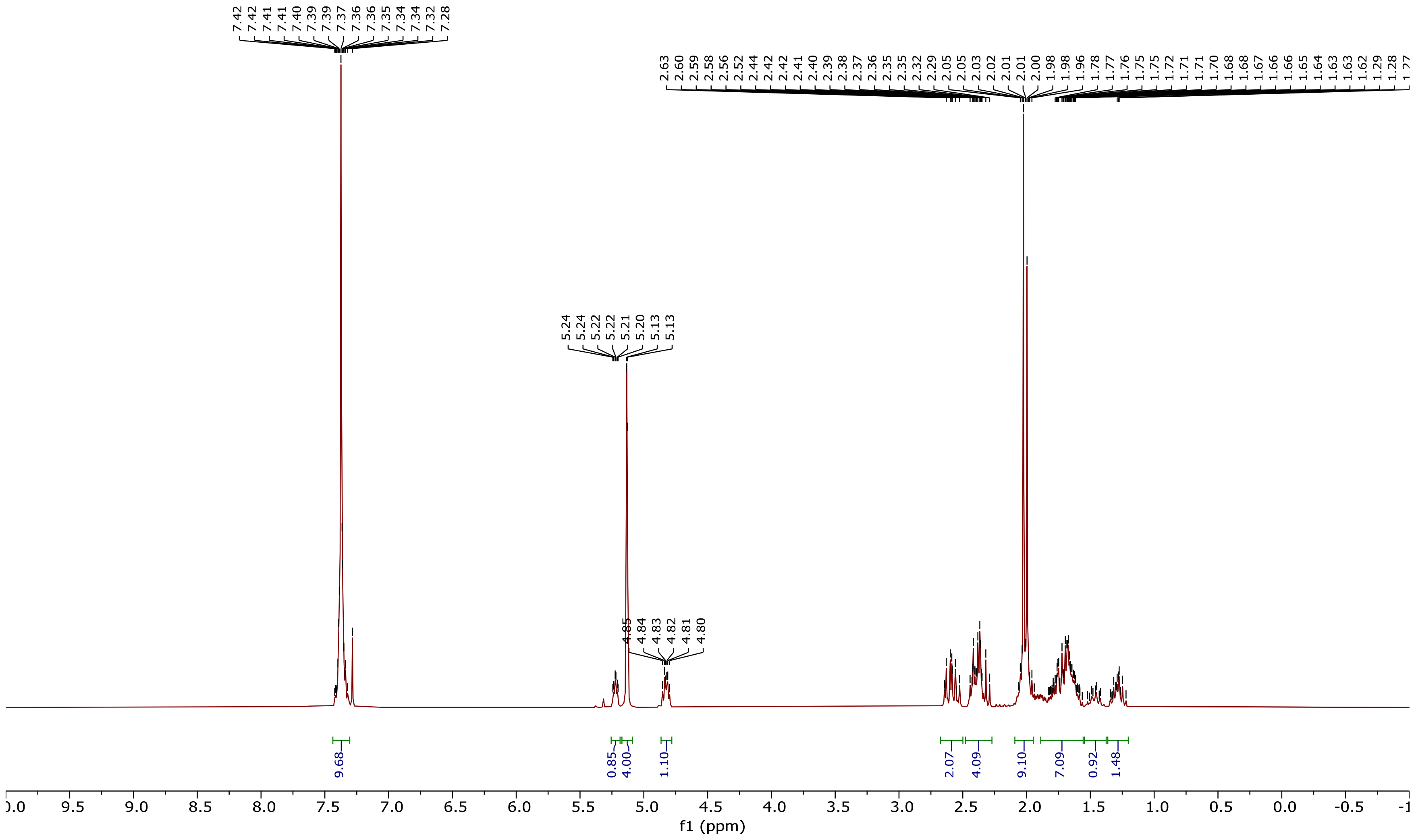

Benzyl 2-(2-acetoxycyclopentyl)acetate (**14**)

^{13}C NMR (75 MHz, $CDCl_3$)

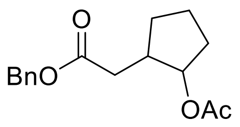

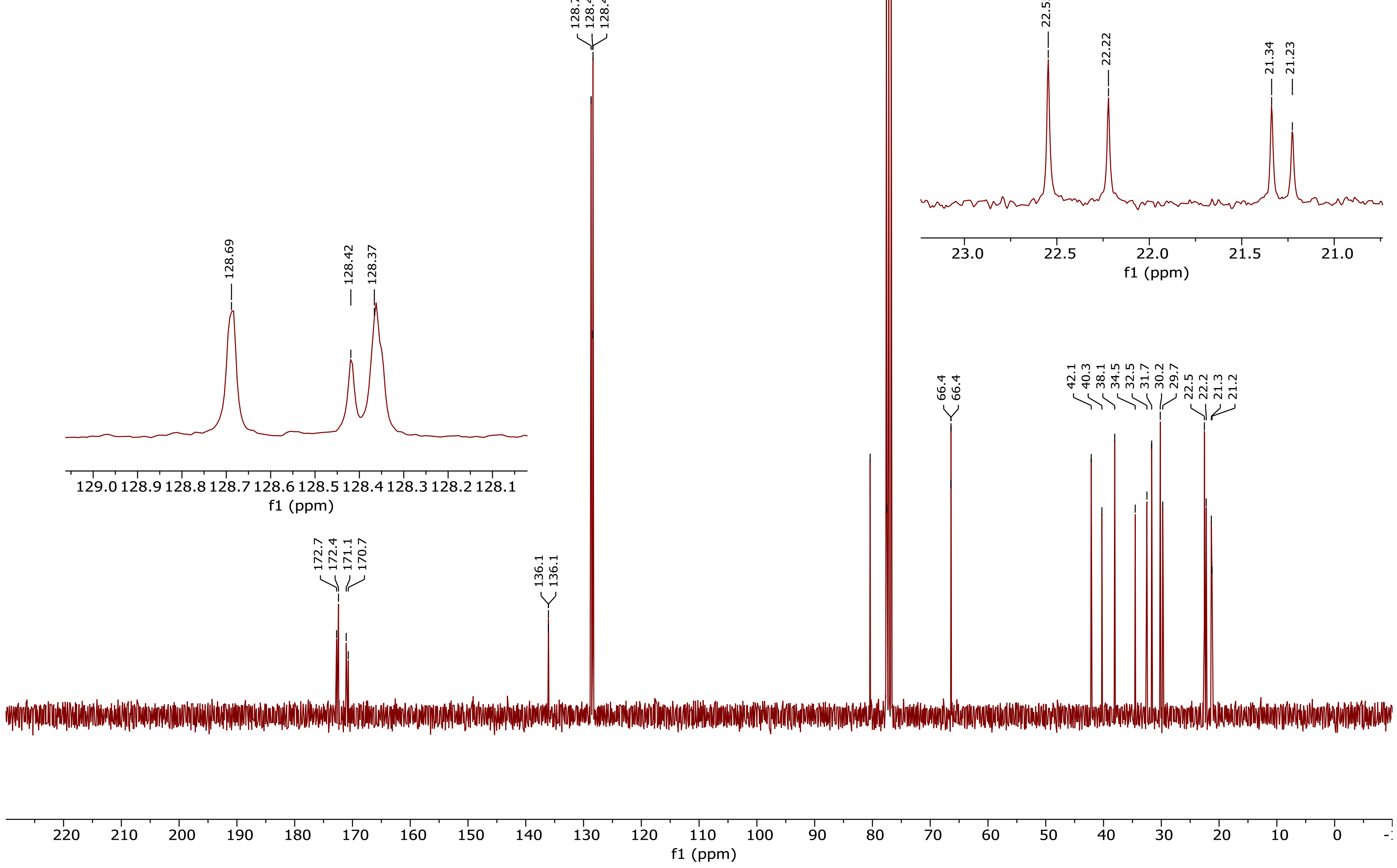

Benzyl 2-(2-acetoxycyclohexyl)acetate (**15**)

^{1}H NMR (300 MHz, $CDCl_3$)

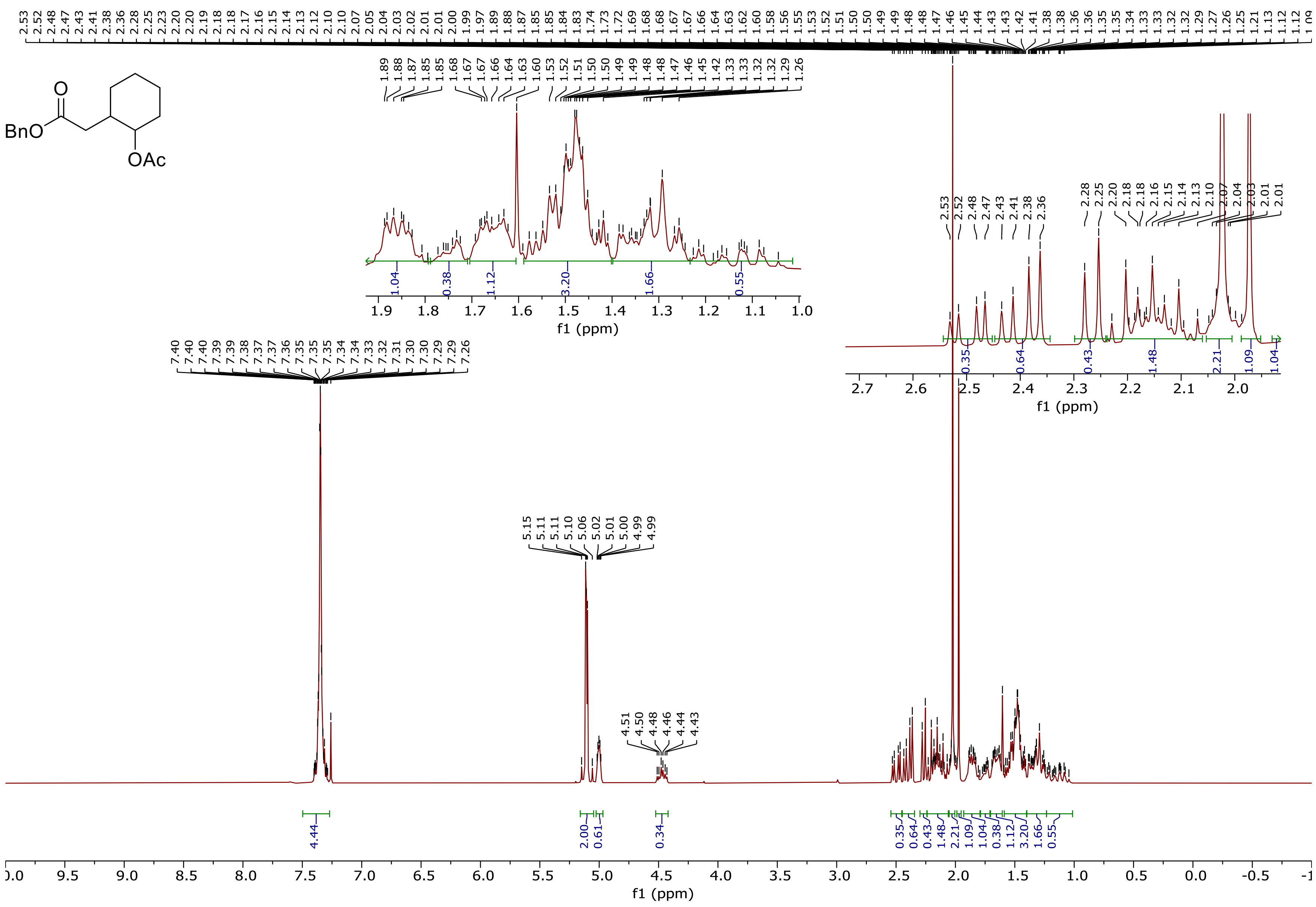

Benzyl 2-(2-acetoxycyclohexyl)acetate (**15**)

^{13}C NMR (75 MHz, $CDCl_3$)

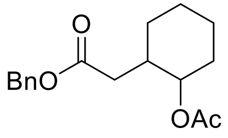

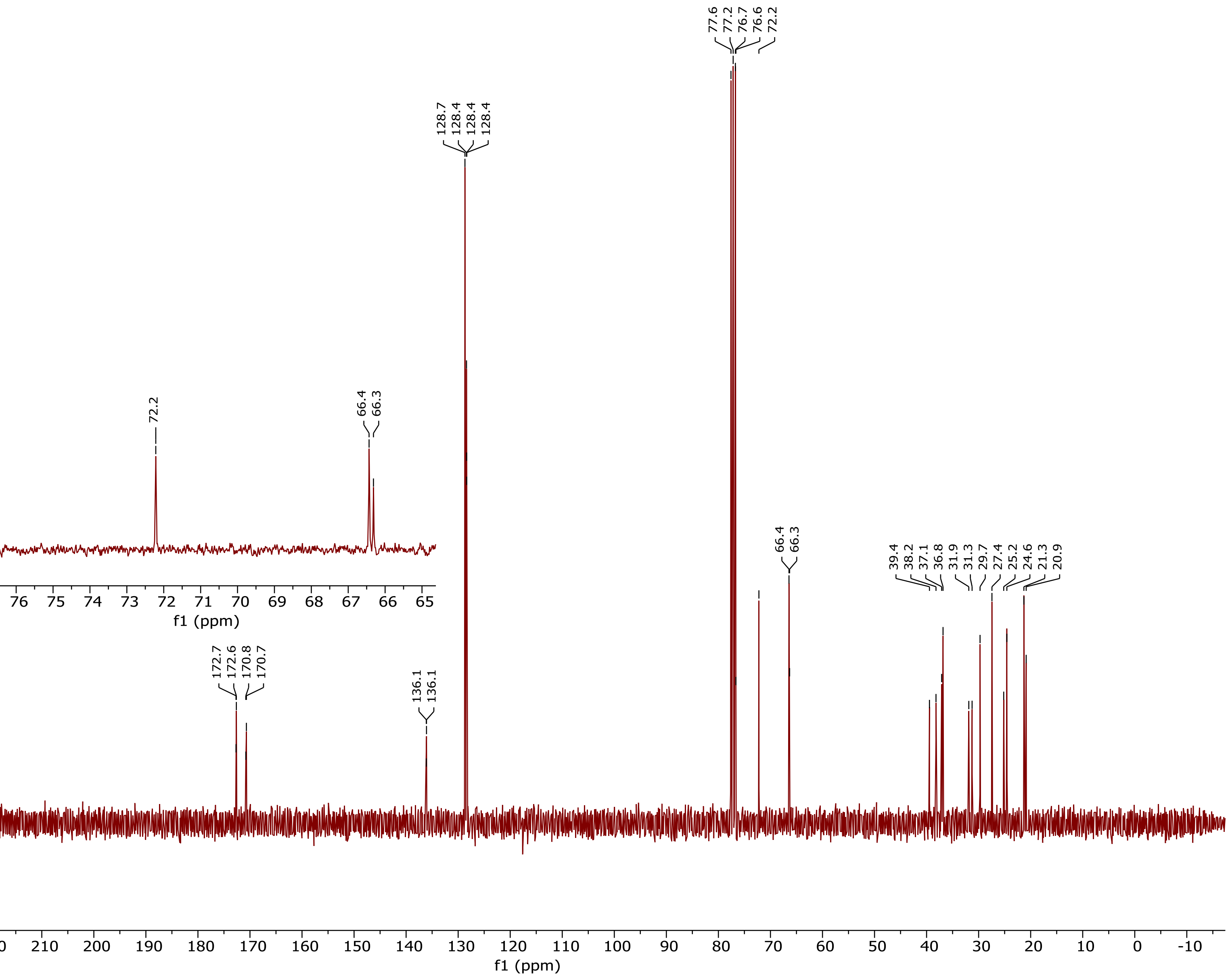

Benzyl 4-((diethoxyphosphoryl)oxy)butanoate (**16**)

^{1}H NMR (300 MHz, $CDCl_3$)

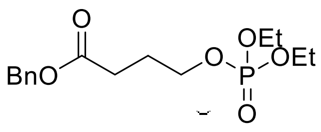

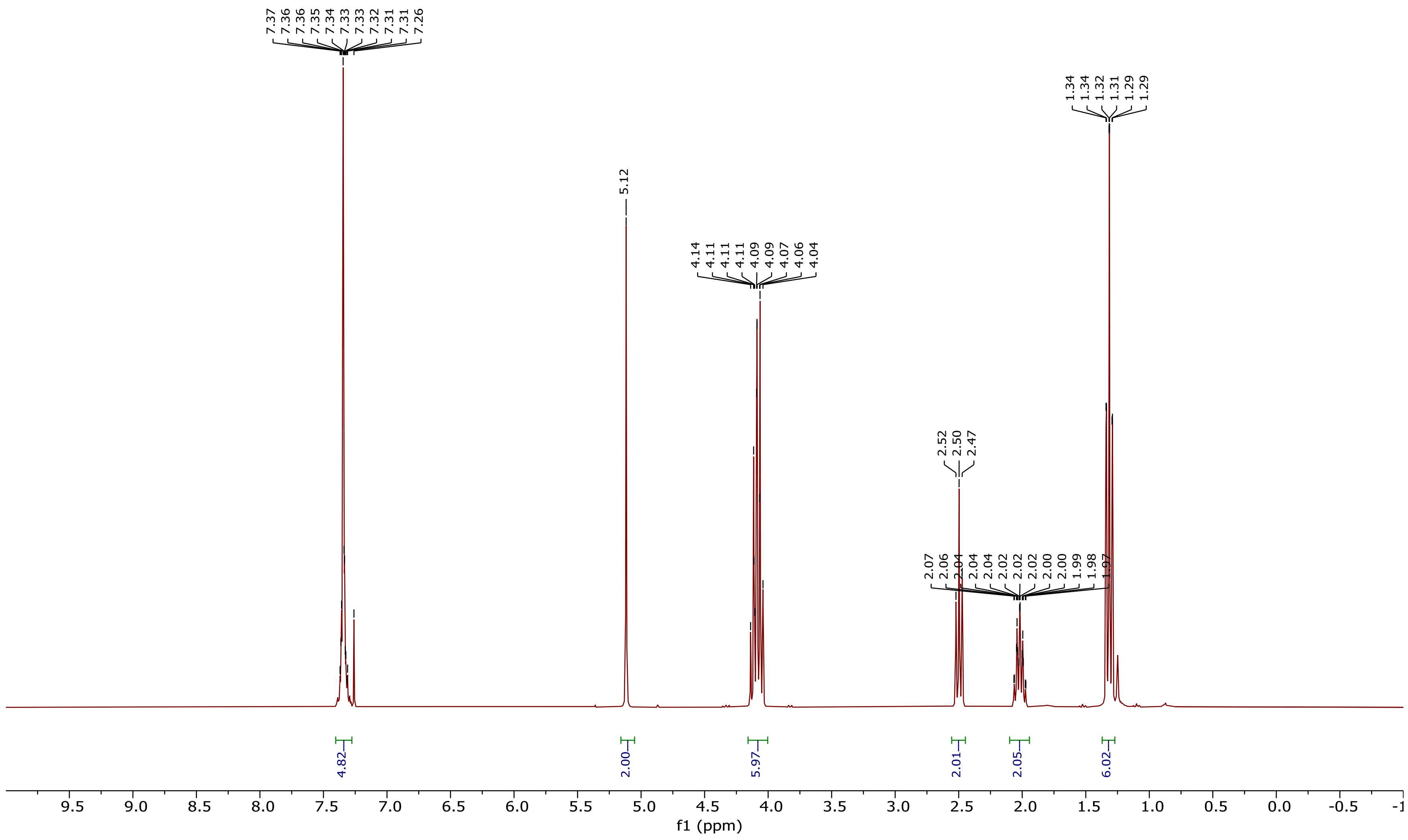

Benzyl 4-((diethoxyphosphoryl)oxy)butanoate (**16**)

^{13}C NMR (75 MHz, $CDCl_3$)

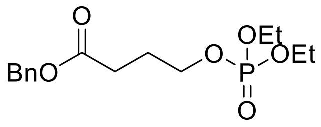

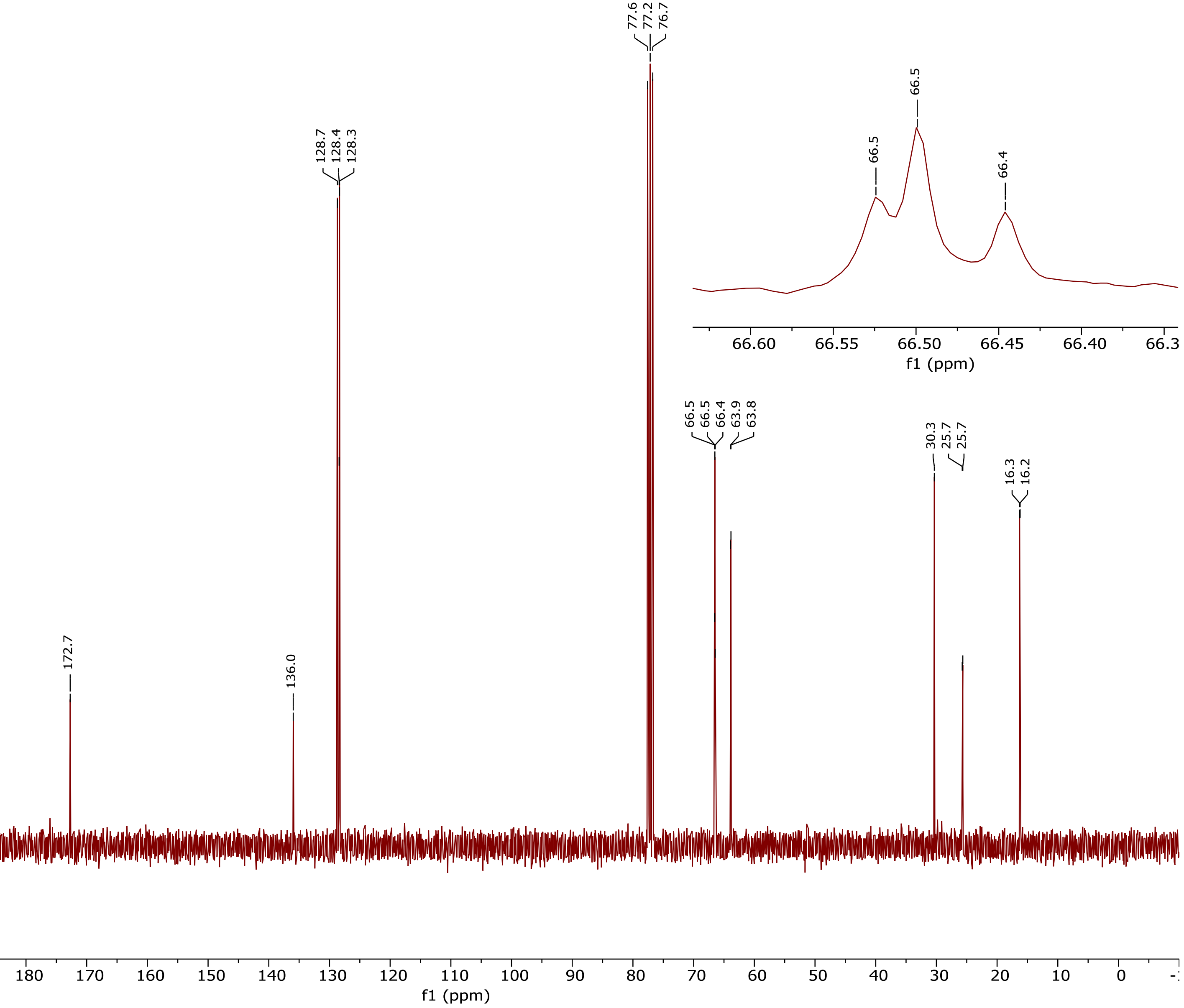

Ethyl 4-(phenylthio) butanoate (**18**)

^{1}H NMR (300 MHz, $CDCl_3$)

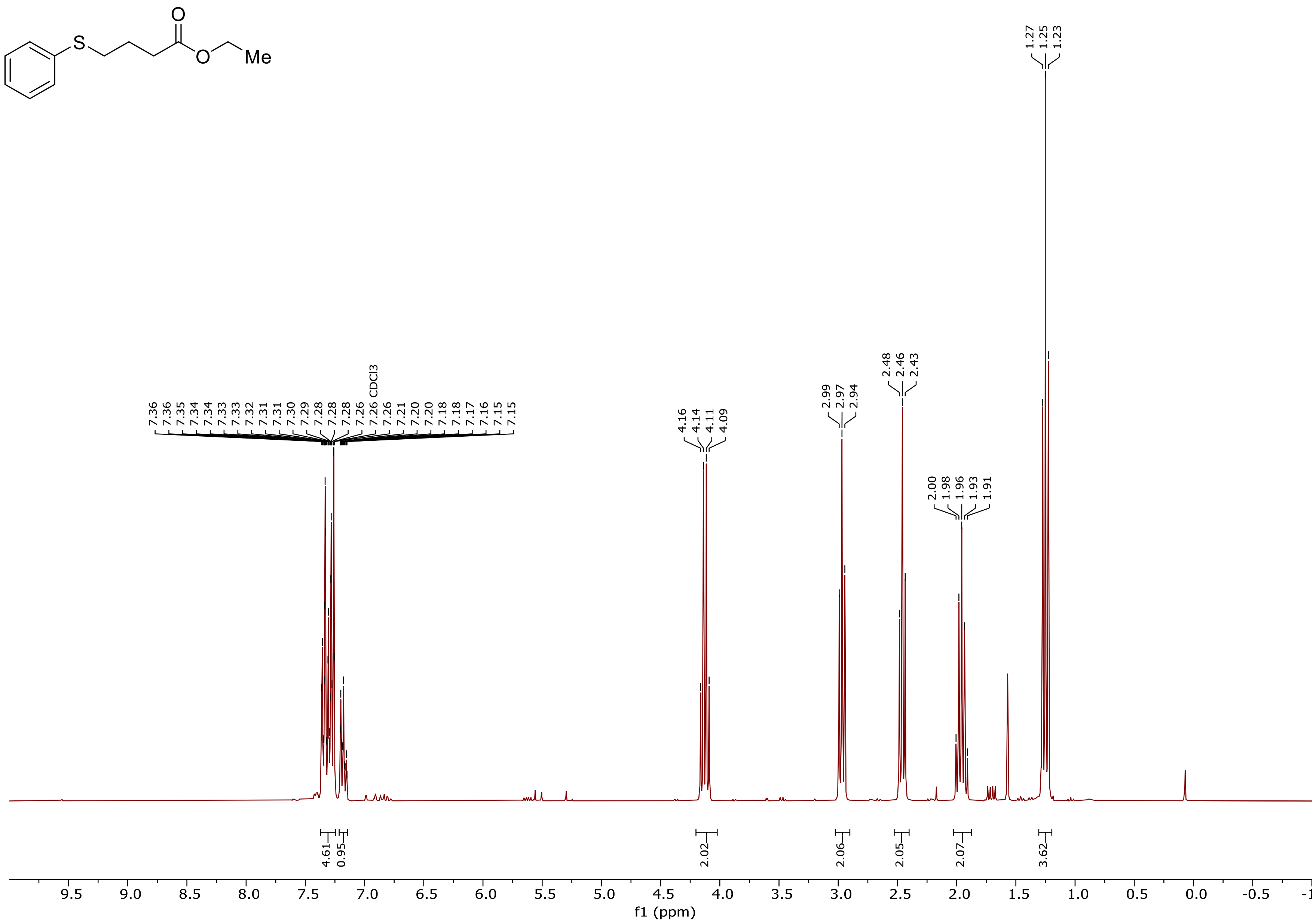

Ethyl 4-(phenylthio) butanoate (**18**)

^{13}C NMR (75 MHz, $CDCl_3$)

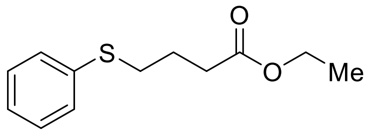

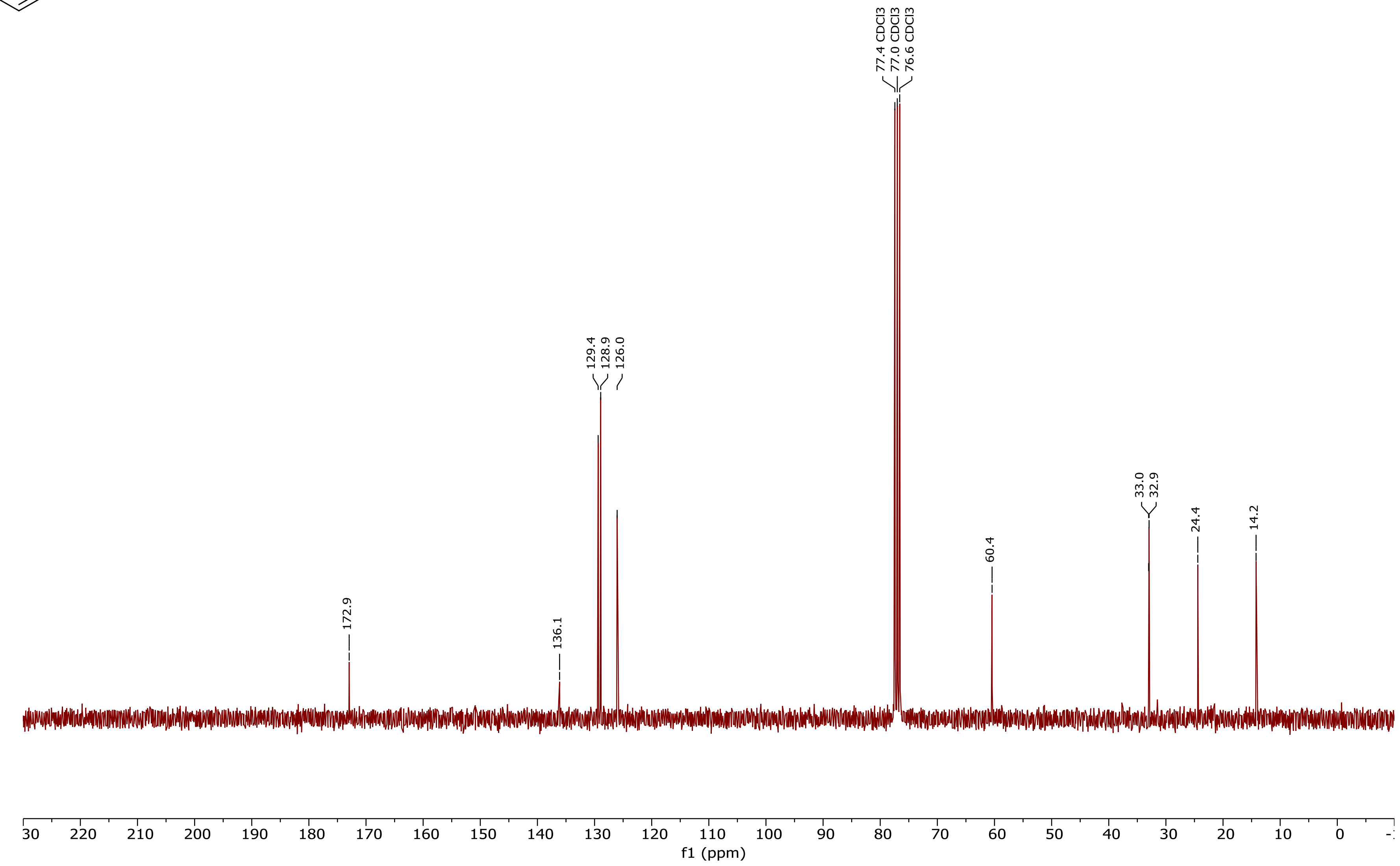

Benzyl 4-(phenylthio)butanoate (**19**)

^{1}H NMR (300 MHz, $CDCl_3$)

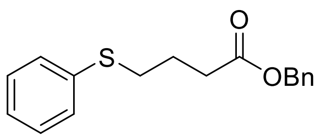

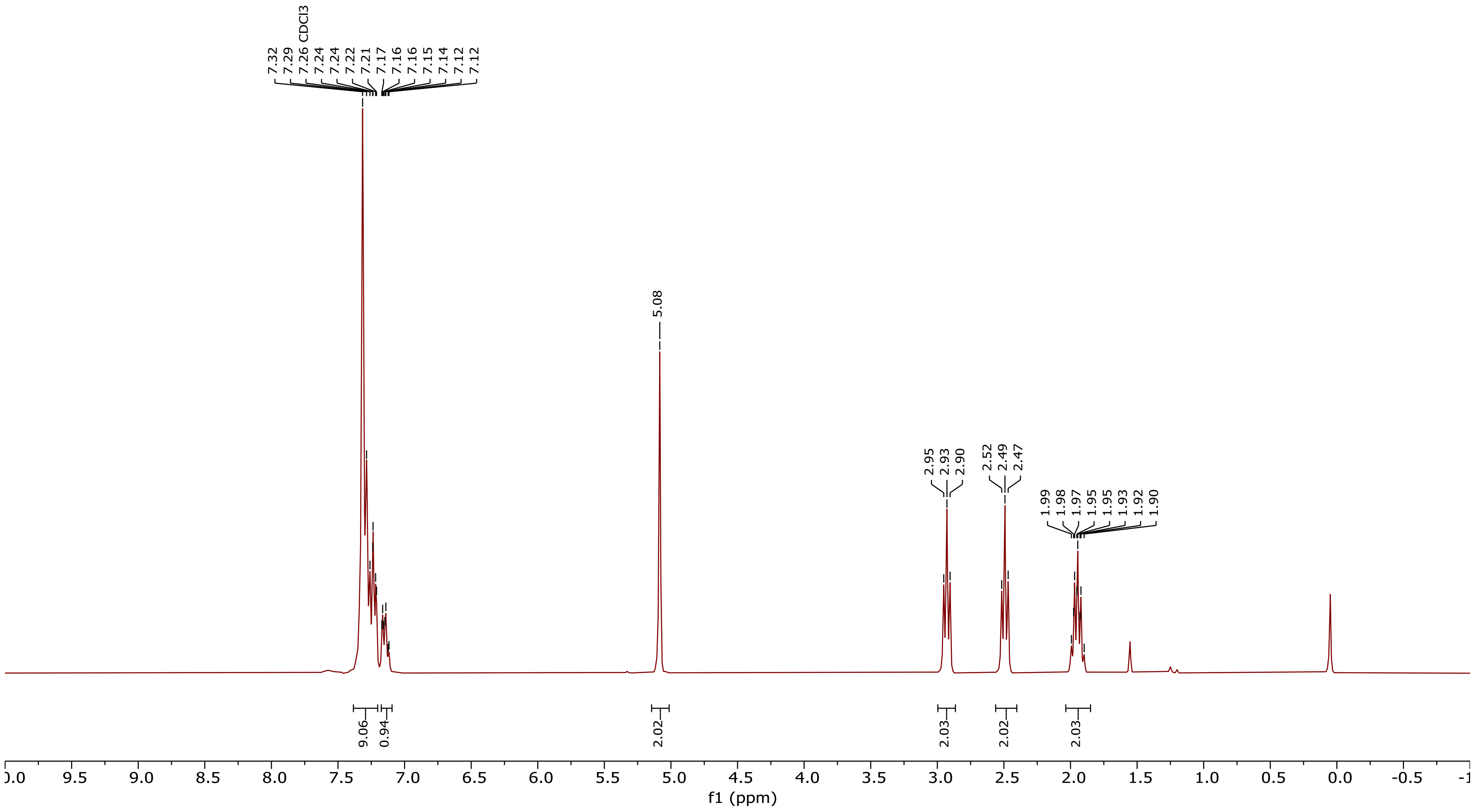

Benzyl 4-(phenylthio)butanoate (**19**)

^{13}C NMR (75 MHz, $CDCl_3$)

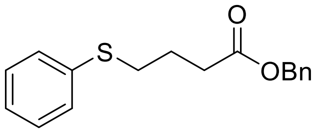

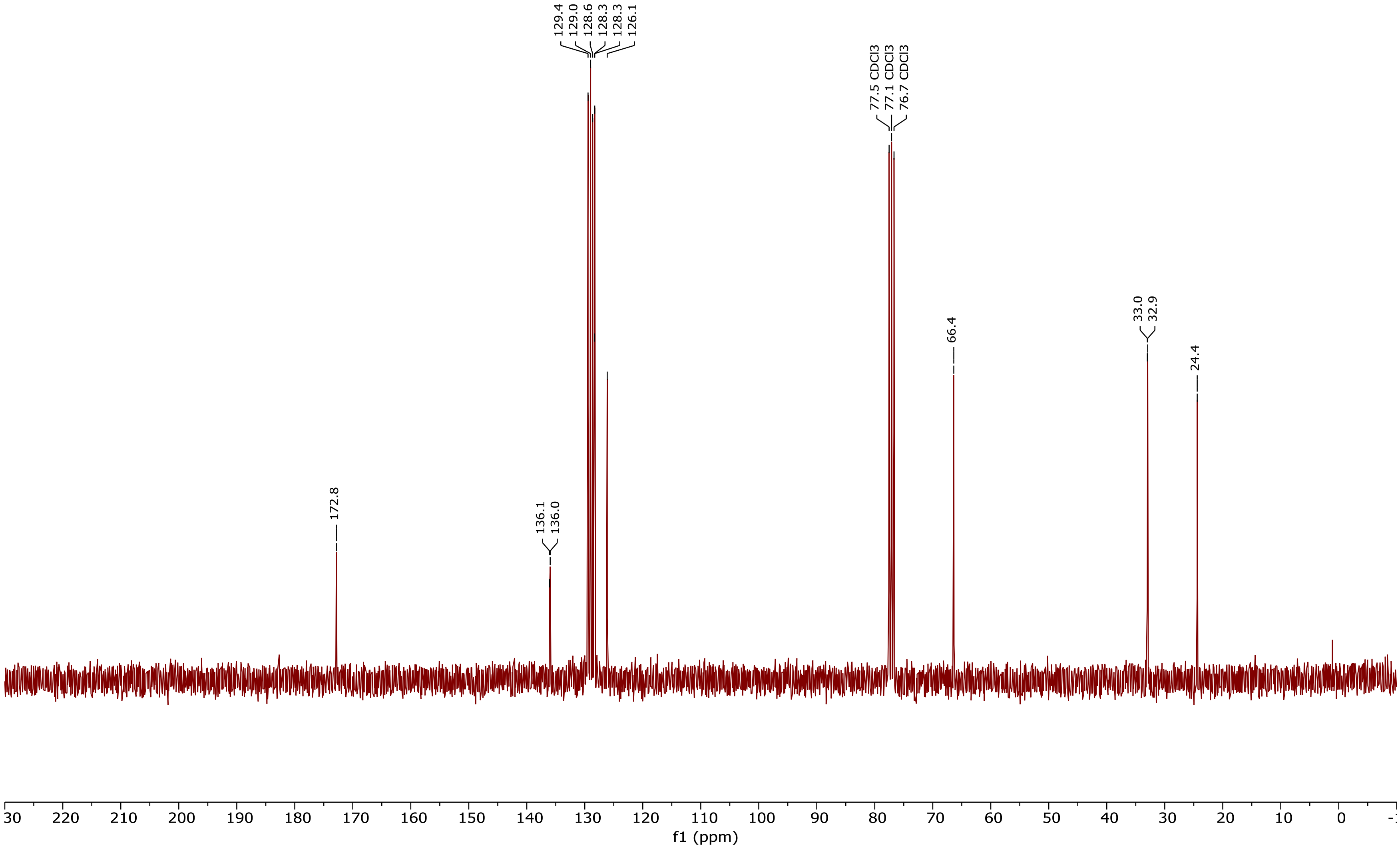

Phenyl 4-(phenylthio) butanoate (**20**)

^{1}H NMR (300 MHz, $CDCl_3$)

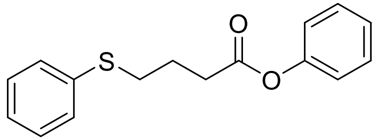

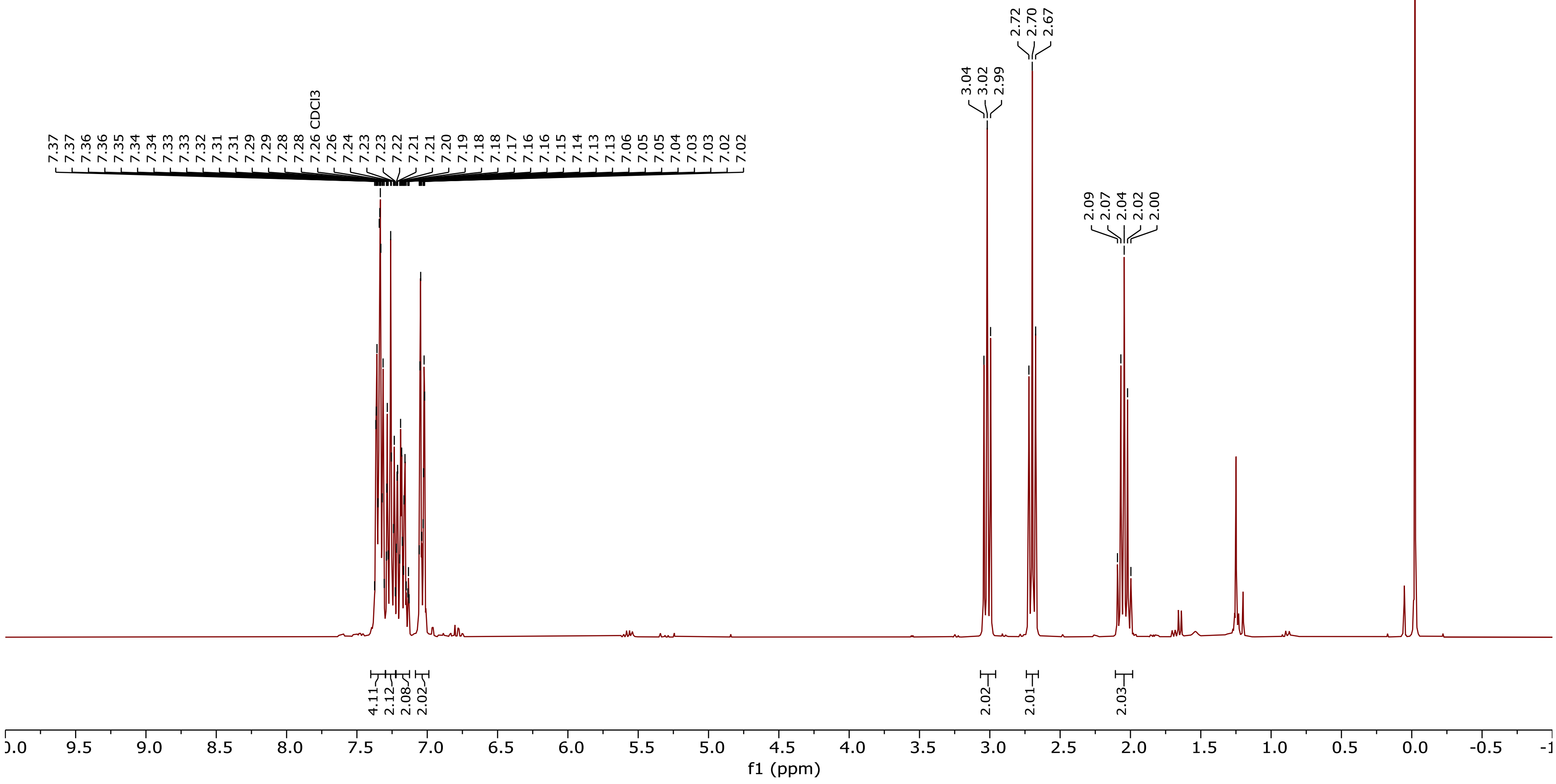

Phenyl 4-(phenylthio) butanoate (**20**)

^{13}C NMR (75 MHz, $CDCl_3$)

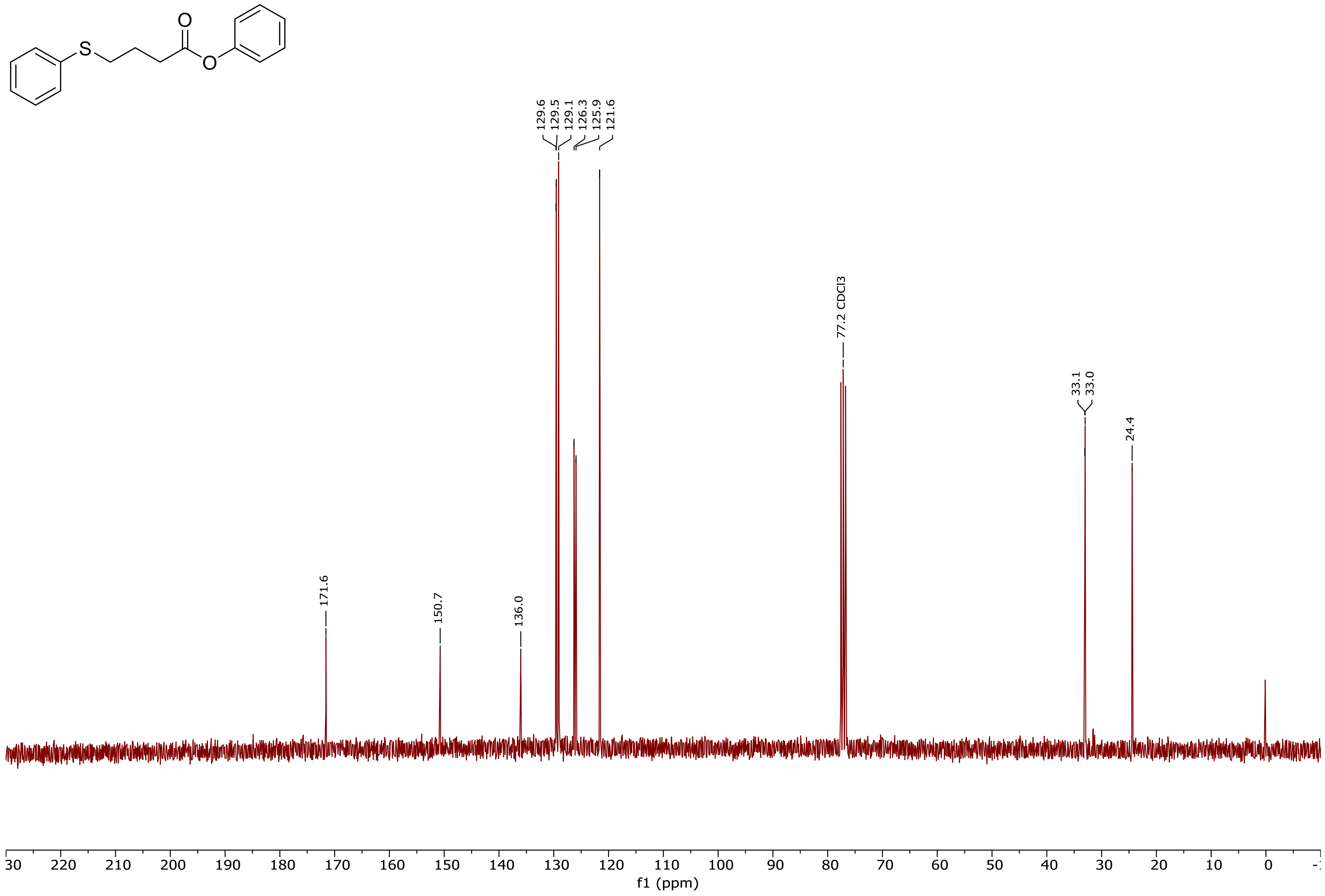

Ethyl 2-methyl-4-(phenylthio)butanoate (**21**)

^{1}H NMR (300 MHz, $CDCl_3$)

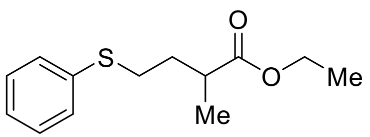

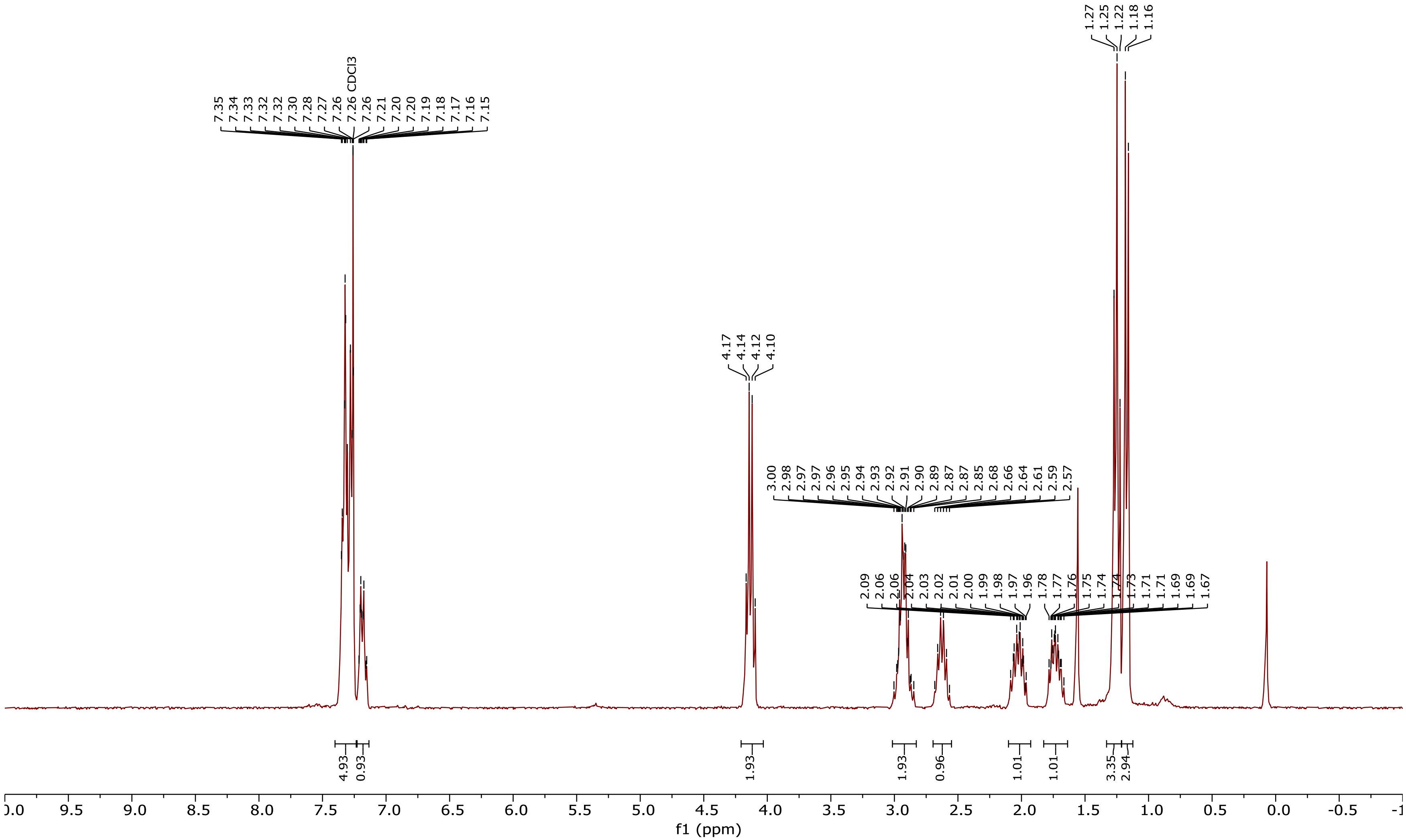

Ethyl 2-methyl-4-(phenylthio)butanoate (**21**)

^{13}C NMR (75 MHz, $CDCl_3$)

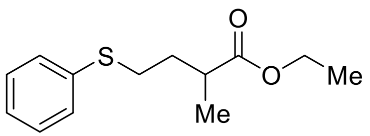

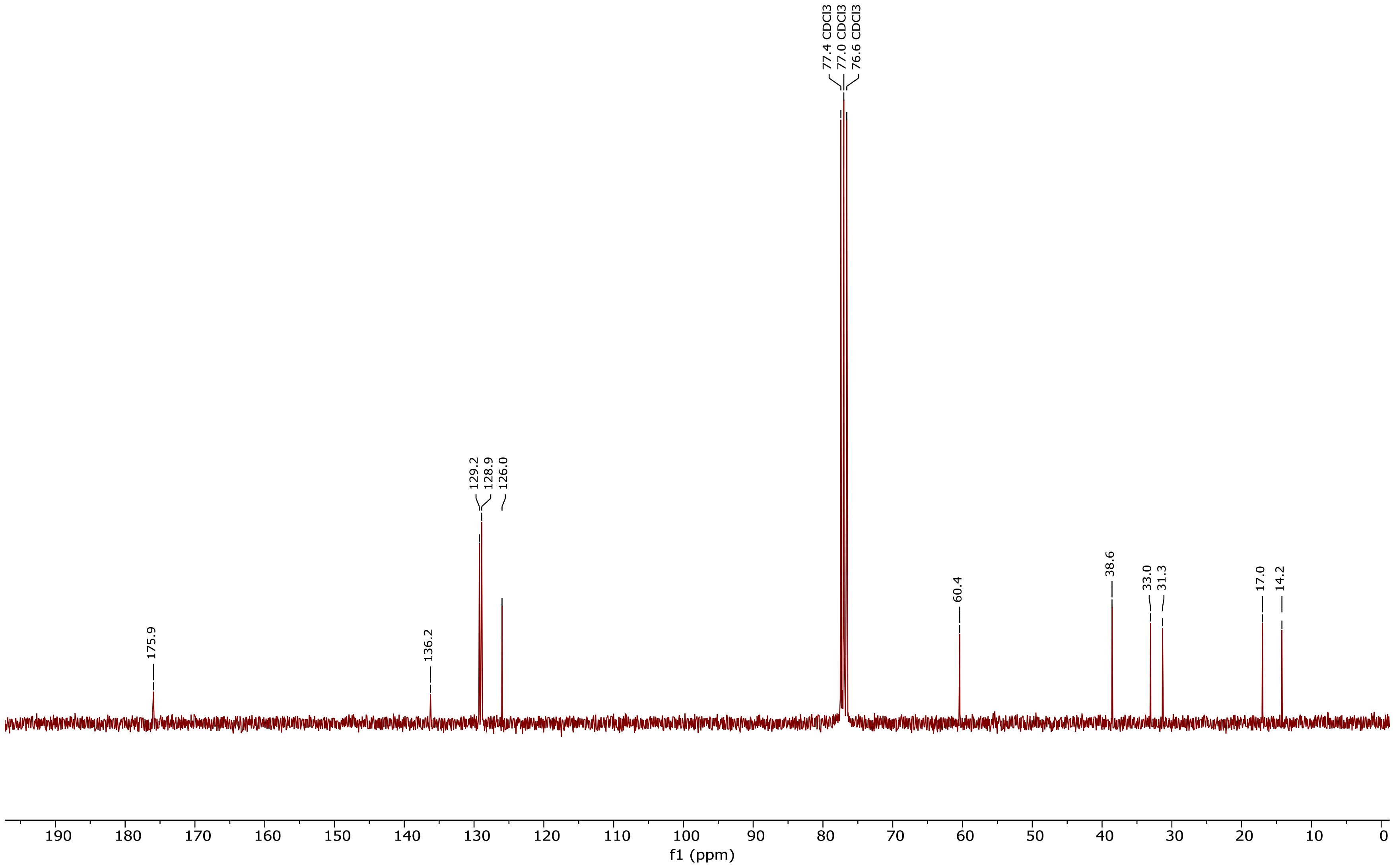

3-(2-(Phenylthio)ethyl)dihydrofuran-2(3*H*)-one (**22**)

^{1}H NMR (300 MHz, $CDCl_3$)

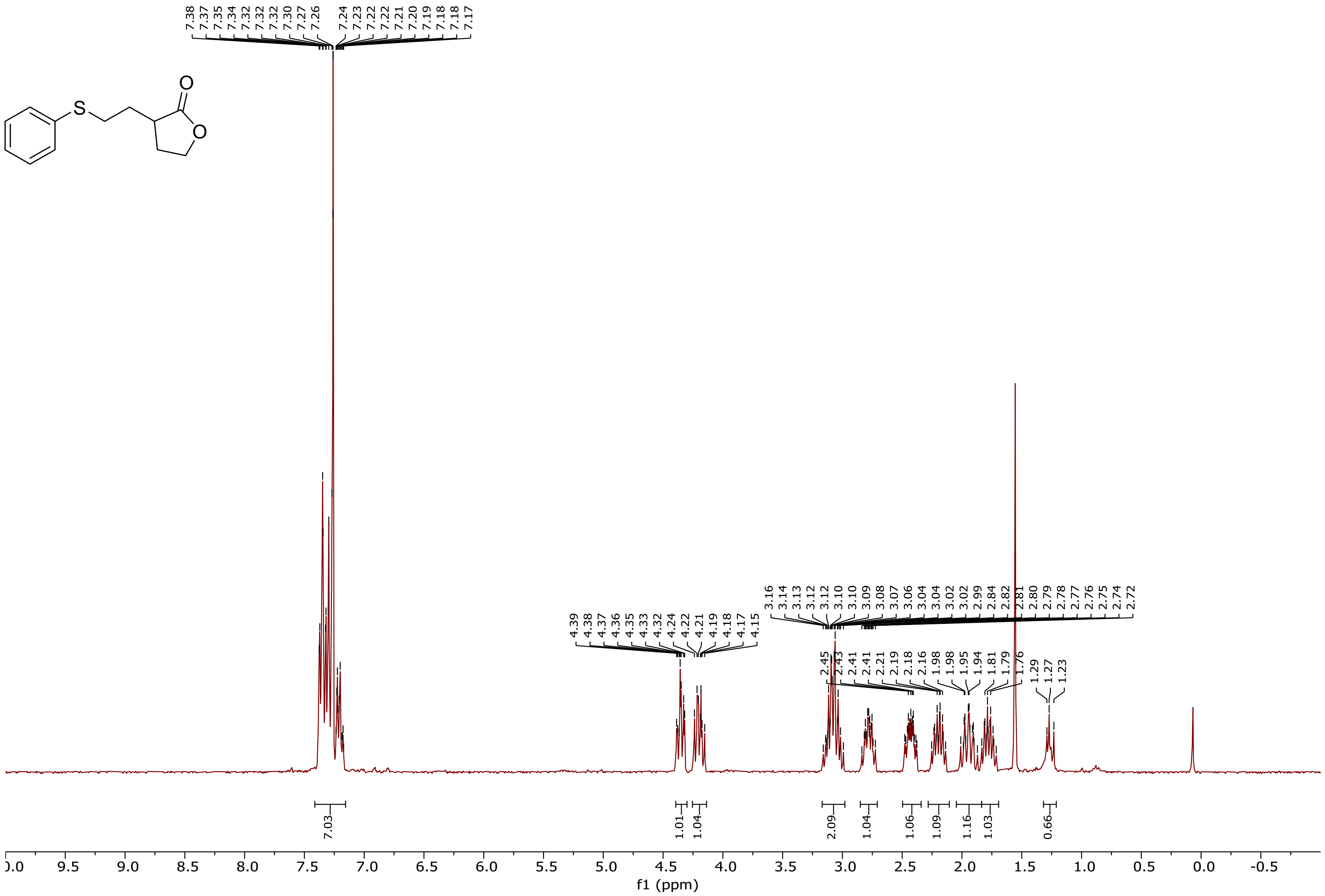

3-(2-(Phenylthio)ethyl)dihydrofuran-2(3*H*)-one (**22**)

^{13}C NMR (75 MHz, $CDCl_3$)

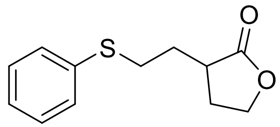

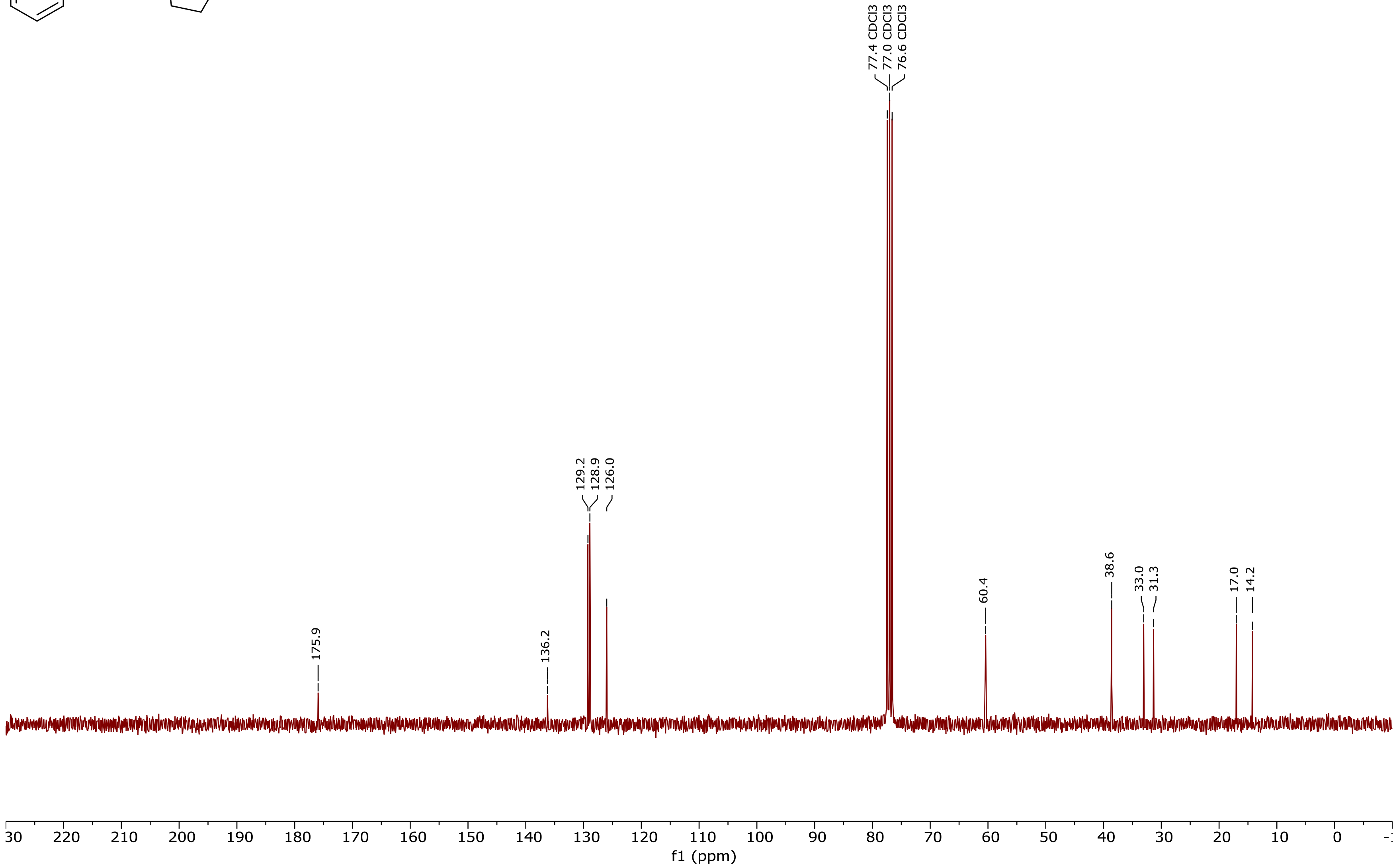

Ethyl 2,2-difluoro-4-(phenylthio)butanoate (**23**)

^{1}H NMR (300 MHz, $CDCl_3$)

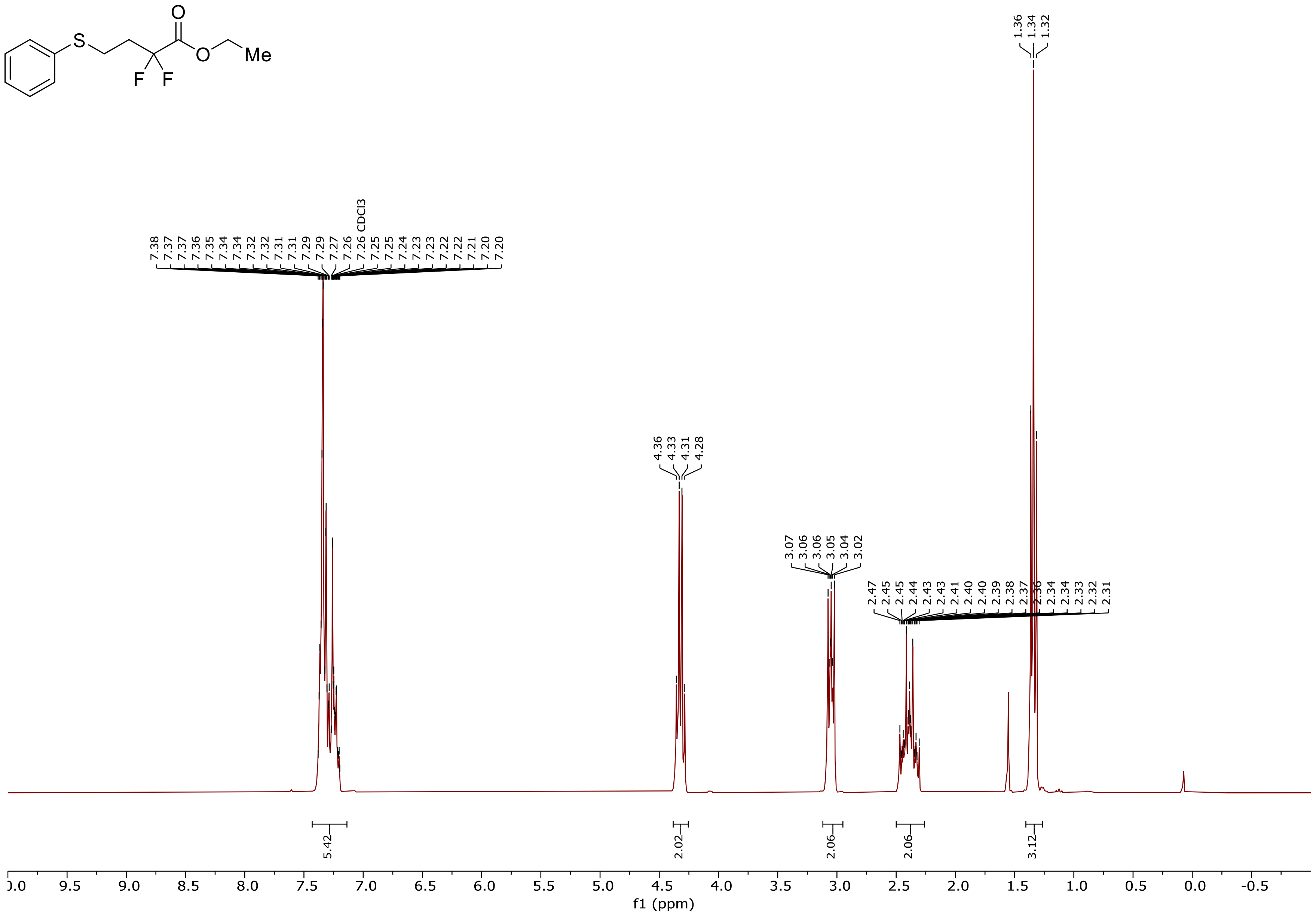

Ethyl 2,2-difluoro-4-(phenylthio)butanoate (**23**)

^{13}C NMR (75 MHz, $CDCl_3$)

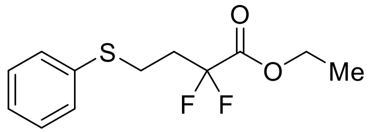

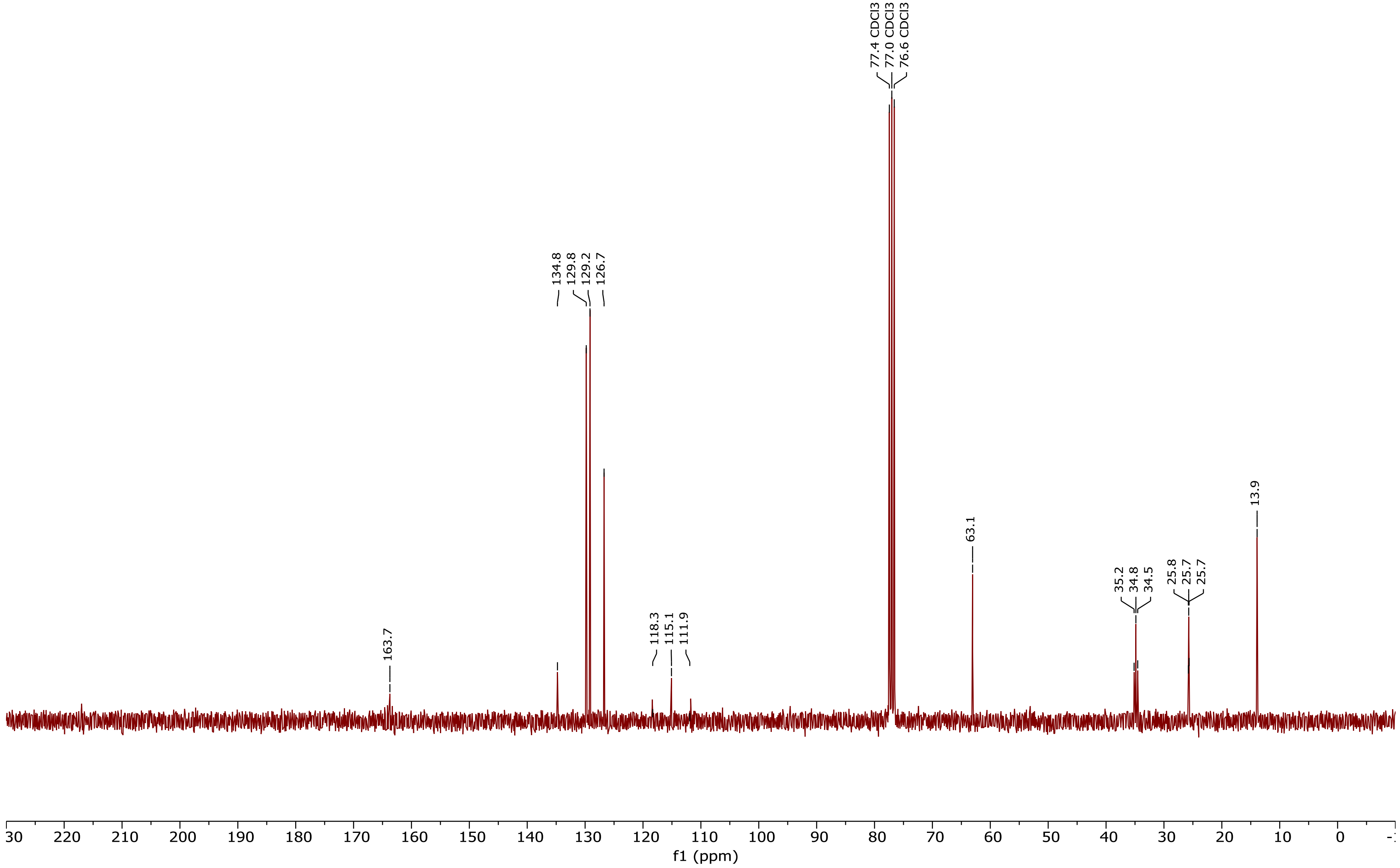

Diethyl 2-(2-(phenylthio)ethyl) malonate (**24**)

^{1}H NMR (300 MHz, $CDCl_3$)

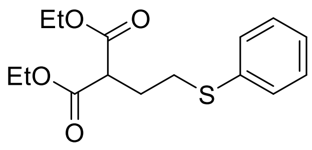

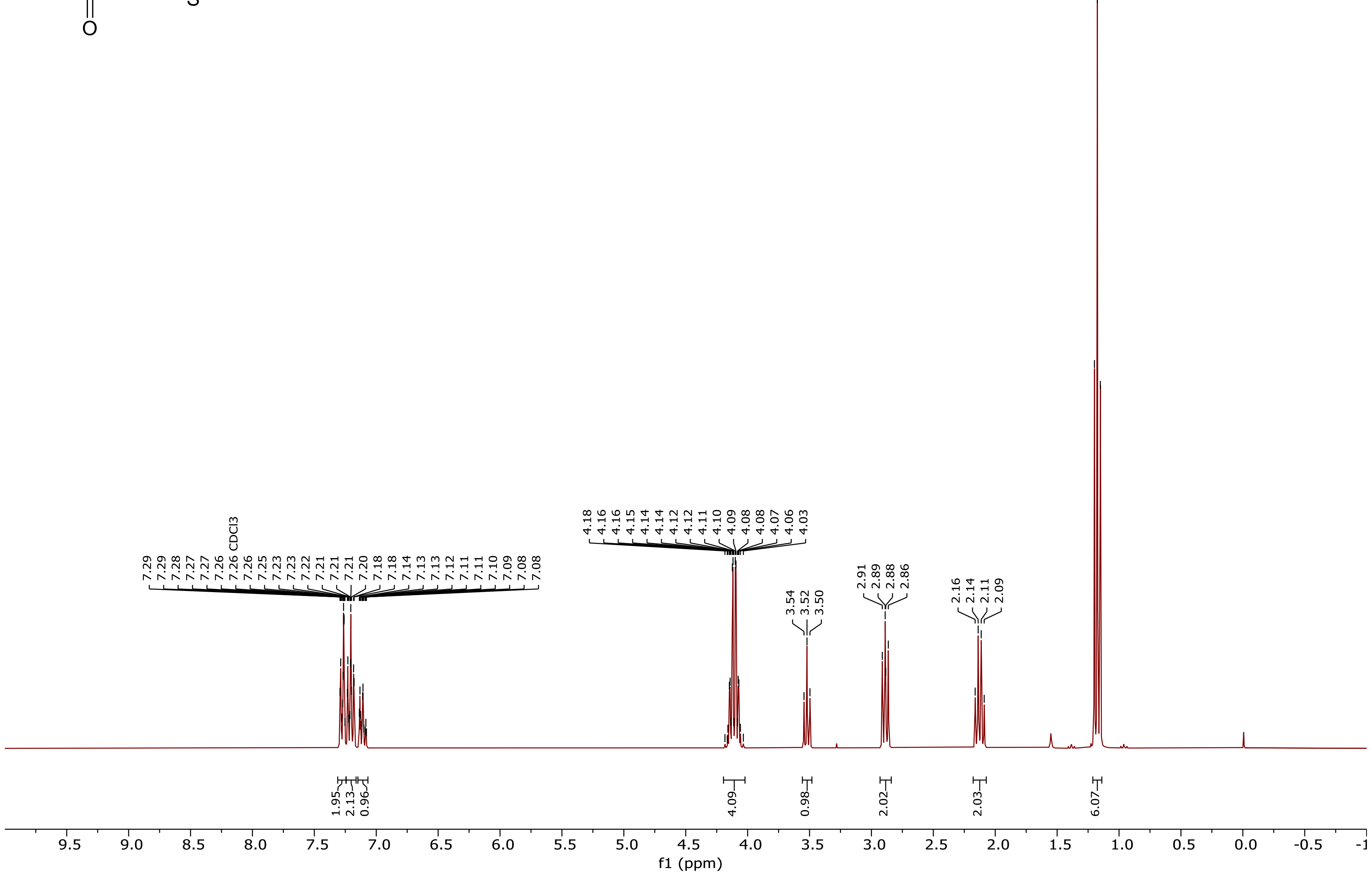

Diethyl 2-(2-(phenylthio)ethyl) malonate (**24**)

^{13}C NMR (75 MHz, $CDCl_3$)

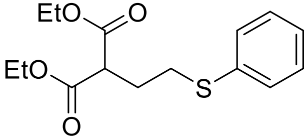

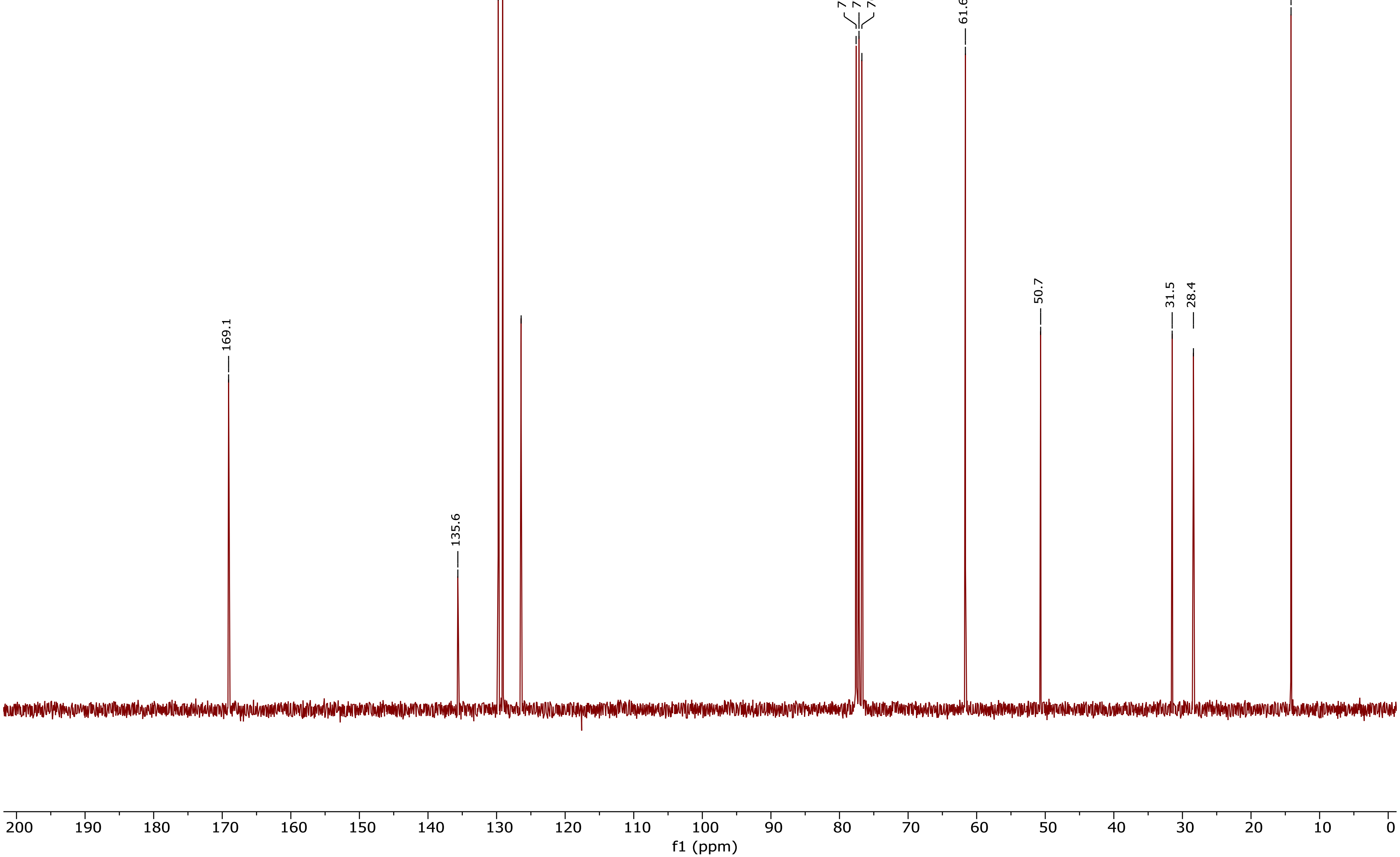

Benzyl 4-(phenylthio)pentanoate (**25**)

^{1}H NMR (300 MHz, $CDCl_3$)

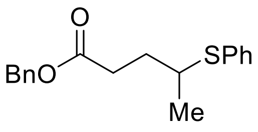

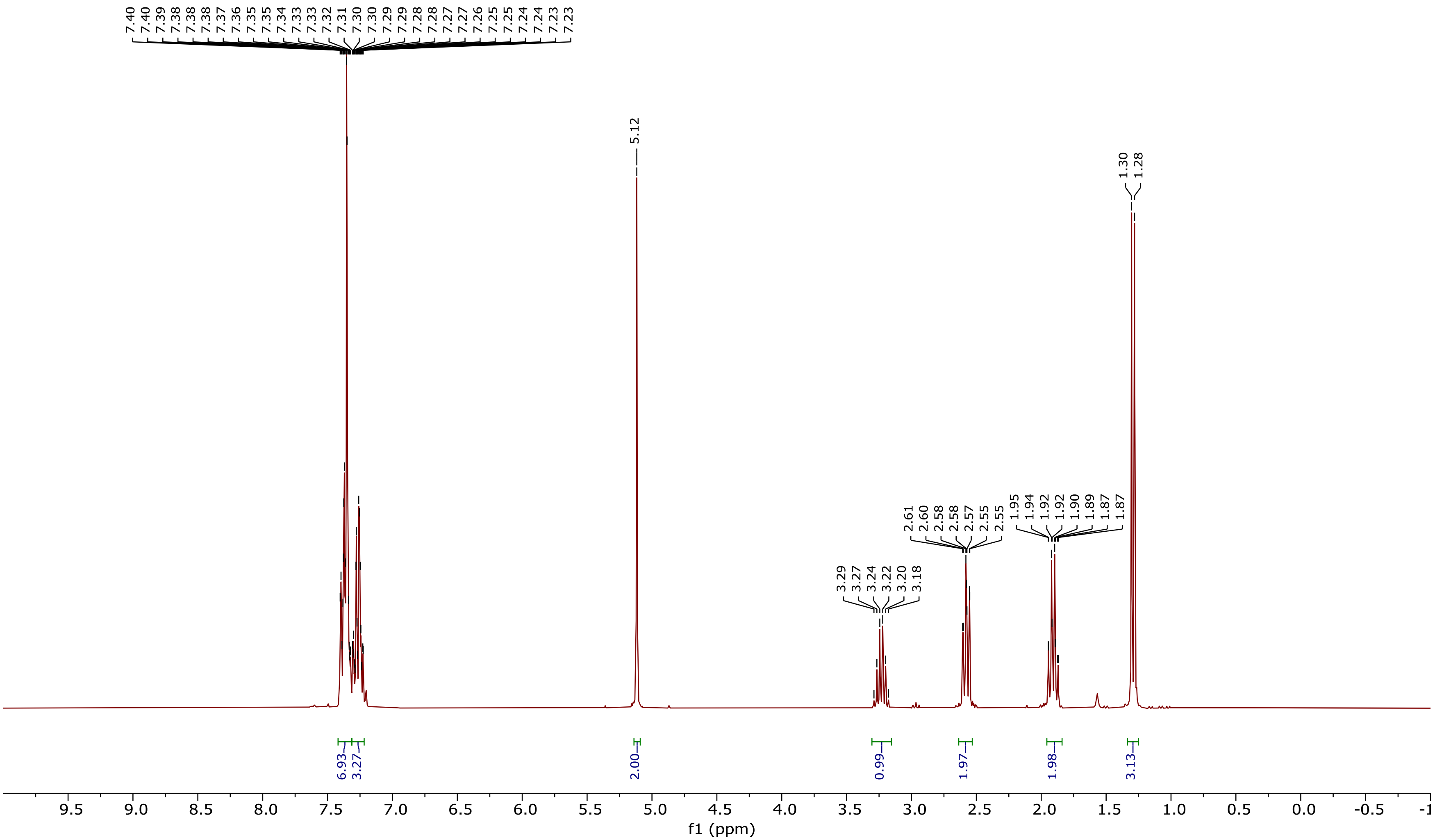

Benzyl 4-(phenylthio)pentanoate (**25**)

^{13}C NMR (75 MHz, $CDCl_3$)

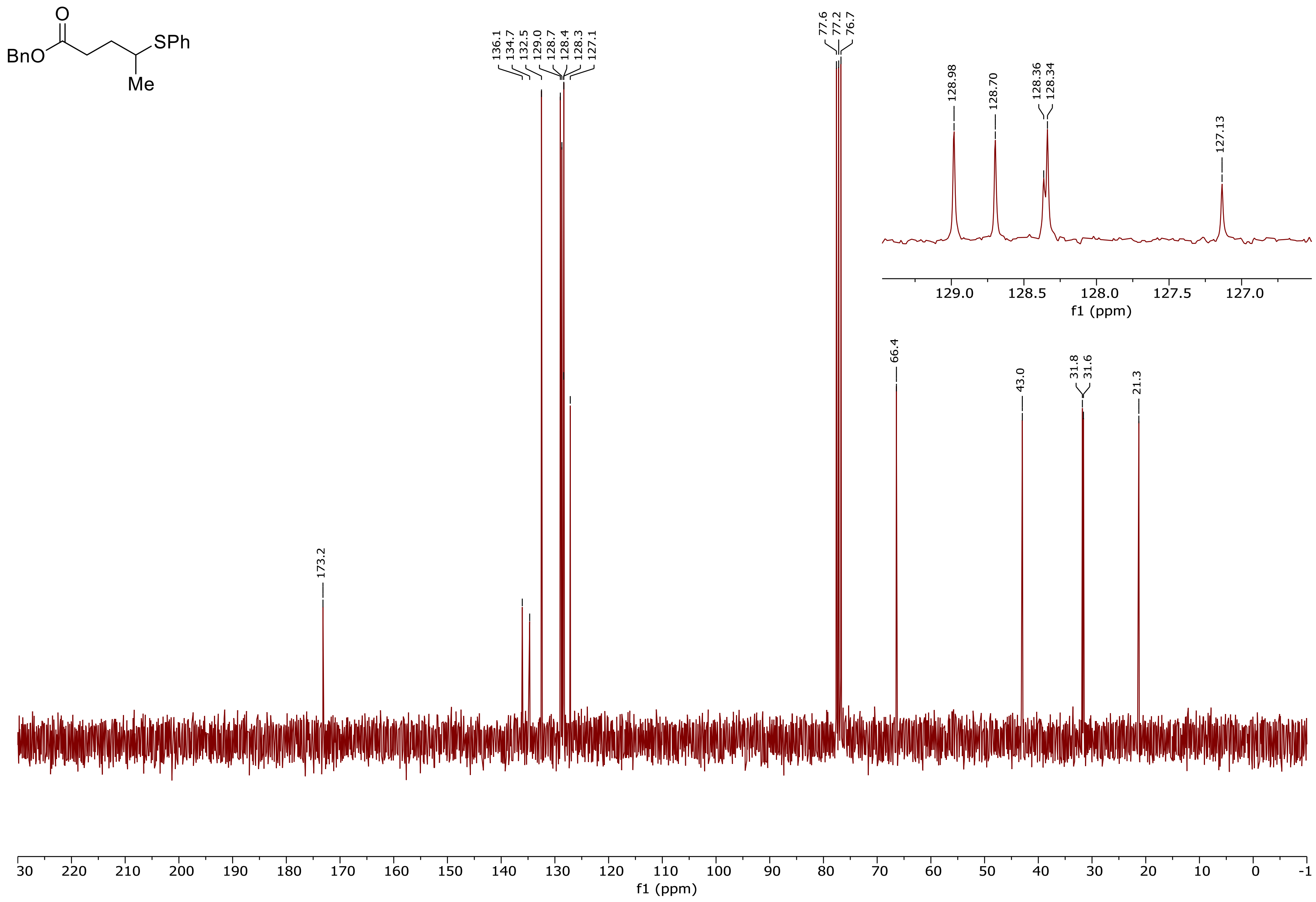

Benzyl 4-butoxybutanoate (**27**)

^{1}H NMR (300 MHz, $CDCl_3$)

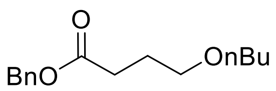

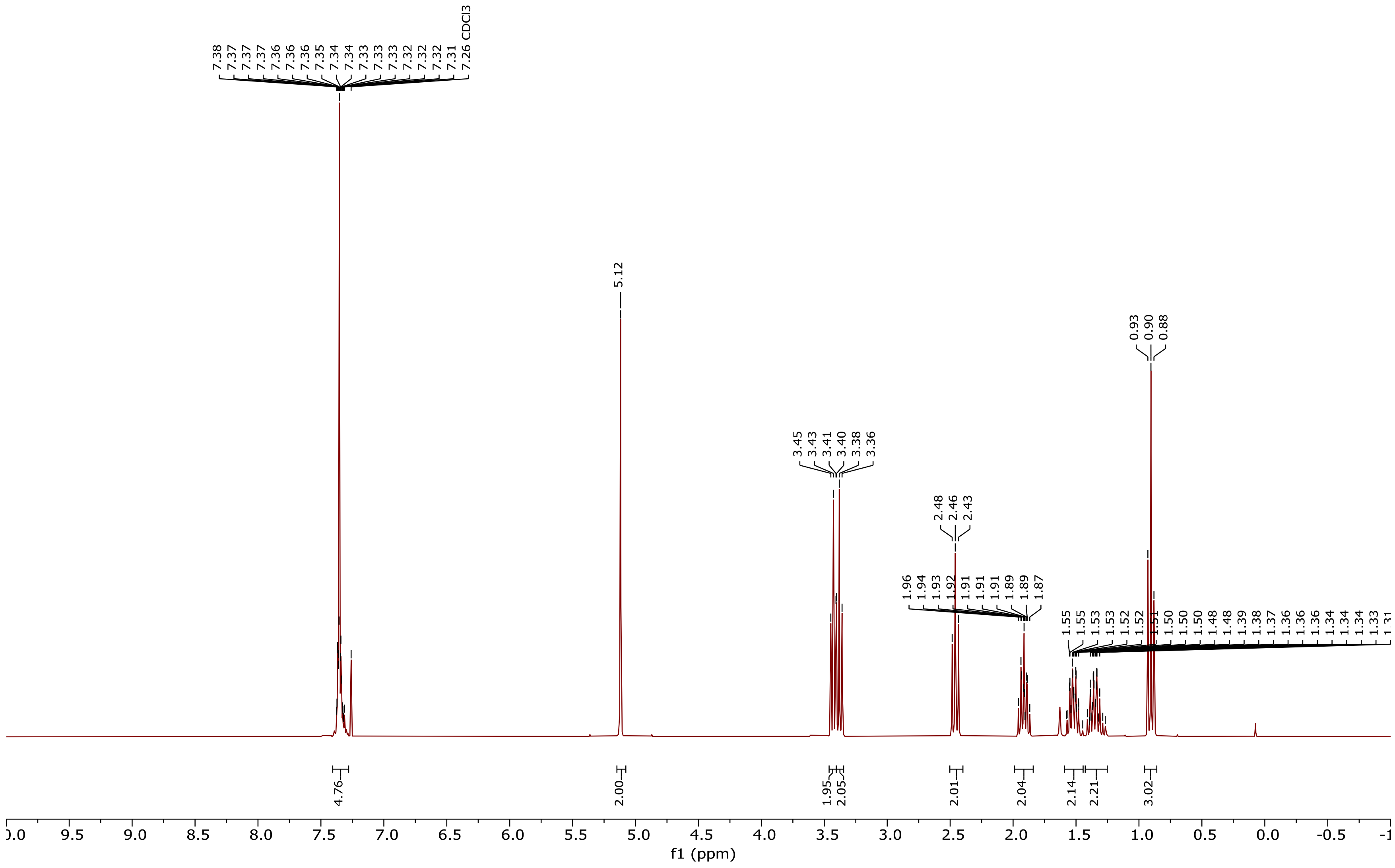

Benzyl 4-butoxybutanoate (**27**)

^{13}C NMR (75 MHz, $CDCl_3$)

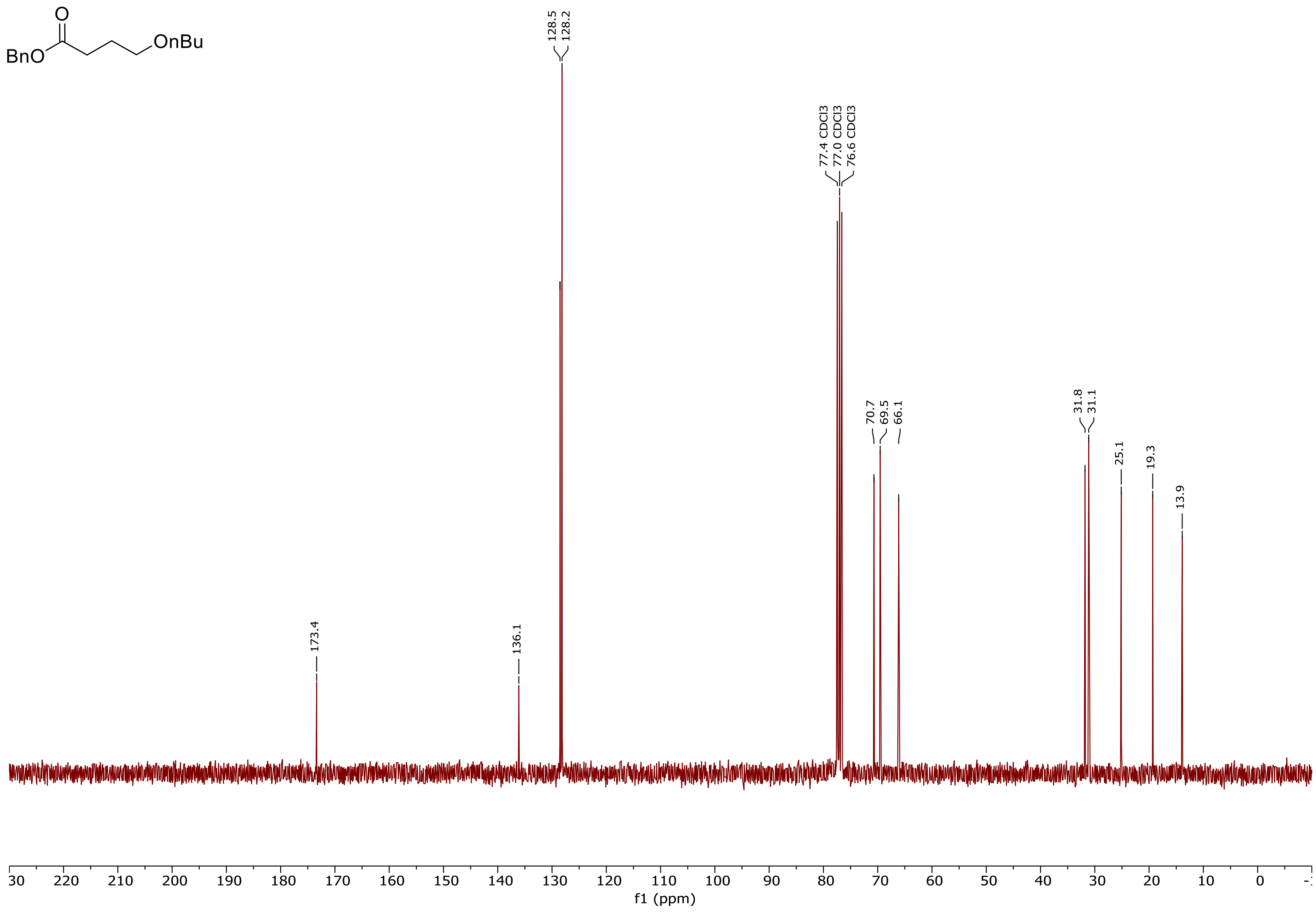

Diethyl 2-(2-butoxyethyl)malonate (**28**)

^{1}H NMR (300 MHz, $CDCl_3$)

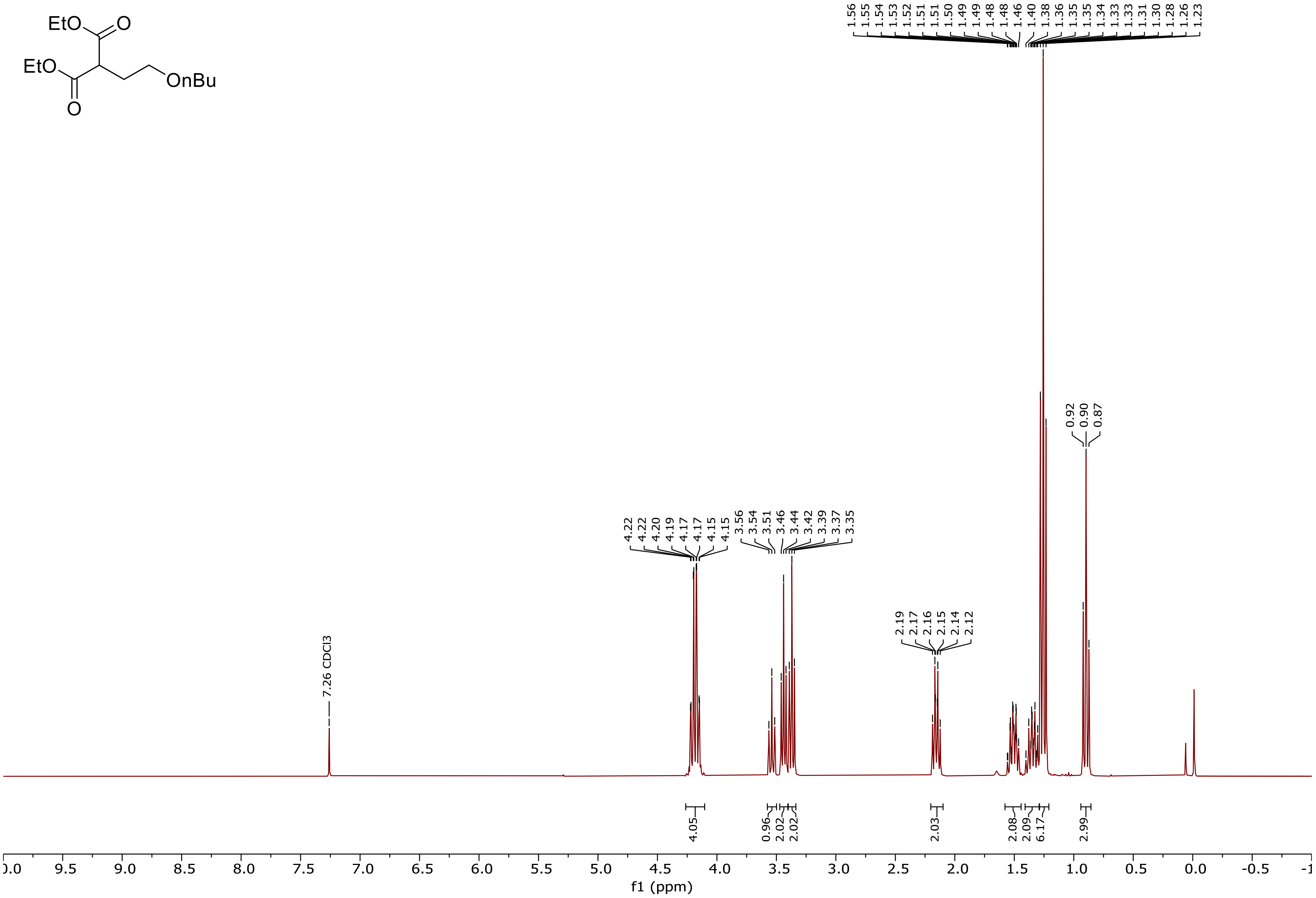

Diethyl 2-(2-butoxyethyl)malonate (**28**)

^{13}C NMR (75 MHz, $CDCl_3$)

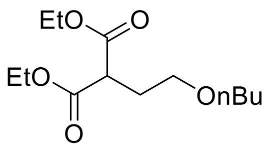

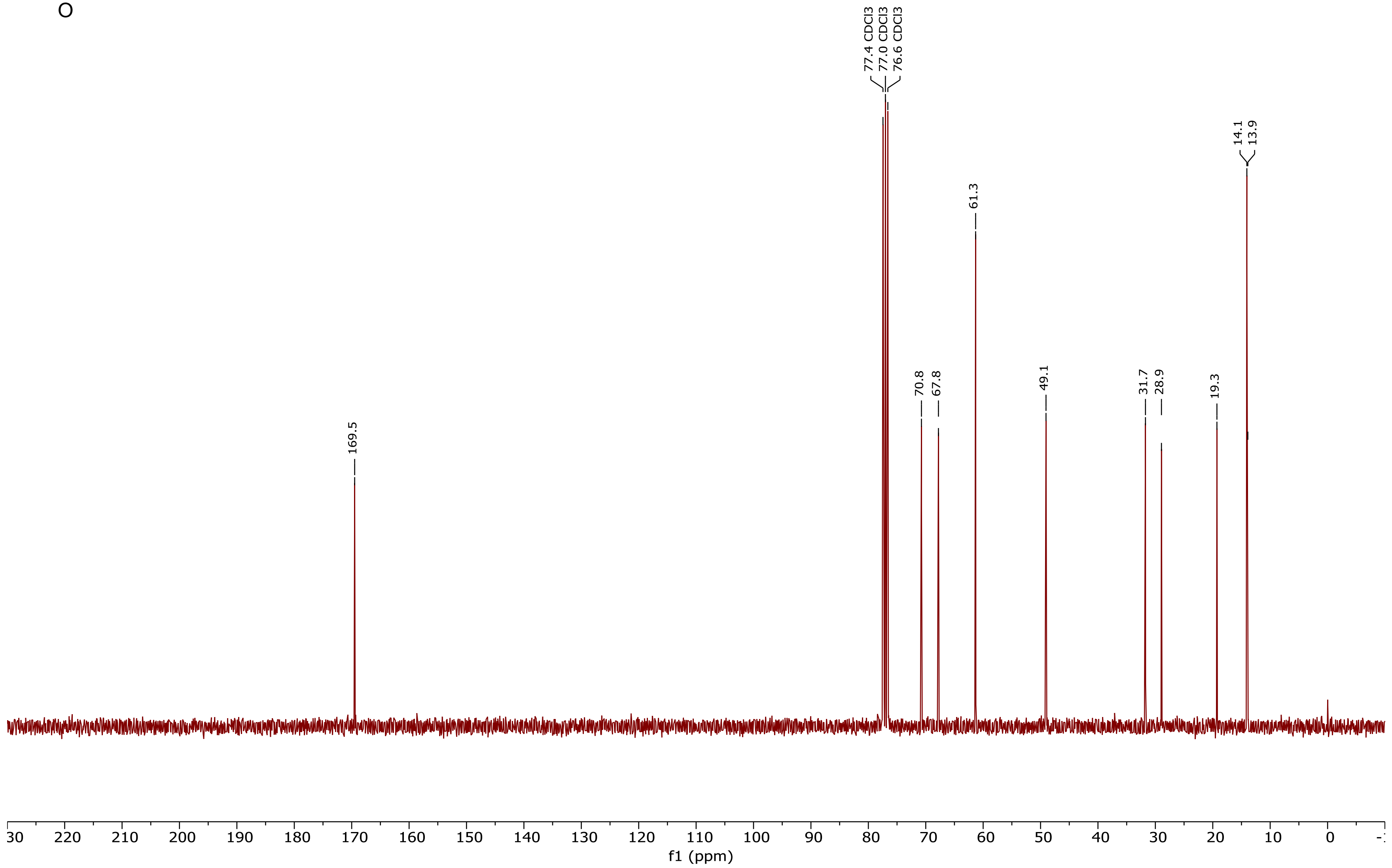

Diethyl 2-(2-butoxyethyl)-2-methylmalonate (**29**)

^{1}H NMR (300 MHz, $CDCl_3$)

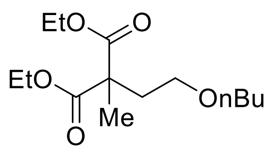

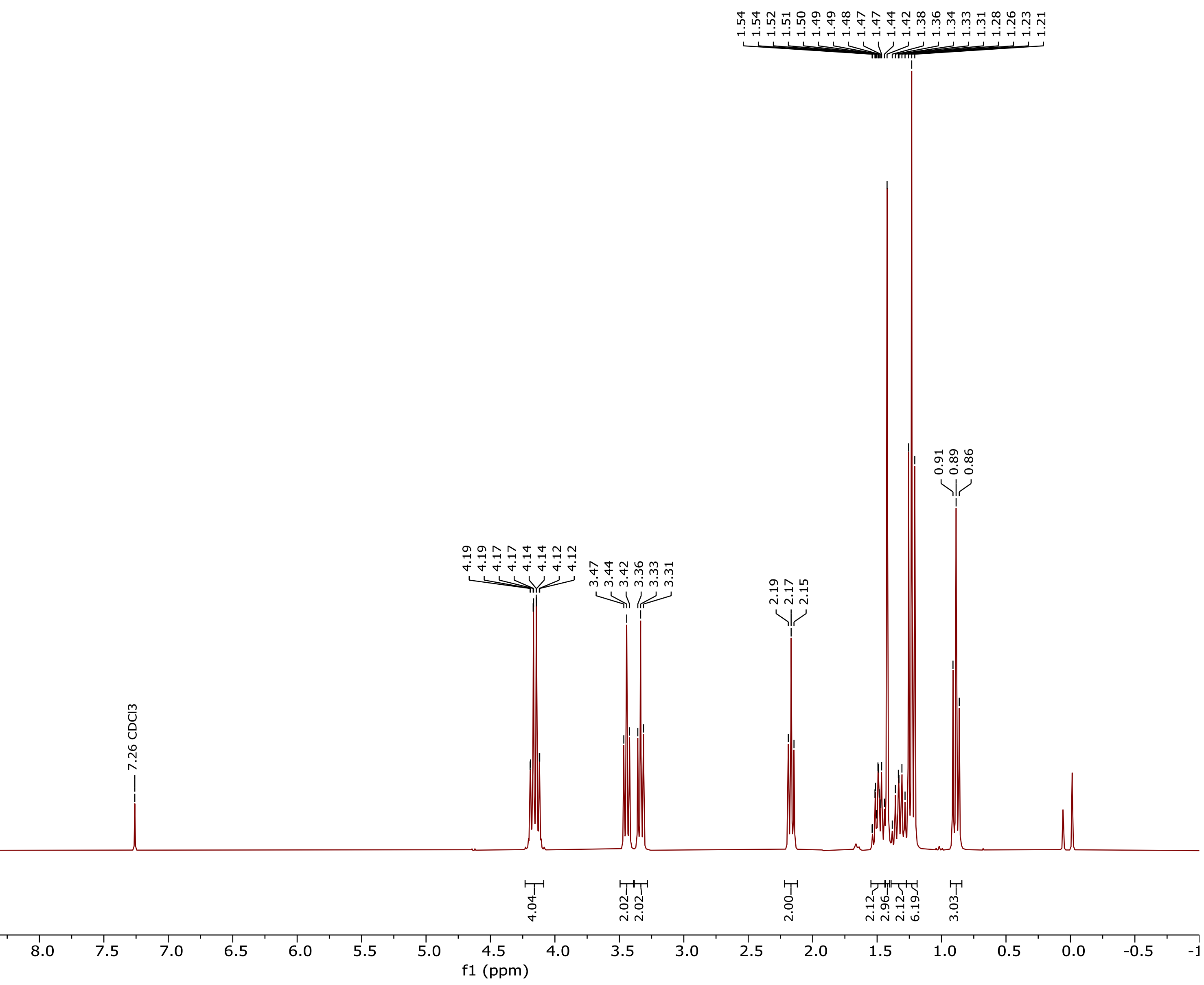

Diethyl 2-(2-butoxyethyl)-2-methylmalonate (**29**)

^{13}C NMR (75 MHz, $CDCl_3$)

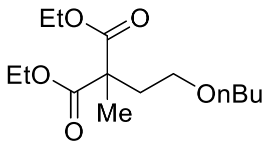

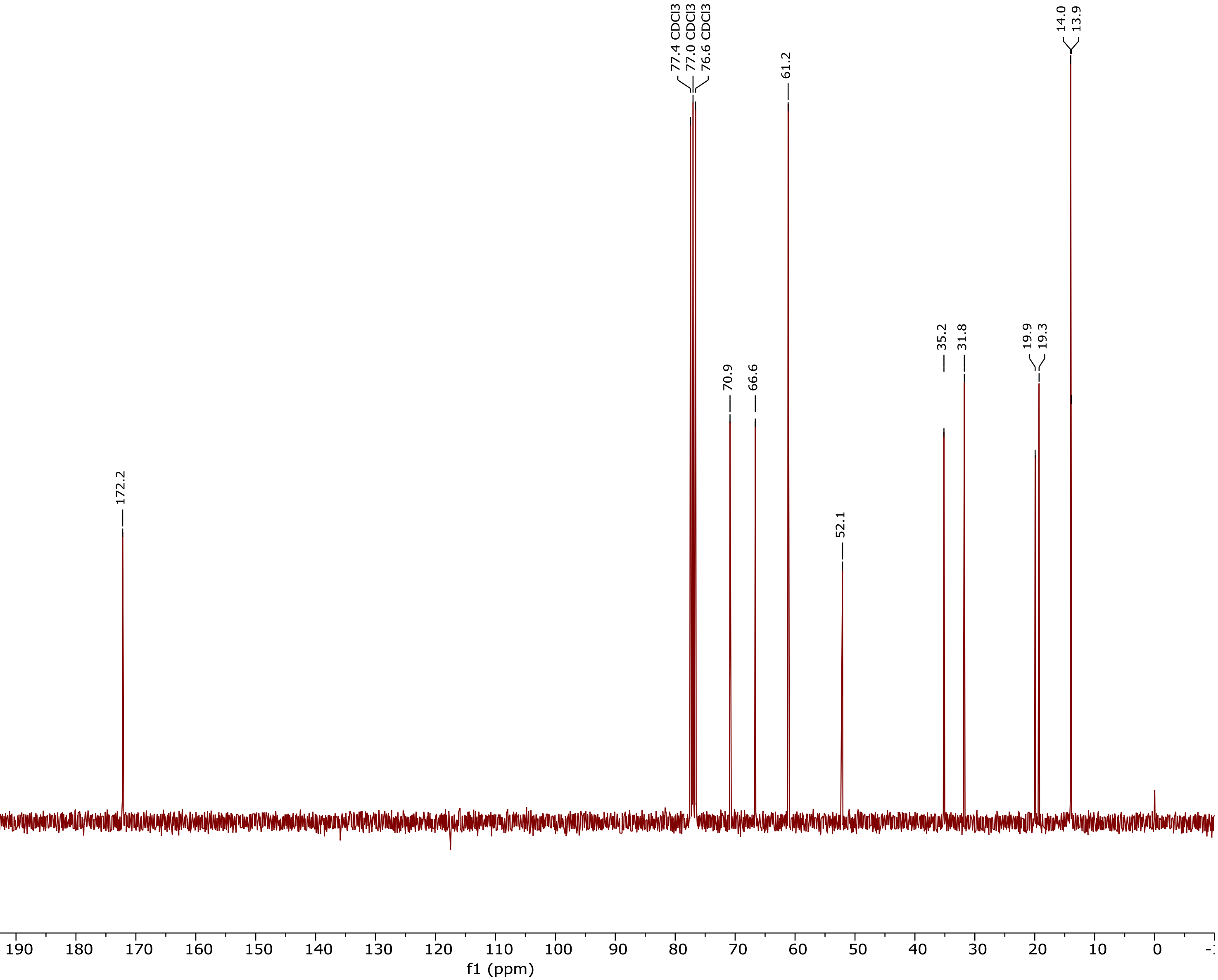

Cyclohex-2-enyl 4-butoxybutanoate (**30**)

^{1}H NMR (300 MHz, $CDCl_3$)

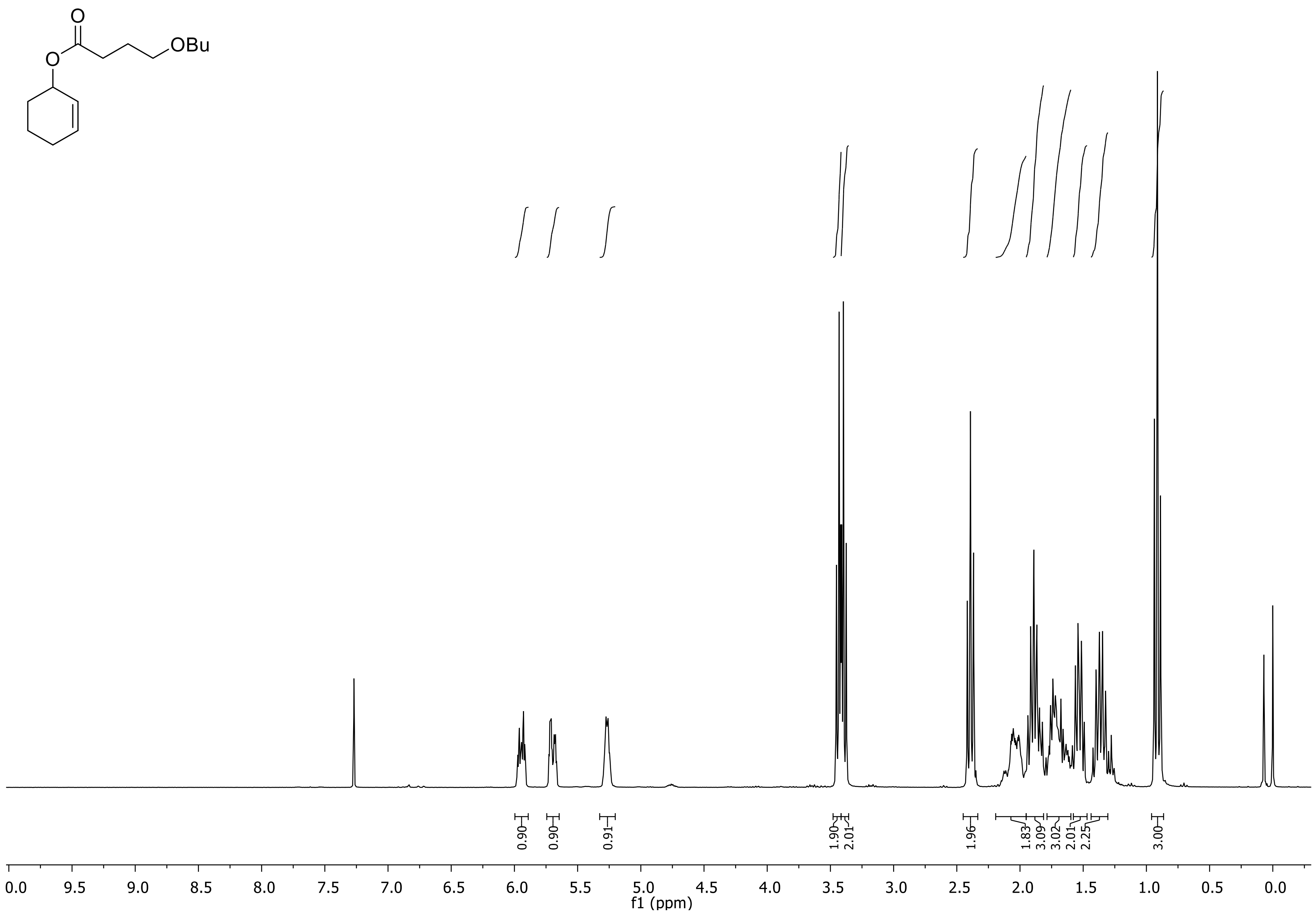

Cyclohex-2-enyl 4-butoxybutanoate (**30**)

^{13}C NMR (75 MHz, $CDCl_3$)

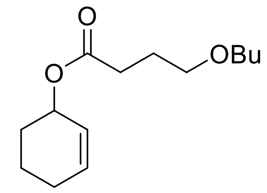

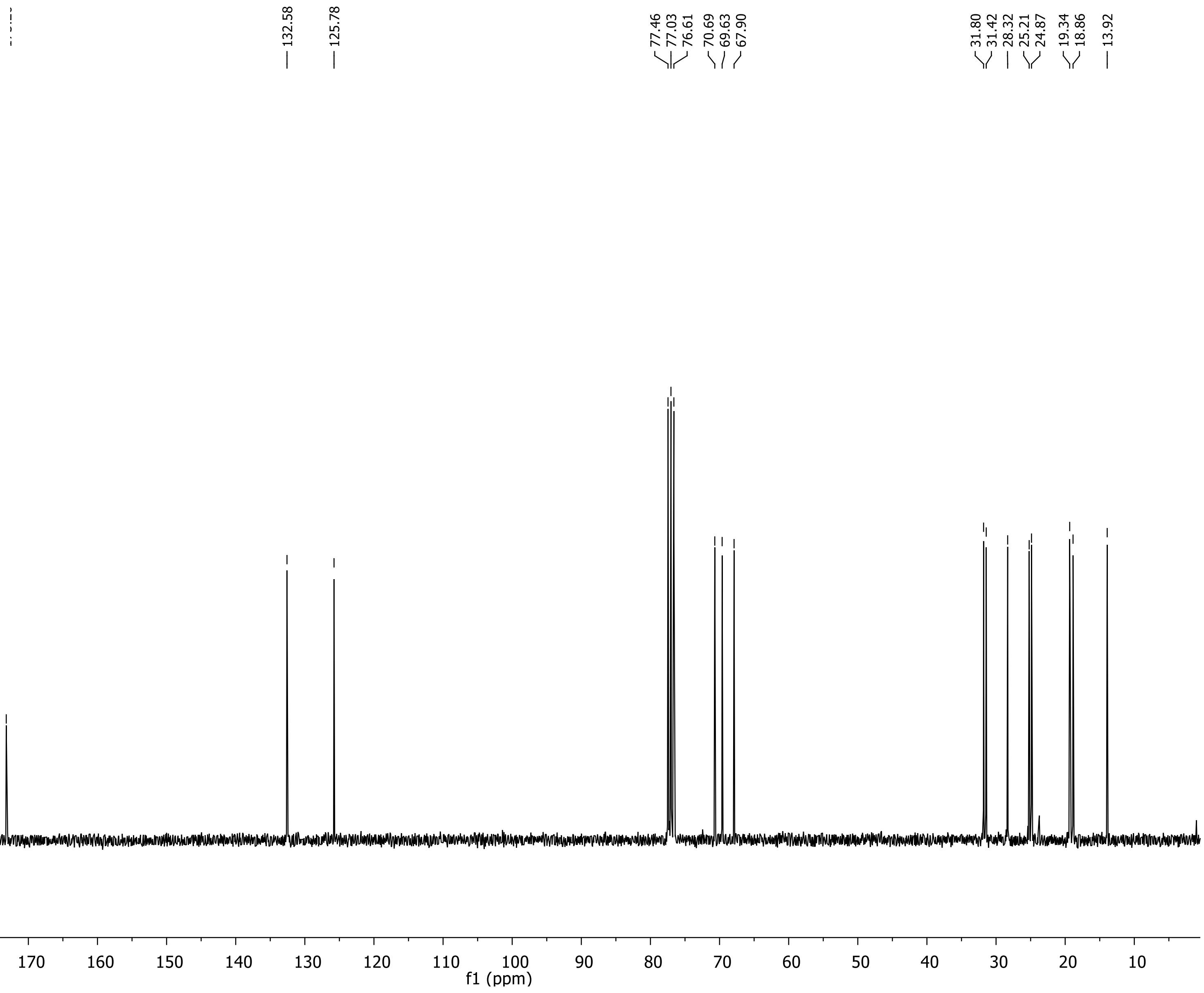

Ethyl 4-(cyclohexyloxy)-2-methylbutanoate (**31**)

^1H NMR (300 MHz, $CDCl_3$)

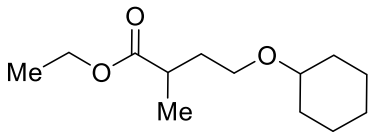

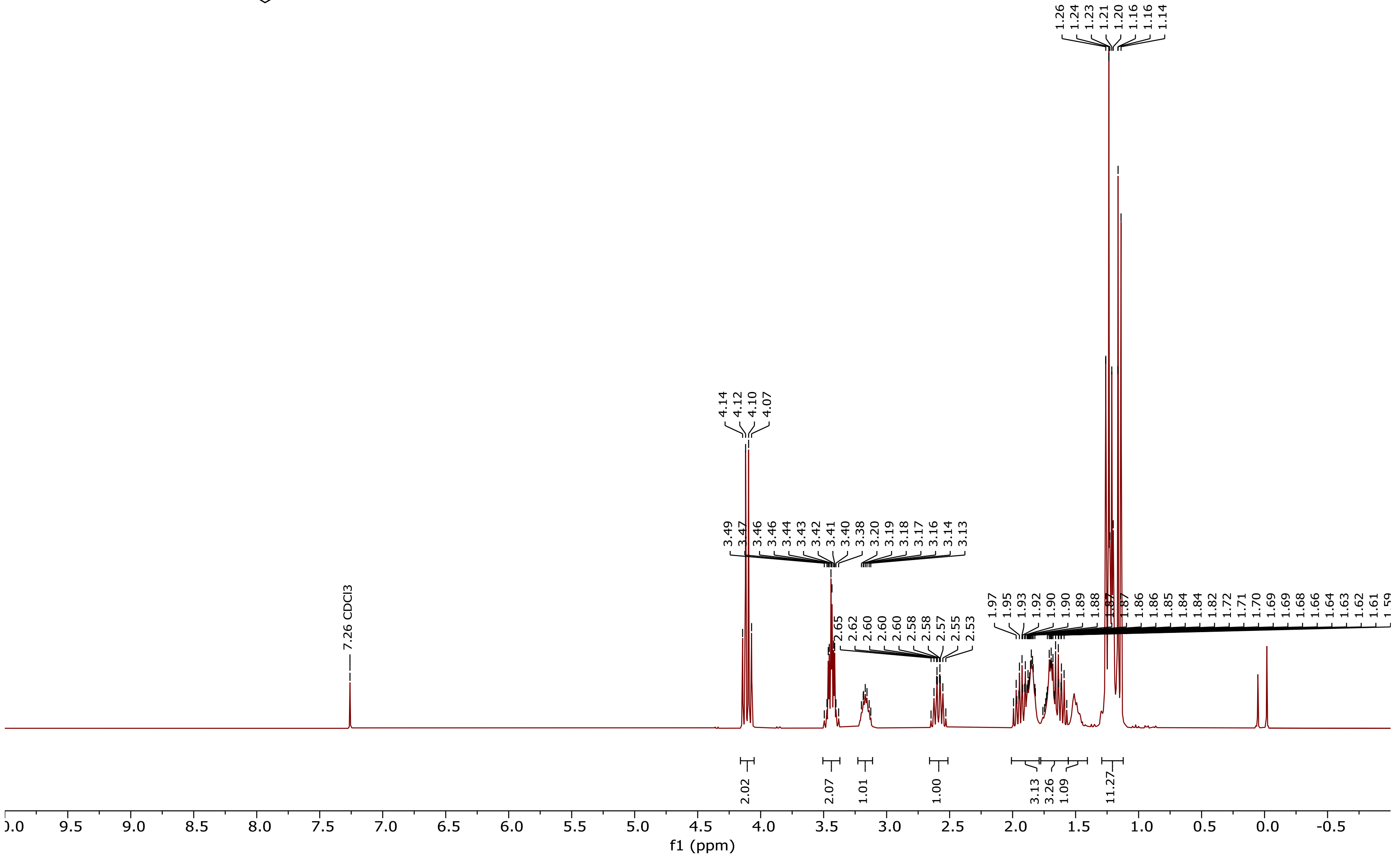

Ethyl 4-(cyclohexyloxy)-2-methylbutanoate (**31**)

^{13}C NMR (75 MHz, $CDCl_3$)

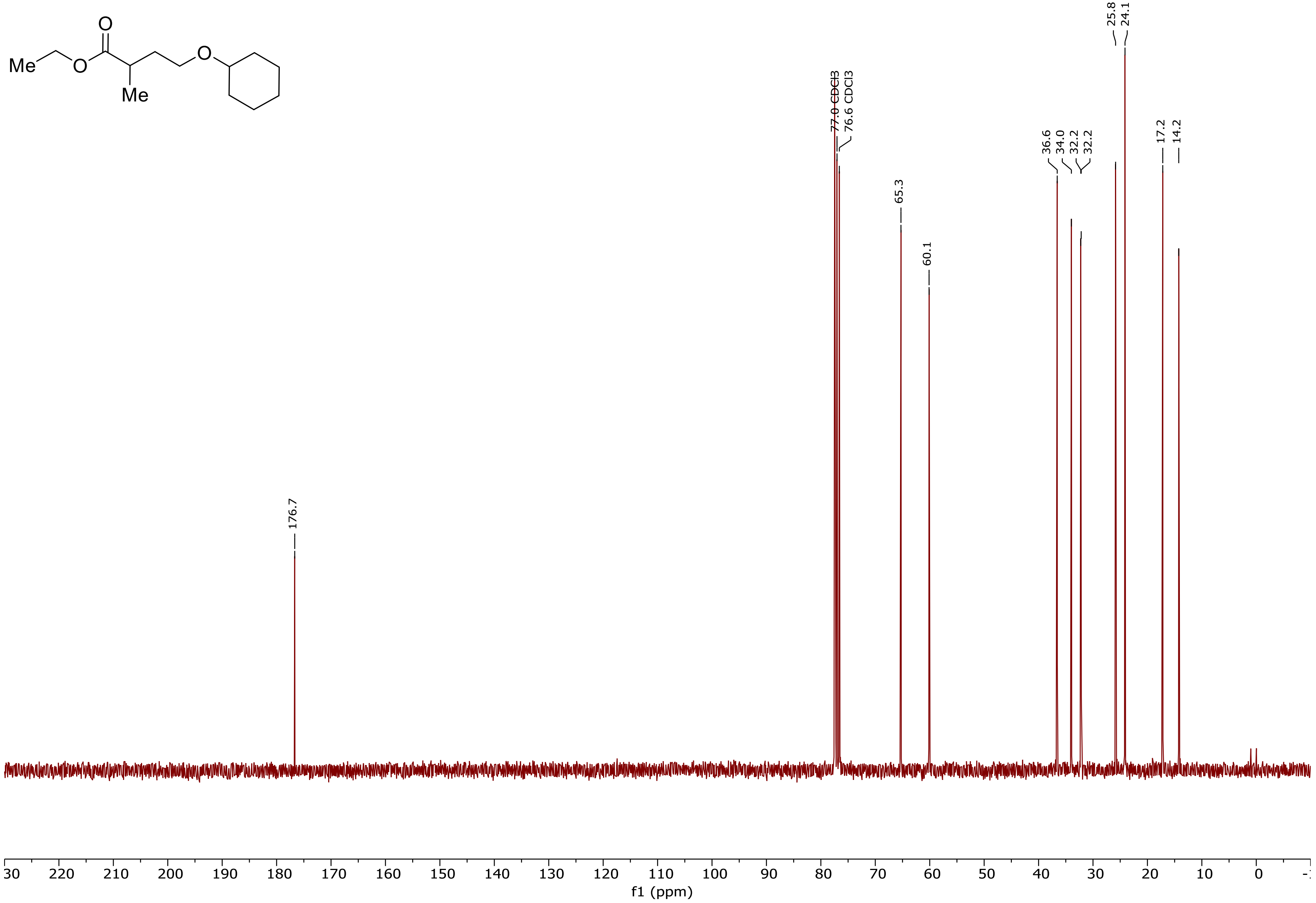

Benzyl 4-methoxypentanoate (**32**)

^{1}H NMR (300 MHz, $CDCl_3$)

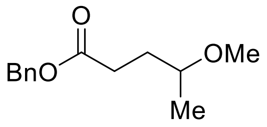

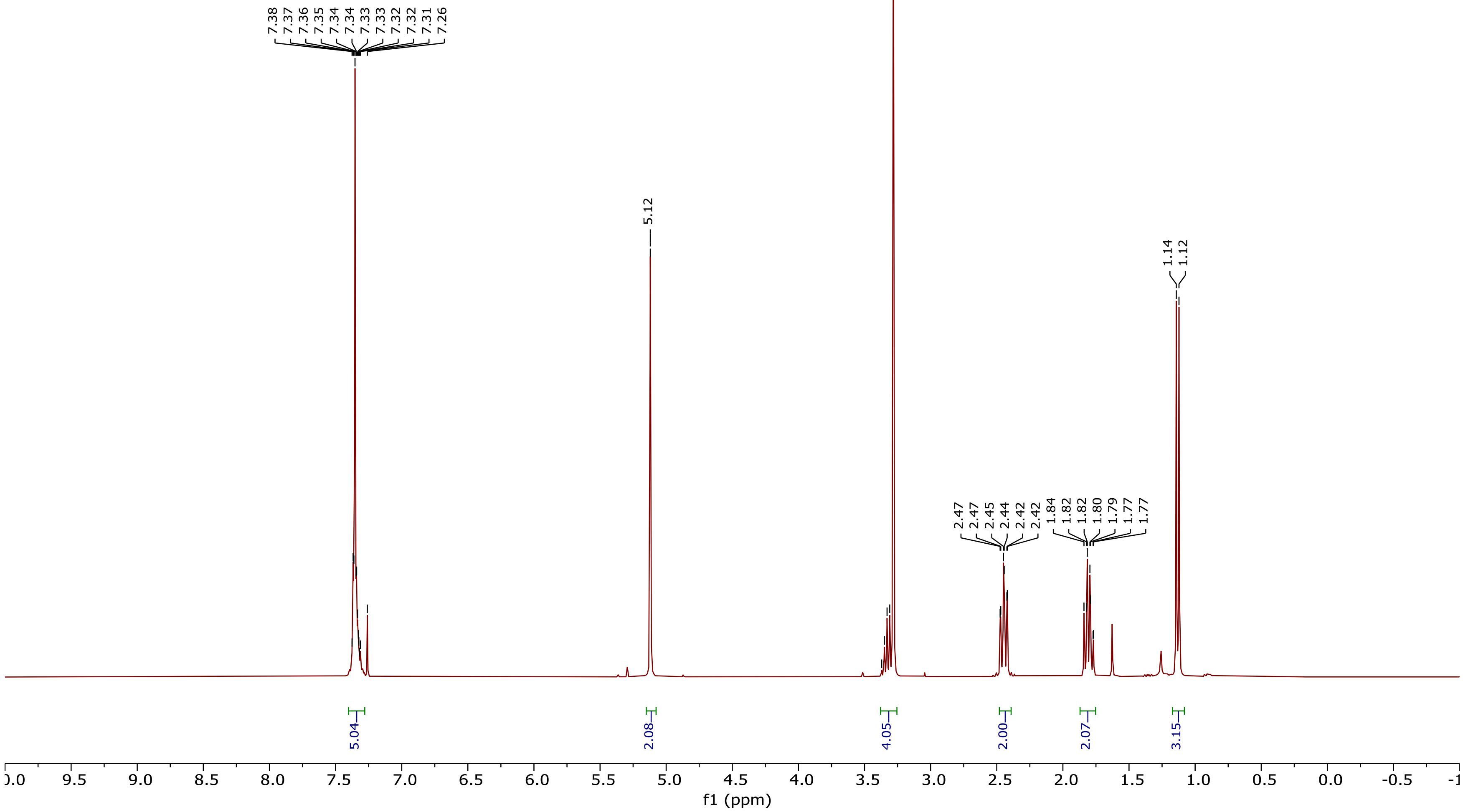

Benzyl 4-methoxypentanoate (**32**)

^{13}C NMR (75 MHz, $CDCl_3$)

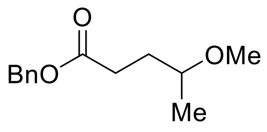

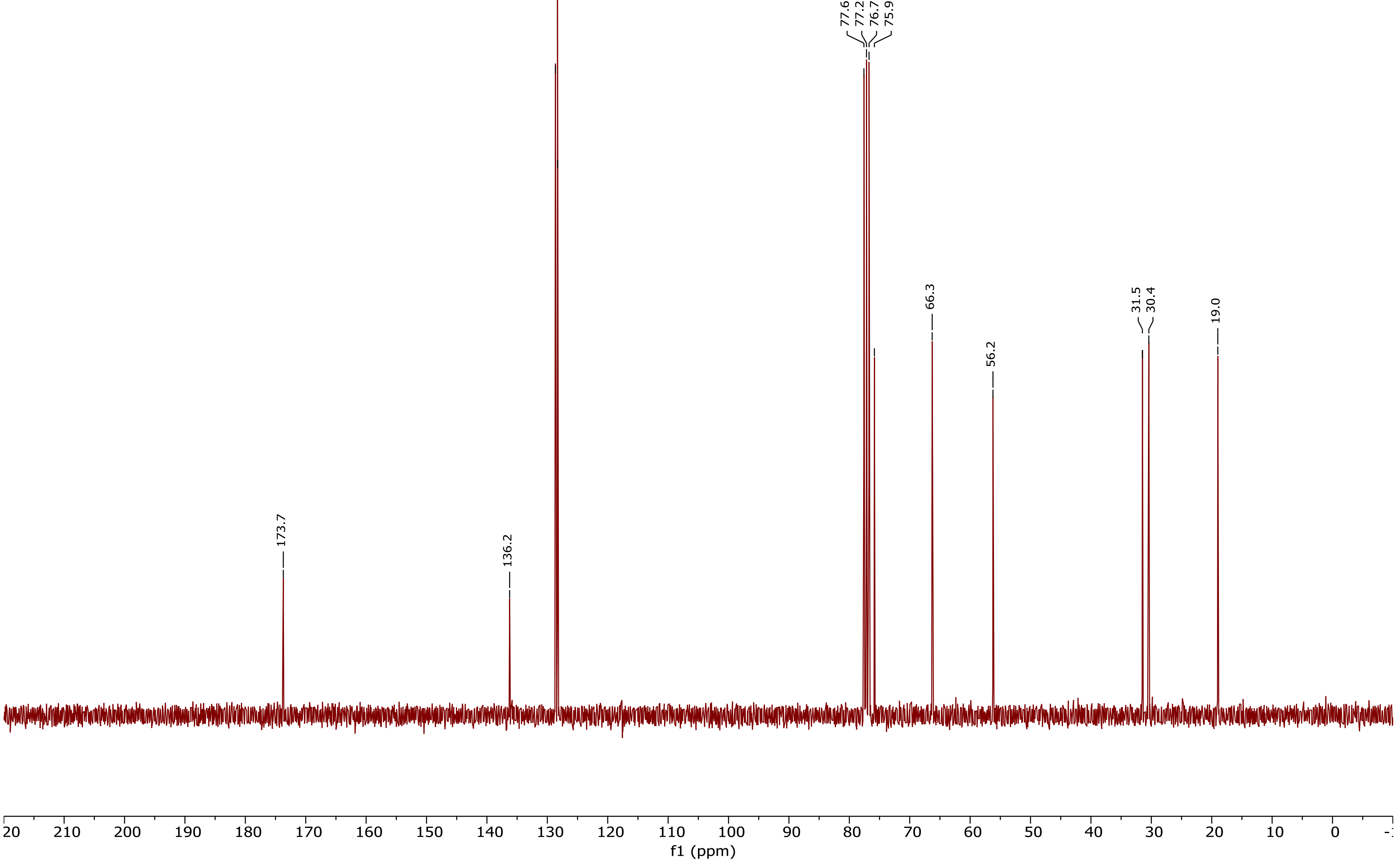

Diethyl 2-(1-ethoxypropan-2-yl)malonate (**33**)

^{1}H NMR (300 MHz, $CDCl_3$)

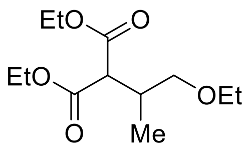

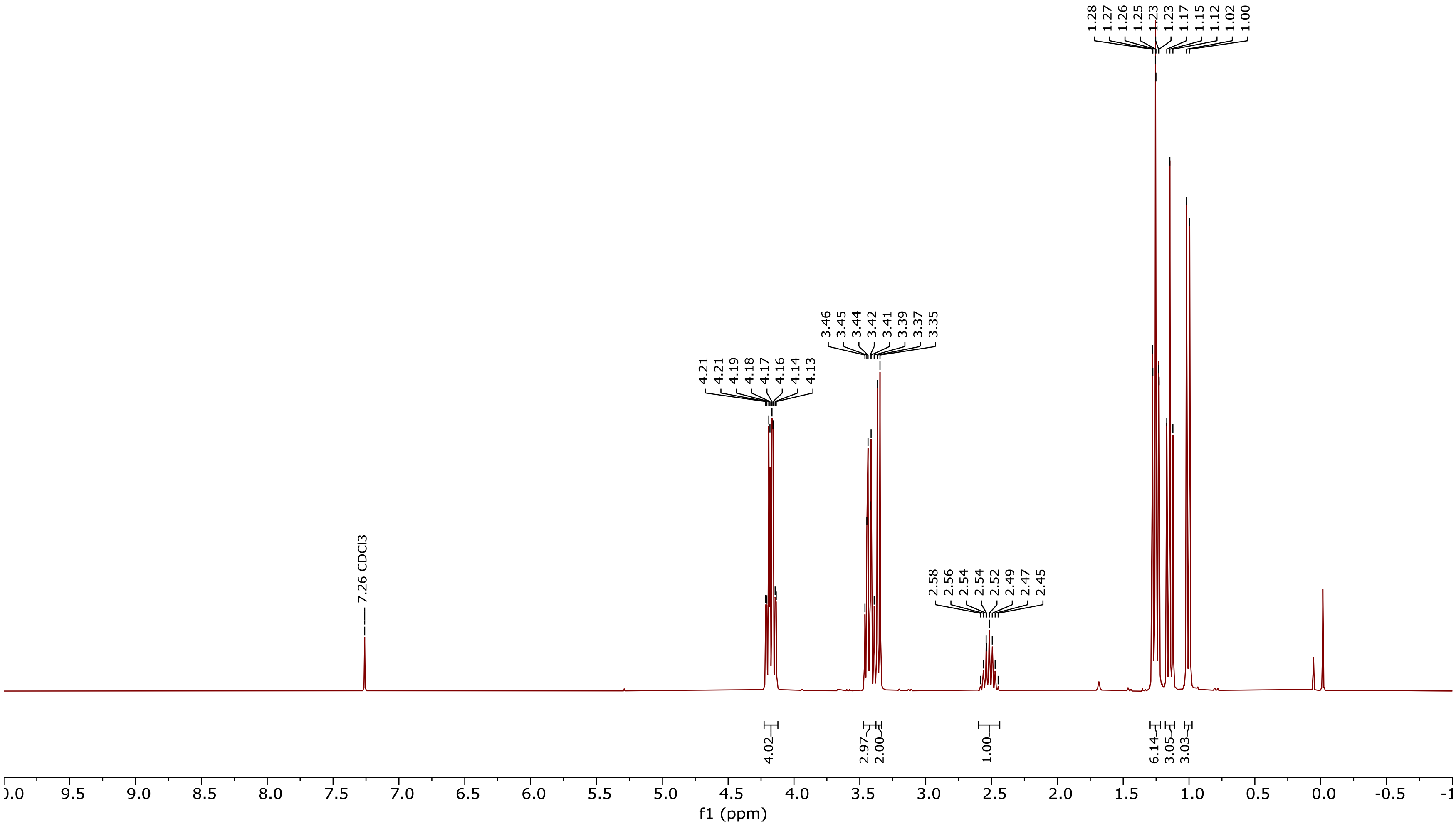

Diethyl 2-(1-ethoxypropan-2-yl)malonate (**33**)

^{13}C NMR (75 MHz, $CDCl_3$)

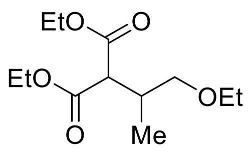

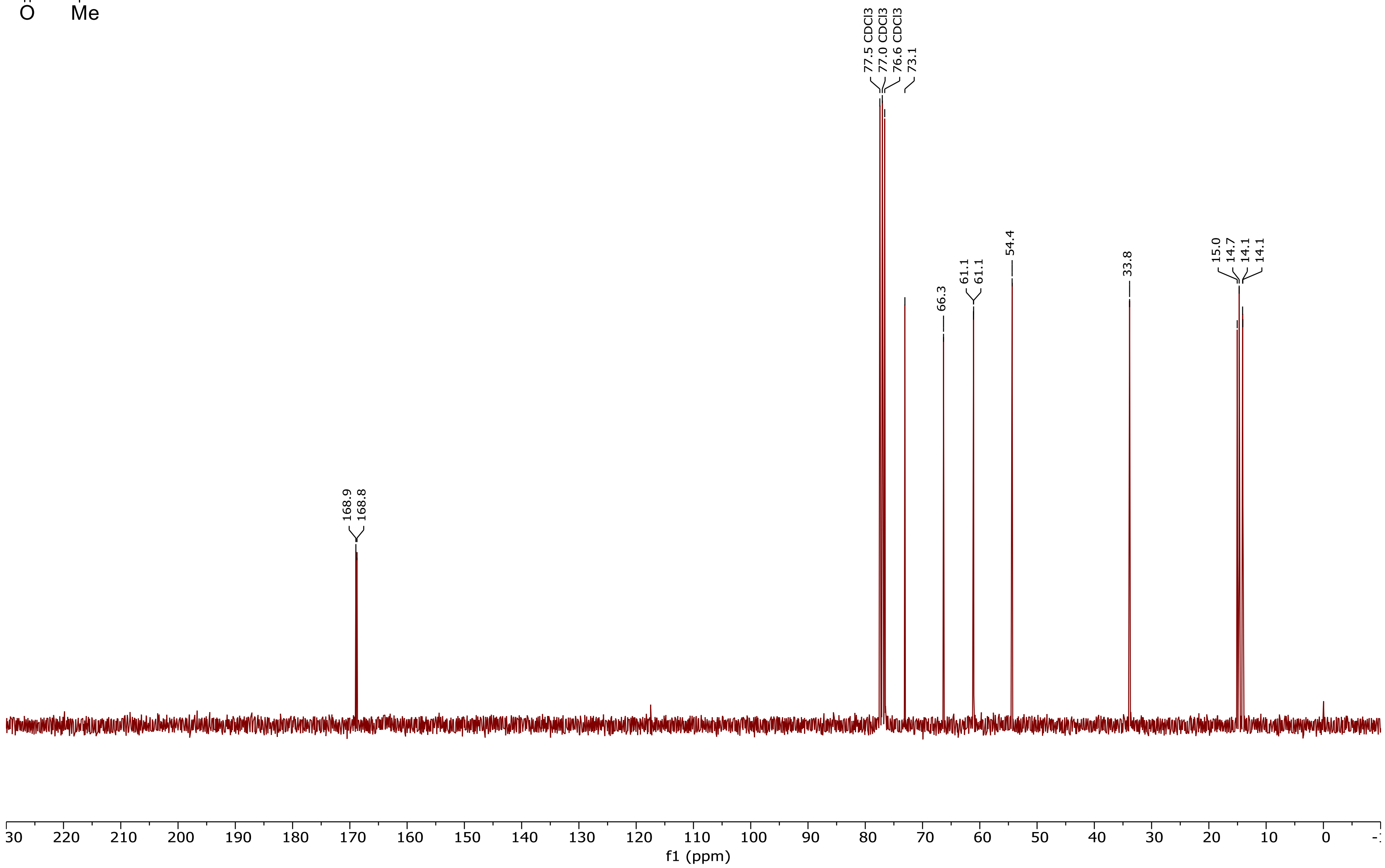

Diethyl 2-(1-ethoxypropan-2-yl)-2-methylmalonate (**34**)

^{1}H NMR (300 MHz, $CDCl_3$)

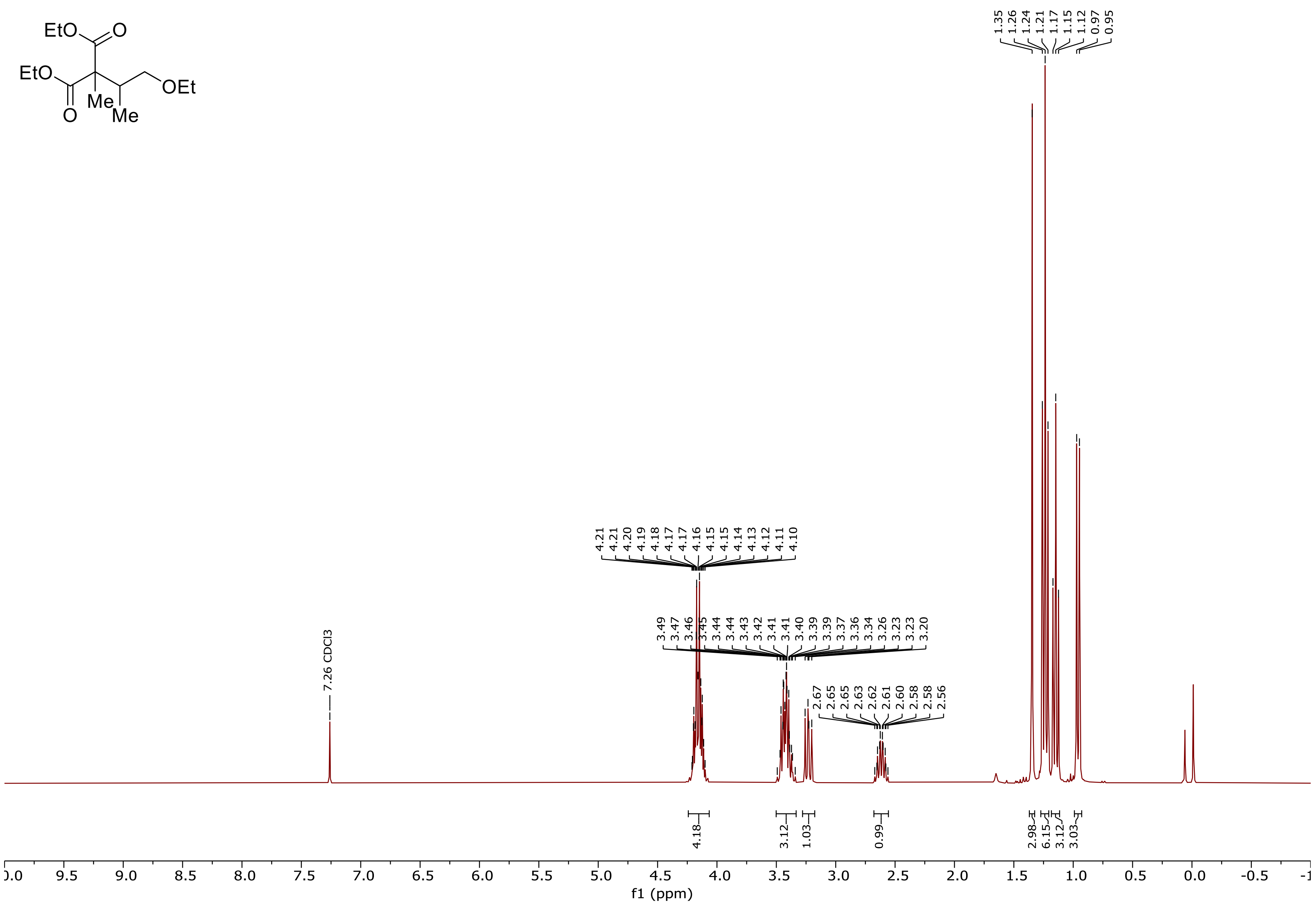

Diethyl 2-(1-ethoxypropan-2-yl)-2-methylmalonate (**34**) ^{13}C NMR (75 MHz, $CDCl_3$)

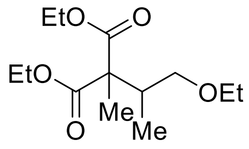

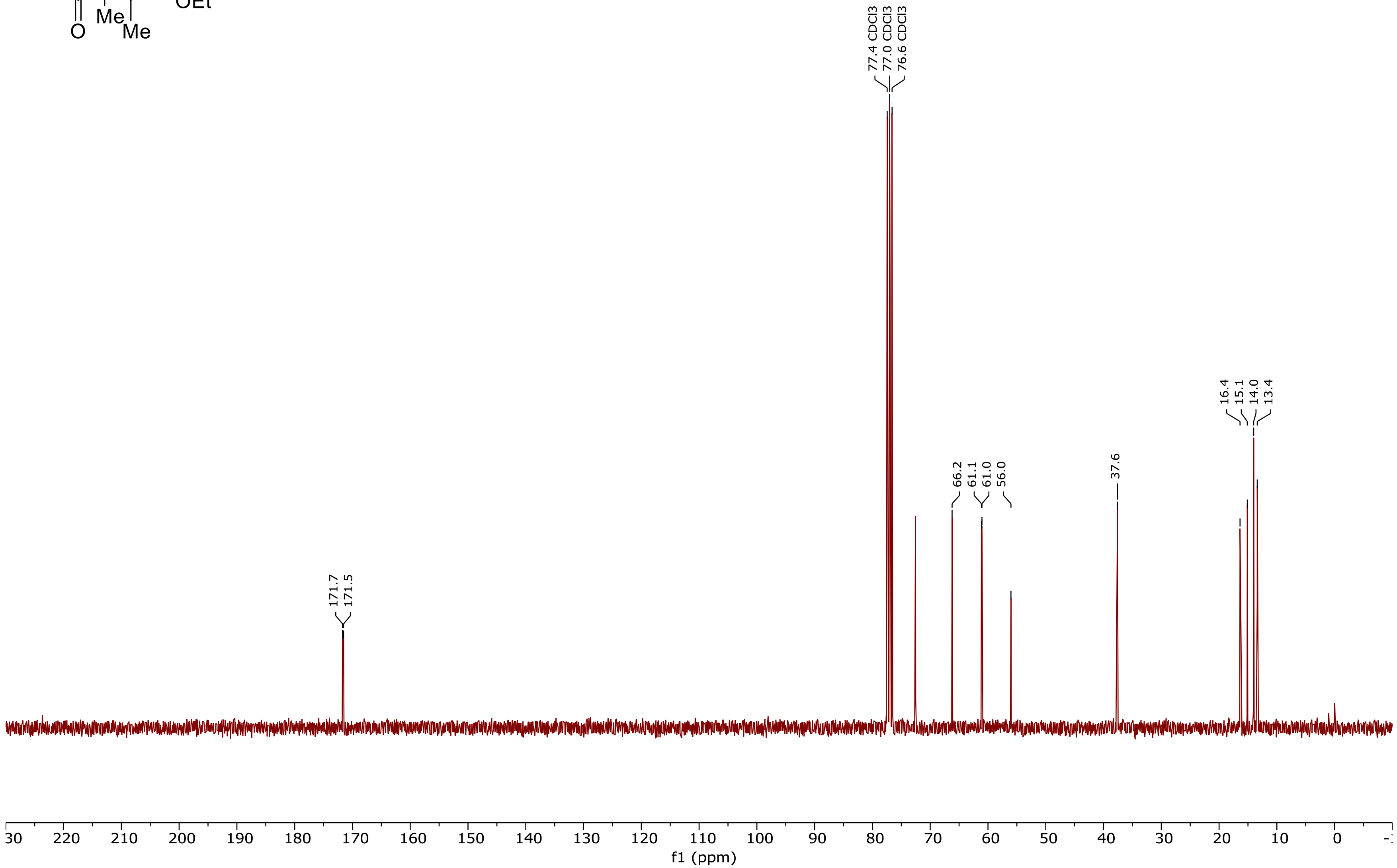

Benzyl 2-(tetrahydrofuran-3-yl)acetate (**35**)

^{1}H NMR (300 MHz, $CDCl_3$)

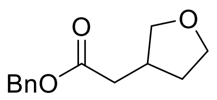

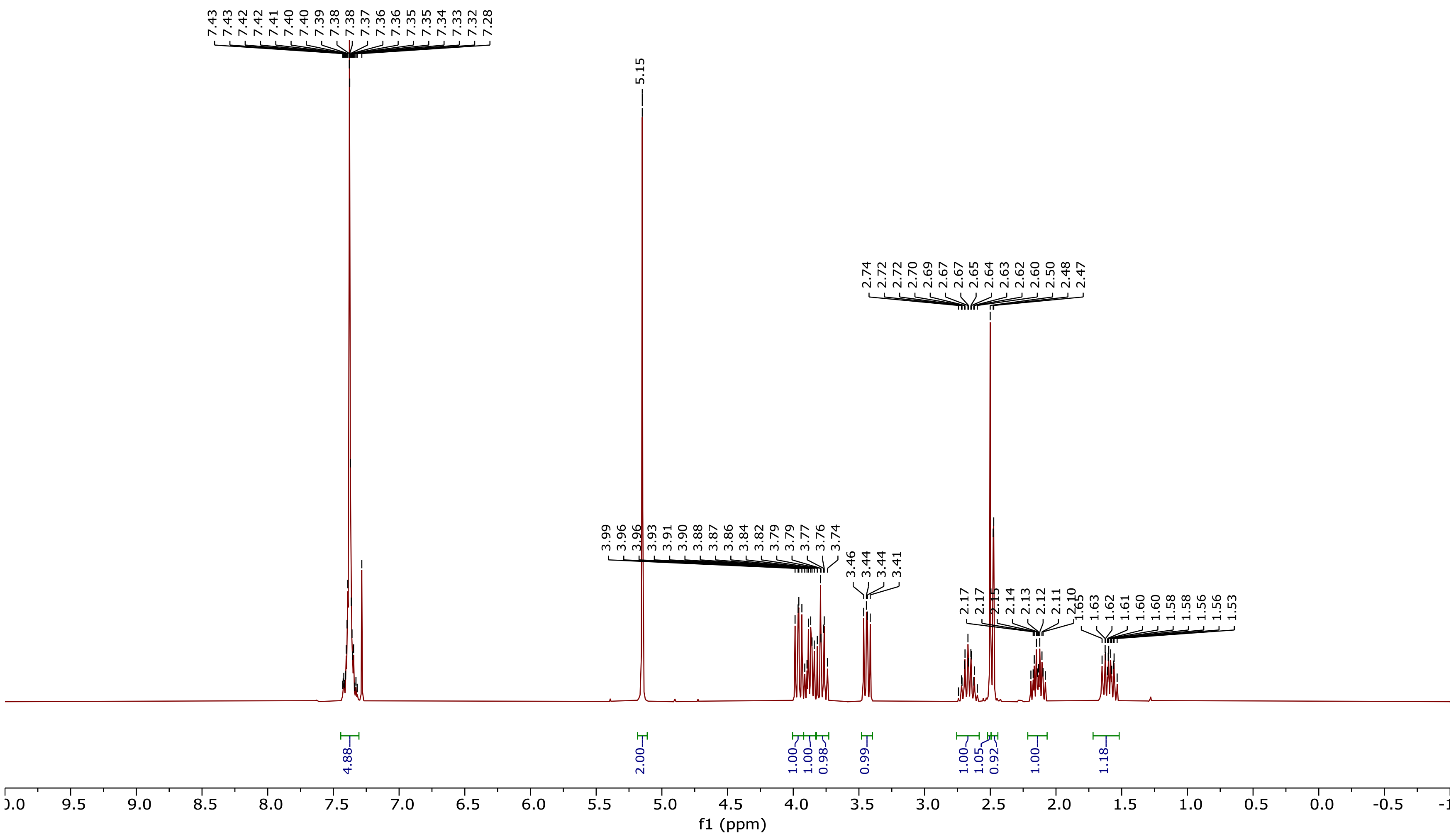

Benzyl 2-(tetrahydrofuran-3-yl)acetate (**35**)

^{13}C NMR (75 MHz, $CDCl_3$)

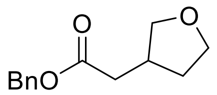

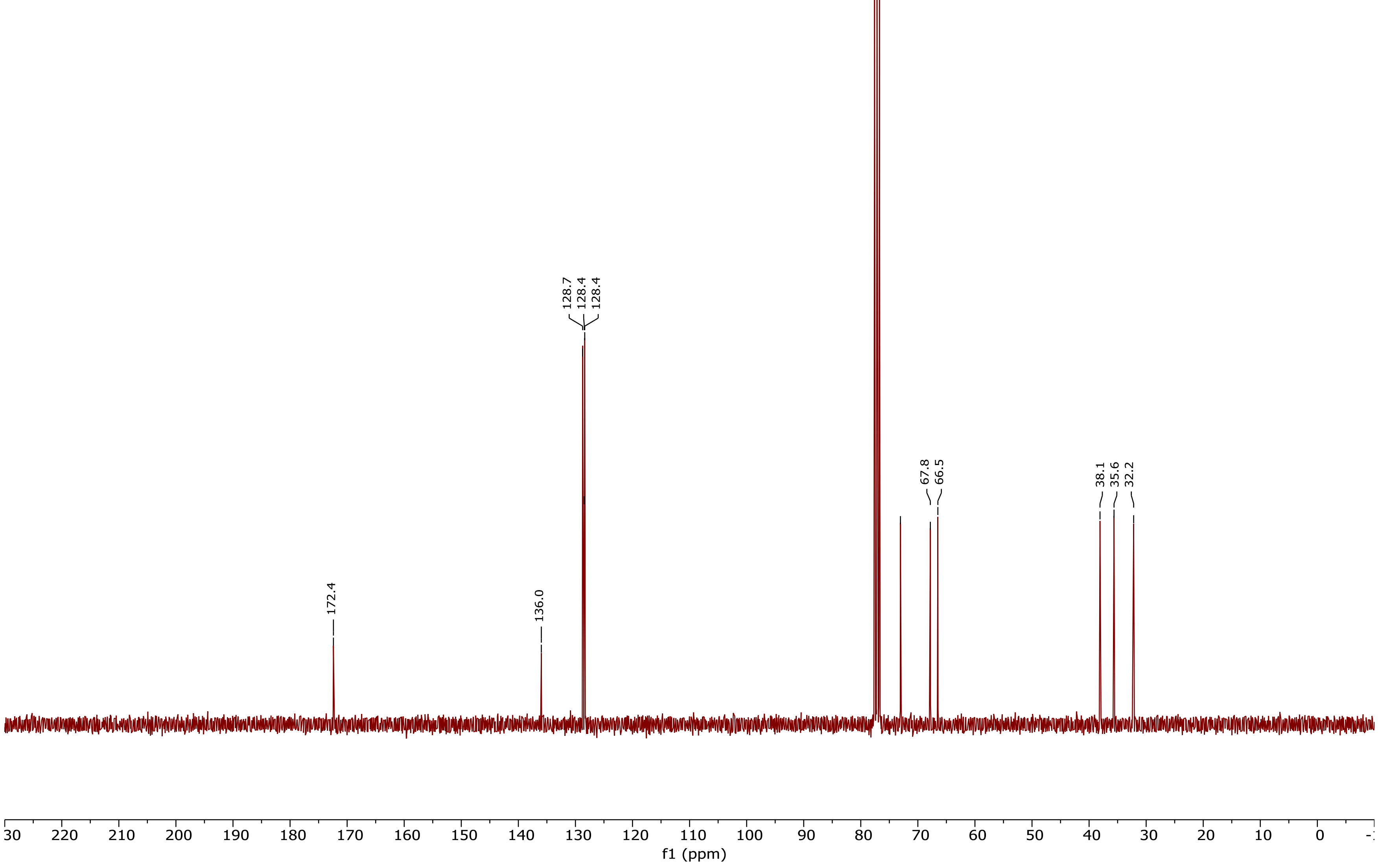

Benzyl 2-(tetrahydro-2*H*-pyran-3-yl)acetate (**36**)

^{1}H NMR (300 MHz, $CDCl_3$)

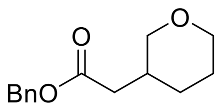

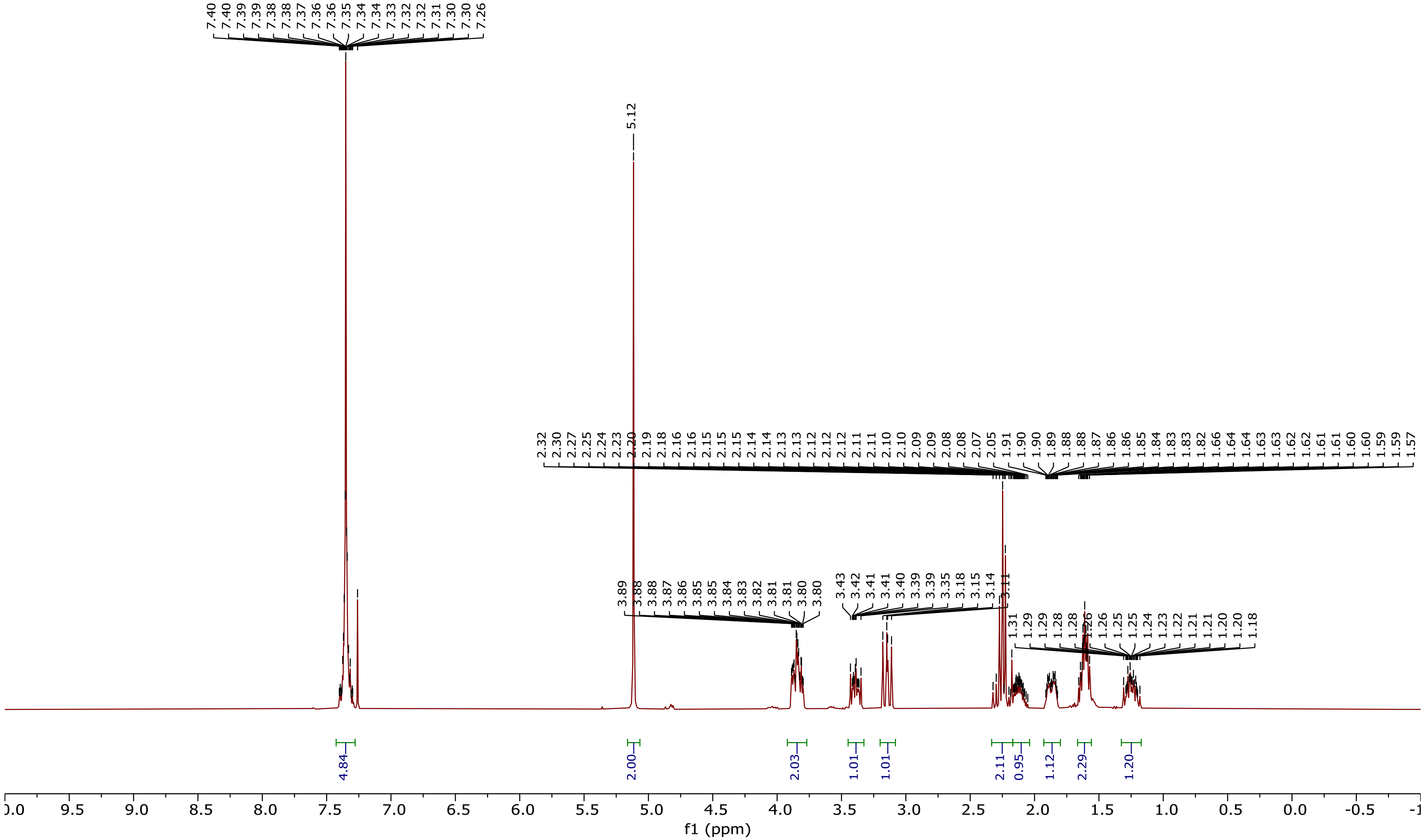

Benzyl 2-(tetrahydro-2*H*-pyran-3-yl)acetate (**36**)

^{13}C NMR (75 MHz, $CDCl_3$)

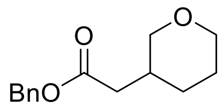

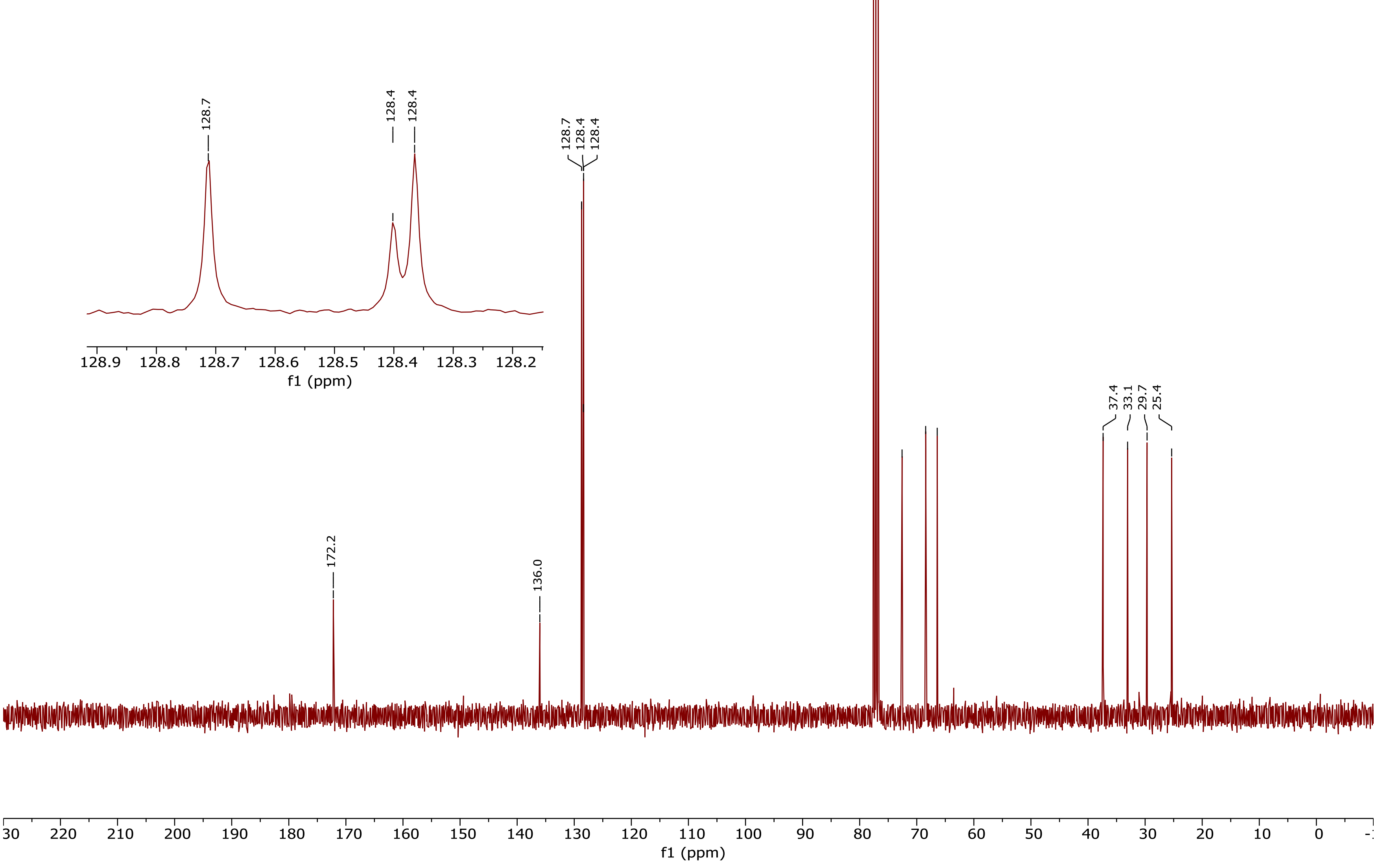

Benzyl 5,5-dimethyl-4-((trimethylsilyl)oxy)hexanoate (**38**)

^{1}H NMR (300 MHz, $CDCl_3$)

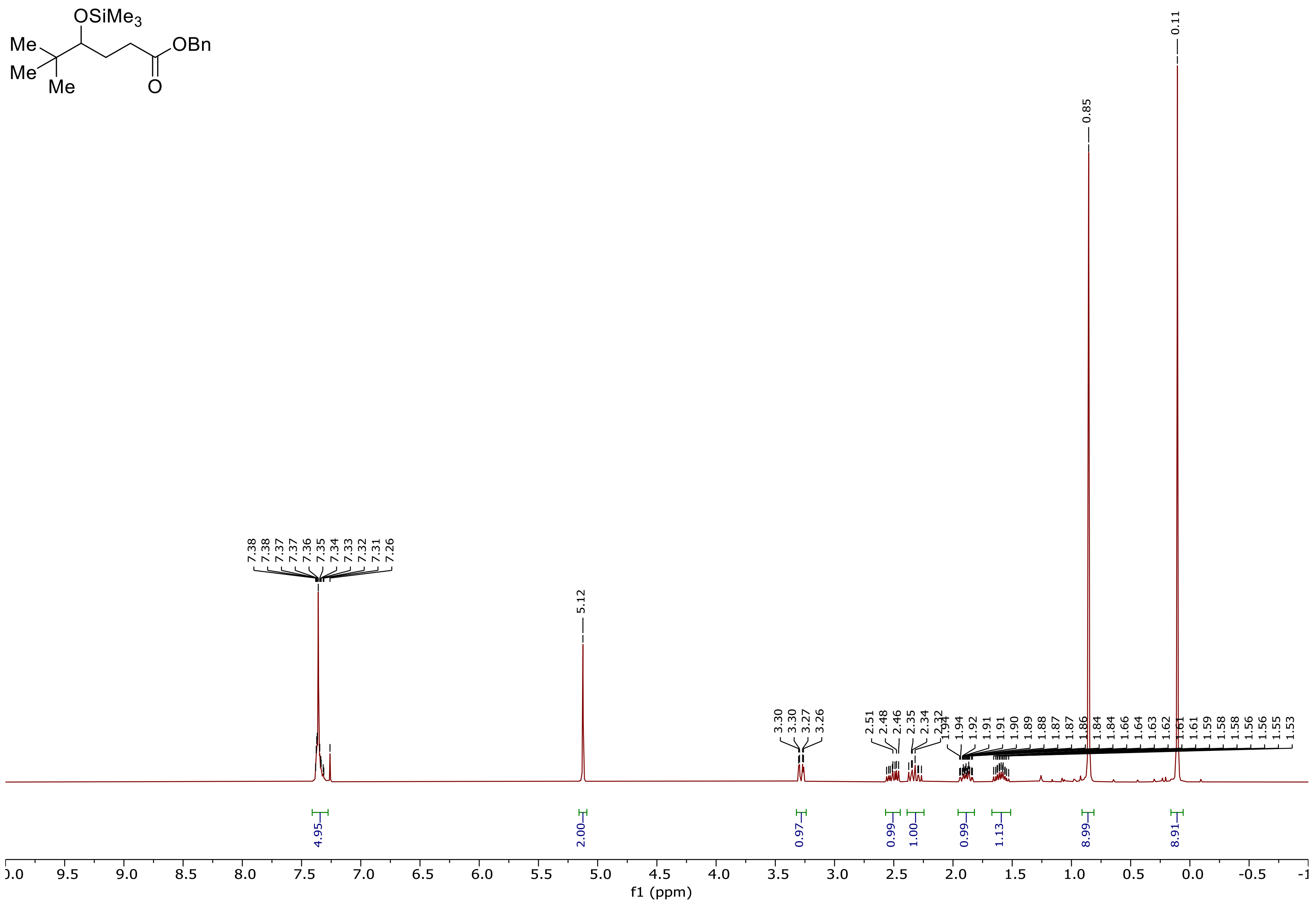

Benzyl 5,5-dimethyl-4-((trimethylsilyl)oxy)hexanoate (**38**)

^{13}C NMR (75 MHz, $CDCl_3$)

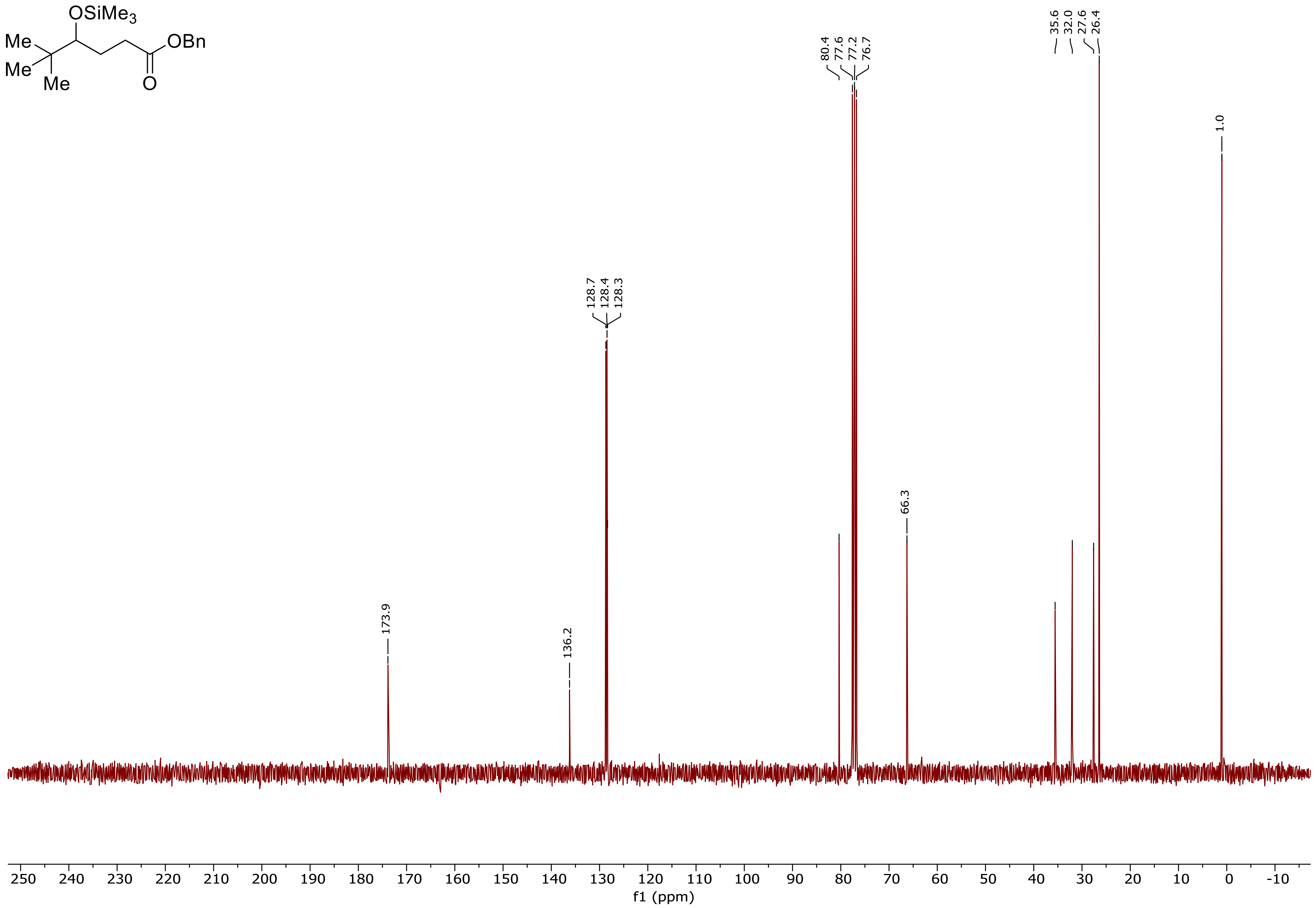

Benzyl 4-((3*S*,8*S*,9*S*,10*R*,13*S*,14*S*,17*S*)-3-acetoxy-10,13-dimethyl-2,3,4,7,8,9,10,11,12,13,14,15,16,17-tetradecahydro-1*H*-cyclopenta[*a*]phenanthren-17-yl)-4-((*tert*-butyldimethylsilyl)oxy)butanoate (**39**)

^{1}H NMR (300 MHz, $CDCl_3$)

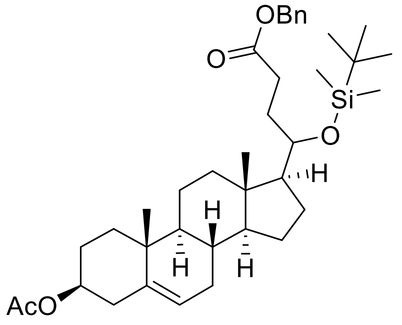

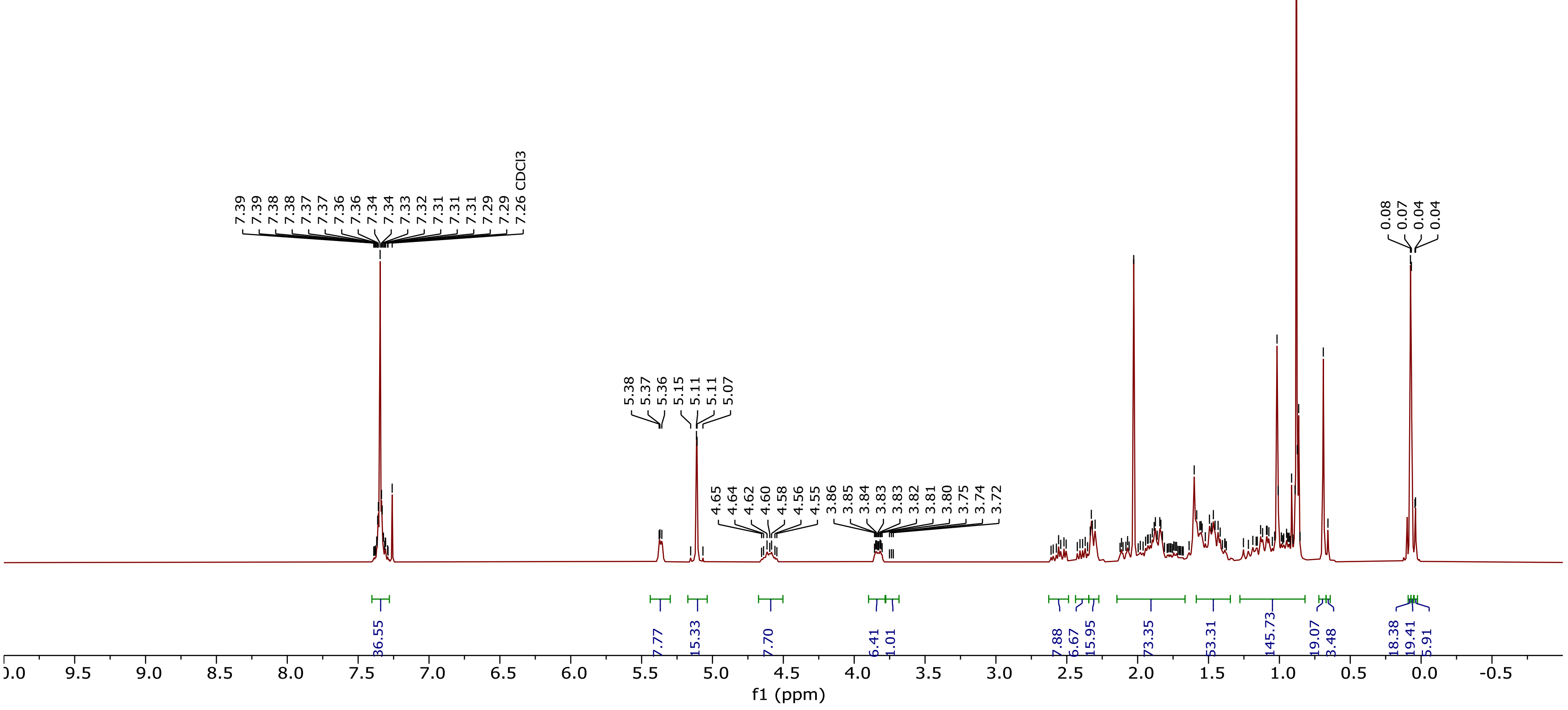

Benzyl 4-((3*S*,8*S*,9*S*,10*R*,13*S*,14*S*,17*S*)-3-acetoxy-10,13-dimethyl-2,3,4,7,8,9,10,11,12,13,14,15,16,17-tetradecahydro-1*H*-cyclopenta[*a*]phenanthren-17-yl)-4-((*tert*-butyldimethylsilyl)oxy)butanoate (**39**)

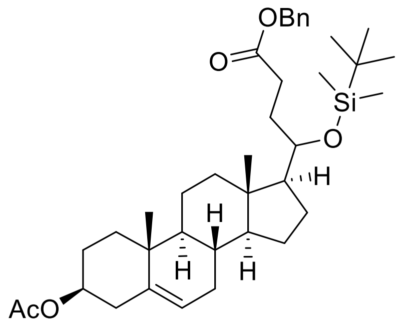

^{13}C NMR (75 MHz, $CDCl_3$)

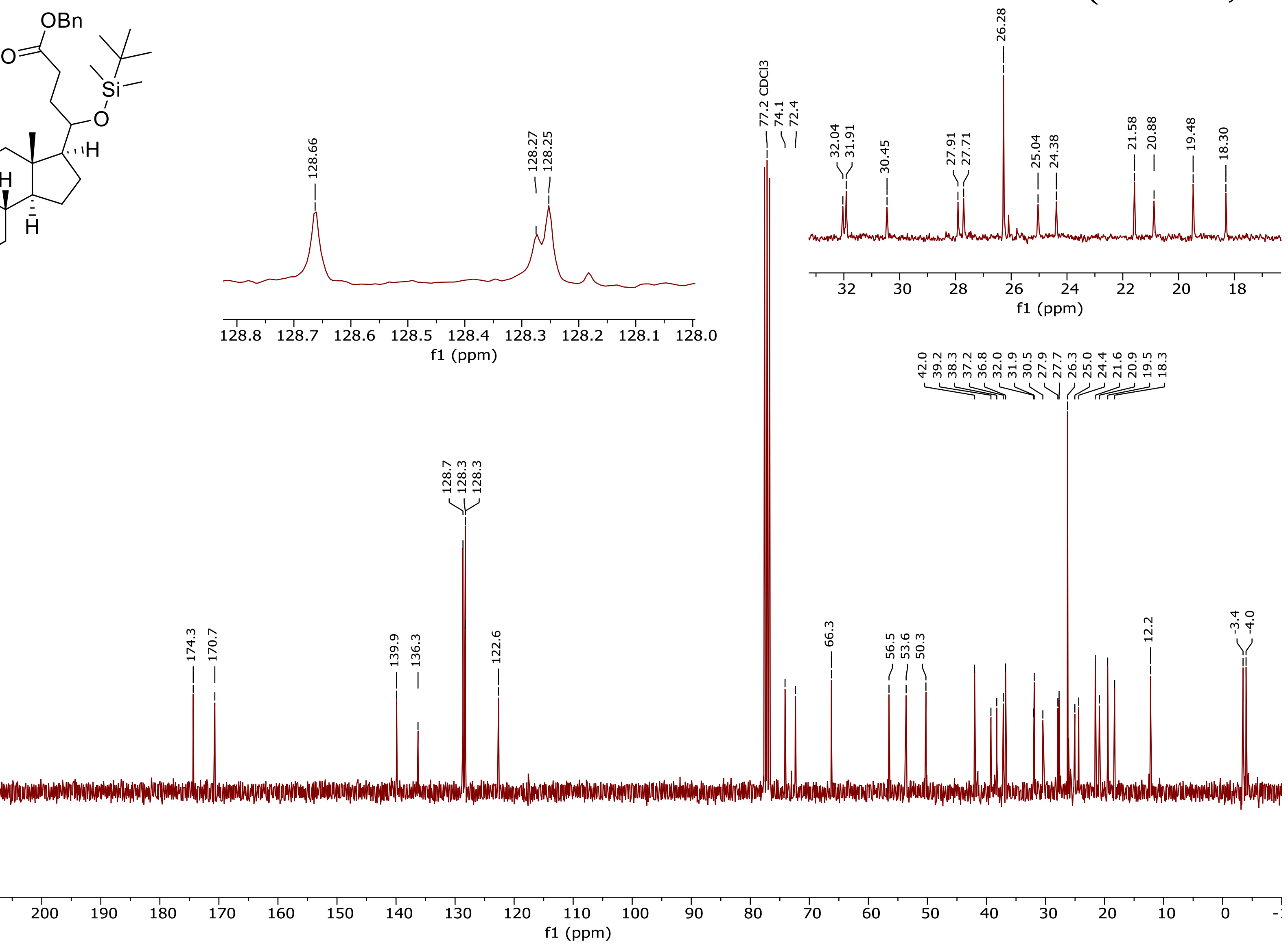

Diethyl 2-(2-(trimethylsilyloxy)cyclohexyl) malonate (**40**)

^{1}H NMR (300 MHz, $CDCl_3$)

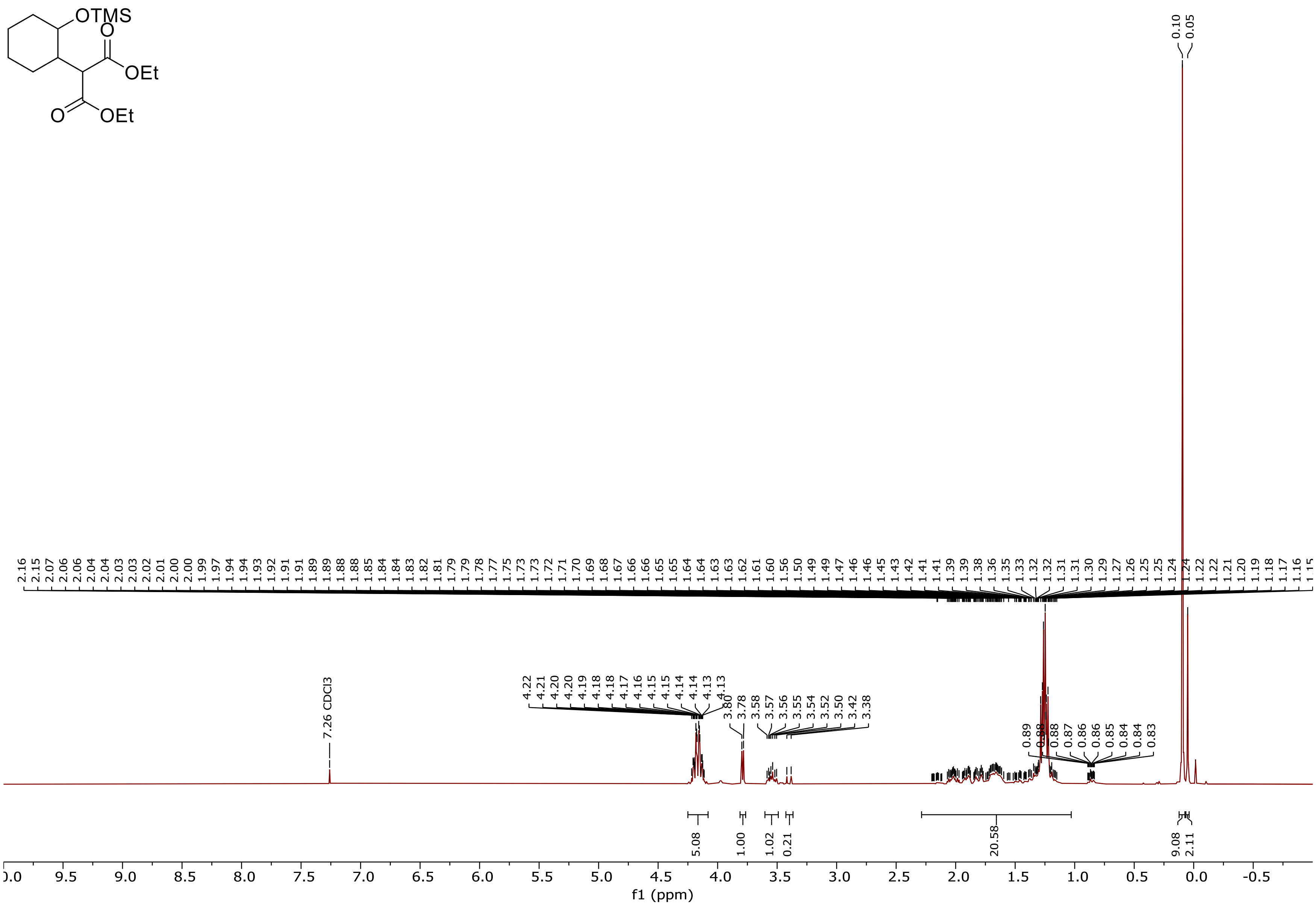

Diethyl 2-(2-(trimethylsilyloxy)cyclohexyl) malonate (**40**)

^{13}C NMR (75 MHz, $CDCl_3$)

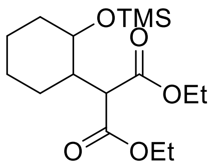

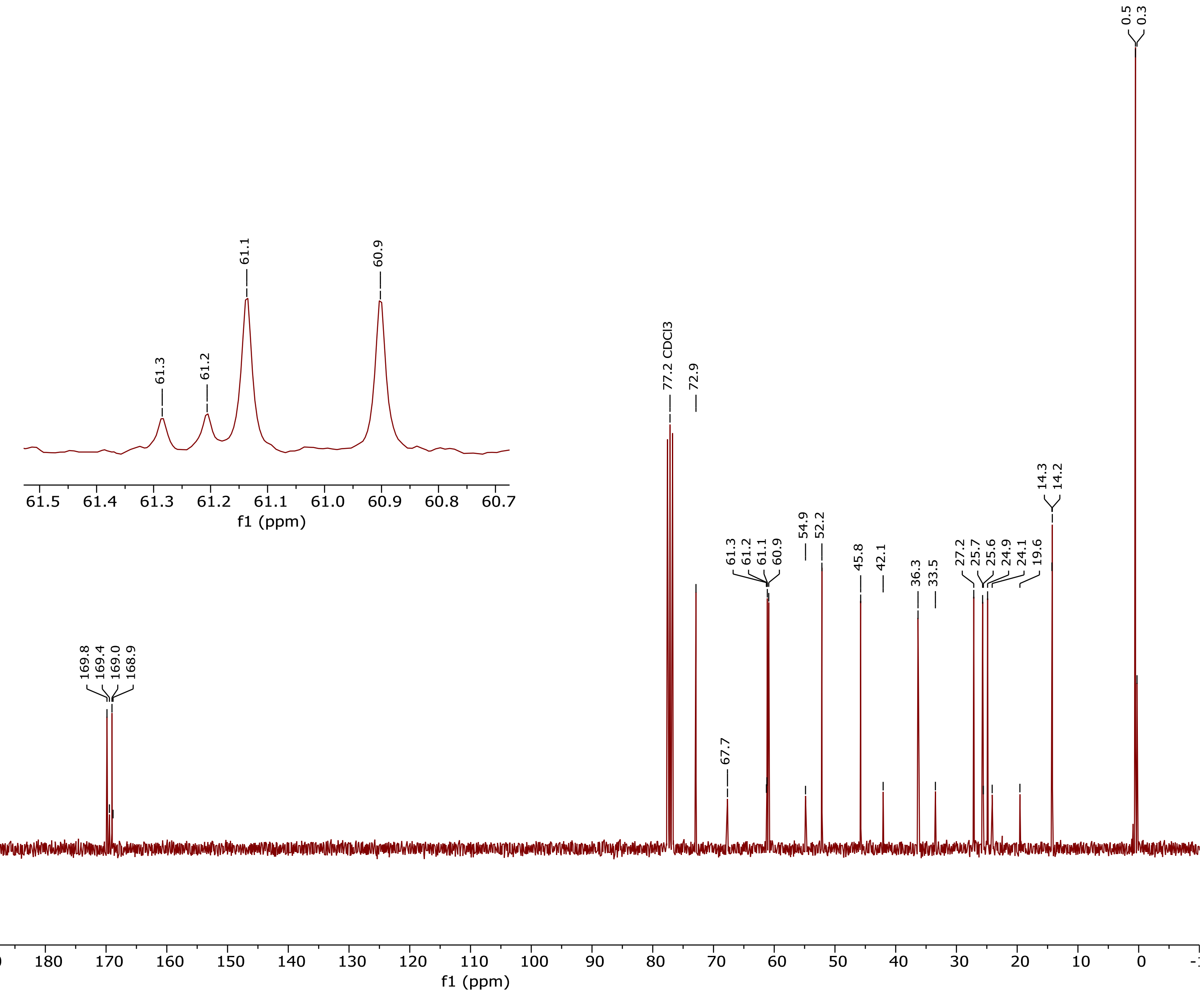

Ethyl 2-((*8R*,*9S*,*13S*,*14S*,*16S*,*17S*)-17-((tert-butyldimethylsilyl)oxy)-3-methoxy-13-methyl-7,8,9,11,12,13,14,15,16,17-decahydro-6*H*-cyclopenta[*a*]phenanthren-16-yl)acetate (**41**)

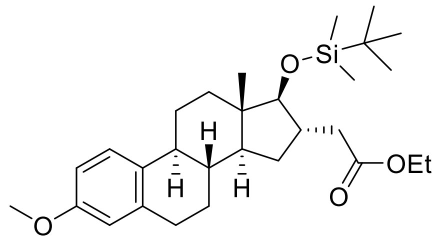

^{1}H NMR (300 MHz, $CDCl_3$)

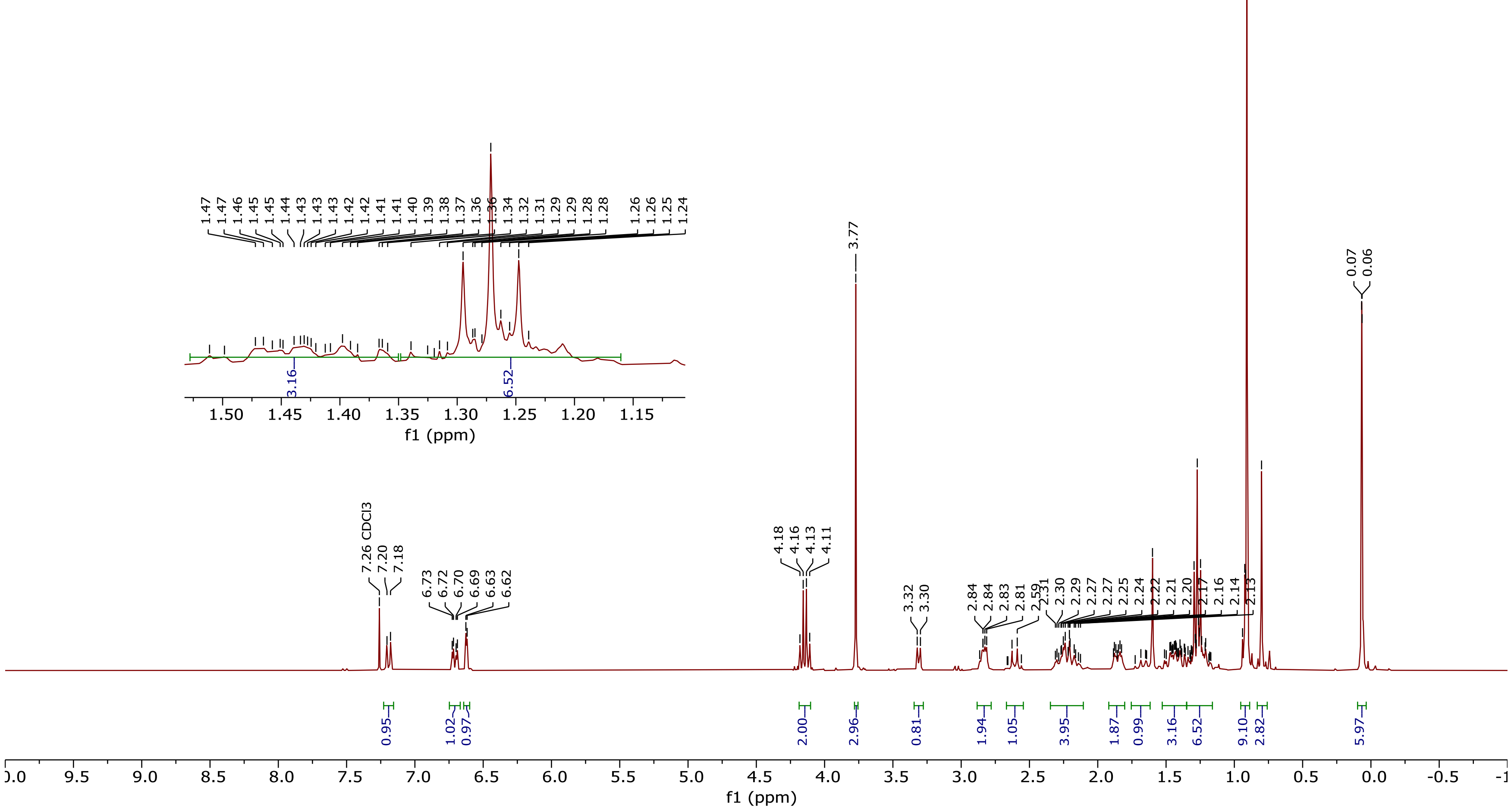

Ethyl 2-((*8R*,*9S*,*13S*,*14S*,*16S*,*17S*)-17-((tert-butyldimethylsilyl)oxy)-3-methoxy-13-methyl-7,8,9,11,12,13,14,15,16,17-decahydro-6*H*-cyclopenta[*a*]phenanthren-16-yl)acetate (**41**)

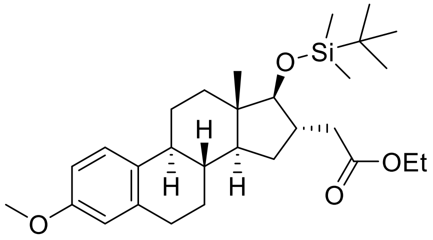

^{13}C NMR (75 MHz, $CDCl_3$)

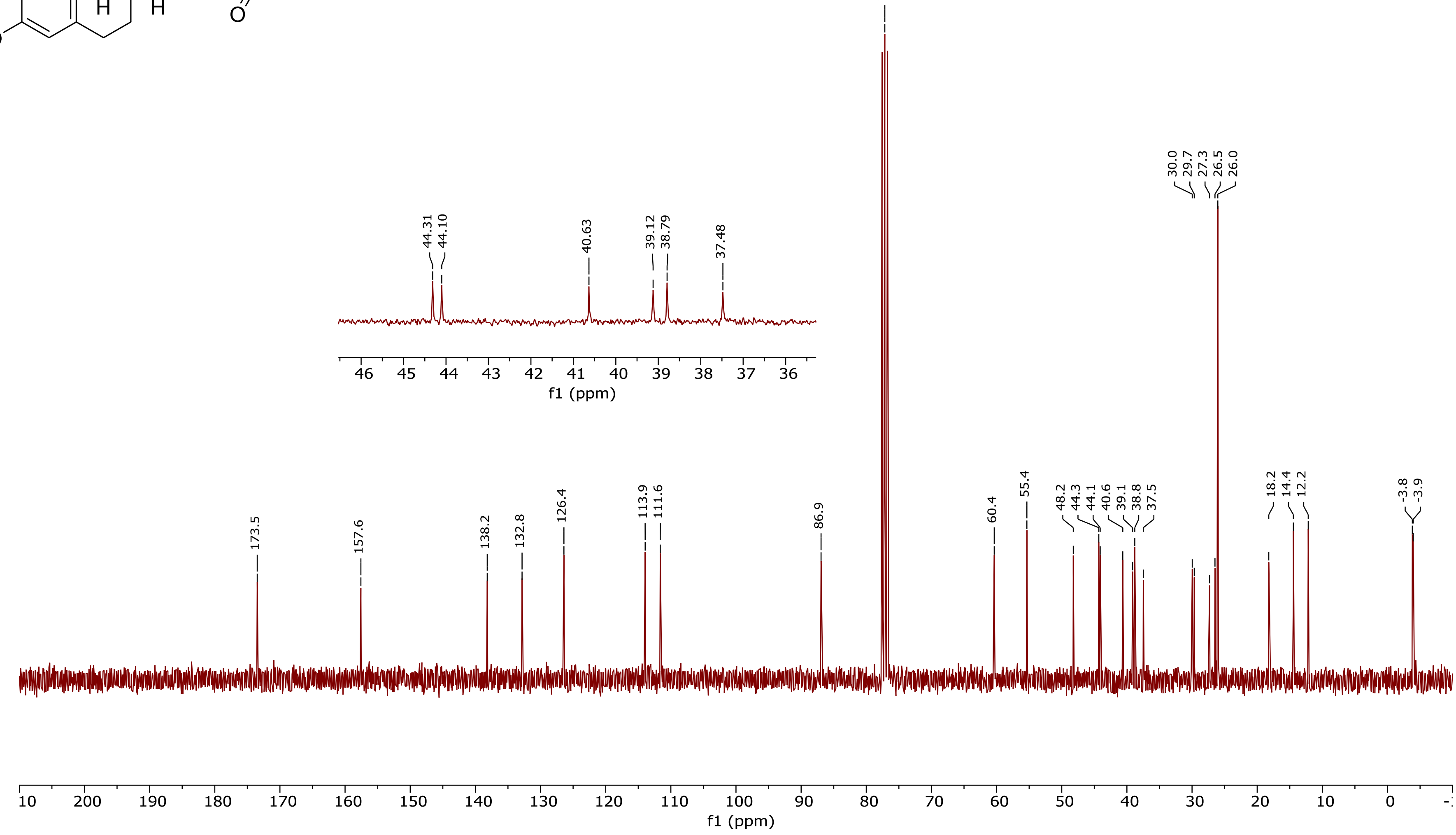

(*E*)-Ethyl 4-(2-oxopyrrolidin-1-yl)but-3-enoate (**45**) ^{1}H NMR (300 MHz, $CDCl_3$)

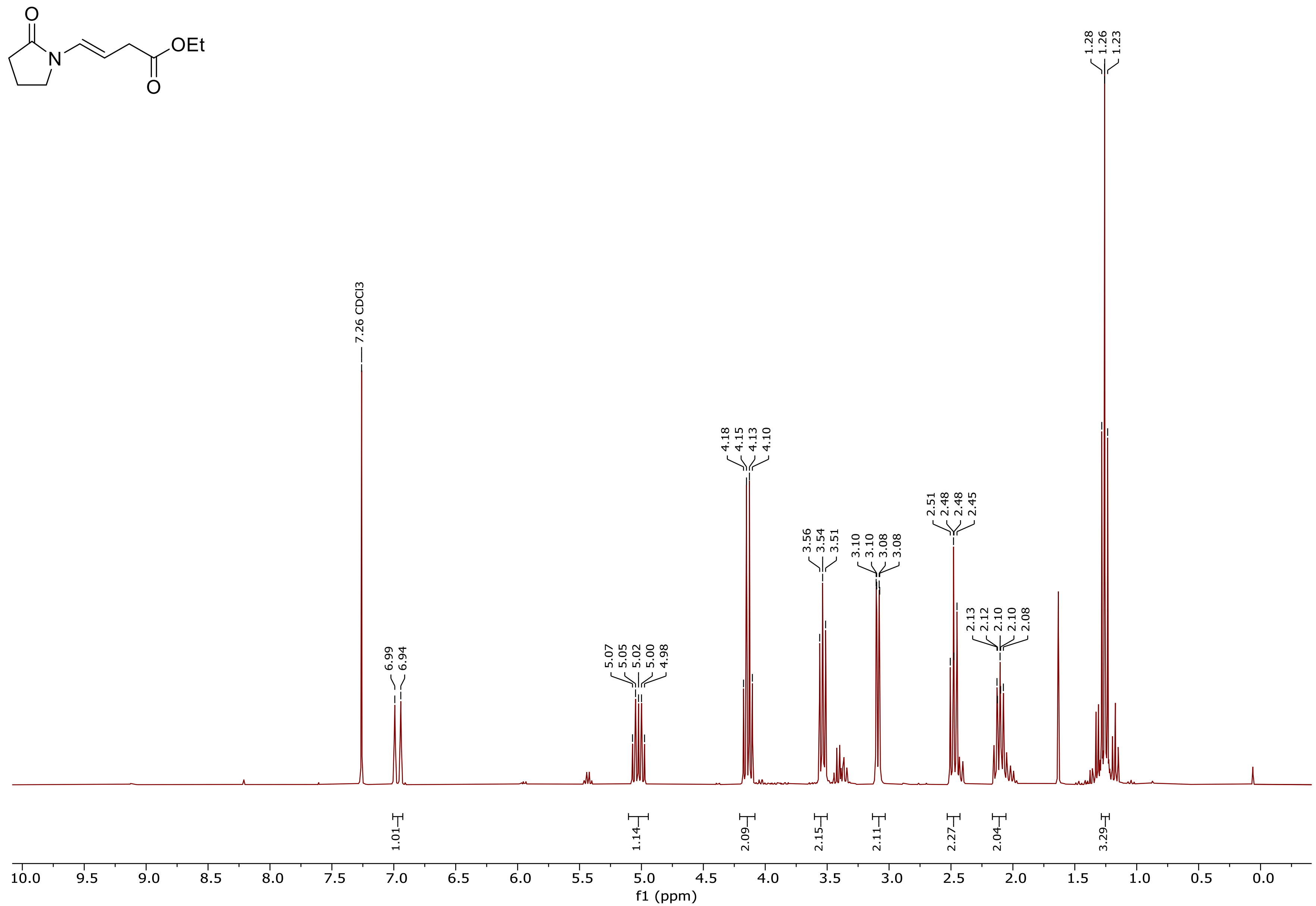

(*E*)-Ethyl 4-(2-oxopyrrolidin-1-yl)but-3-enoate (**45**)

^{13}C NMR (75 MHz, $CDCl_3$)

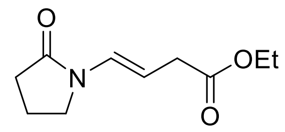

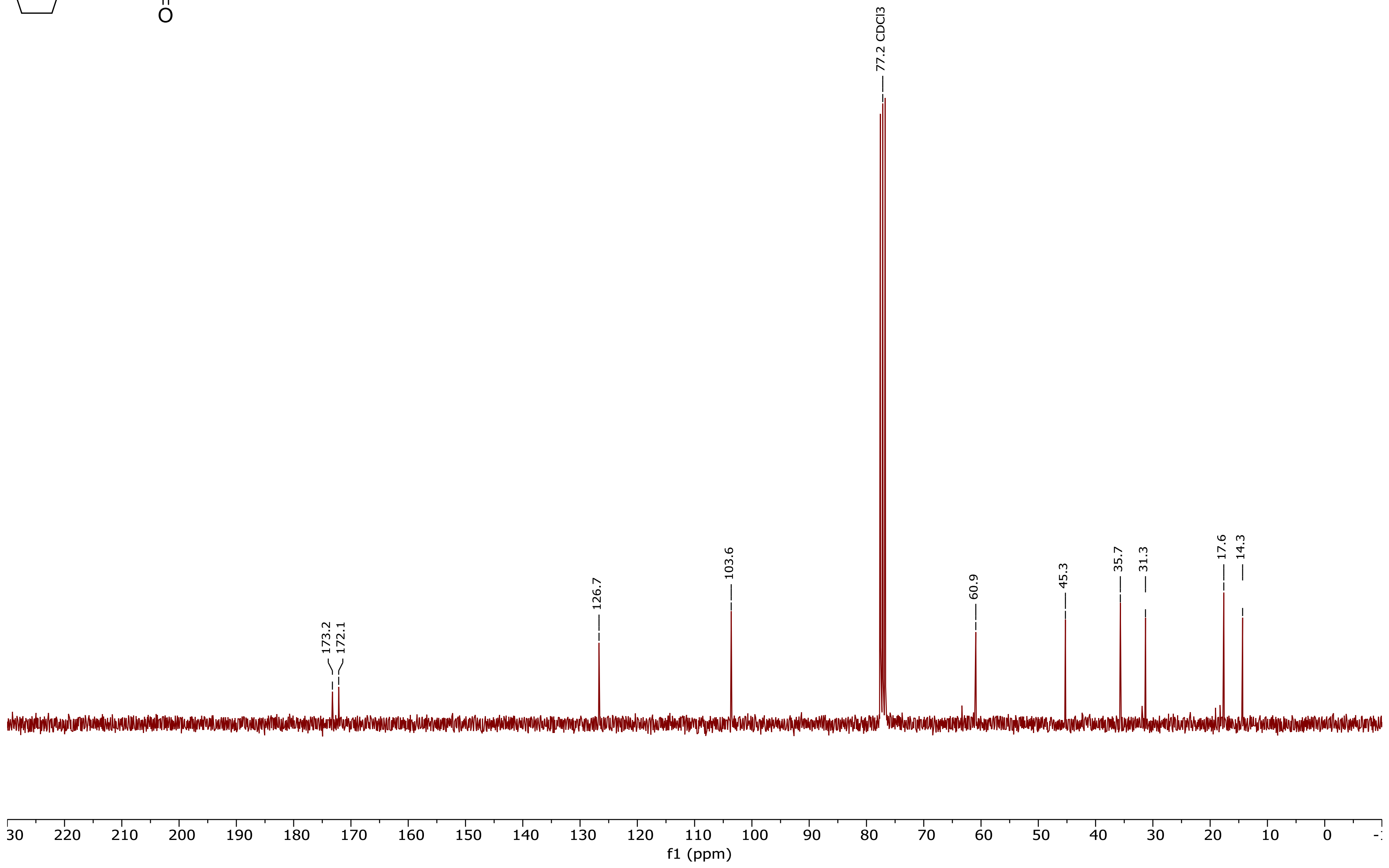

(*E*)-Phenyl 4-(2-oxopyrrolidin-1-yl) but-3-enoate (**46**)

^{1}H NMR (300 MHz, $CDCl_3$)

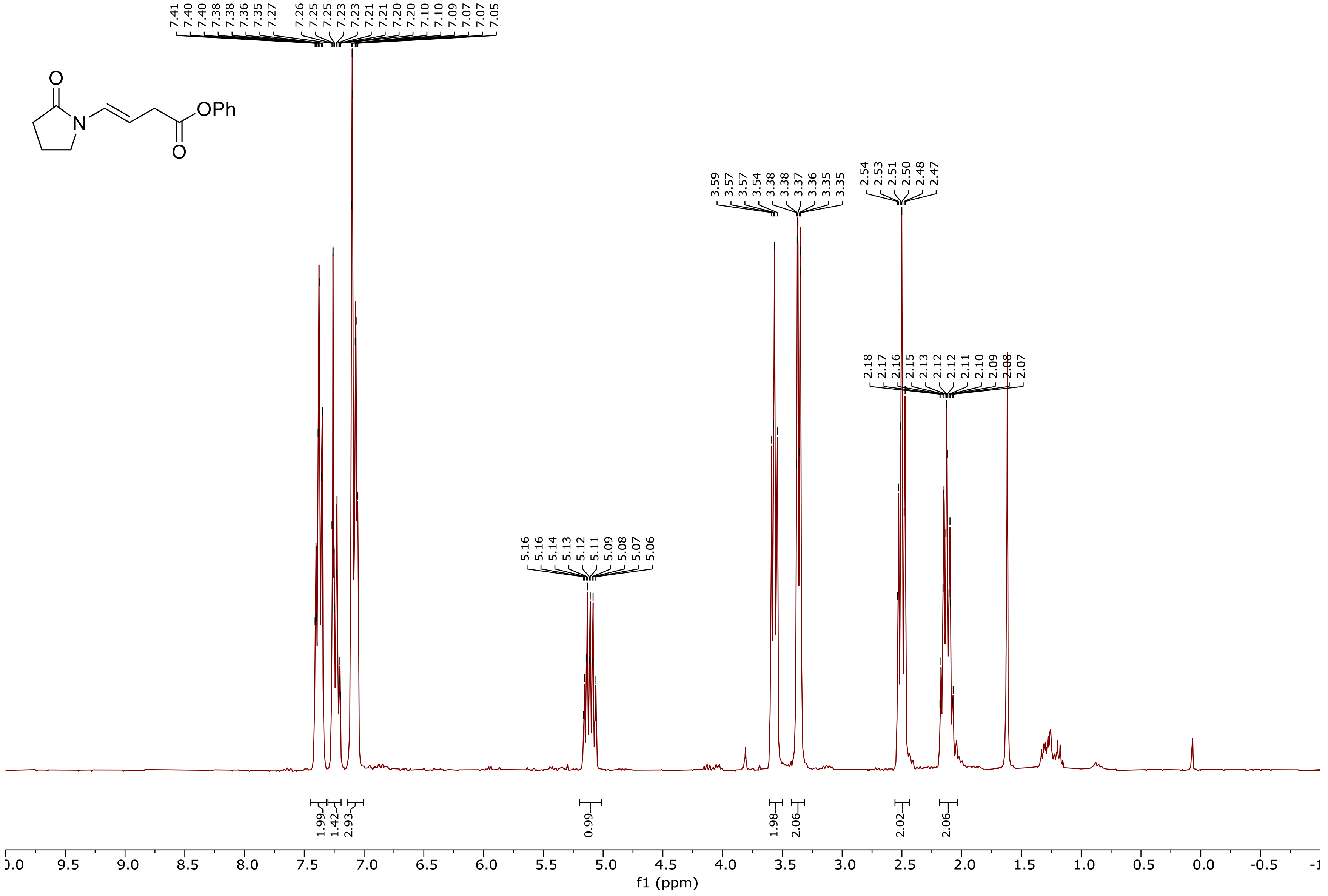

(*E*)-Phenyl 4-(2-oxopyrrolidin-1-yl) but-3-enoate (**46**)

^{13}C NMR (75 MHz, $CDCl_3$)

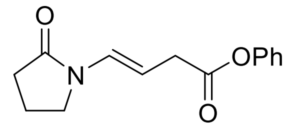

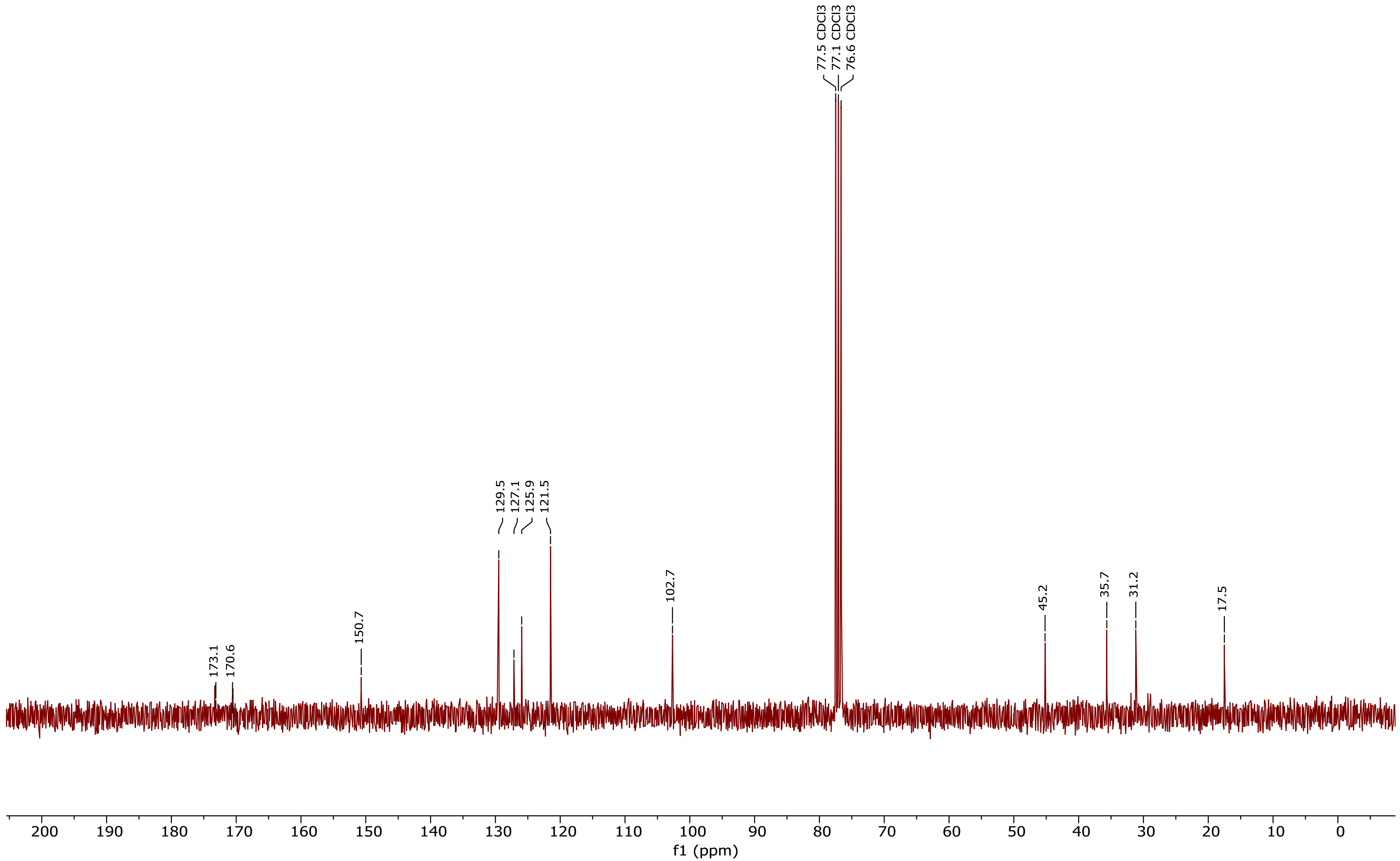

(*E*)-Ethyl 2-methyl-4-(2-oxopyrrolidin-1-yl)but-3-enoate (**47**)

^{1}H NMR (300 MHz, $CDCl_3$)

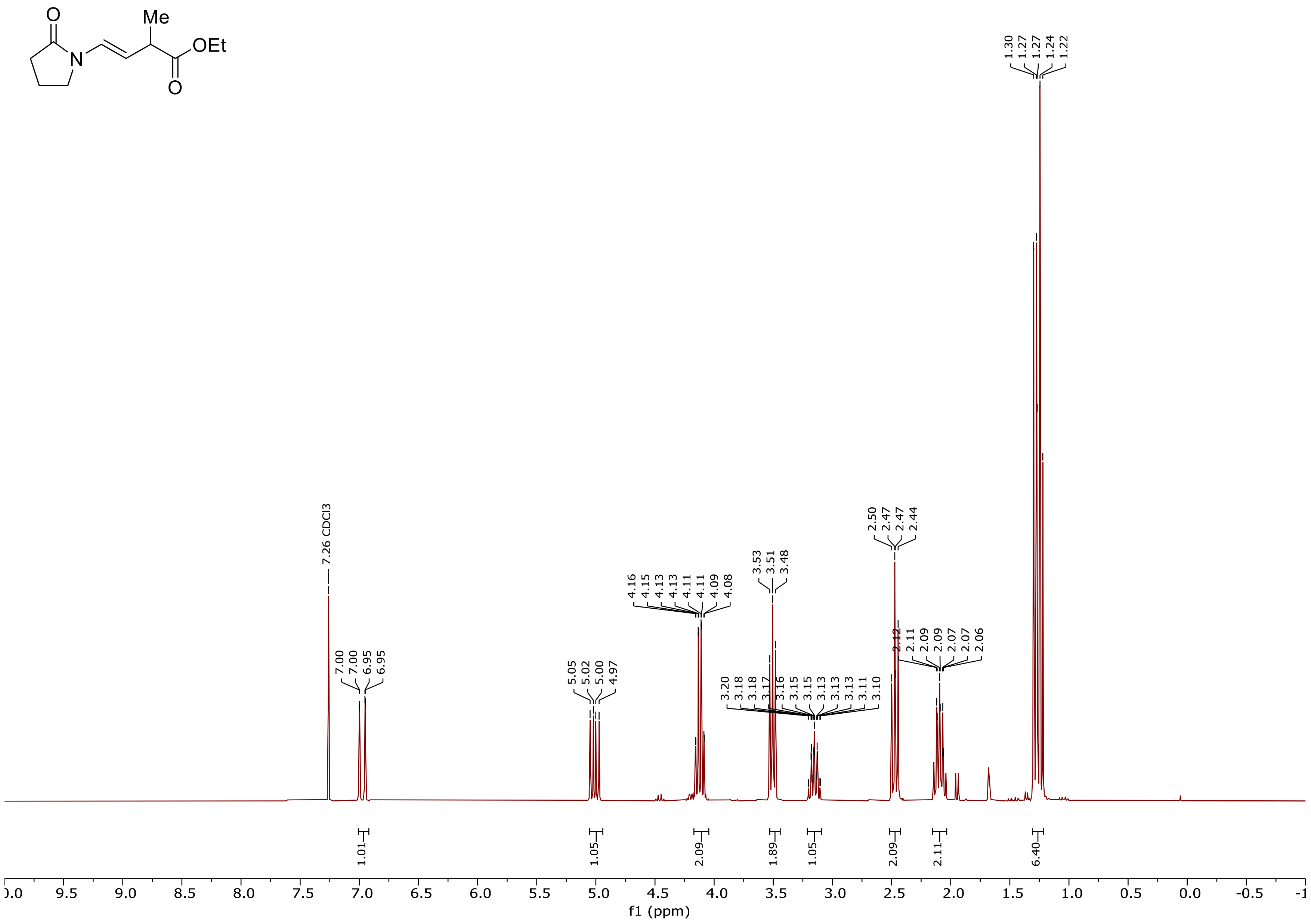

(*E*)-Ethyl 2-methyl-4-(2-oxopyrrolidin-1-yl)but-3-enoate (**47**)

^{13}C NMR (75 MHz, $CDCl_3$)

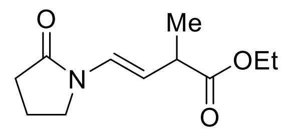

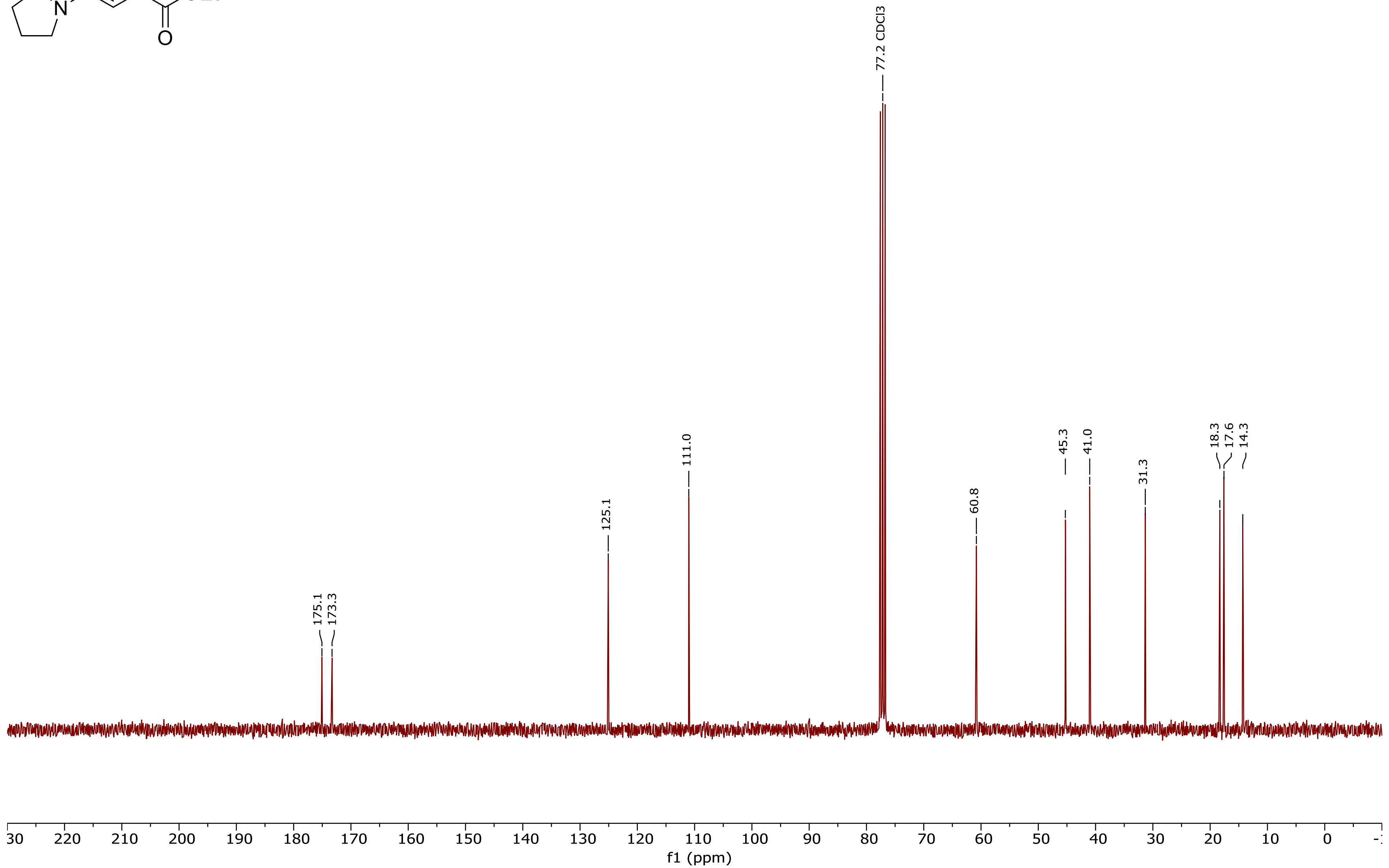

Ethyl 4-(2-oxopyrrolidin-1-yl) butanoate (**48**)

^{1}H NMR (300 MHz, $CDCl_3$)

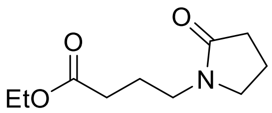

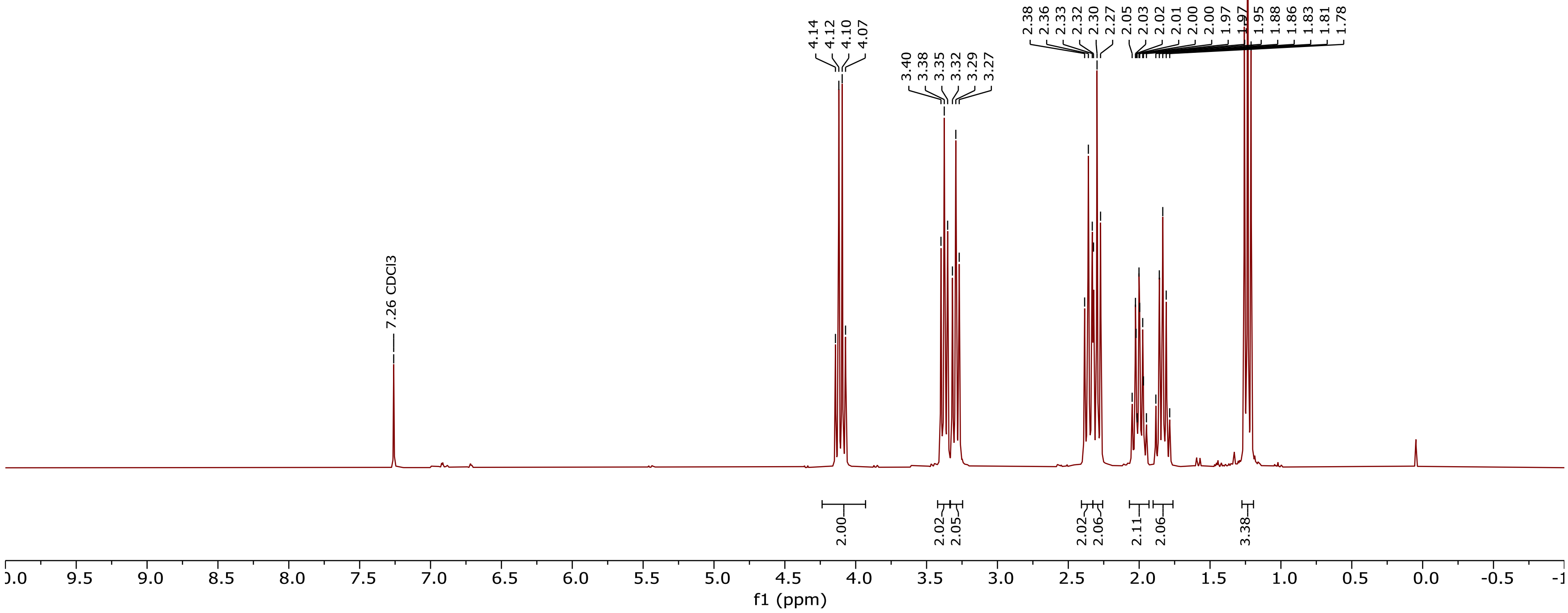

Ethyl 4-(2-oxopyrrolidin-1-yl) butanoate (**48**)

^{13}C NMR (75 MHz, $CDCl_3$)

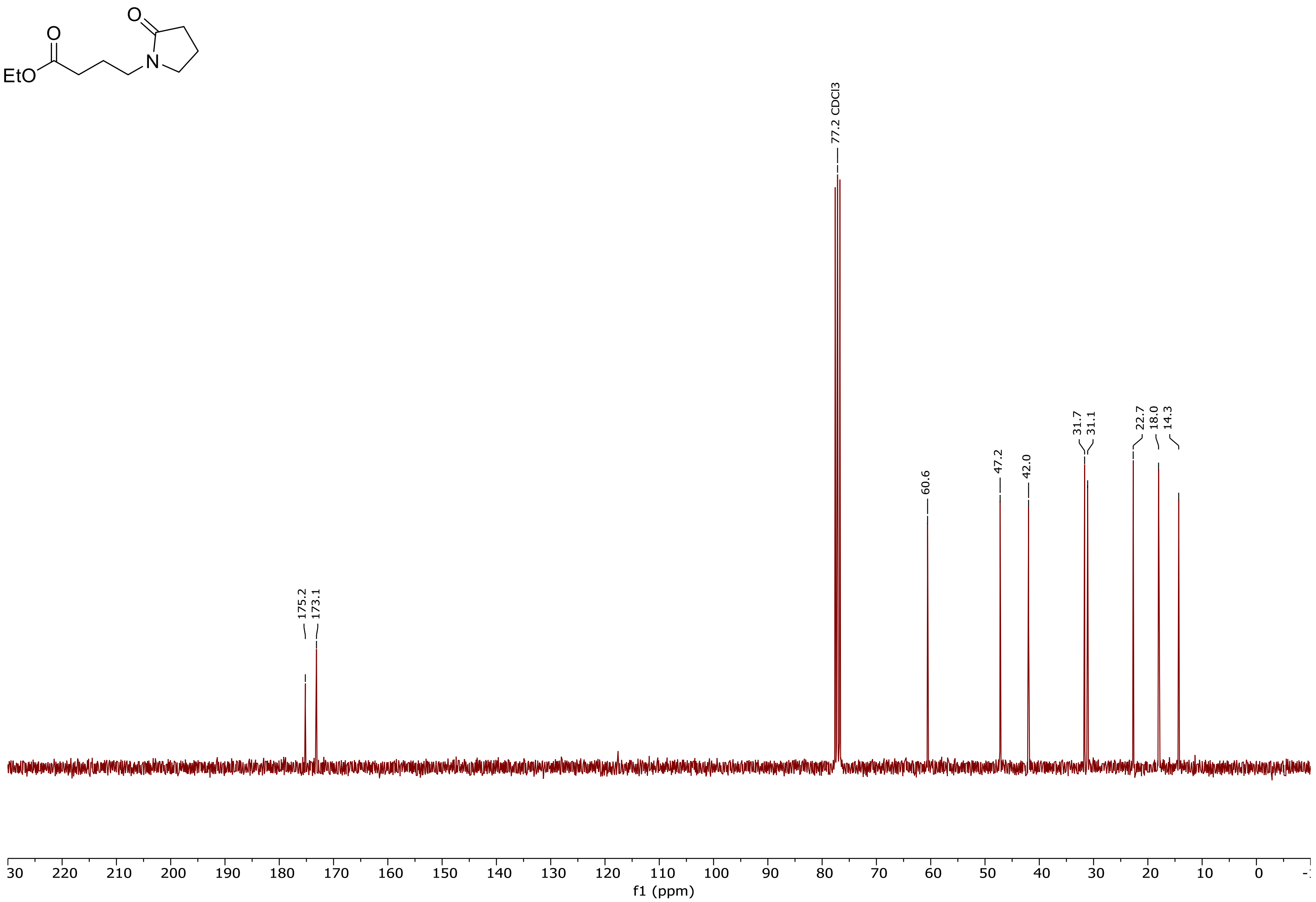

Benzyl 4-(2-oxopyrrolidin-1-yl)butanoate (**49**)

^{1}H NMR (300 MHz, $CDCl_3$)

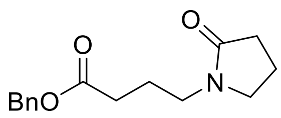

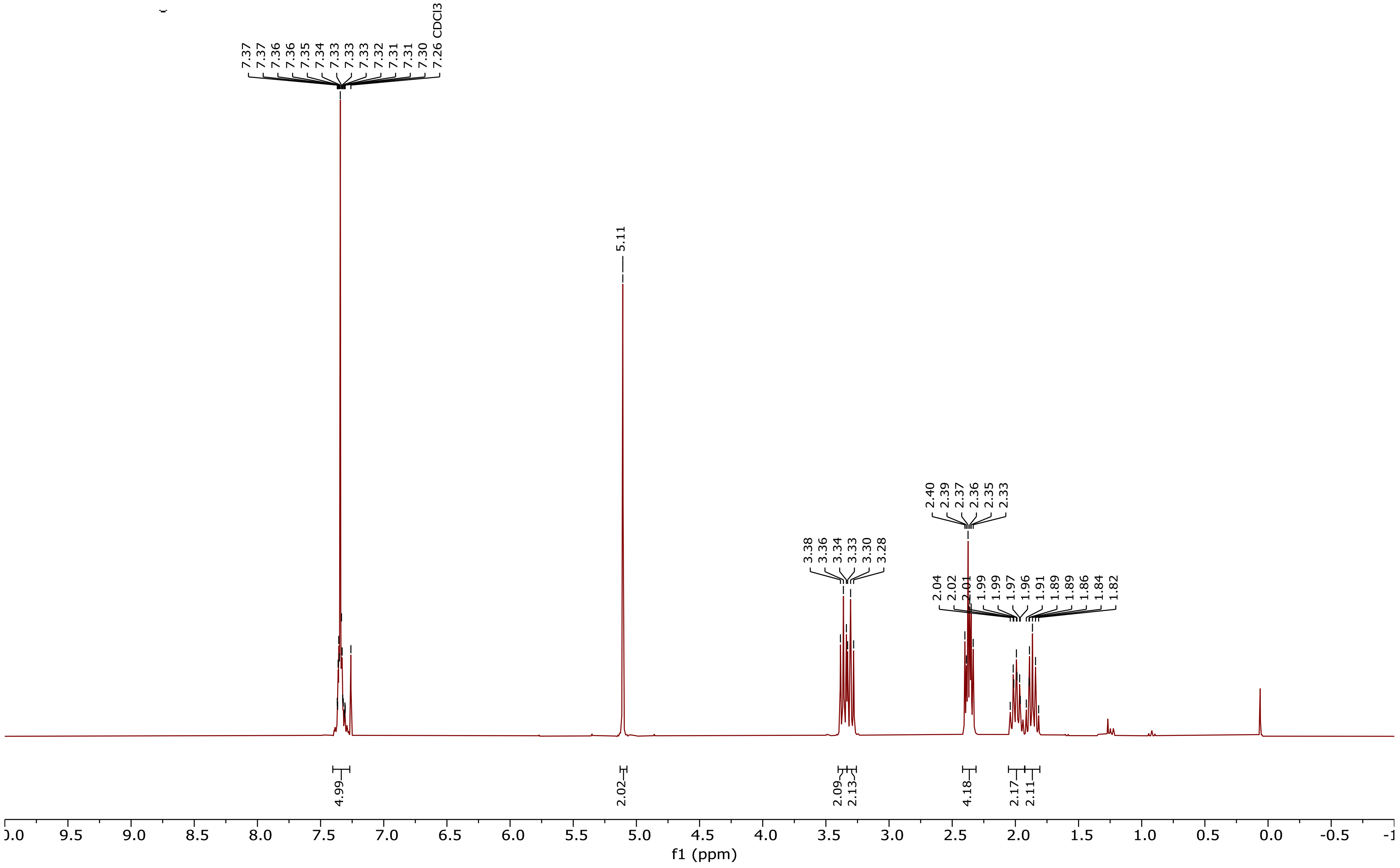

Benzyl 4-(2-oxopyrrolidin-1-yl)butanoate (**49**)

^{13}C NMR (75 MHz, $CDCl_3$)

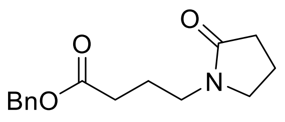

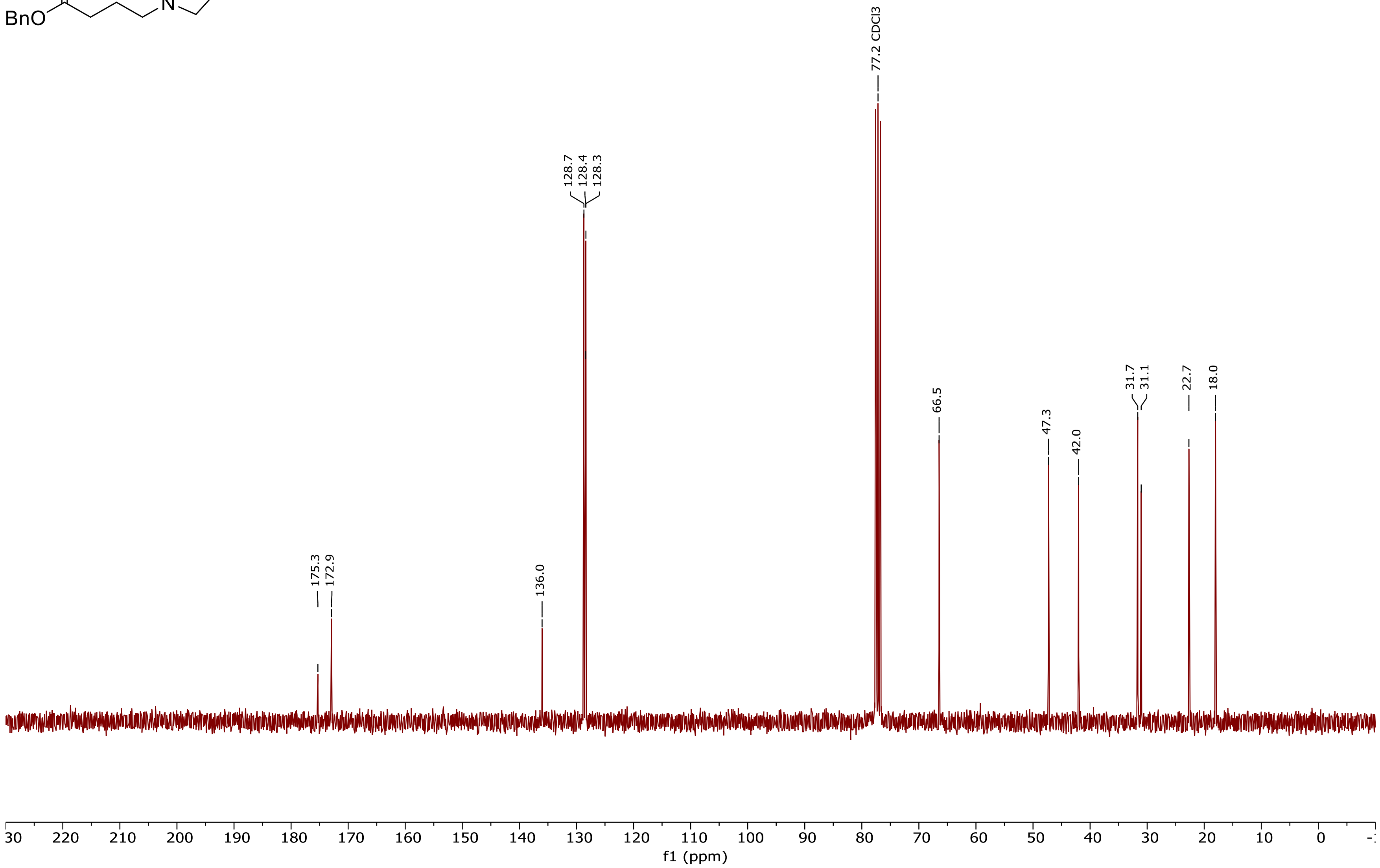

Phenyl 4-(2-oxopyrrolidin-1-yl)butanoate (**50**)

^{1}H NMR (300 MHz, $CDCl_3$)

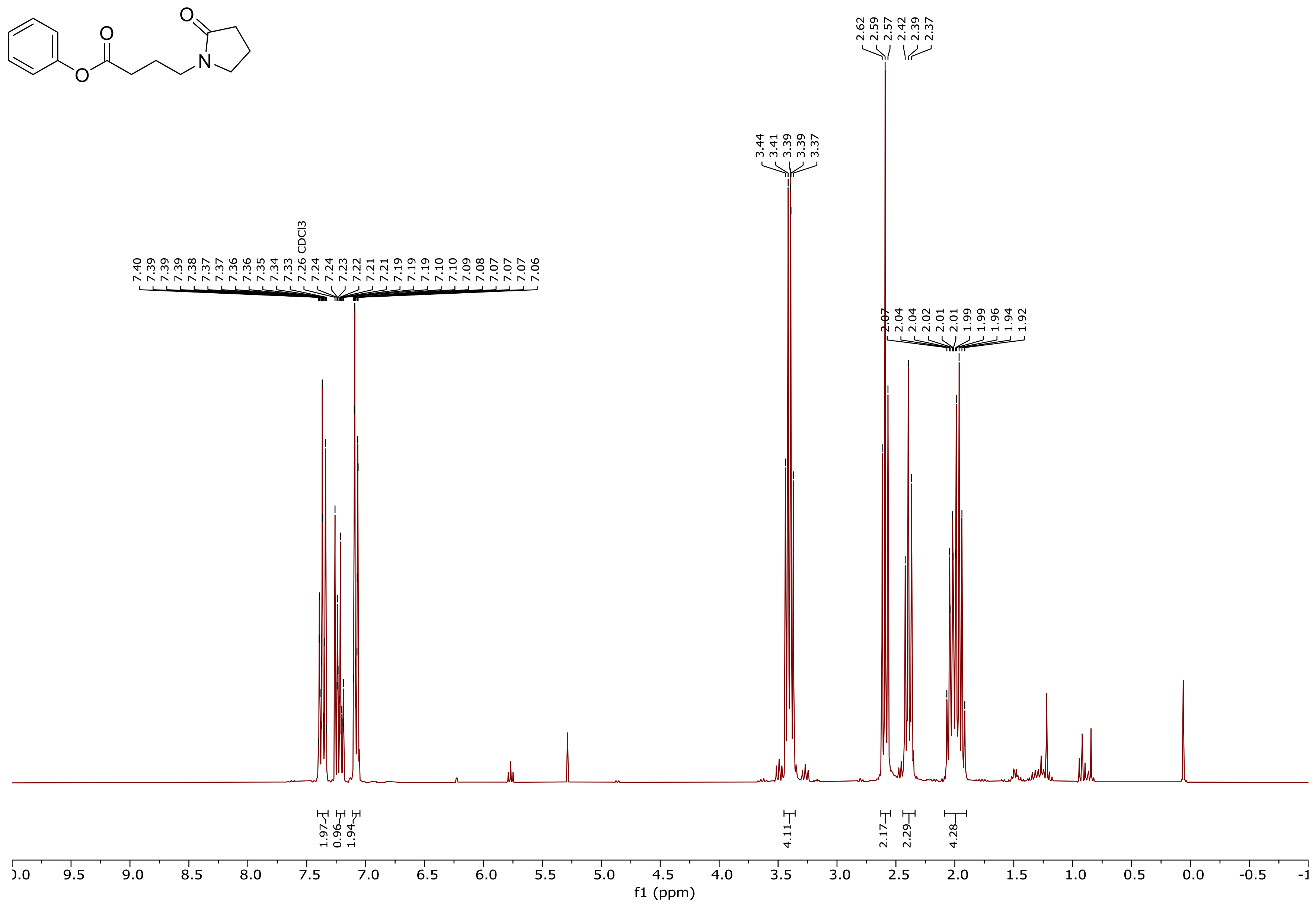

Phenyl 4-(2-oxopyrrolidin-1-yl)butanoate (**50**)

^{13}C NMR (75 MHz, $CDCl_3$)

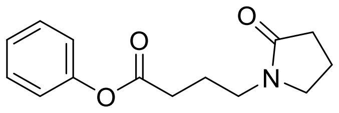

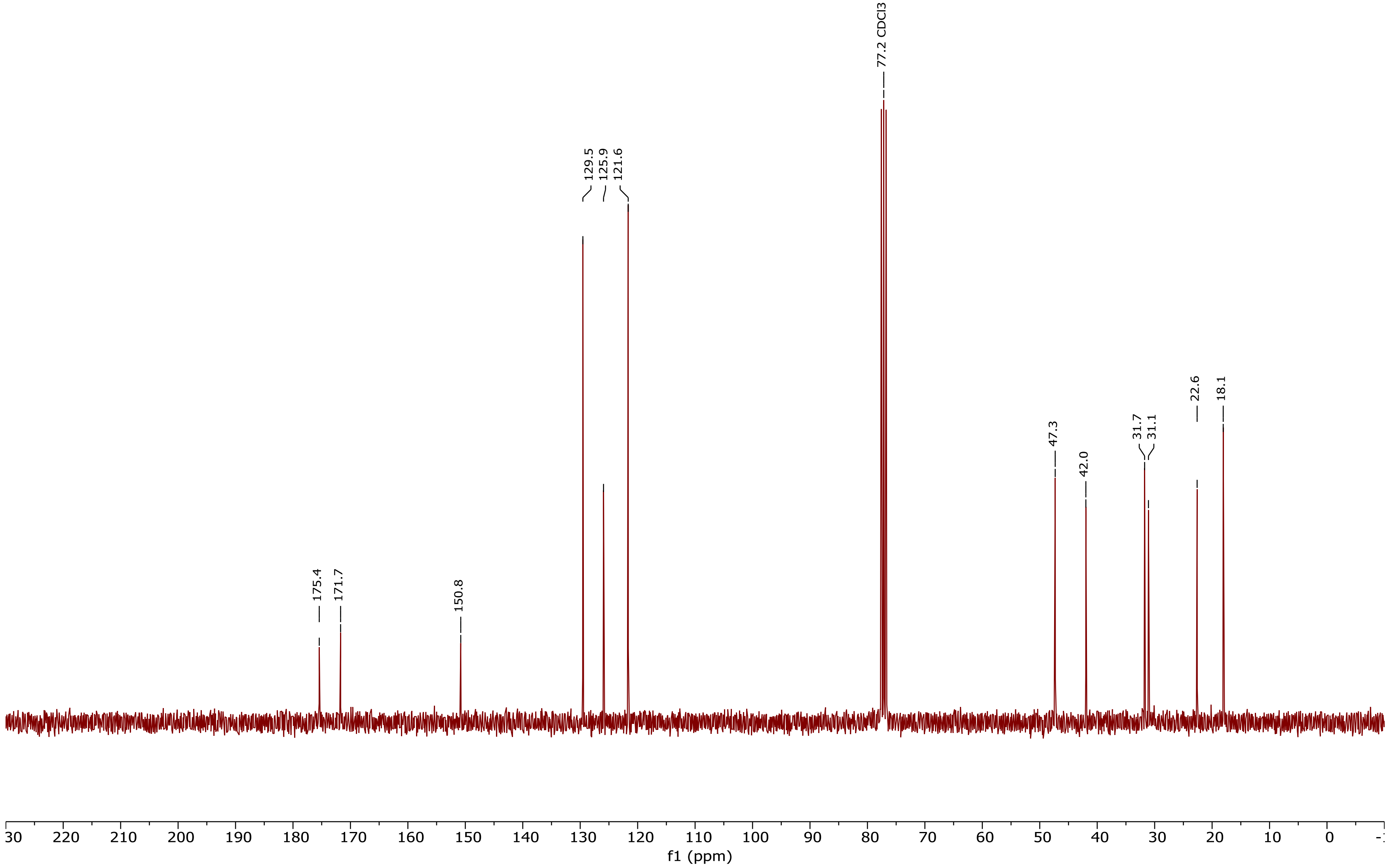

Benzyl 4-(*N*-phenylacetamido)butanoate (**51**)

^{1}H NMR (300 MHz, $CDCl_3$)

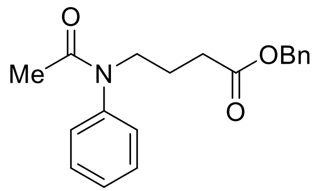

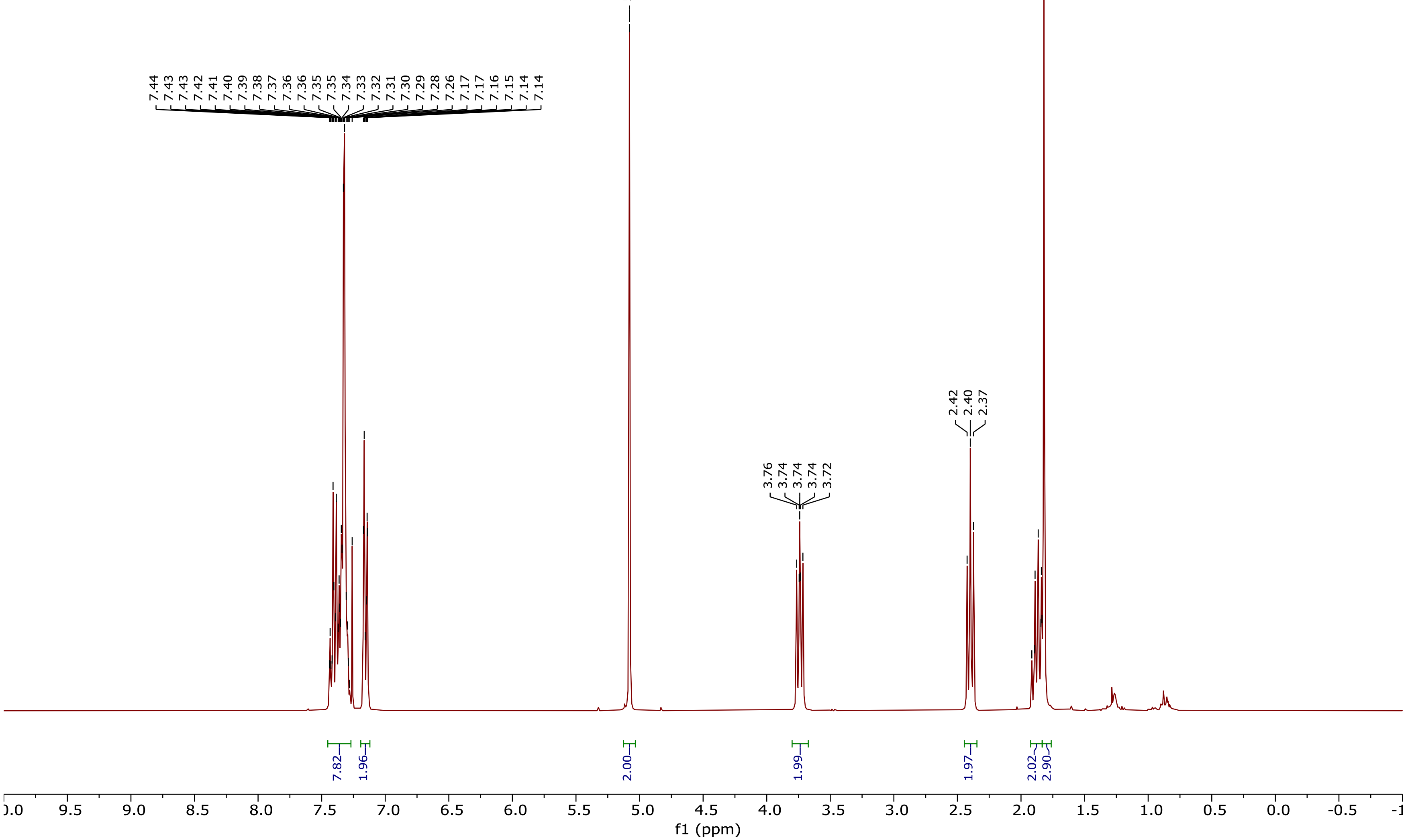

Benzyl 4-(*N*-phenylacetamido)butanoate (**51**)

^{13}C NMR (75 MHz, $CDCl_3$)

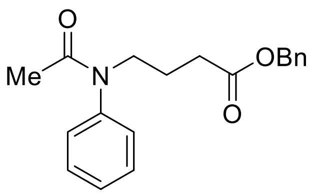

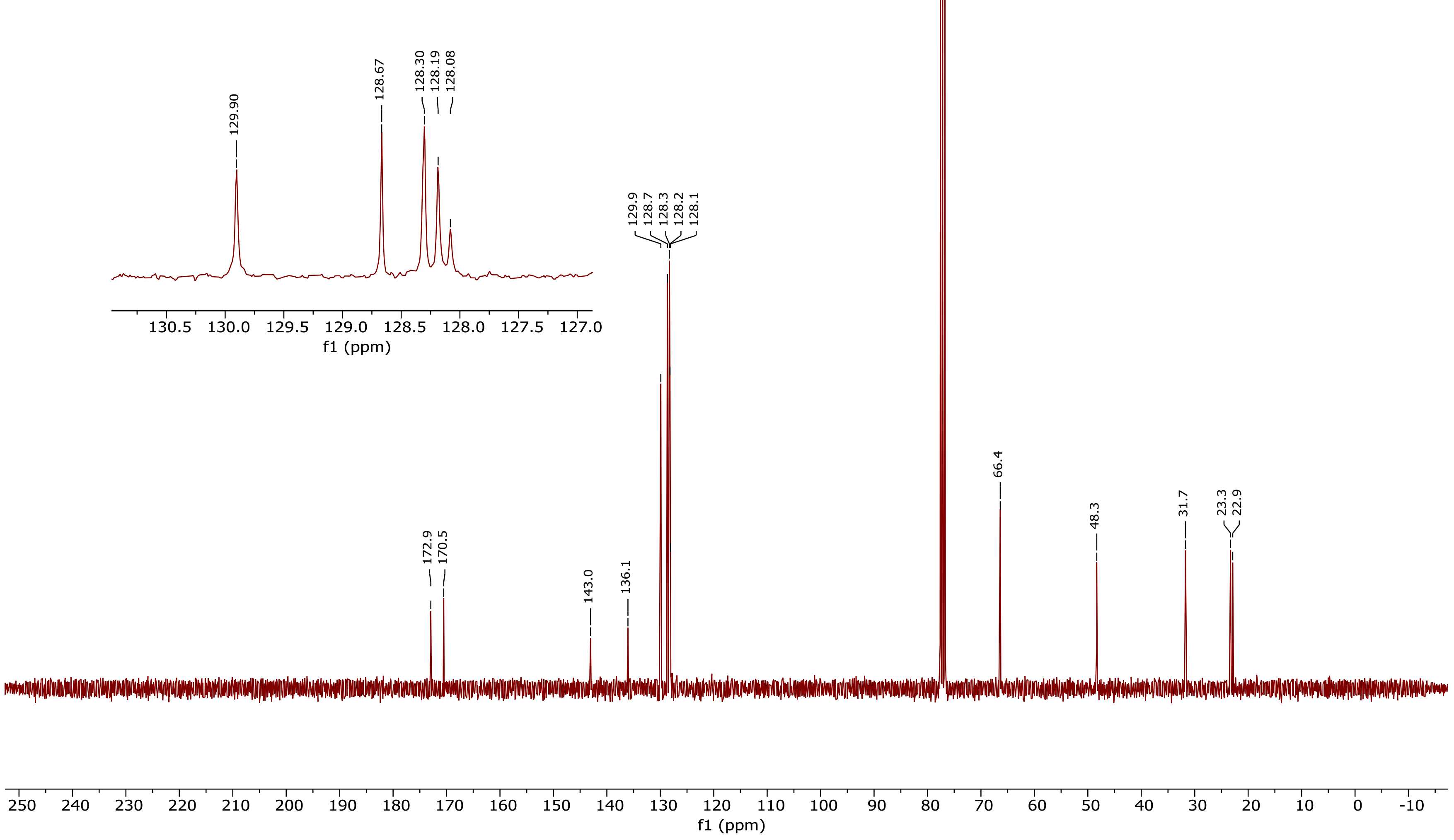

Diethyl 2-(2-(1,3-dioxoisoindolin-2-yl)ethyl)malonate (**52**)

^{1}H NMR (300 MHz, $CDCl_3$)

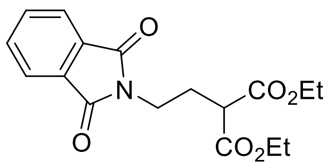

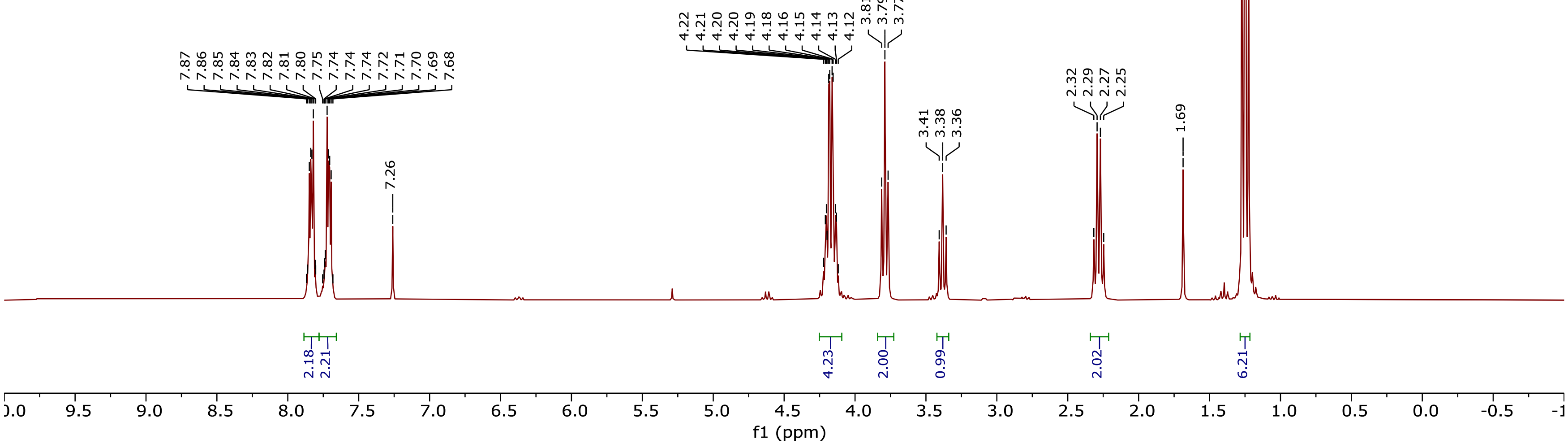

Diethyl 2-(2-(1,3-dioxoisoindolin-2-yl)ethyl)malonate (**52**)

^{13}C NMR (75 MHz, $CDCl_3$)

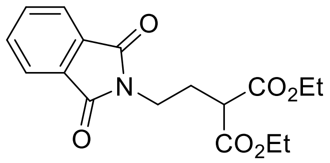

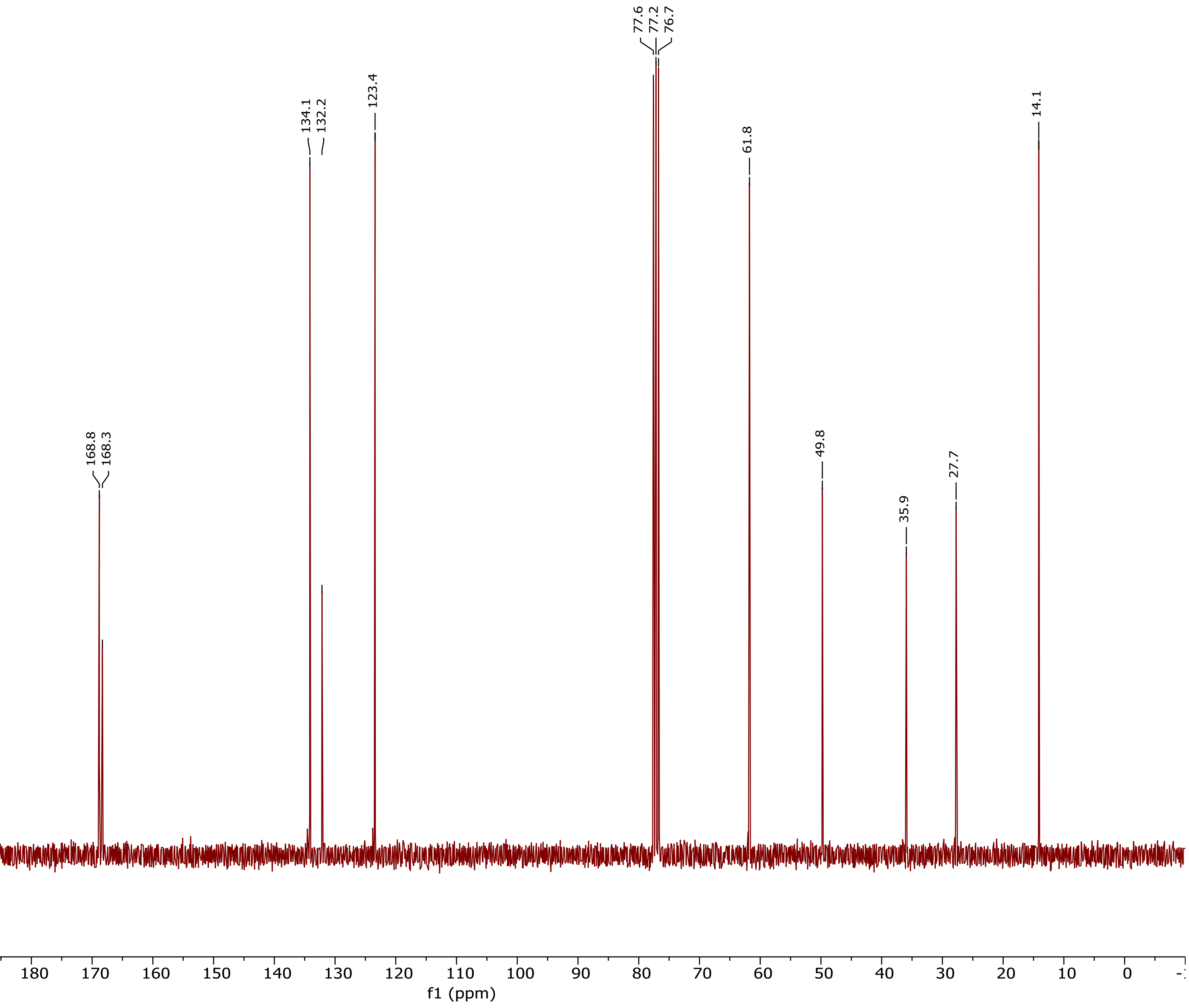

Benzyl 3-methyl-4-(2-oxopyrrolidin-1-yl)butanoate (**53**)

^{1}H NMR (300 MHz, $CDCl_3$)

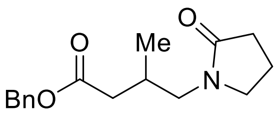

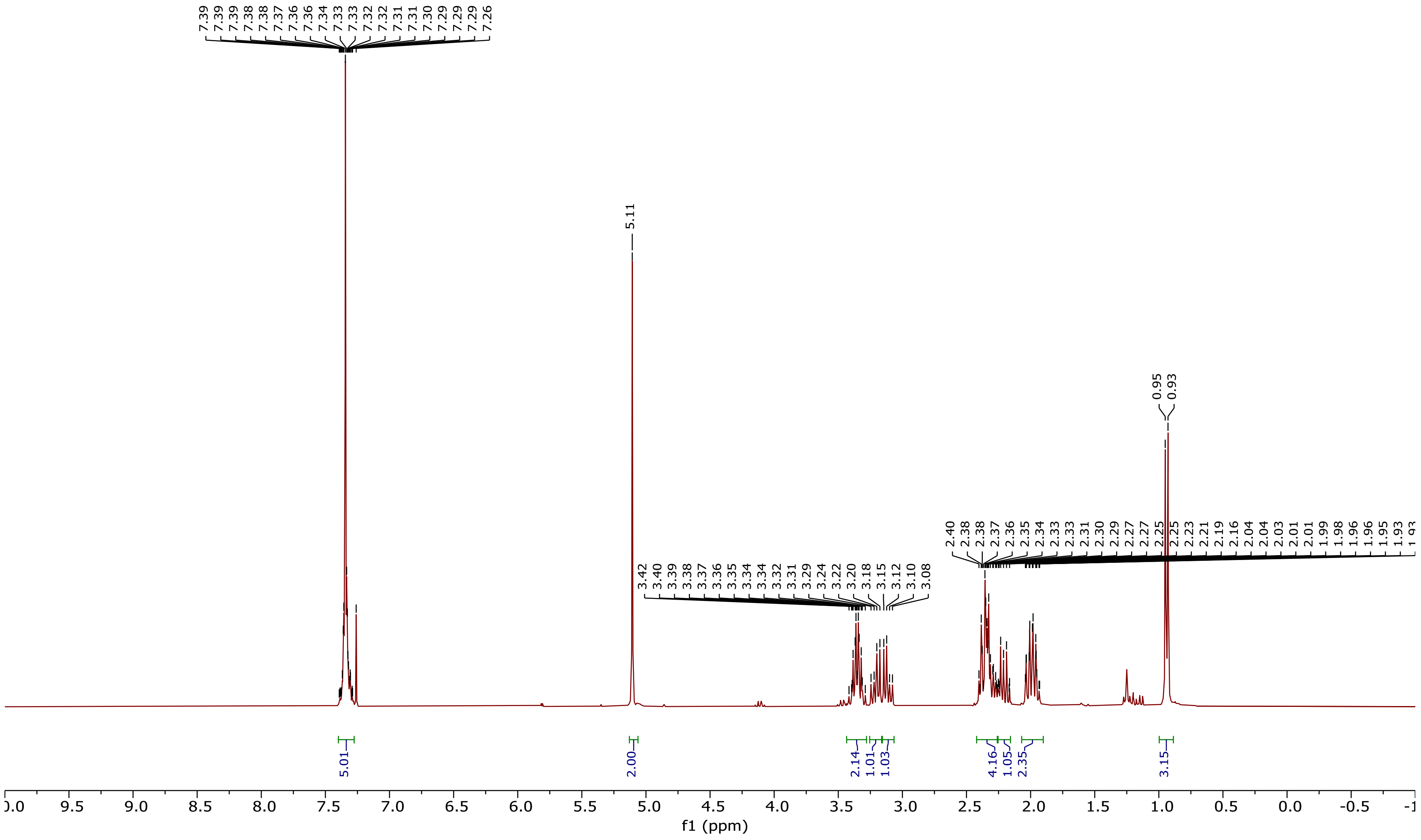

Benzyl 3-methyl-4-(2-oxopyrrolidin-1-yl)butanoate (**53**)

^{13}C NMR (75 MHz, $CDCl_3$)

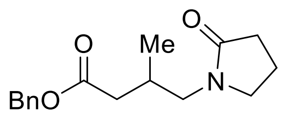

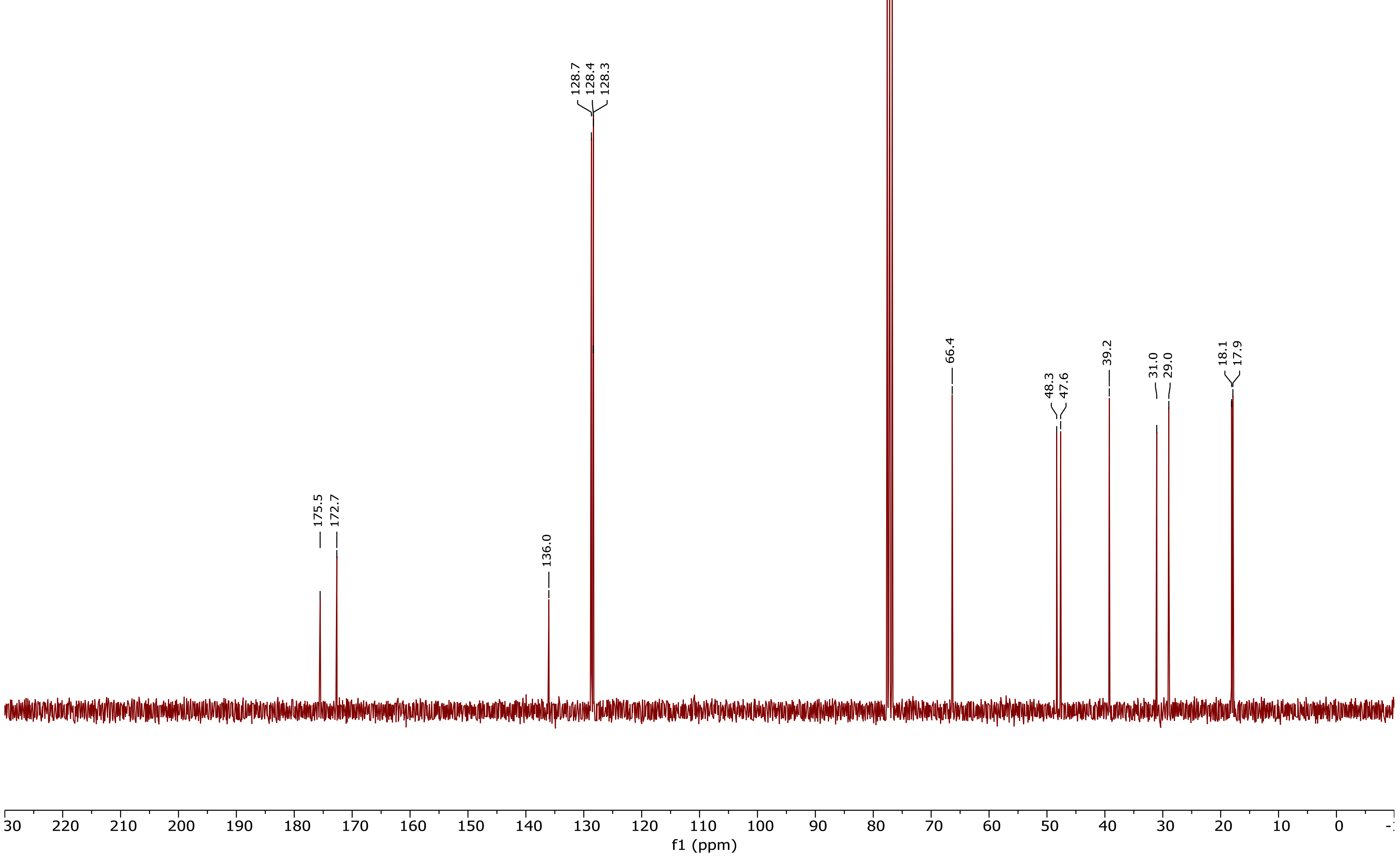

Benzyl 4-(2-oxooxazolidin-3-yl)butanoate (**54**)

^{1}H NMR (300 MHz, $CDCl_3$)

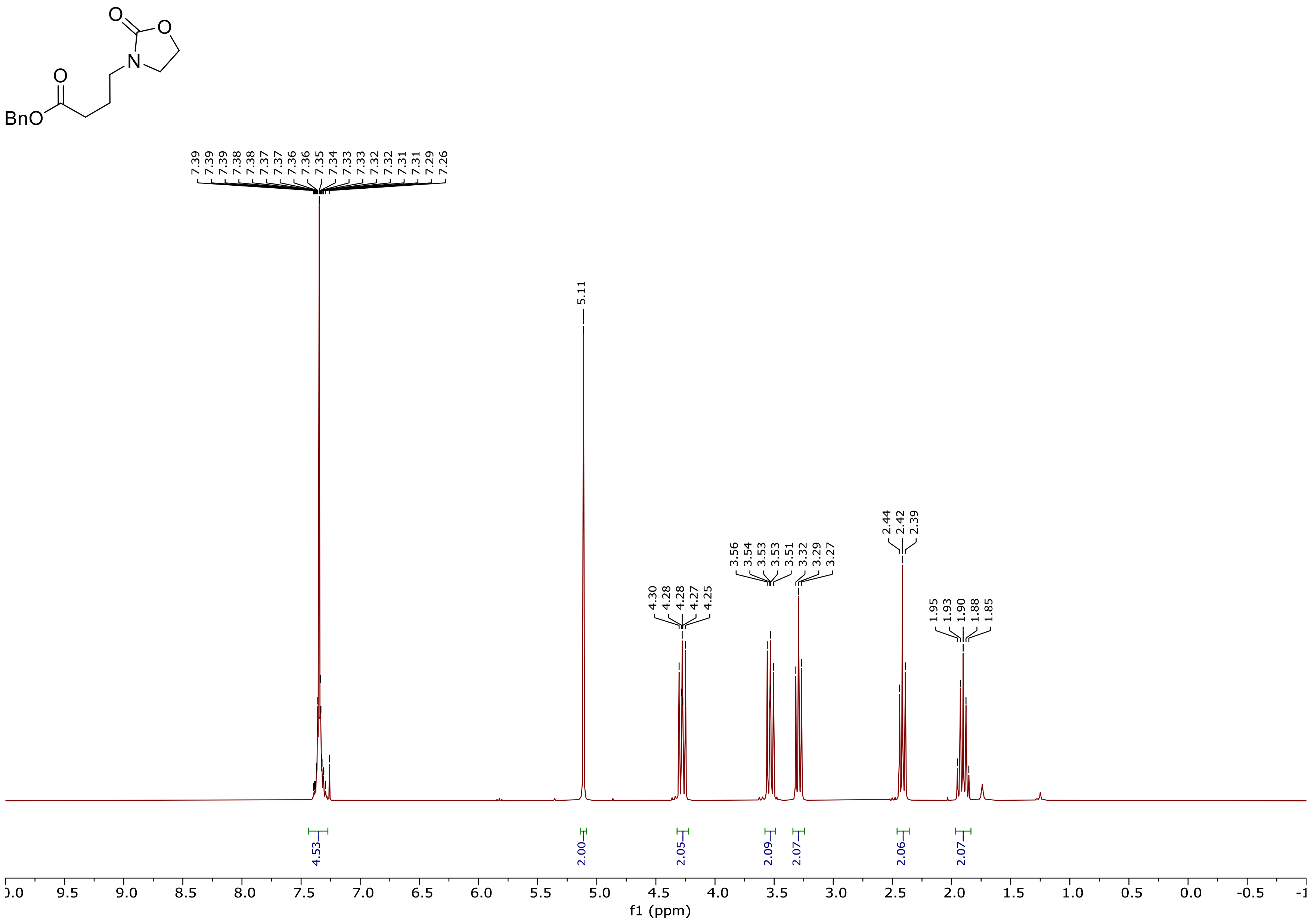

Benzyl 4-(2-oxooxazolidin-3-yl)butanoate (**54**)

^{13}C NMR (75 MHz, $CDCl_3$)

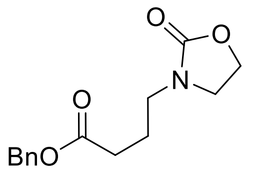

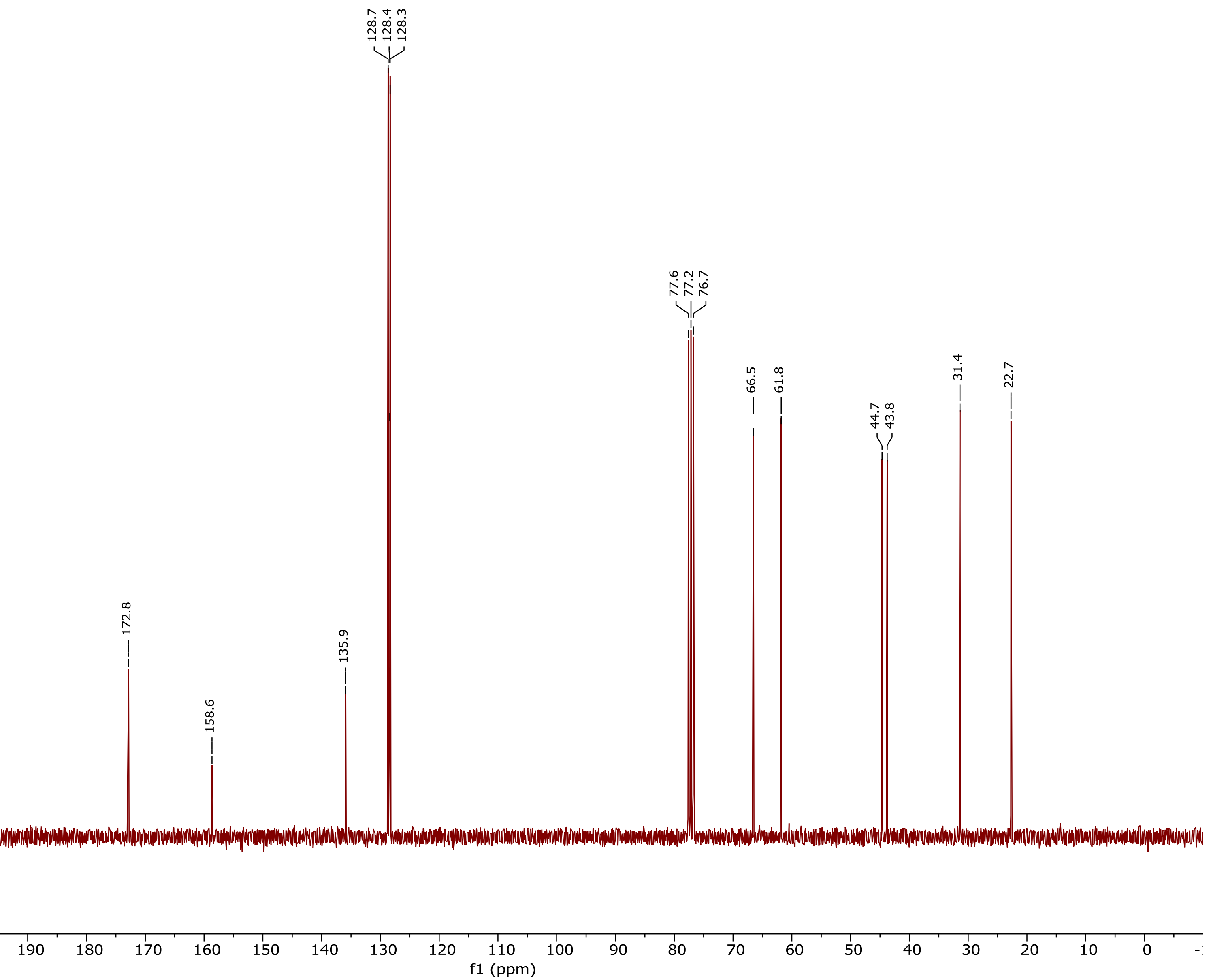

Benzyl 4-(3-acetyl-2-oxoimidazolidin-1-yl)butanoate (**55**)

^{1}H NMR (300 MHz, $CDCl_3$)

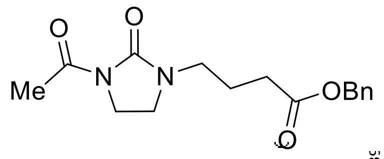

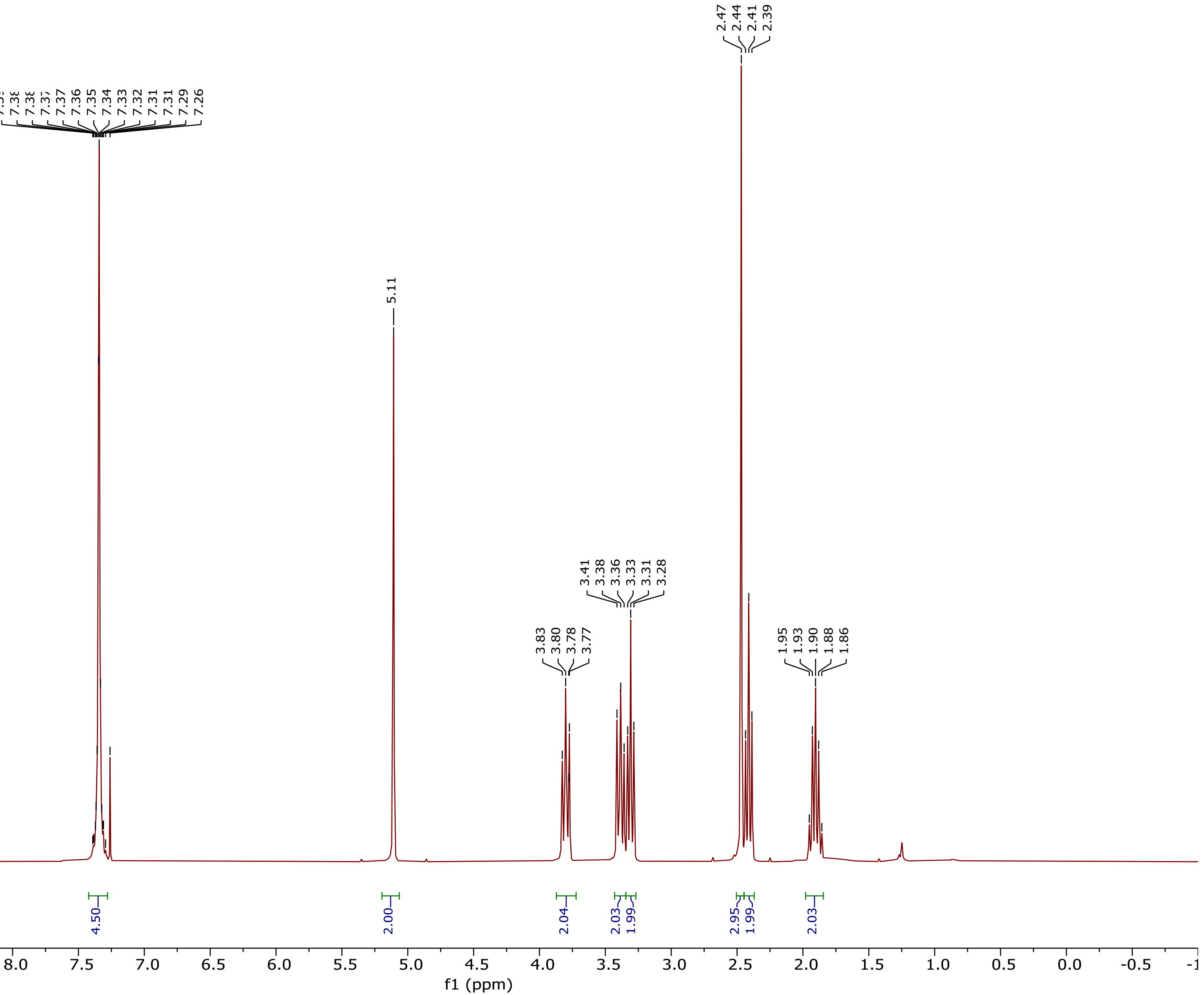

Benzyl 4-(3-acetyl-2-oxoimidazolidin-1-yl)butanoate (**55**)

^{13}C NMR (75 MHz, $CDCl_3$)

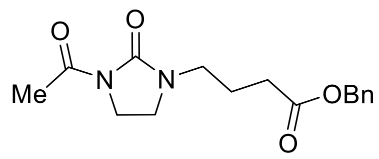

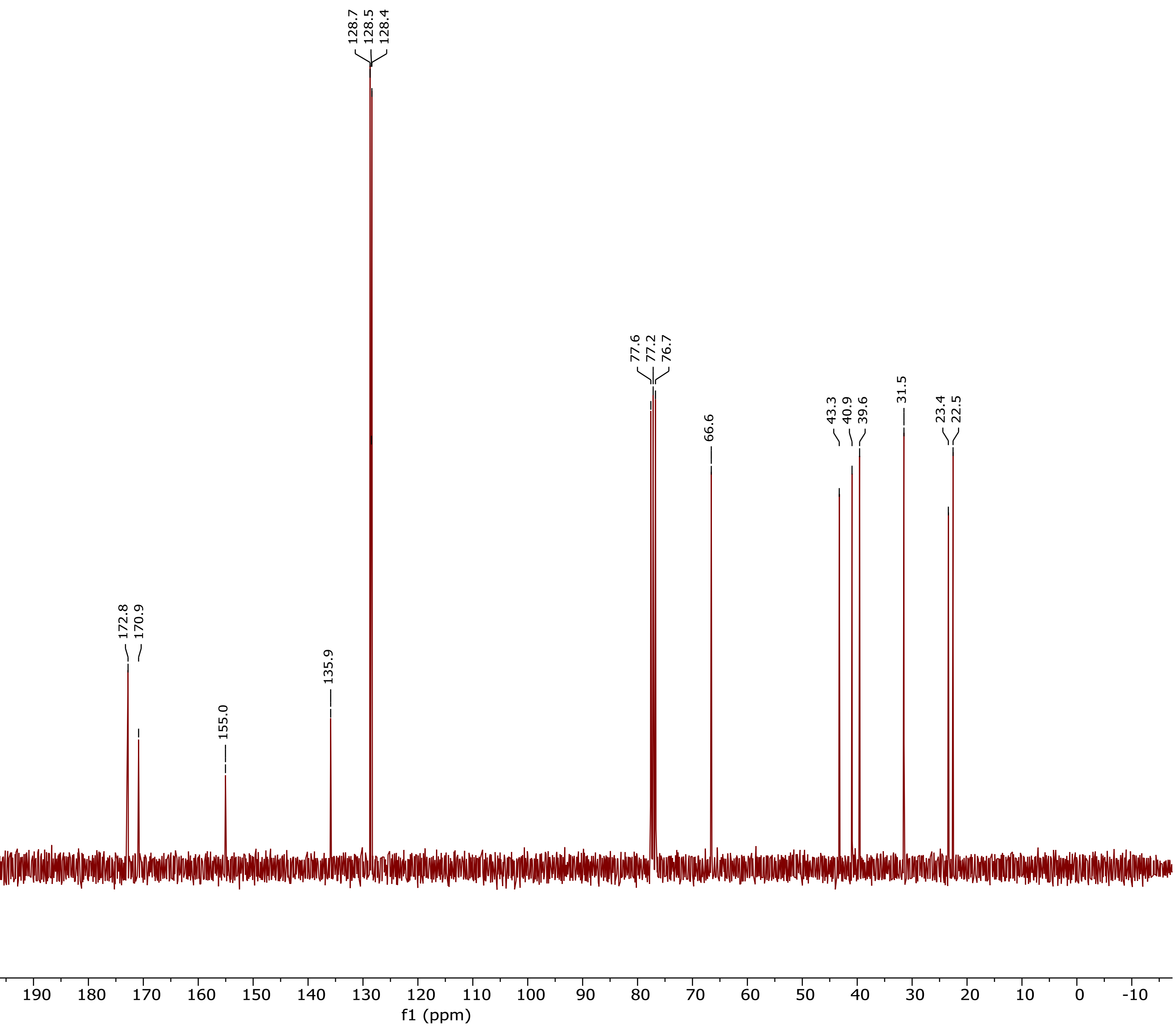

Benzyl 3-(2-(benzyloxy)-2-oxoethyl)pyrrolidine-1-carboxylate (**56**)

^{1}H NMR (300 MHz, $CDCl_3$)

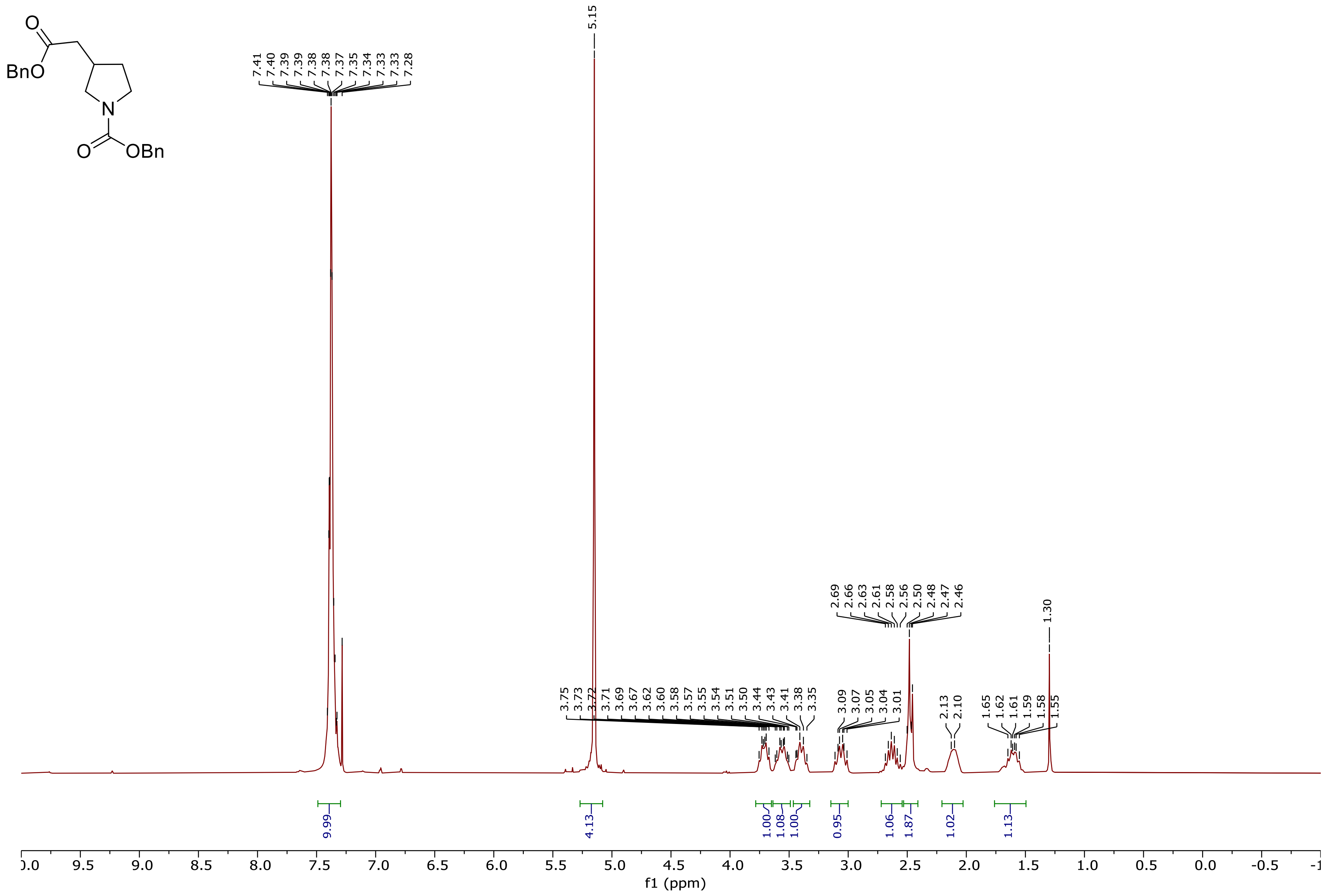

Benzyl 3-(2-(benzyloxy)-2-oxoethyl)pyrrolidine-1-carboxylate (**56**) ^{13}C NMR (75 MHz, $CDCl_3$)

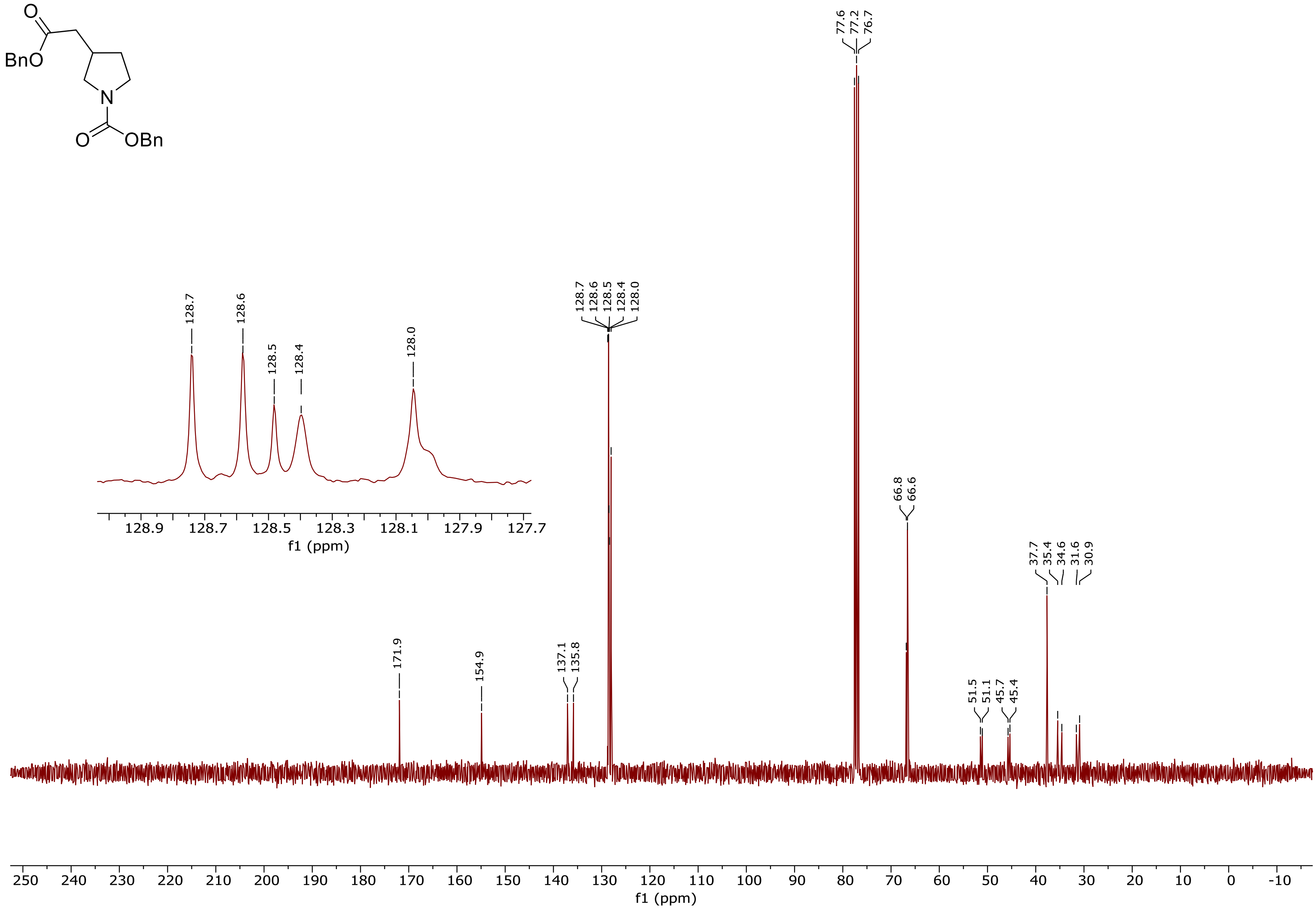

Benzyl 3-(2-(benzyloxy)-2-oxoethyl)piperidine-1-carboxylate (**57**)

^{1}H NMR (300 MHz, $CDCl_3$)

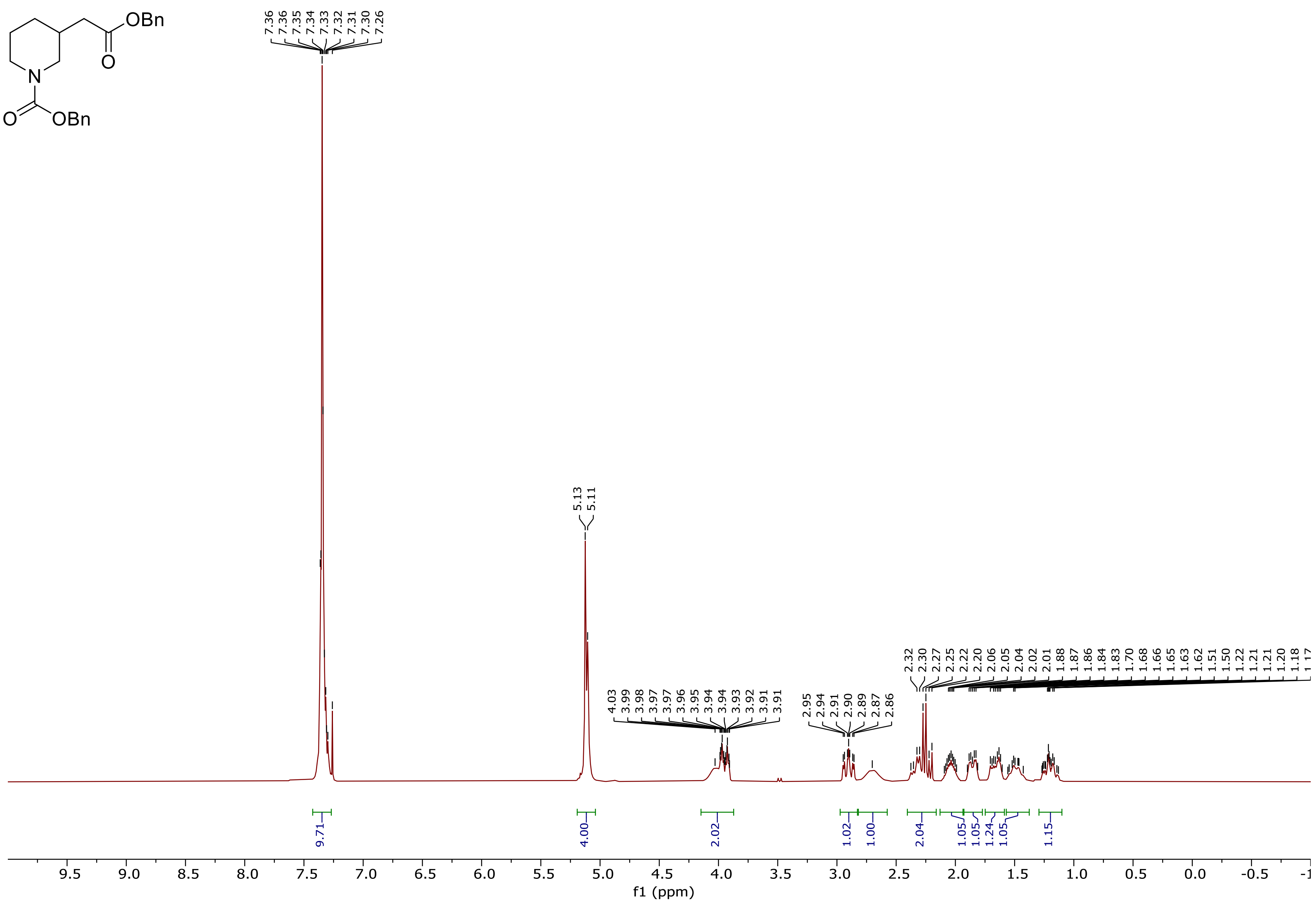

Benzyl 3-(2-(benzyloxy)-2-oxoethyl)piperidine-1-carboxylate (**57**) ^{13}C NMR (75 MHz, $CDCl_3$)

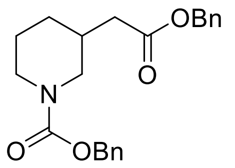

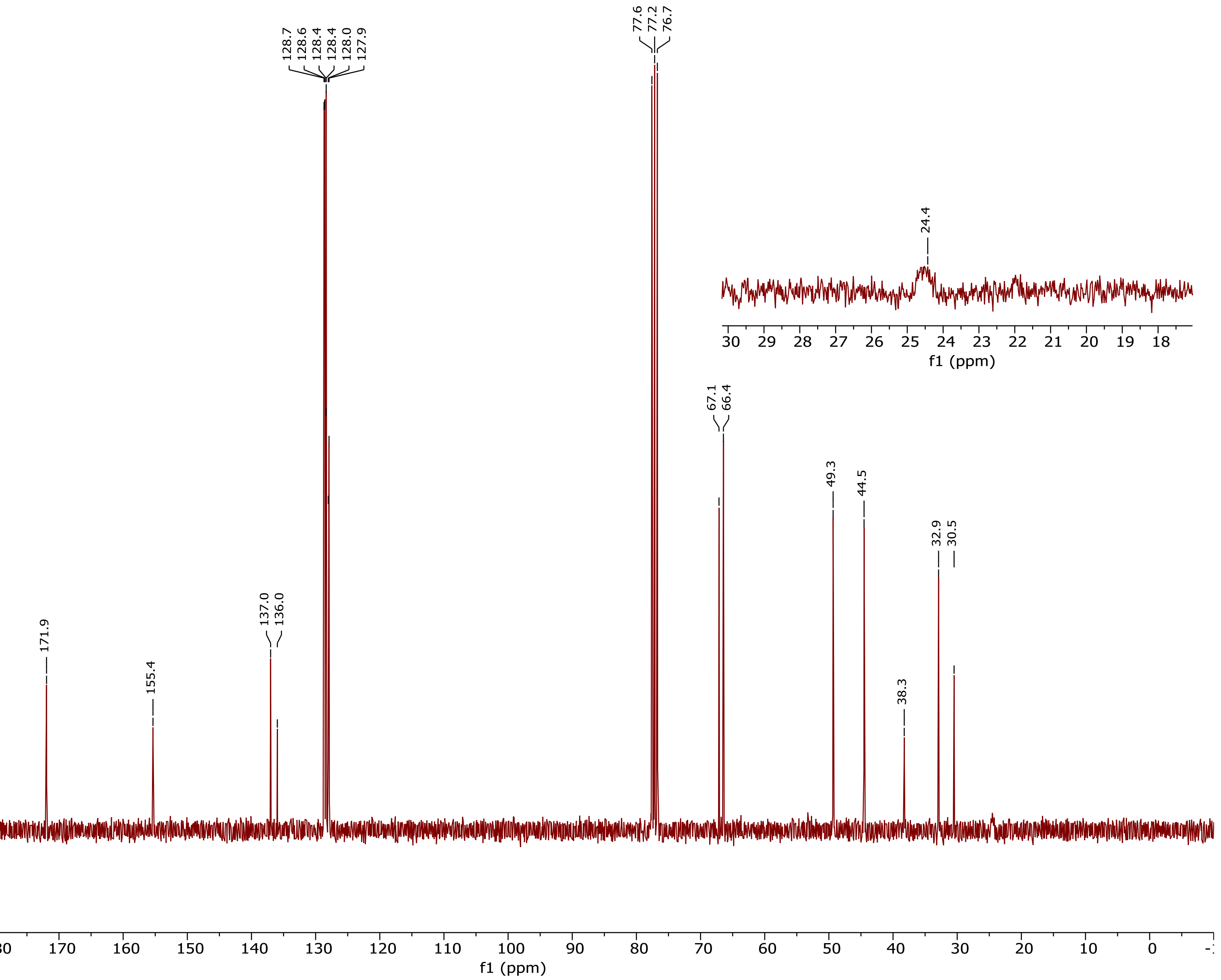

Benzyl 3-((2-oxooxazolidin-3-yl)methyl)heptanoate (**58**)

^1H NMR (300 MHz, $CDCl_3$)

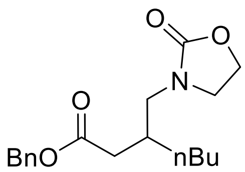

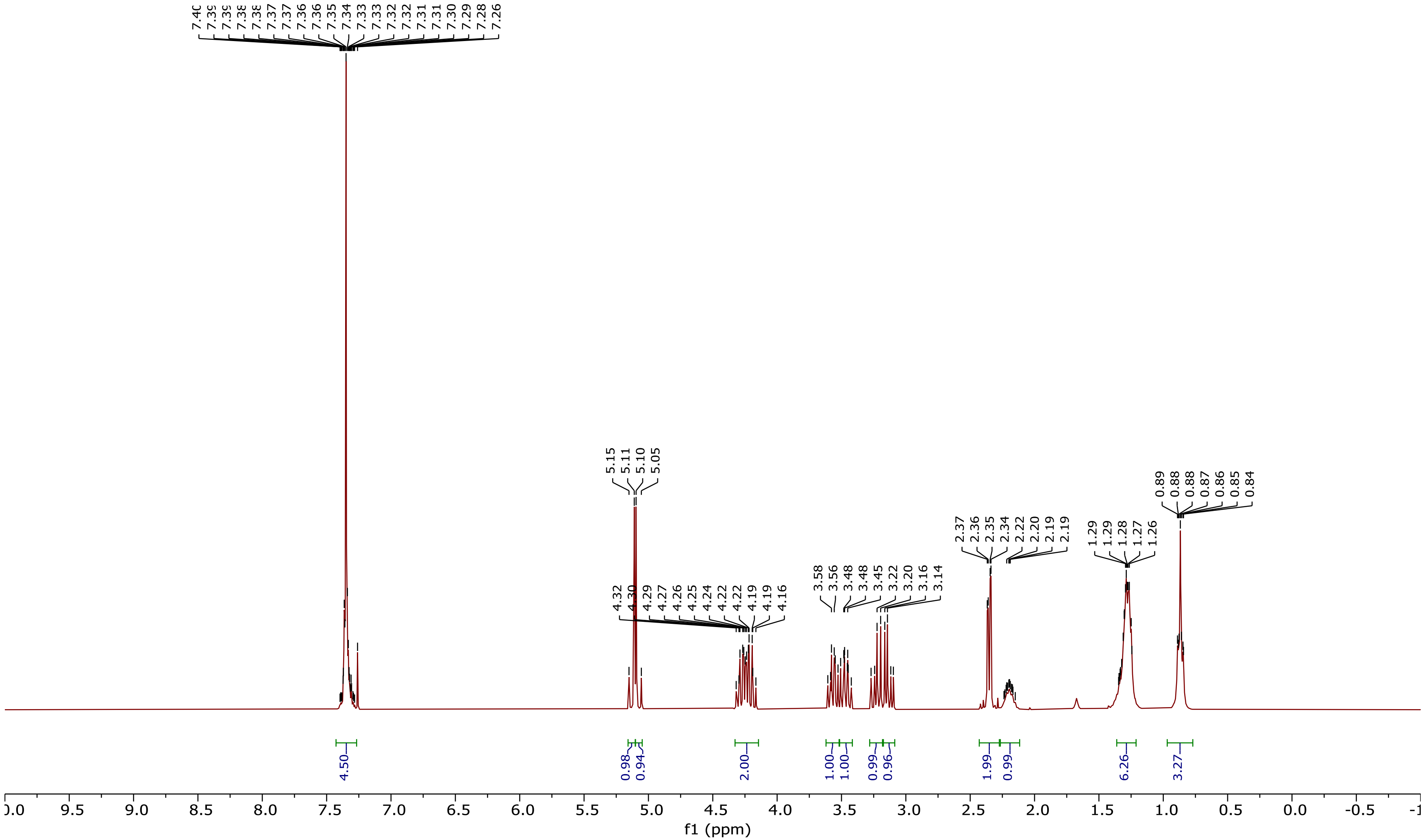

Benzyl 3-((2-oxooxazolidin-3-yl)methyl)heptanoate (**58**)

^{13}C NMR (75 MHz, $CDCl_3$)

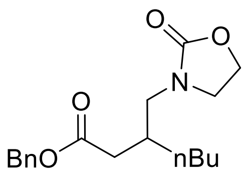

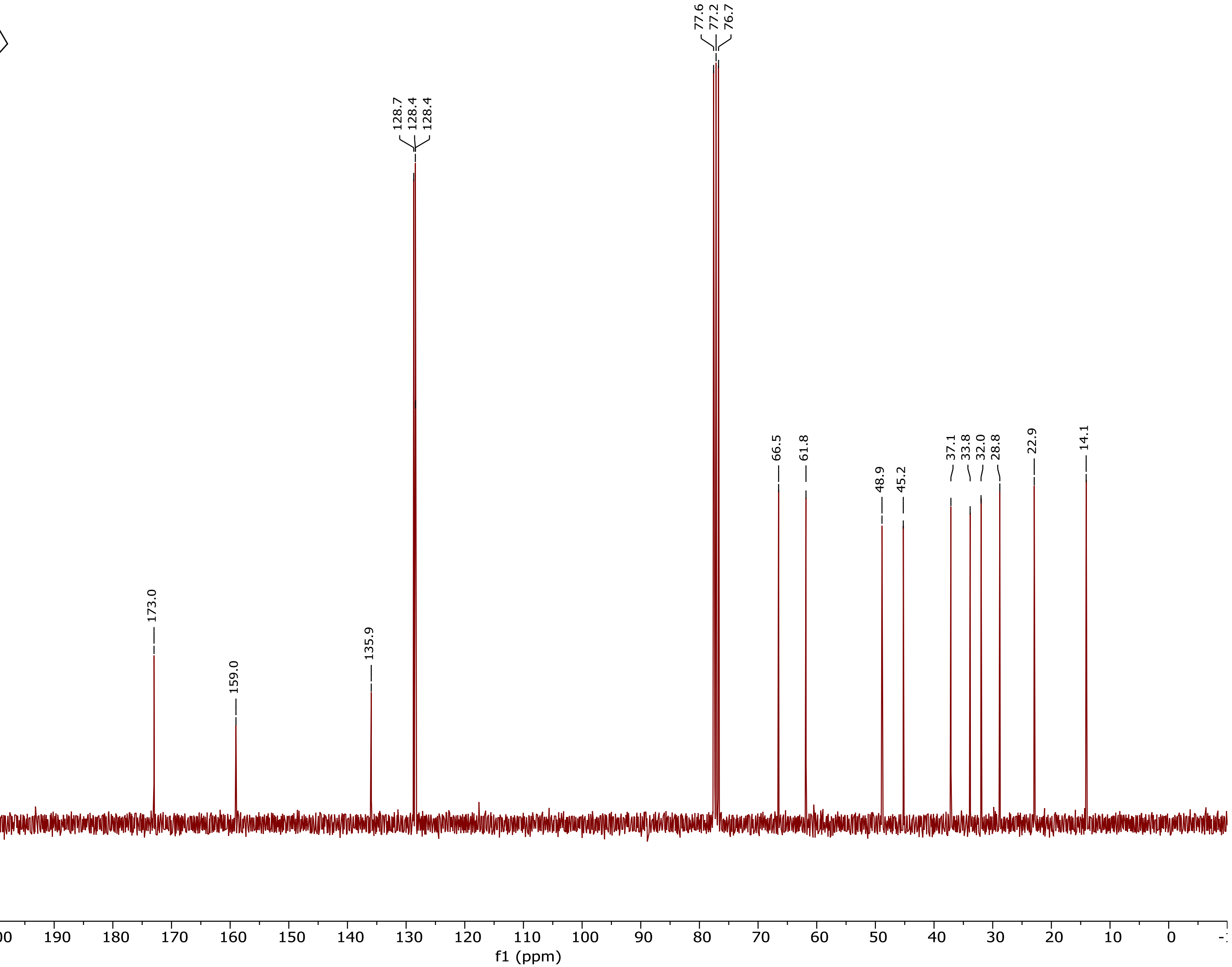

Benzyl 3-methyl-4-(2-oxooxazolidin-3-yl)pentanoate (**59**)
1st diastereoisomer

^{1}H NMR (300 MHz, $CDCl_3$)

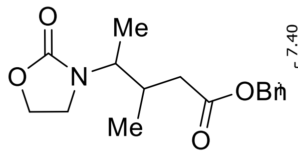

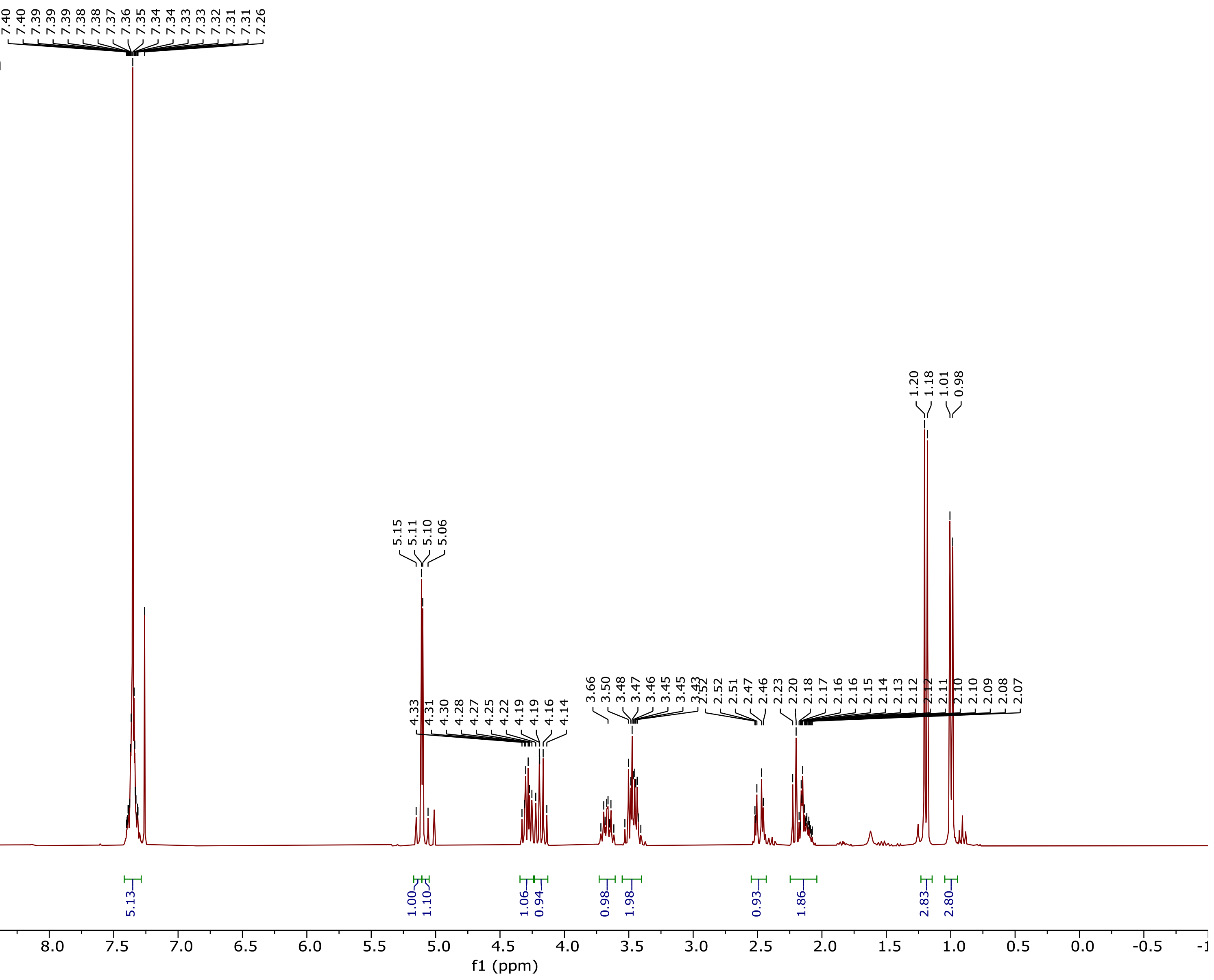

Benzyl 3-methyl-4-(2-oxooxazolidin-3-yl)pentanoate (**59**) ^{13}C NMR (75 MHz, $CDCl_3$)

1st diastereoisomer

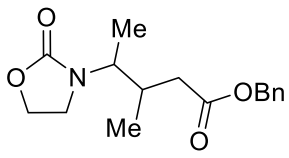

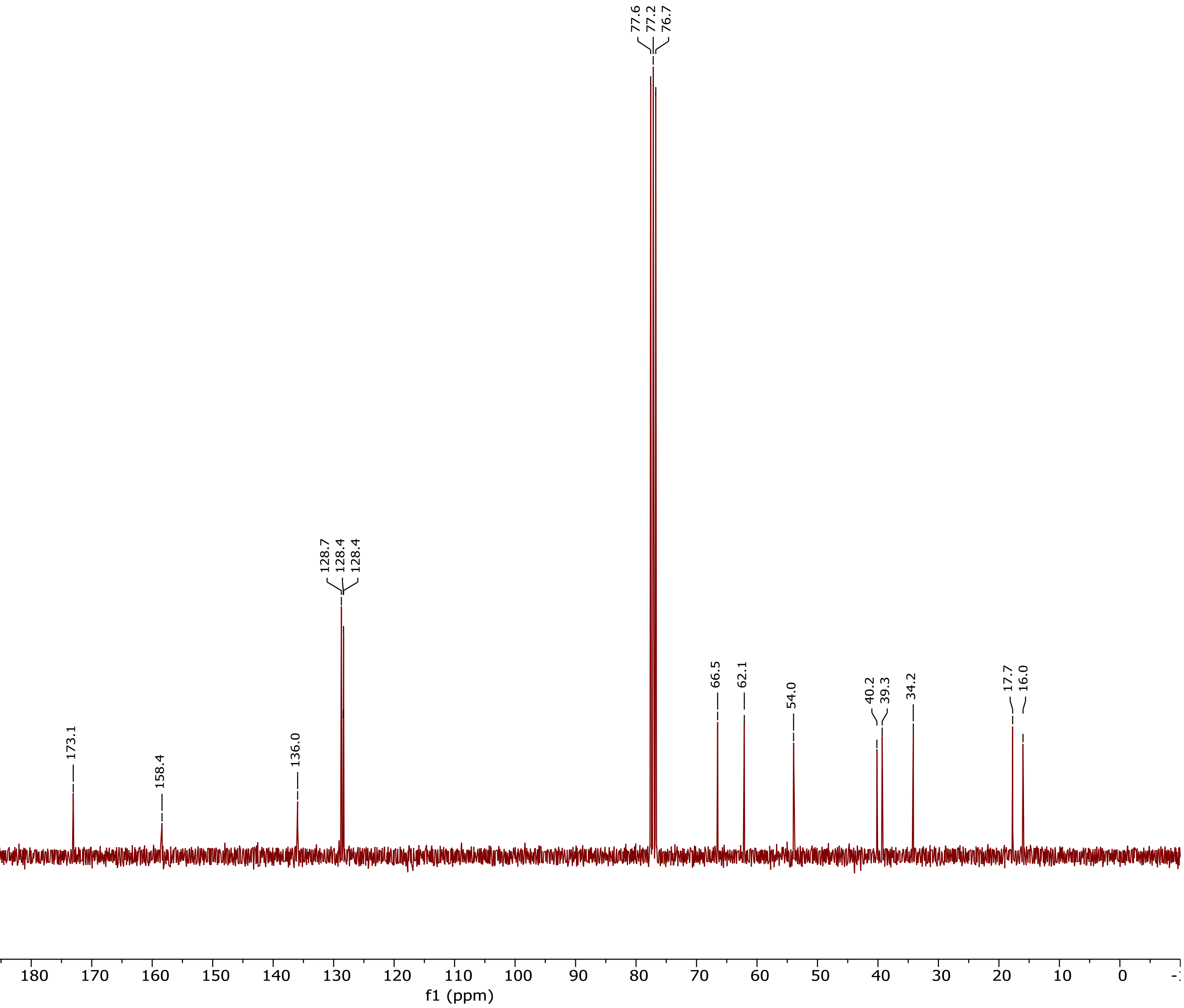

Benzyl 3-methyl-4-(2-oxooxazolidin-3-yl)pentanoate (**59**)
2nd diastereoisomer

^{1}H NMR (300 MHz, $CDCl_3$)

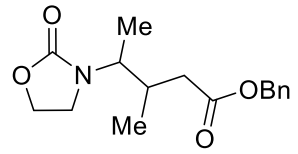

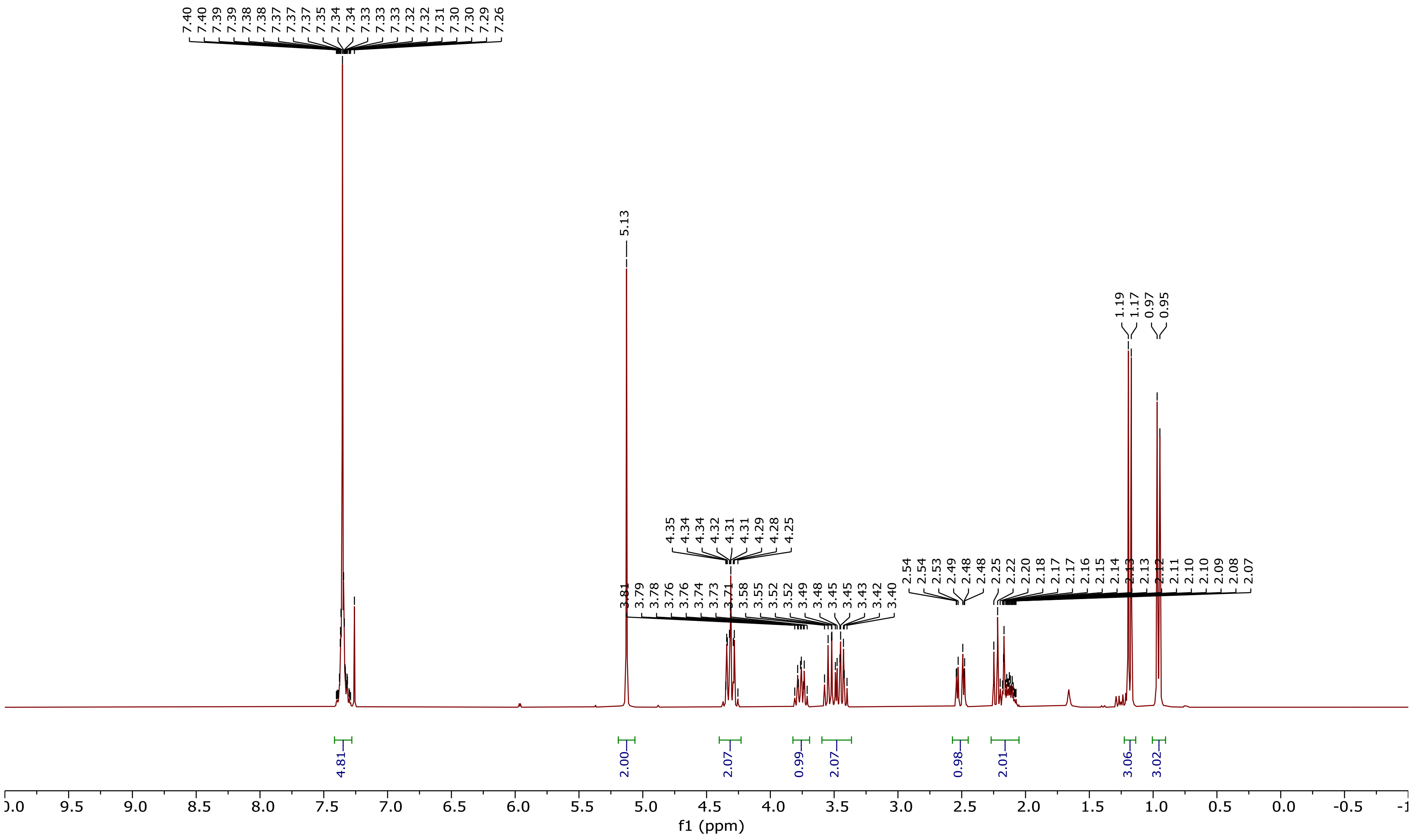

Benzyl 3-methyl-4-(2-oxooxazolidin-3-yl)pentanoate (**59**) ^{13}C NMR (75 MHz, $CDCl_3$)

2nd diastereoisomer

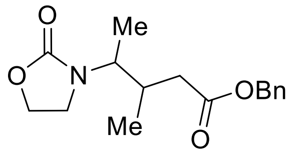

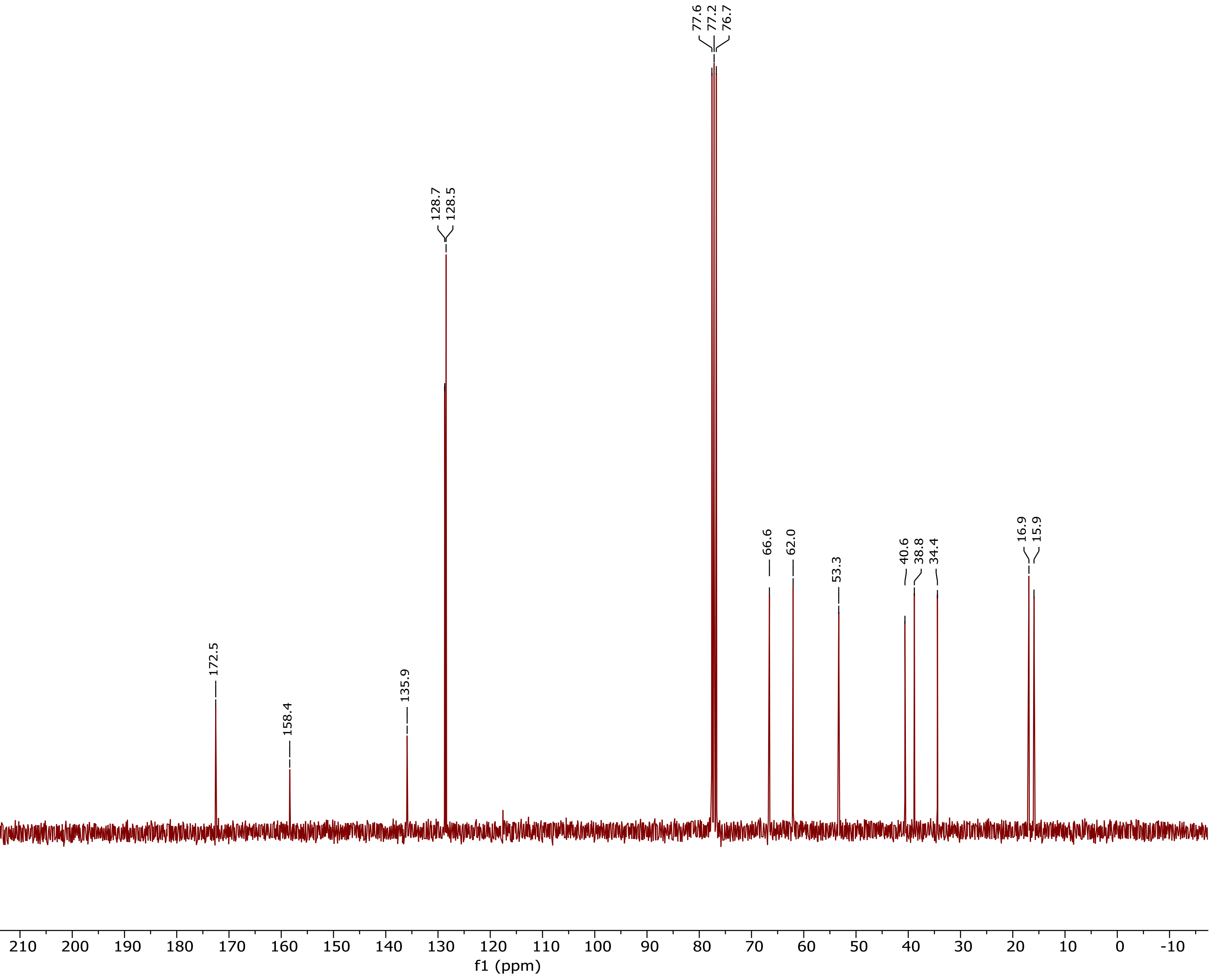

Benzyl 2-(2-(2-oxooxazolidin-3-yl)cyclohexyl)acetate (**60**)

1st diastereoisomer

^{1}H NMR (300 MHz, $CDCl_3$)

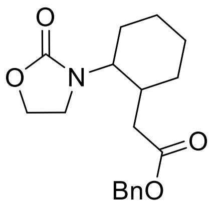

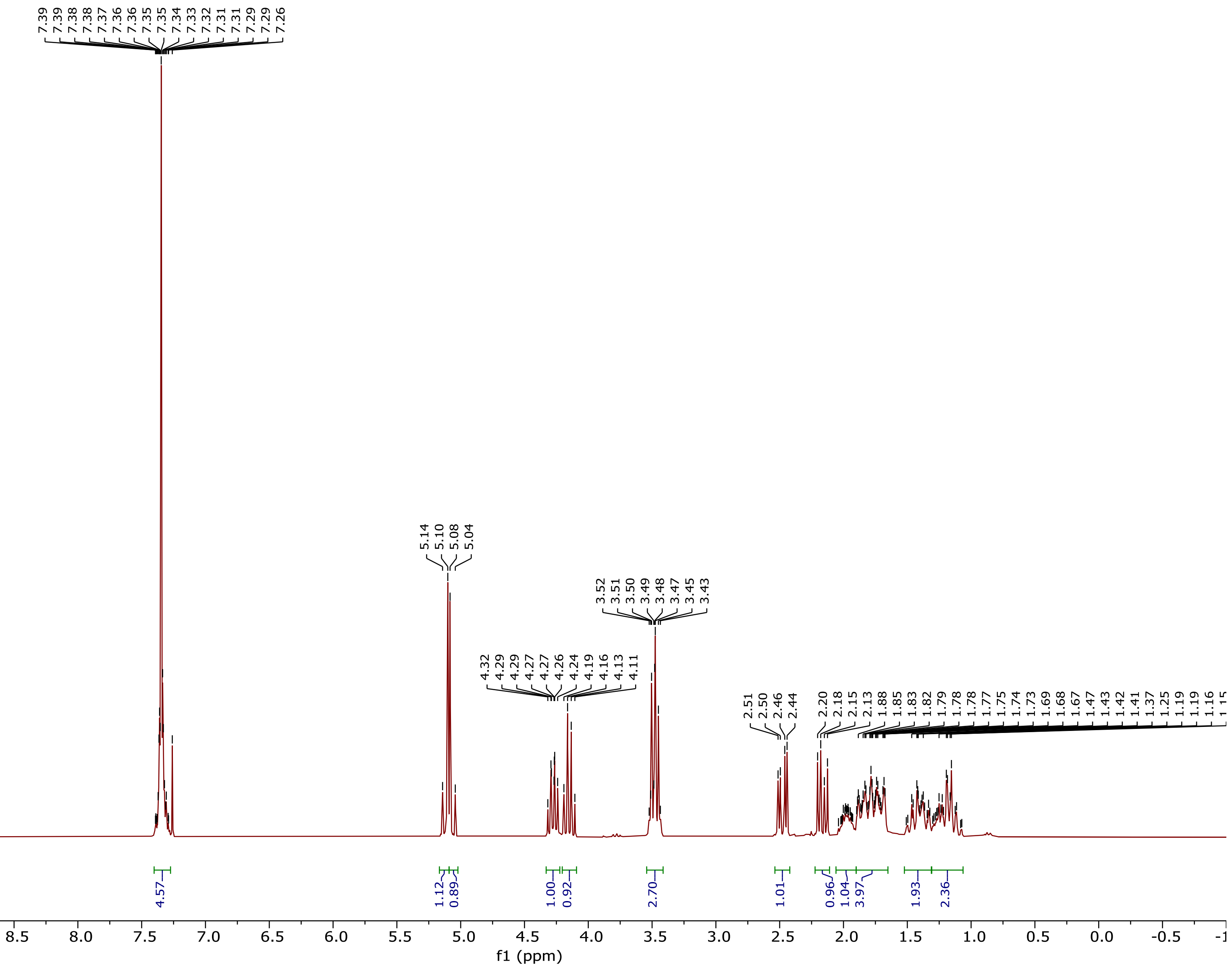

Benzyl 2-(2-(2-oxooxazolidin-3-yl)cyclohexyl)acetate (**60**)

^{13}C NMR (75 MHz, $CDCl_3$)

1st diastereoisomer

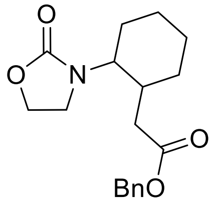

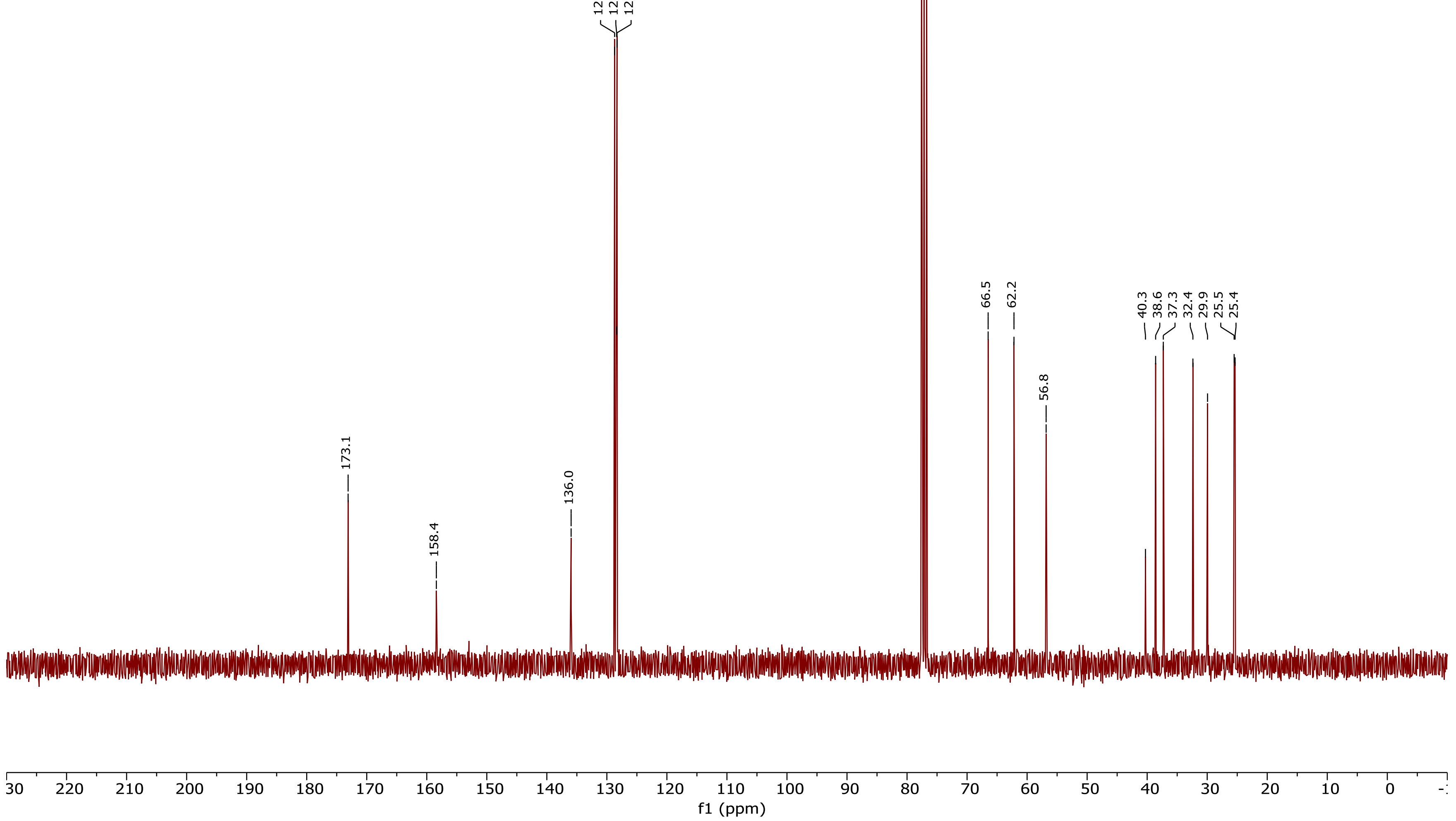

Benzyl 2-(2-(2-oxooxazolidin-3-yl)cyclohexyl)acetate (**60**)

2nd diastereoisomer

^{1}H NMR (300 MHz, $CDCl_3$)

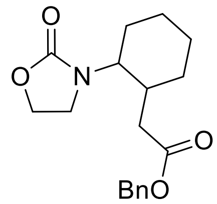

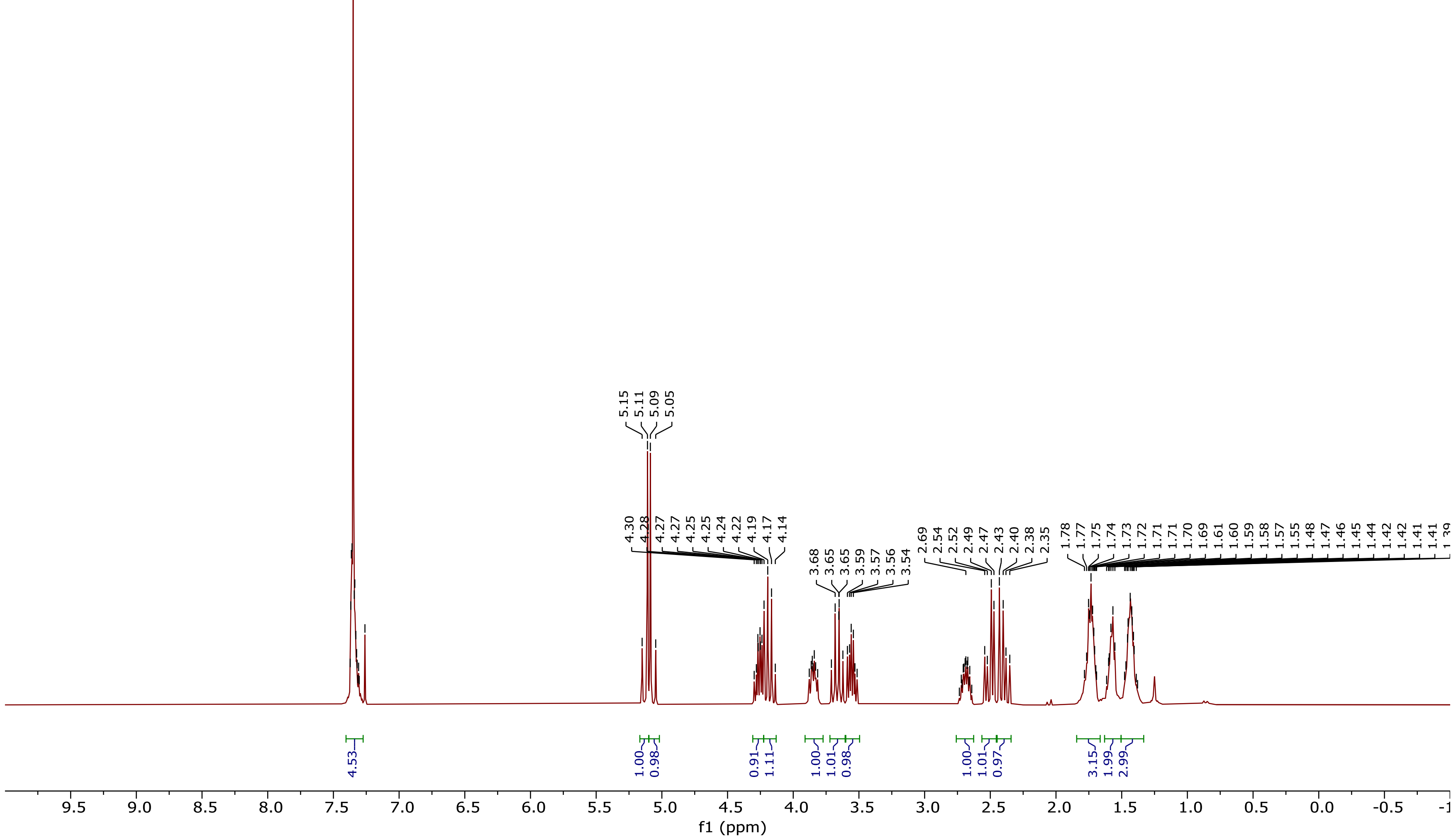

Benzyl 2-(2-(2-oxooxazolidin-3-yl)cyclohexyl)acetate (**60**)
2nd diastereoisomer

^{13}C NMR (75 MHz, $CDCl_3$)

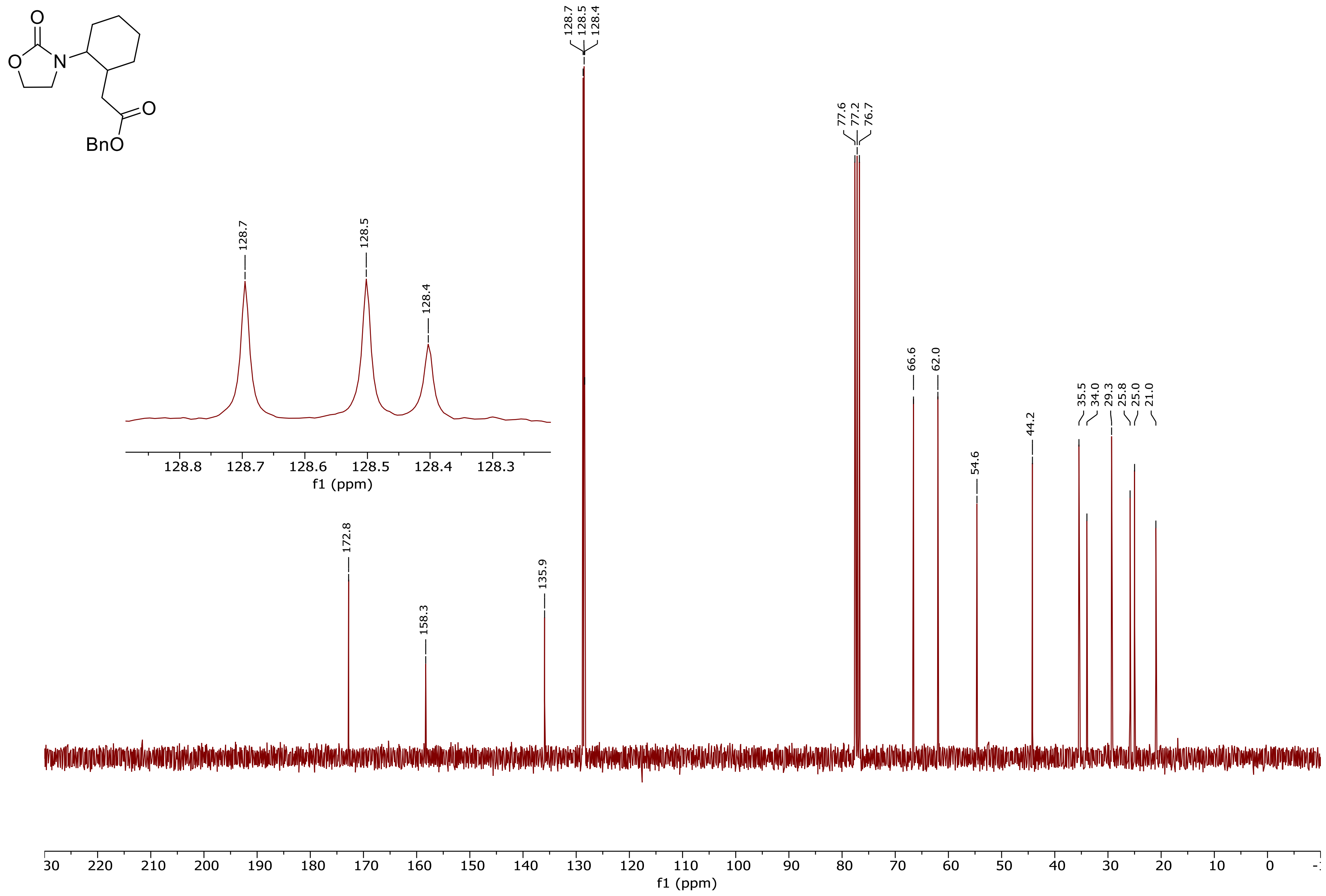

Ethyl 4-((*S*)-2-oxo-4-phenyloxazolidin-3-yl)pentanoate (**62**)
1st diastereoisomer

^{1}H NMR (300 MHz, $CDCl_3$)

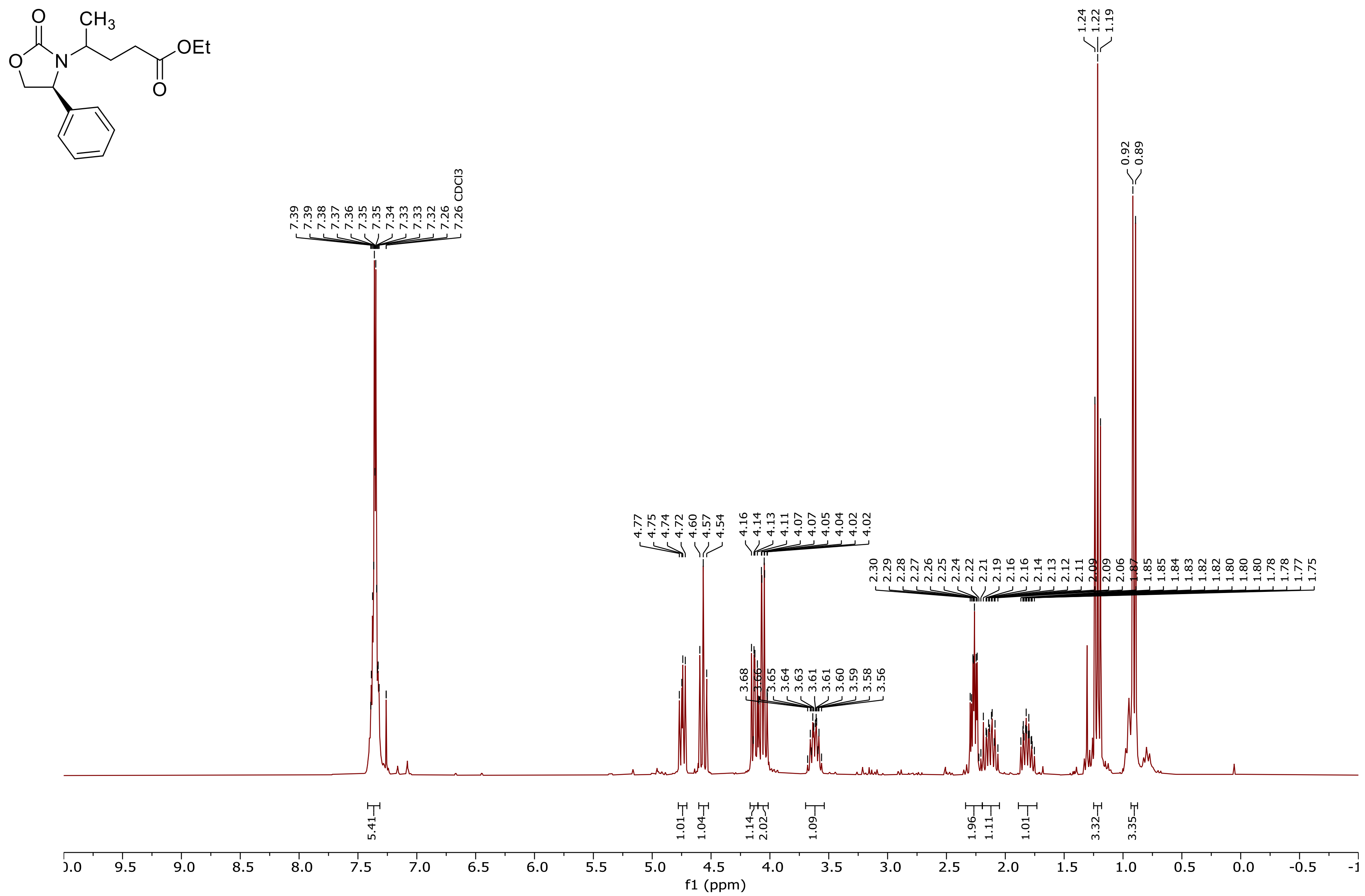

Ethyl 4-((*S*)-2-oxo-4-phenyloxazolidin-3-yl)pentanoate (**62**)
1st diastereoisomer

^{13}C NMR (75 MHz, $CDCl_3$)

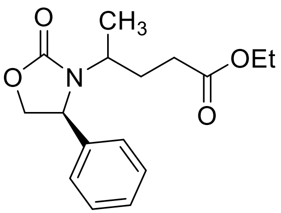

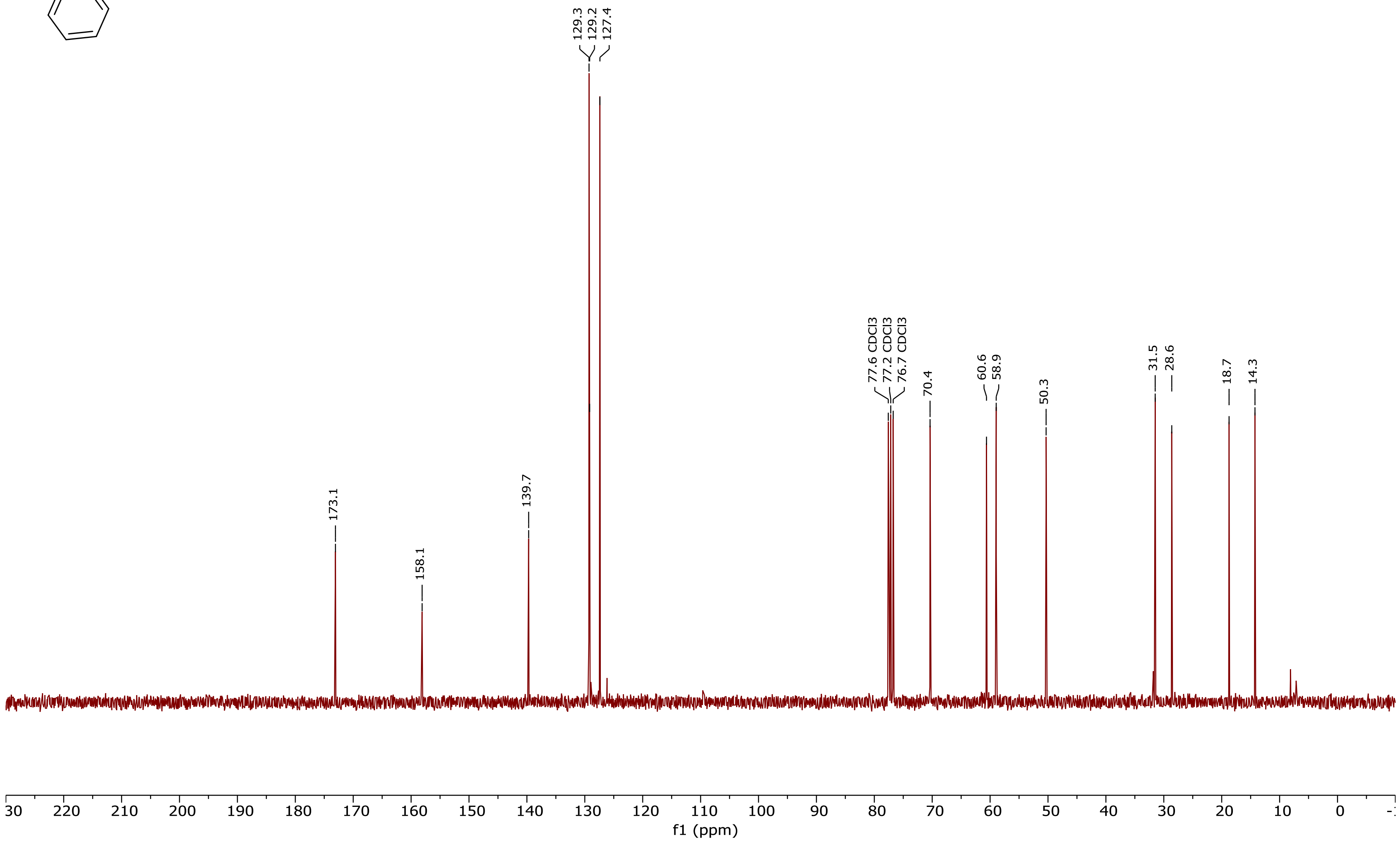

Ethyl 4-((*S*)-2-oxo-4-phenyloxazolidin-3-yl)pentanoate (**62**)
2nd diastereoisomer

^{1}H NMR (300 MHz, $CDCl_3$)

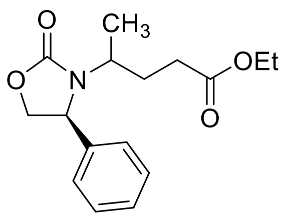

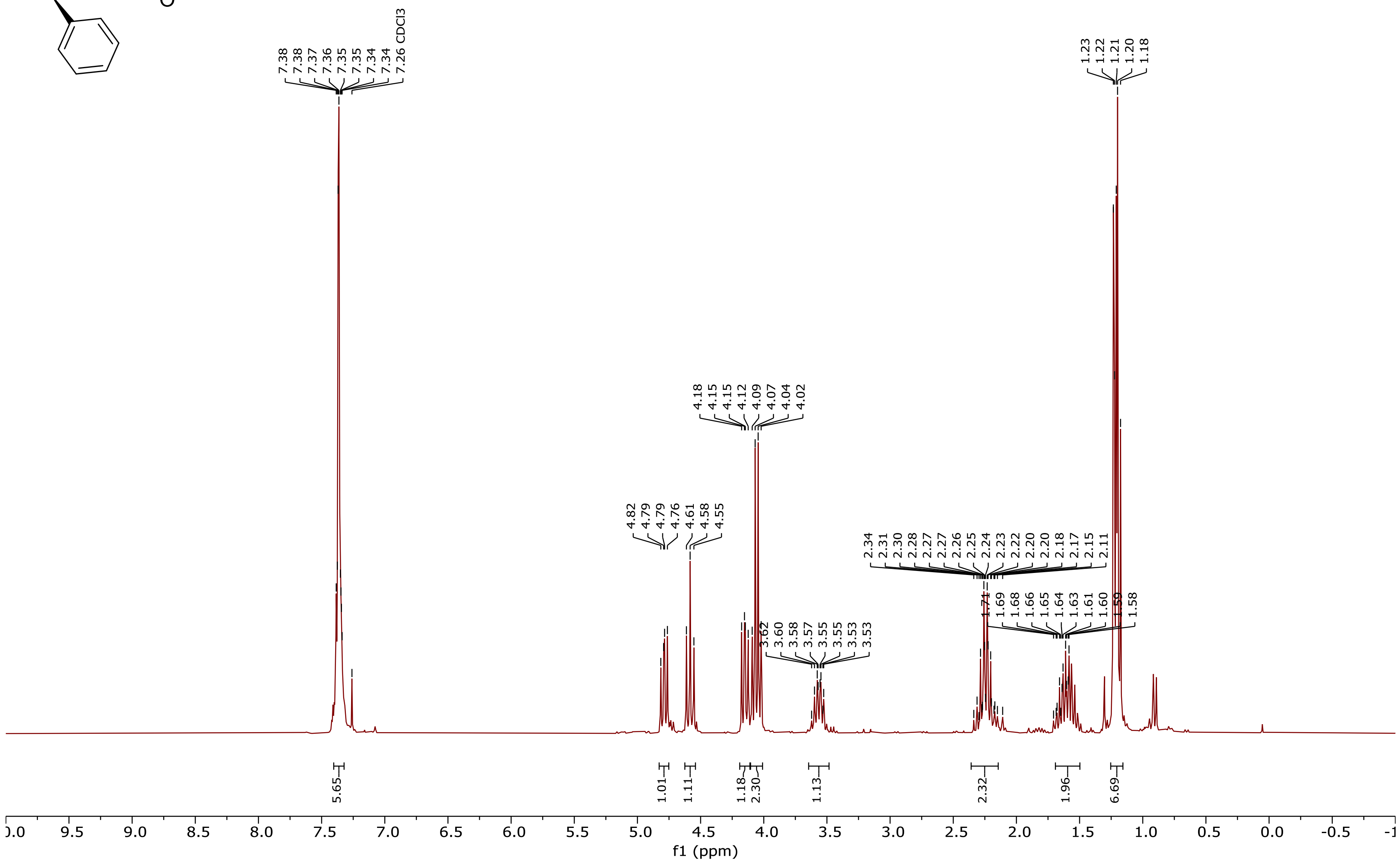

Ethyl 4-((*S*)-2-oxo-4-phenyloxazolidin-3-yl)pentanoate (**62**)
2nd diastereoisomer

^{13}C NMR (75 MHz, $CDCl_3$)

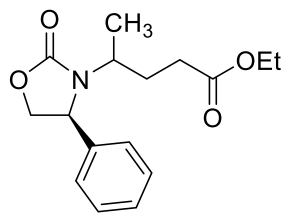

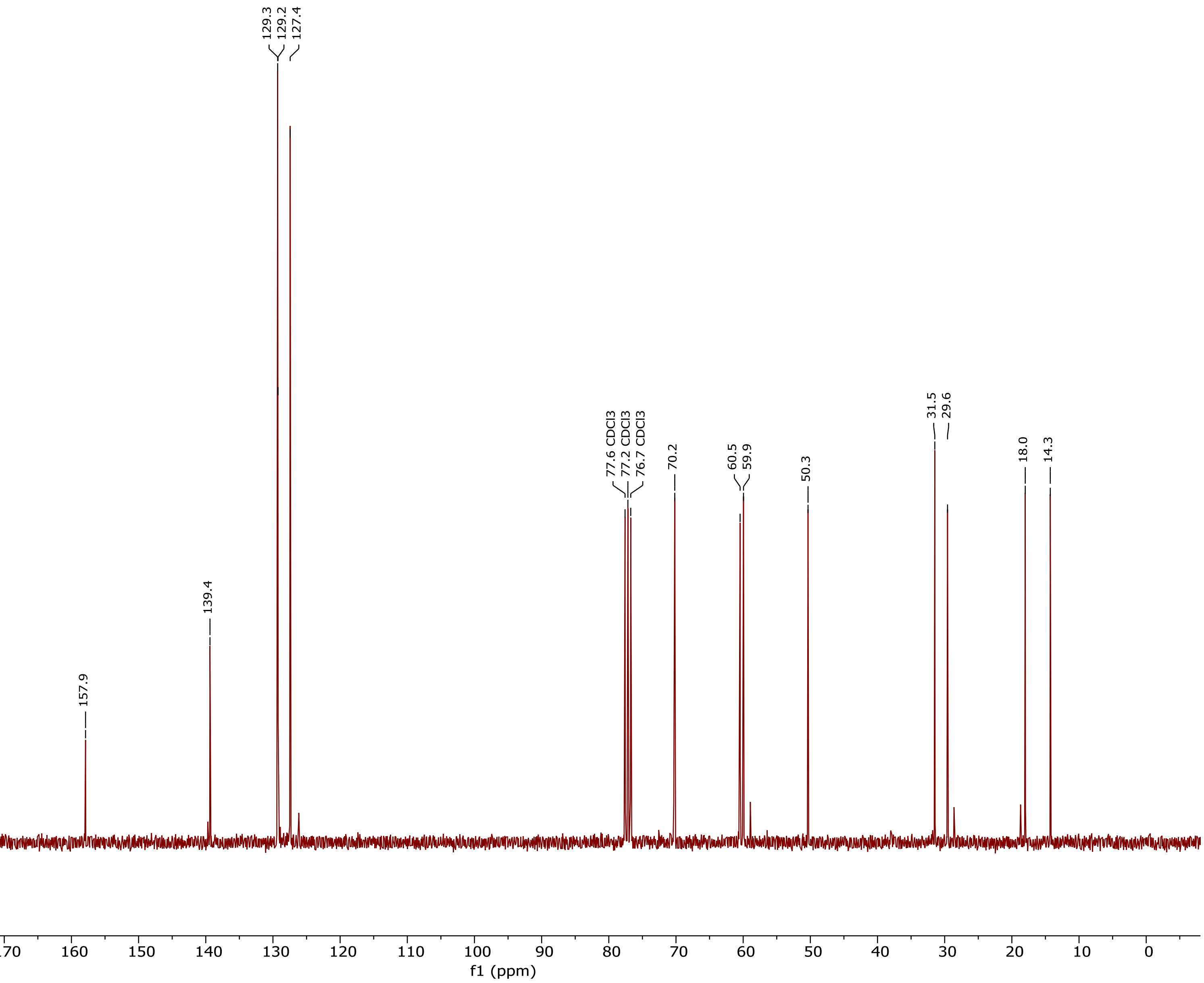

Ethyl 4-((*S*)-4-benzyl-2-oxooxazolidin-3-yl)pentanoate (**63**)

^{1}H NMR (300 MHz, $CDCl_3$)

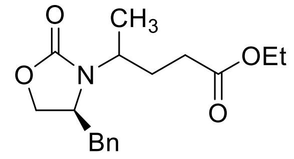

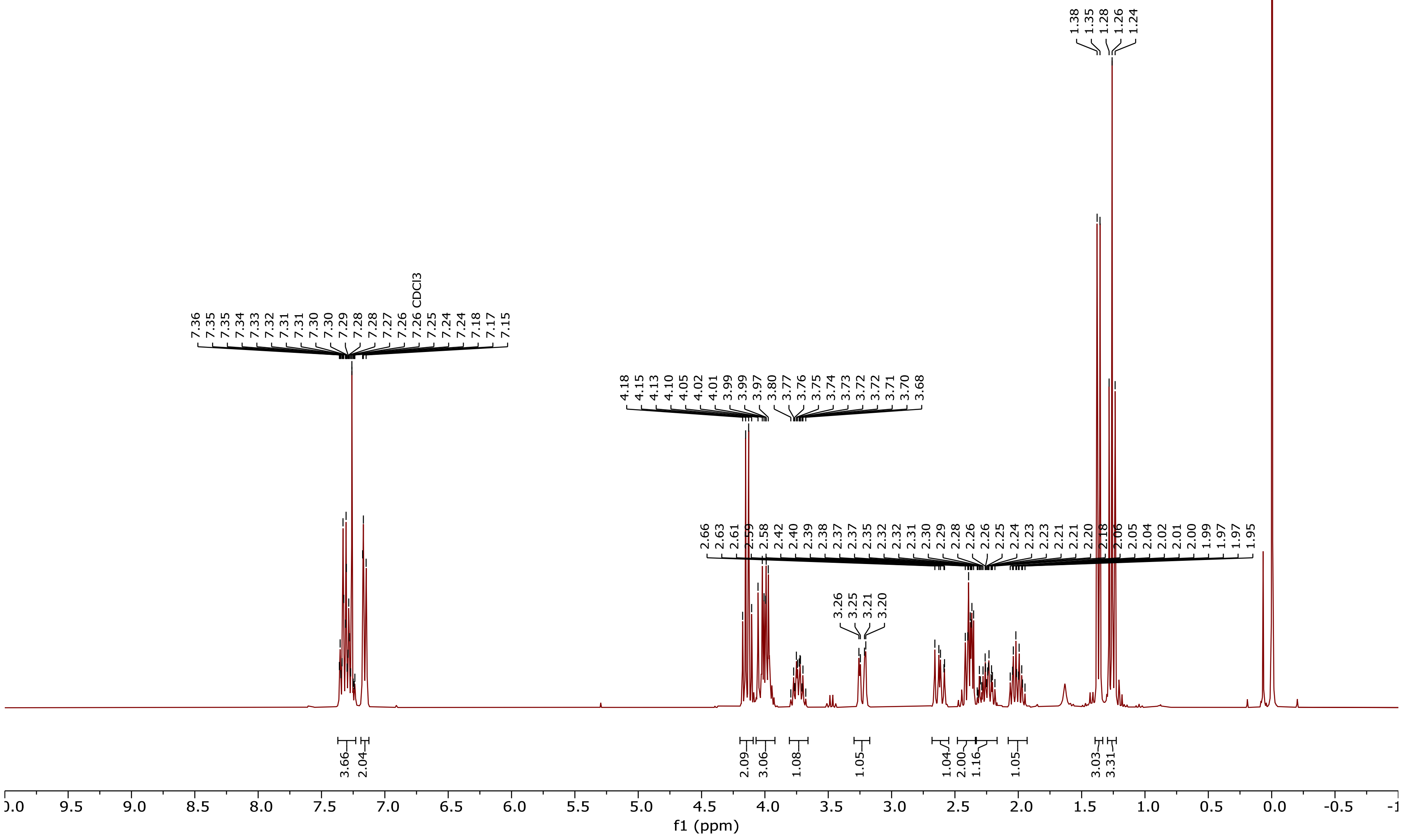

Ethyl 4-((*S*)-4-benzyl-2-oxooxazolidin-3-yl)pentanoate (**63**)

^{13}C NMR (75 MHz, $CDCl_3$)

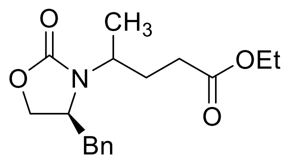

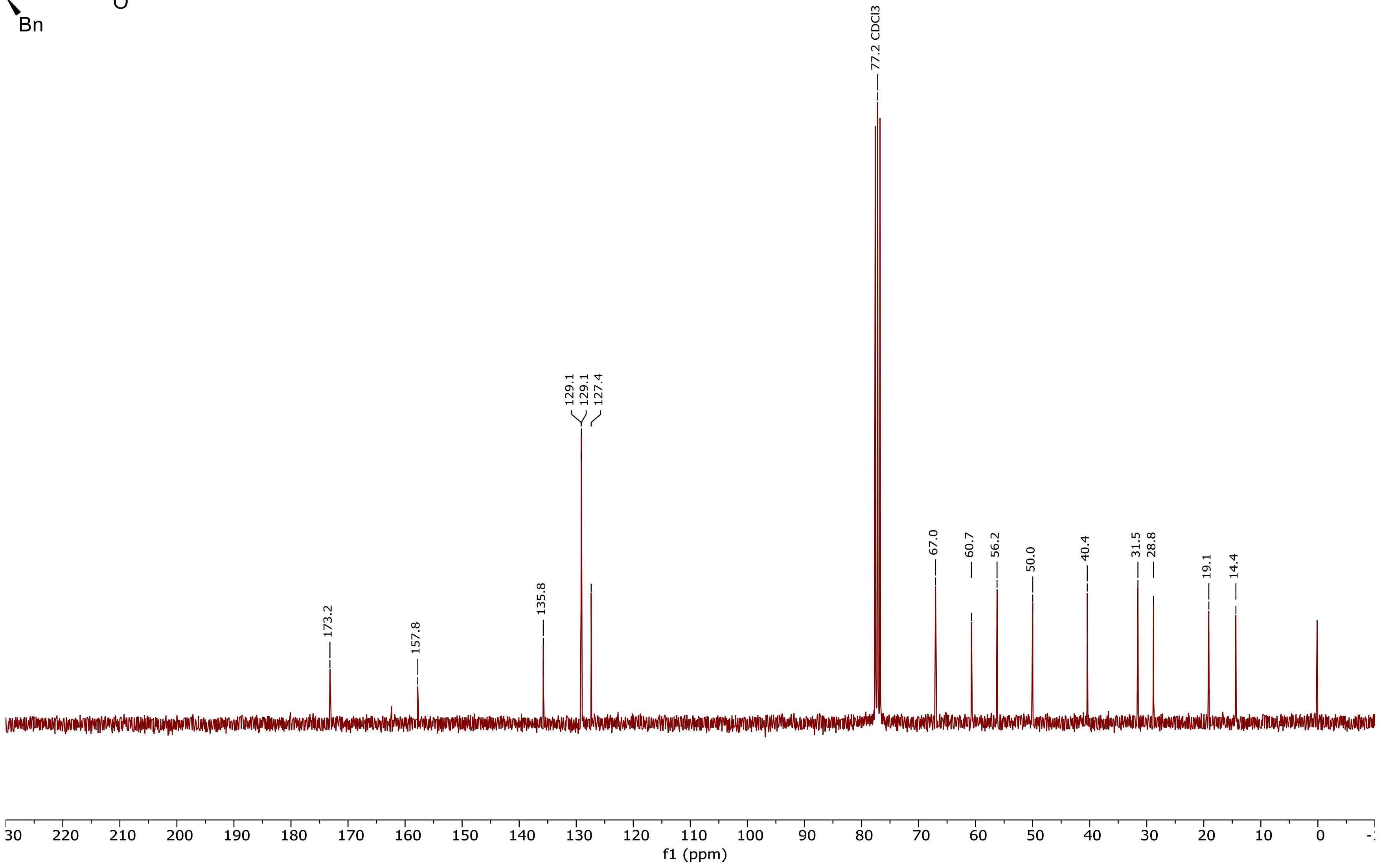

Ethyl 4-((*S*)-4-benzyl-2-oxooxazolidin-3-yl)pentanoate (**63**-*diastereomeric mixture*)

^{1}H NMR (300 MHz, $CDCl_3$)

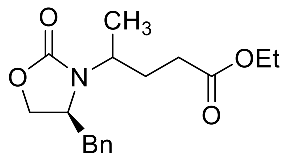

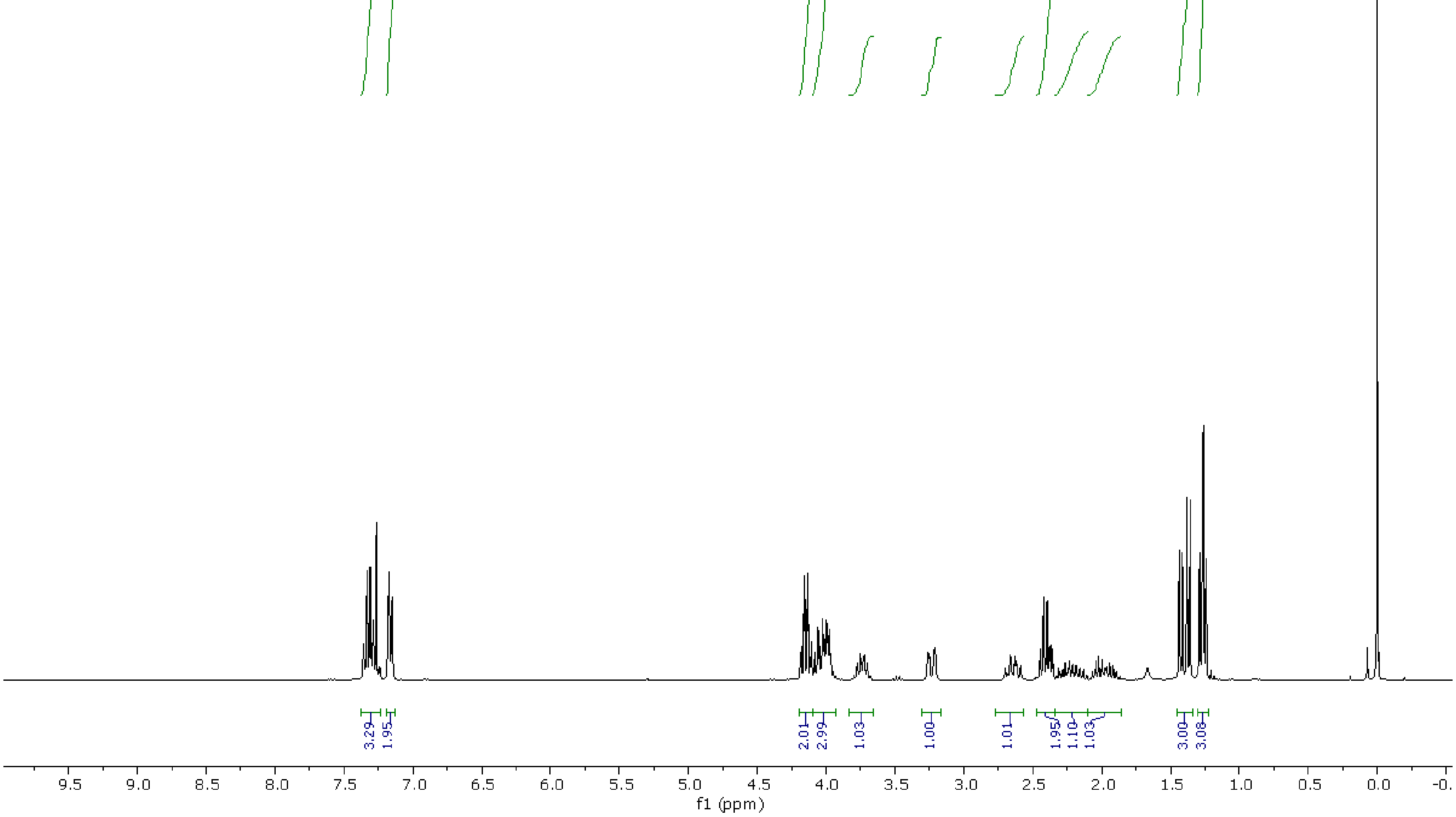

Ethyl 4-((*S*)-4-benzyl-2-oxooxazolidin-3-yl)pentanoate (**63-***diastereomeric mixture*)

^{13}C NMR (75 MHz, $CDCl_3$)

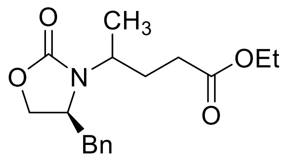

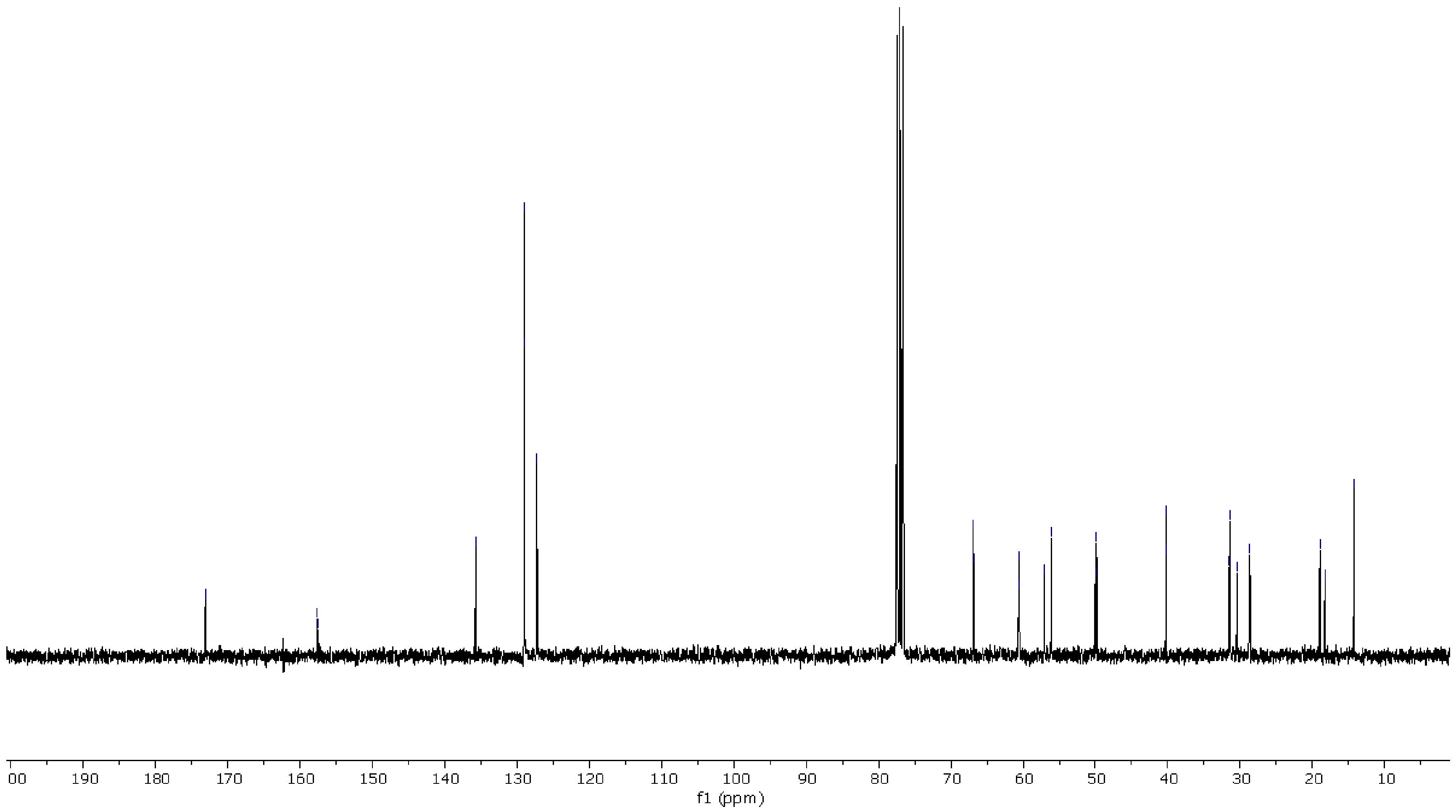

Benzyl 4-((*S*)-4-isopropyl-2-oxooxazolidin-3-yl)pentanoate (**64**) ^{1}H NMR (300 MHz, $CDCl_3$)

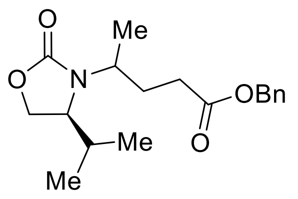

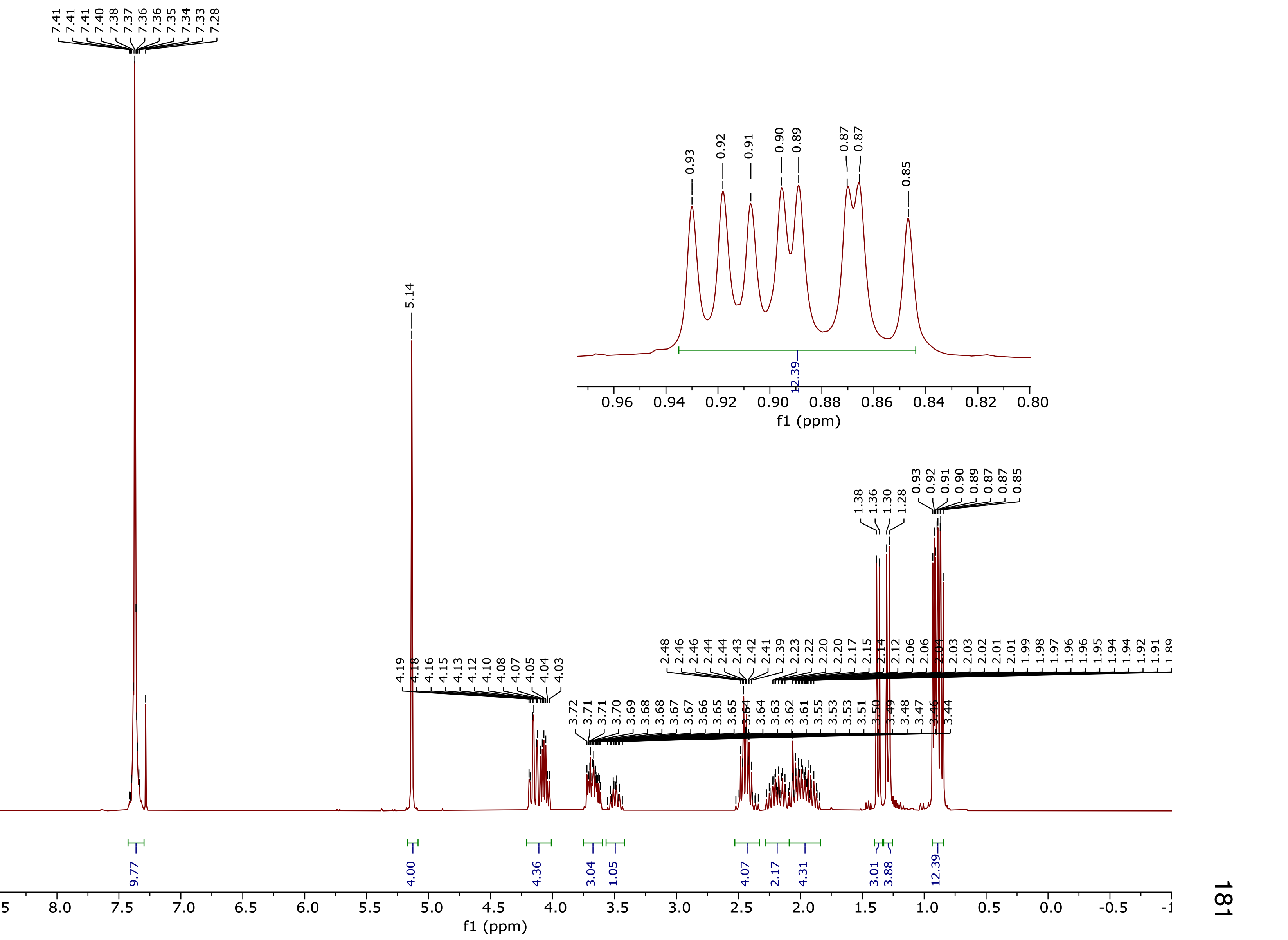

Benzyl 4-((*S*)-4-isopropyl-2-oxooxazolidin-3-yl)pentanoate (**64**)

^{13}C NMR (75 MHz, $CDCl_3$)

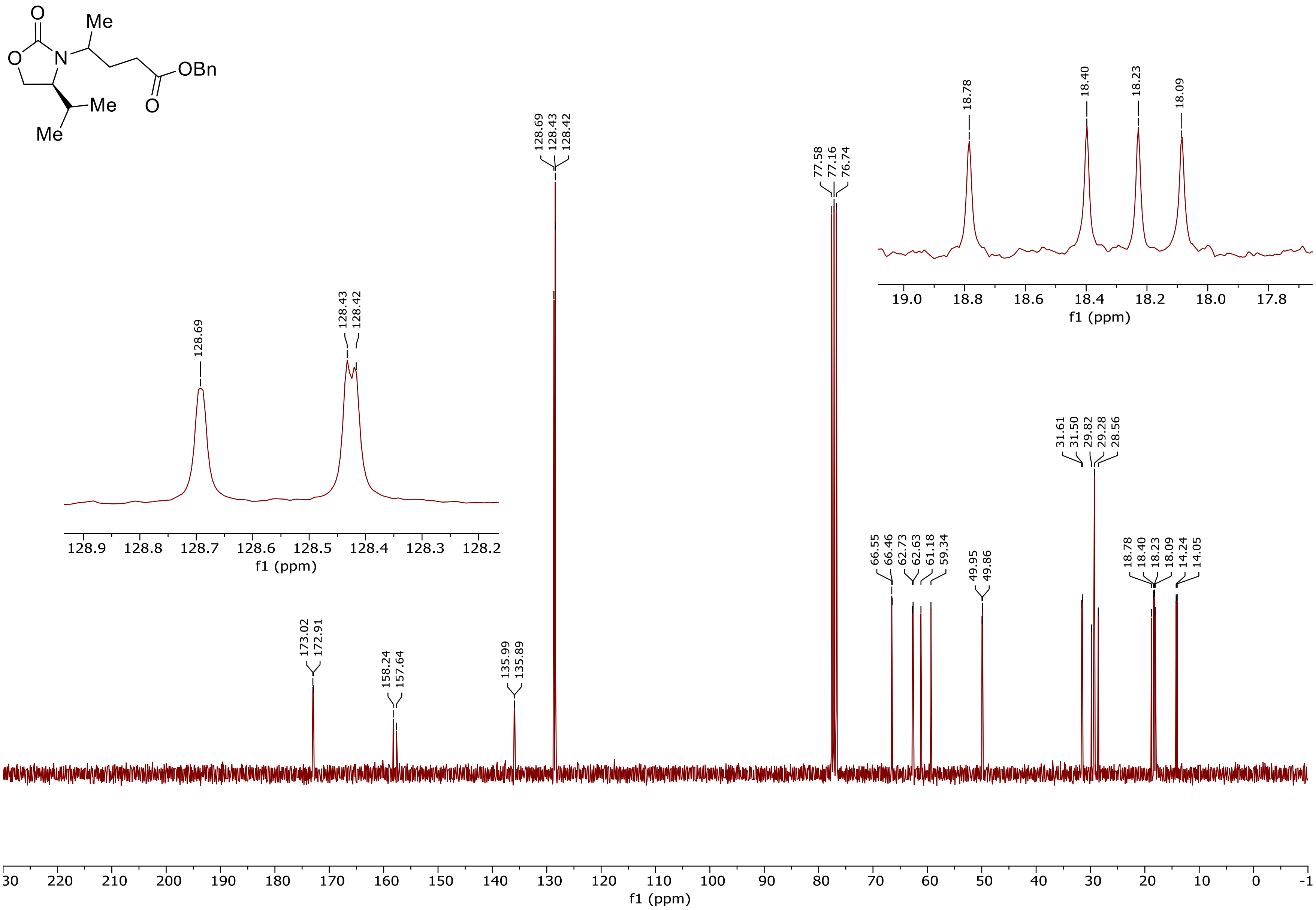

Benzyl 3-(((S)-4-isopropyl-2-oxooxazolidin-3-yl)methyl)pentanoate (**65**) ^{1}H NMR (300 MHz, $CDCl_3$)

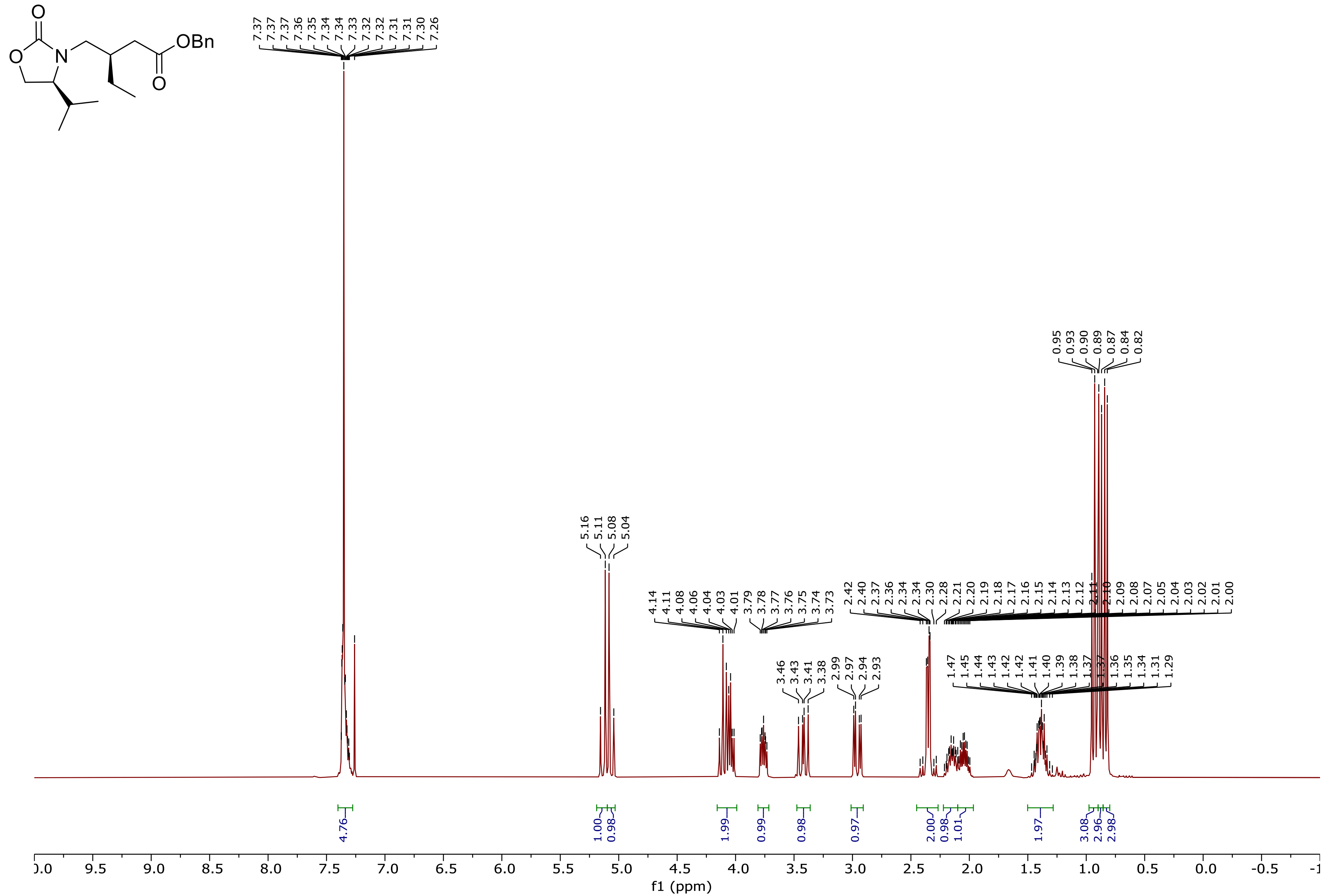

Benzyl 3-(((S)-4-isopropyl-2-oxooxazolidin-3-yl)methyl)pentanoate (**65**) ^{13}C NMR (75 MHz, $CDCl_3$)

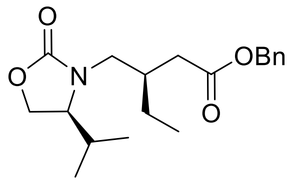

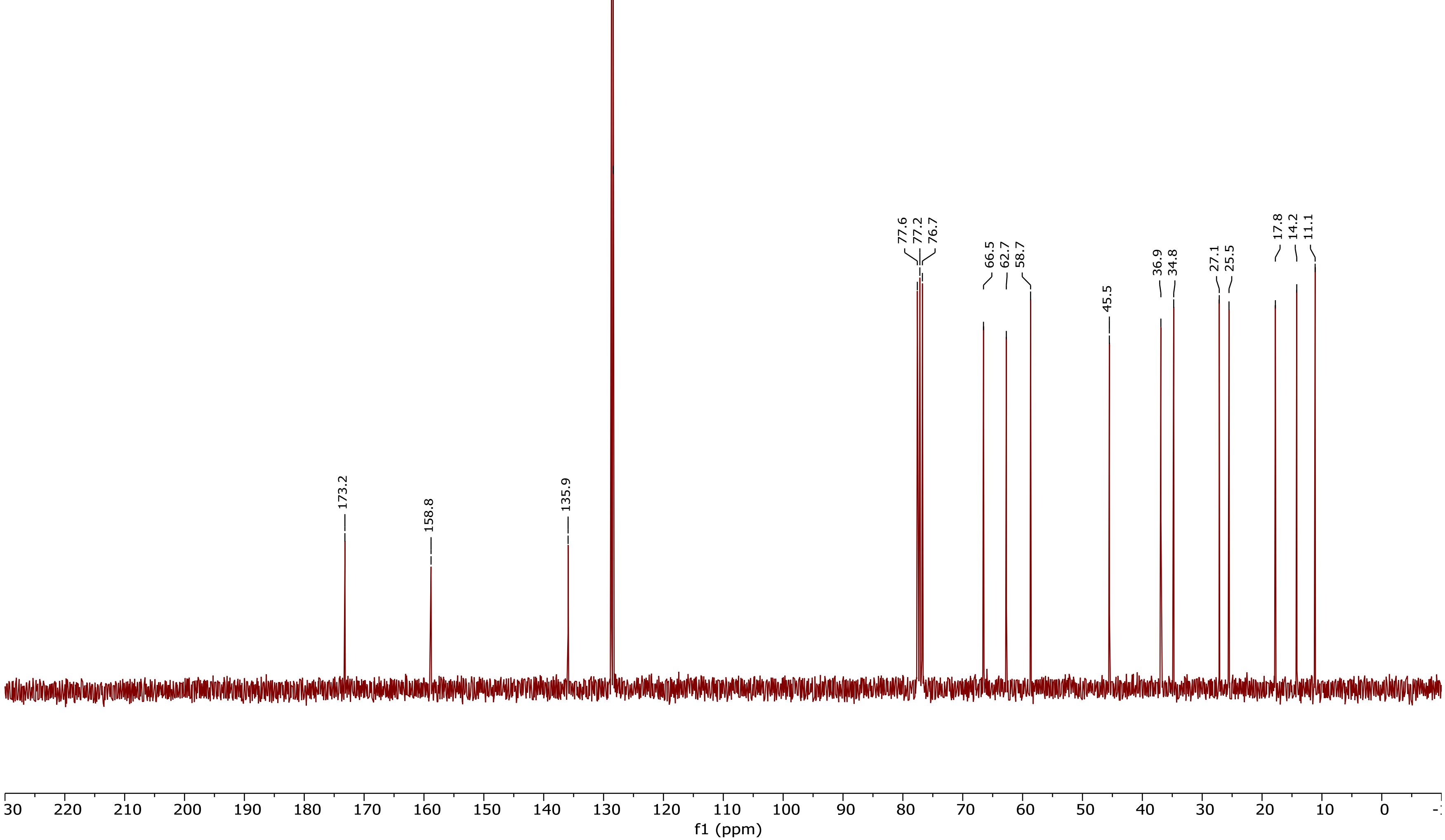

Benzyl 3-(((*S*)-2-oxo-4-phenyloxazolidin-3-yl)methyl)pentanoate (**66**)
Major diastereoisomer

^{1}H NMR (300 MHz, $CDCl_3$)

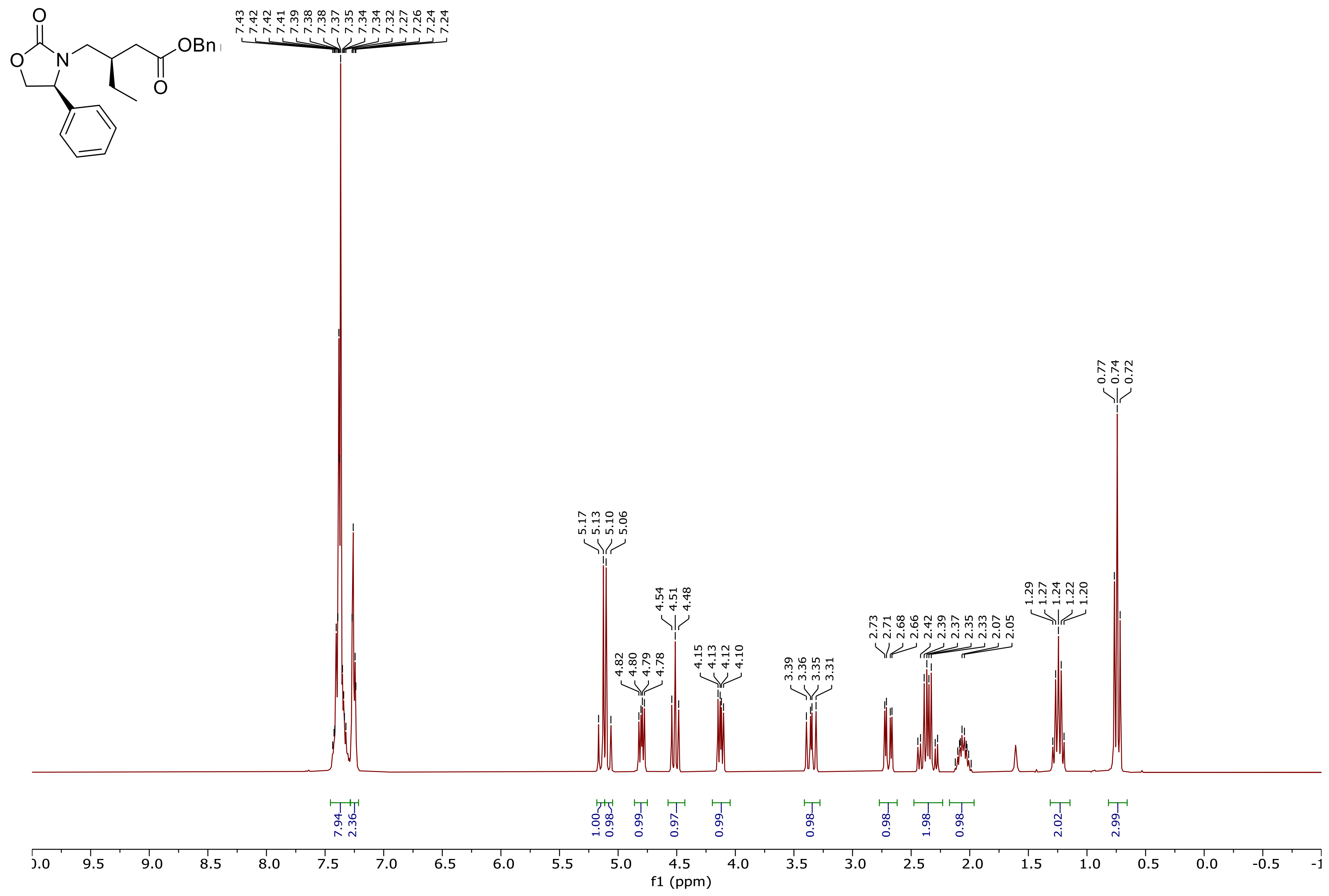

Benzyl 3-(((*S*)-2-oxo-4-phenyloxazolidin-3-yl)methyl)pentanoate (**66**)
Major diastereoisomer

^{13}C NMR (75 MHz, $CDCl_3$)

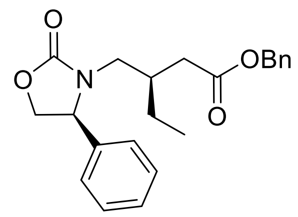

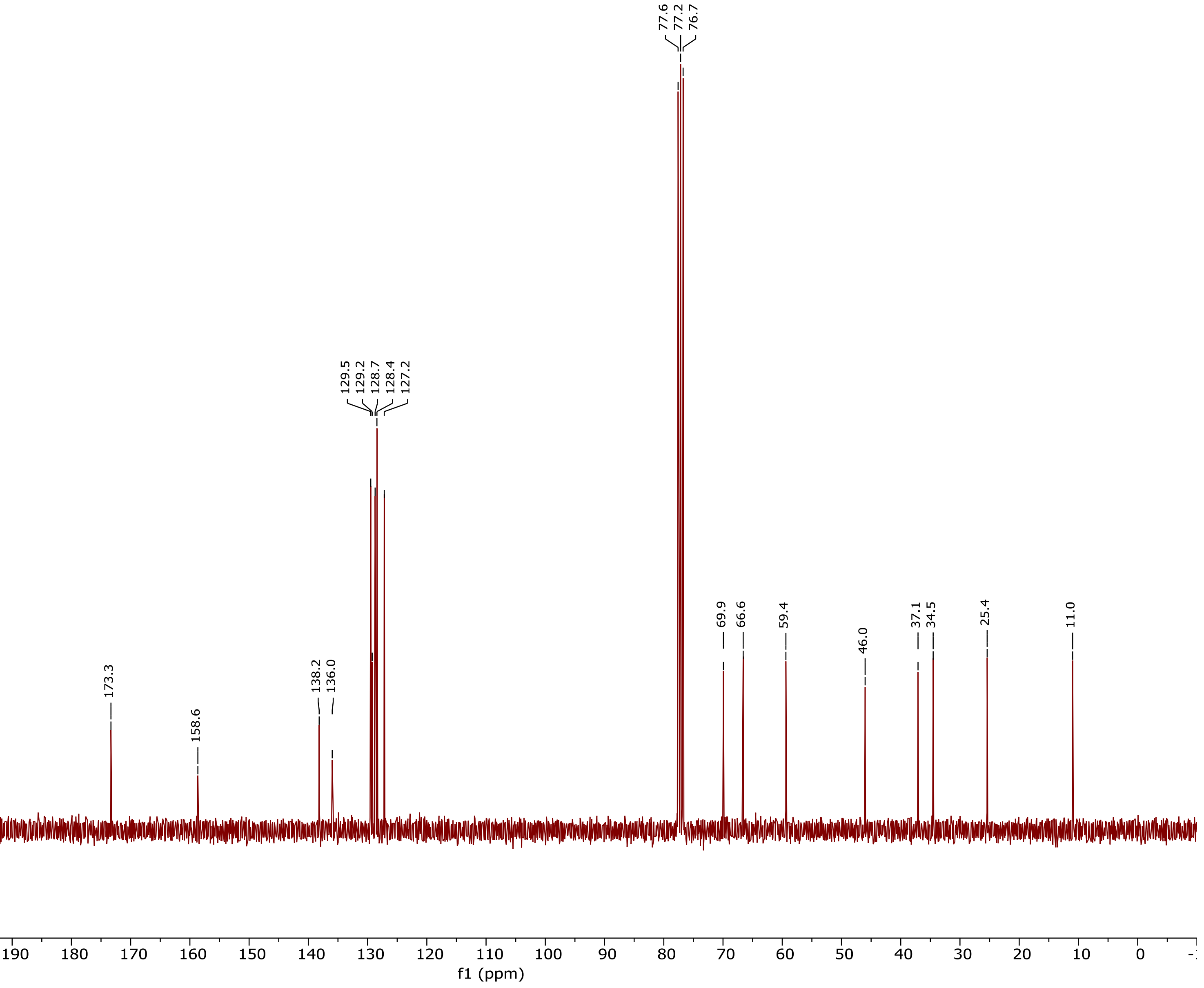

Benzyl 3-(((*S*)-2-oxo-4-phenyloxazolidin-3-yl)methyl)pentanoate (**66**)

^{1}H NMR (300 MHz, $CDCl_3$)

Minor diastereoisomer

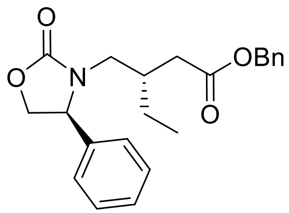

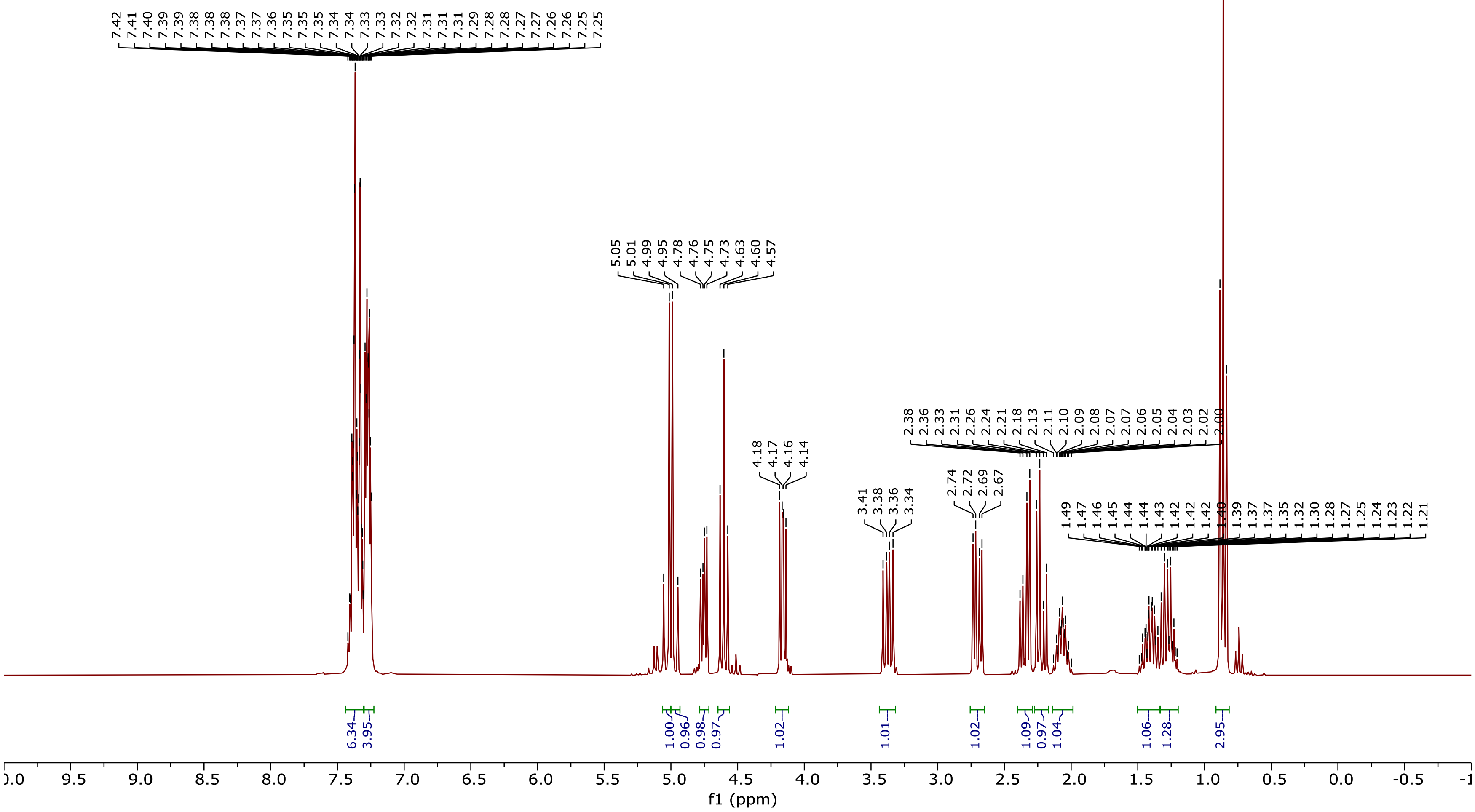

Benzyl 3-(((*S*)-2-oxo-4-phenyloxazolidin-3-yl)methyl)pentanoate (**66**) ^{13}C NMR (75 MHz, $CDCl_3$)

Minor diastereoisomer

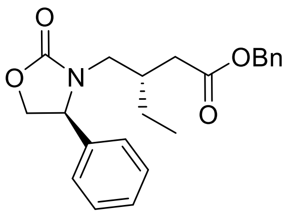

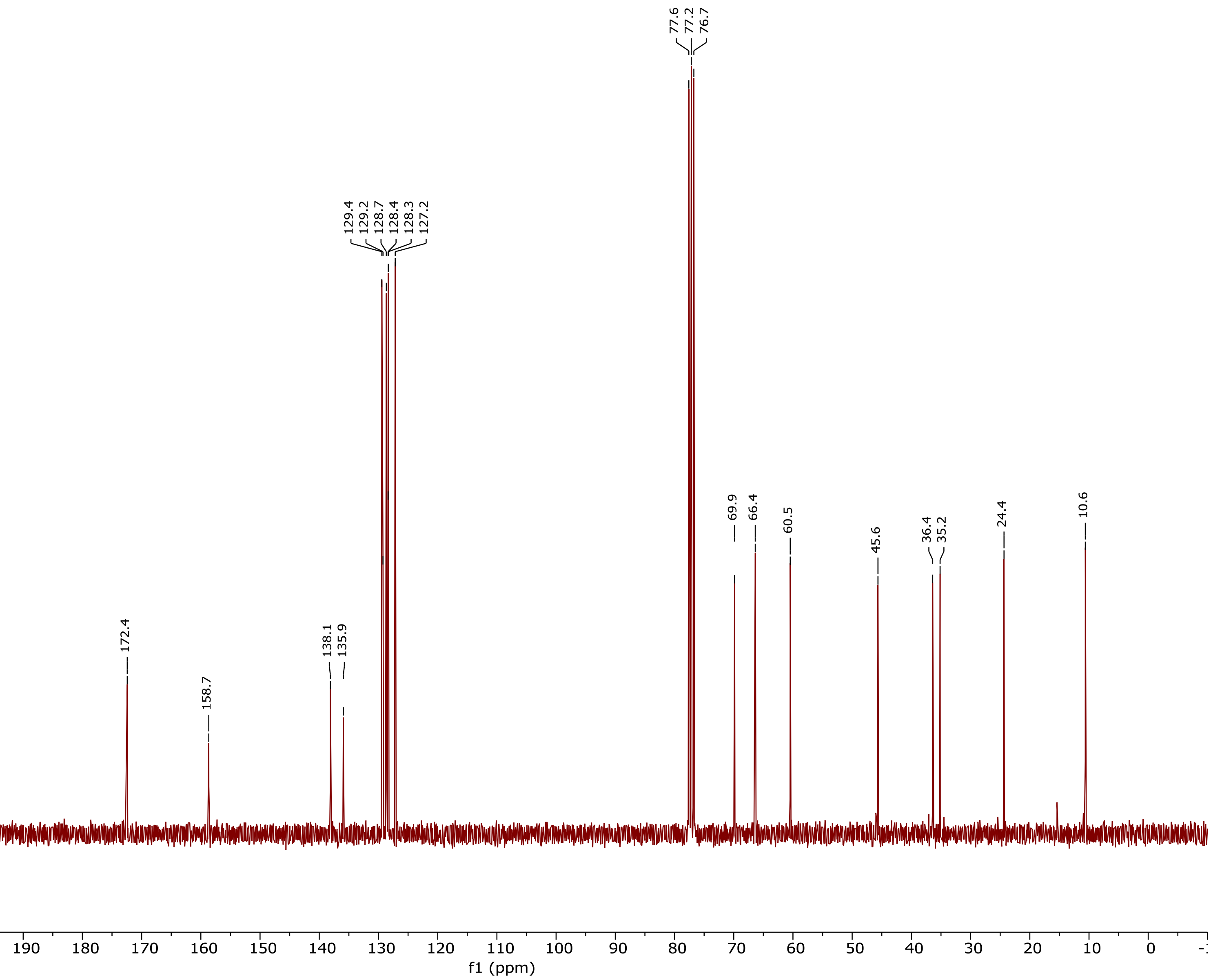

Diethyl 2-(1-((S)-4-isopropyl-2-oxooxazolidin-3-yl)butan-2-yl)malonate (**67**) ^{1}H NMR (300 MHz, $CDCl_3$)

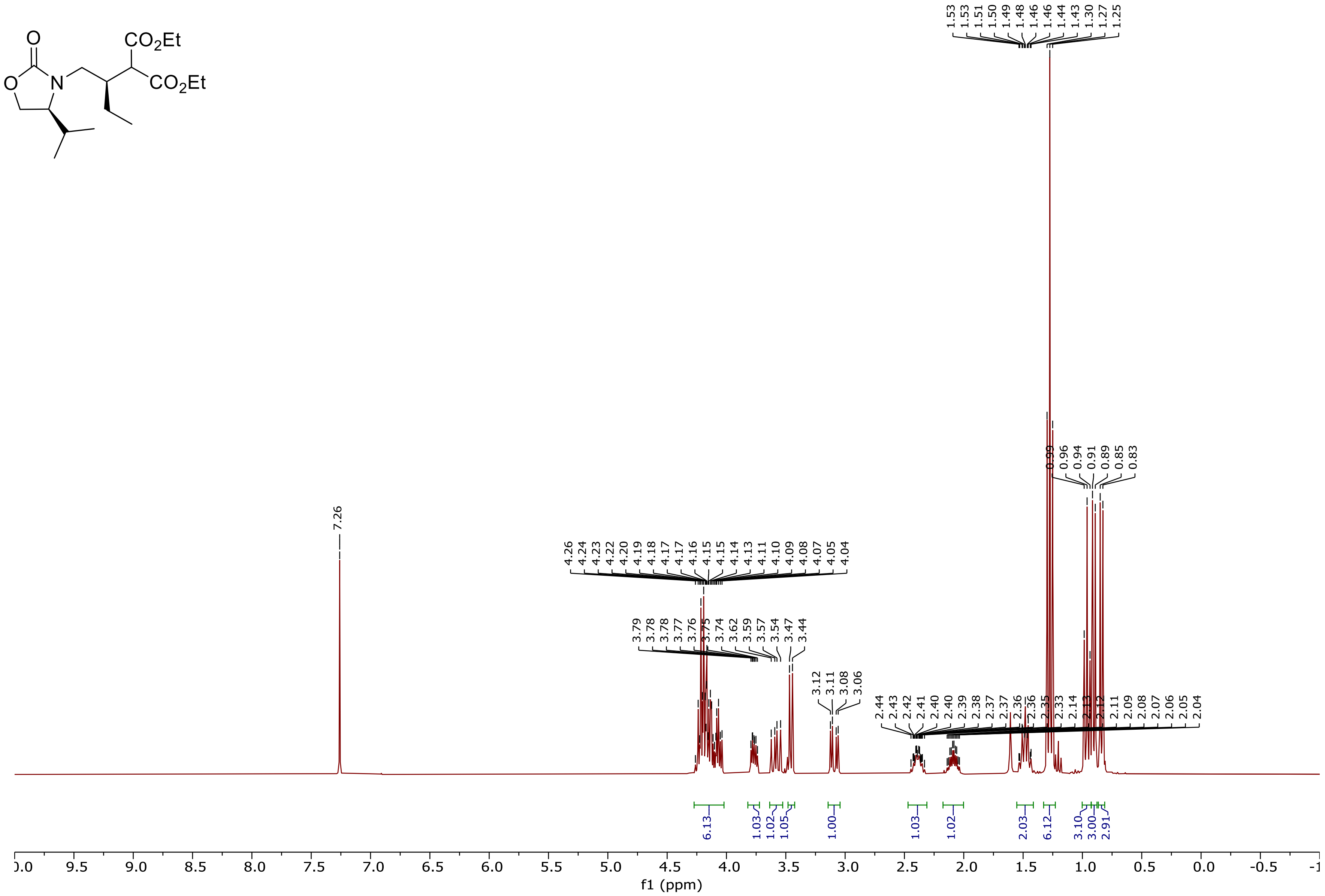

Diethyl 2-(1-((S)-4-isopropyl-2-oxooxazolidin-3-yl)butan-2-yl)malonate (**67**)

^{13}C NMR (75 MHz, $CDCl_3$)

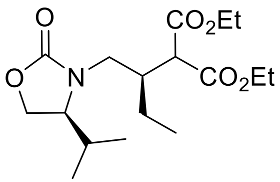

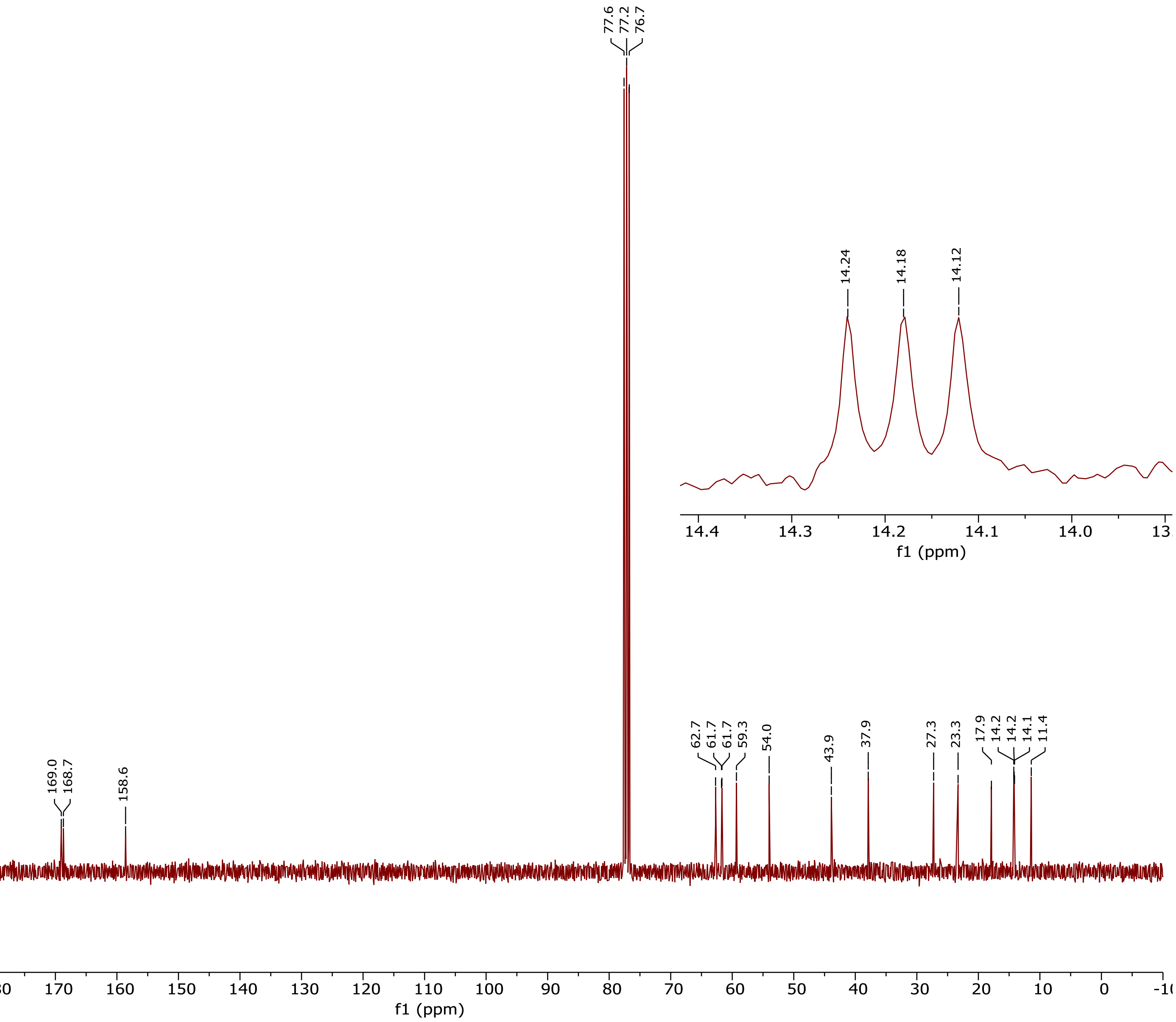

Diethyl 2-(1-((*S*)-2-oxo-4-phenyloxazolidin-3-yl)butan-2-yl)malonate (**68**) ^{1}H NMR (300 MHz, $CDCl_3$)

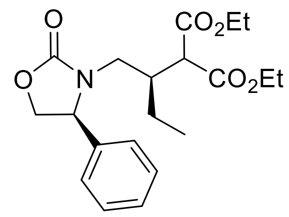

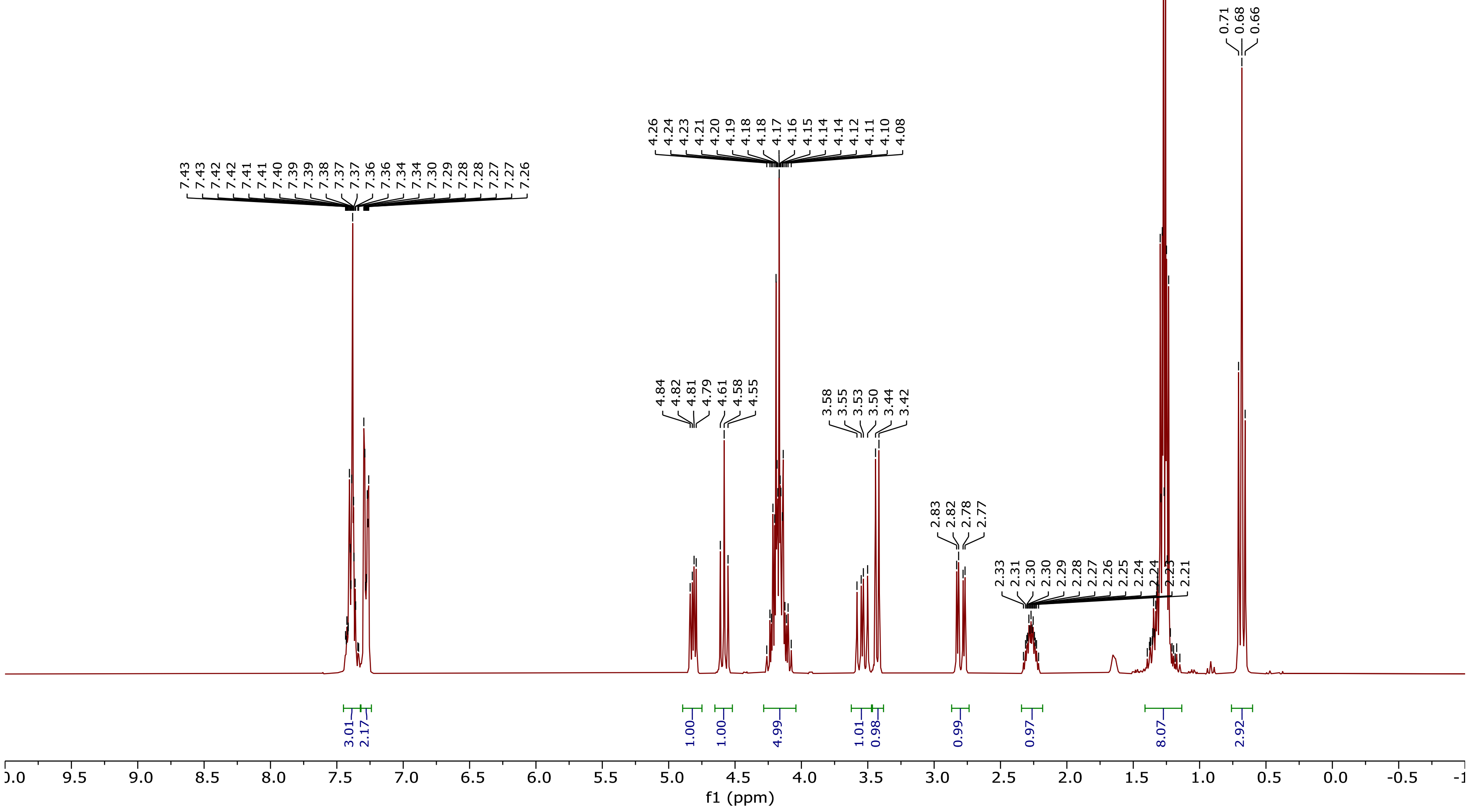

Diethyl 2-(1-((*S*)-2-oxo-4-phenyloxazolidin-3-yl)butan-2-yl)malonate (**68**) ^{13}C NMR (75 MHz, $CDCl_3$)

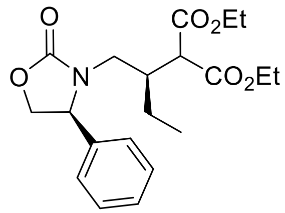

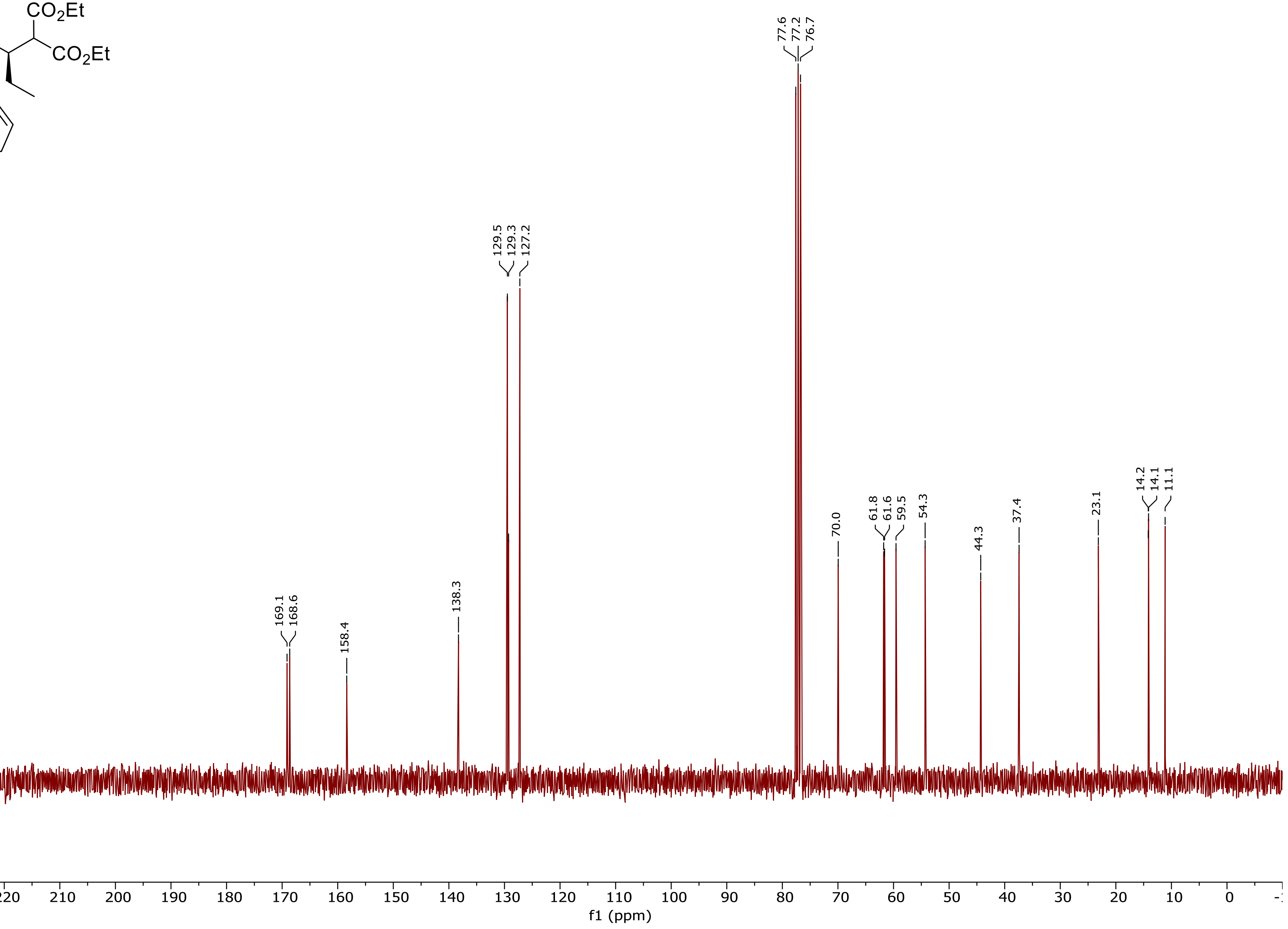

Benzyl 3-(((*S*)-4-isopropyl-2-oxooxazolidin-3-yl)methyl)-4-methyl-pentanoate (**69**)

^1H NMR (300 MHz, $CDCl_3$)

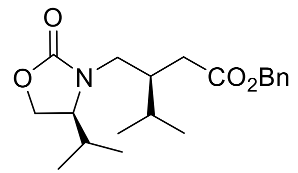

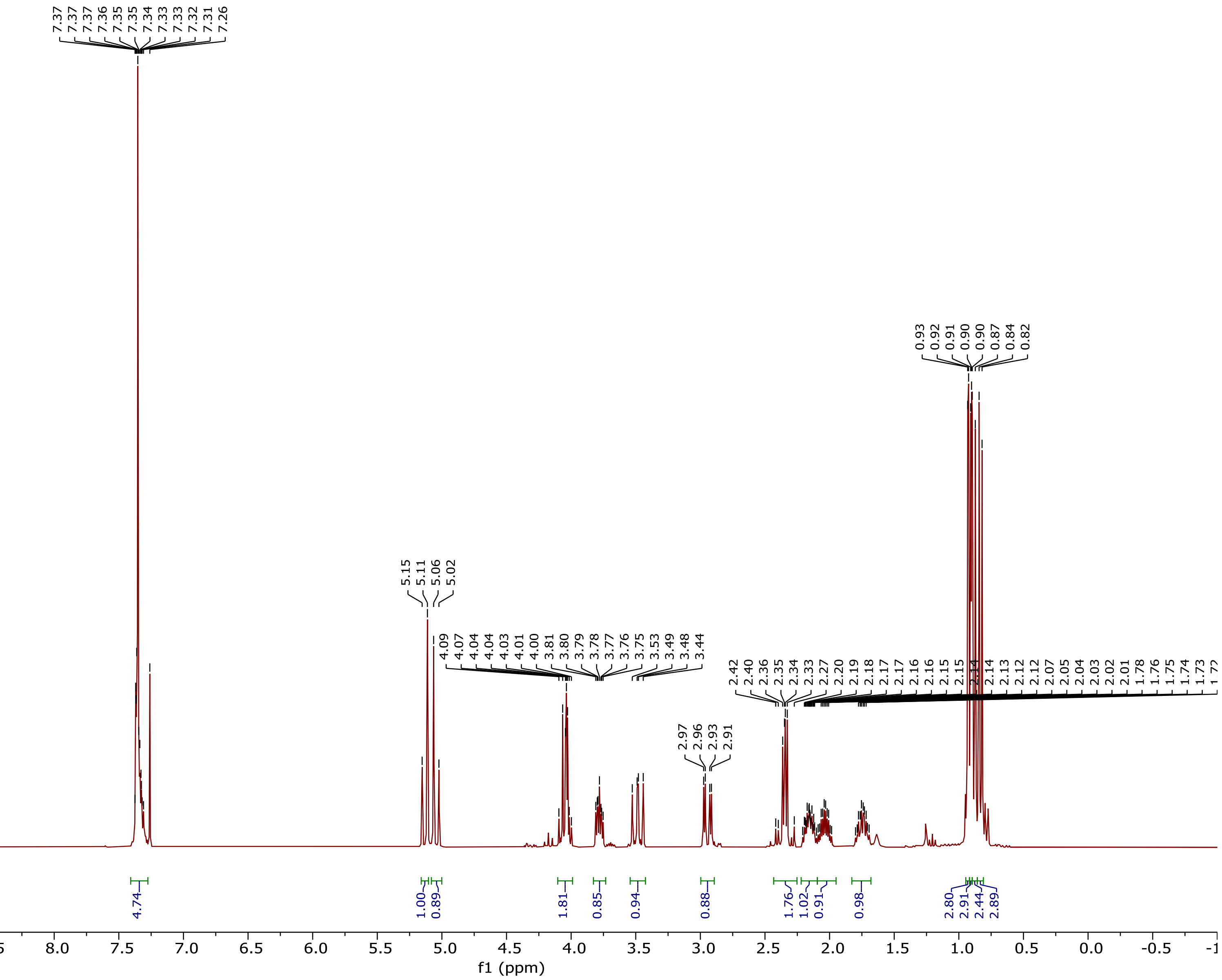

Benzyl 3-(((*S*)-4-isopropyl-2-oxooxazolidin-3-yl)methyl)-4-methyl-pentanoate (**69**)

^{13}C NMR (75 MHz, $CDCl_3$)

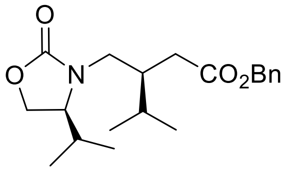

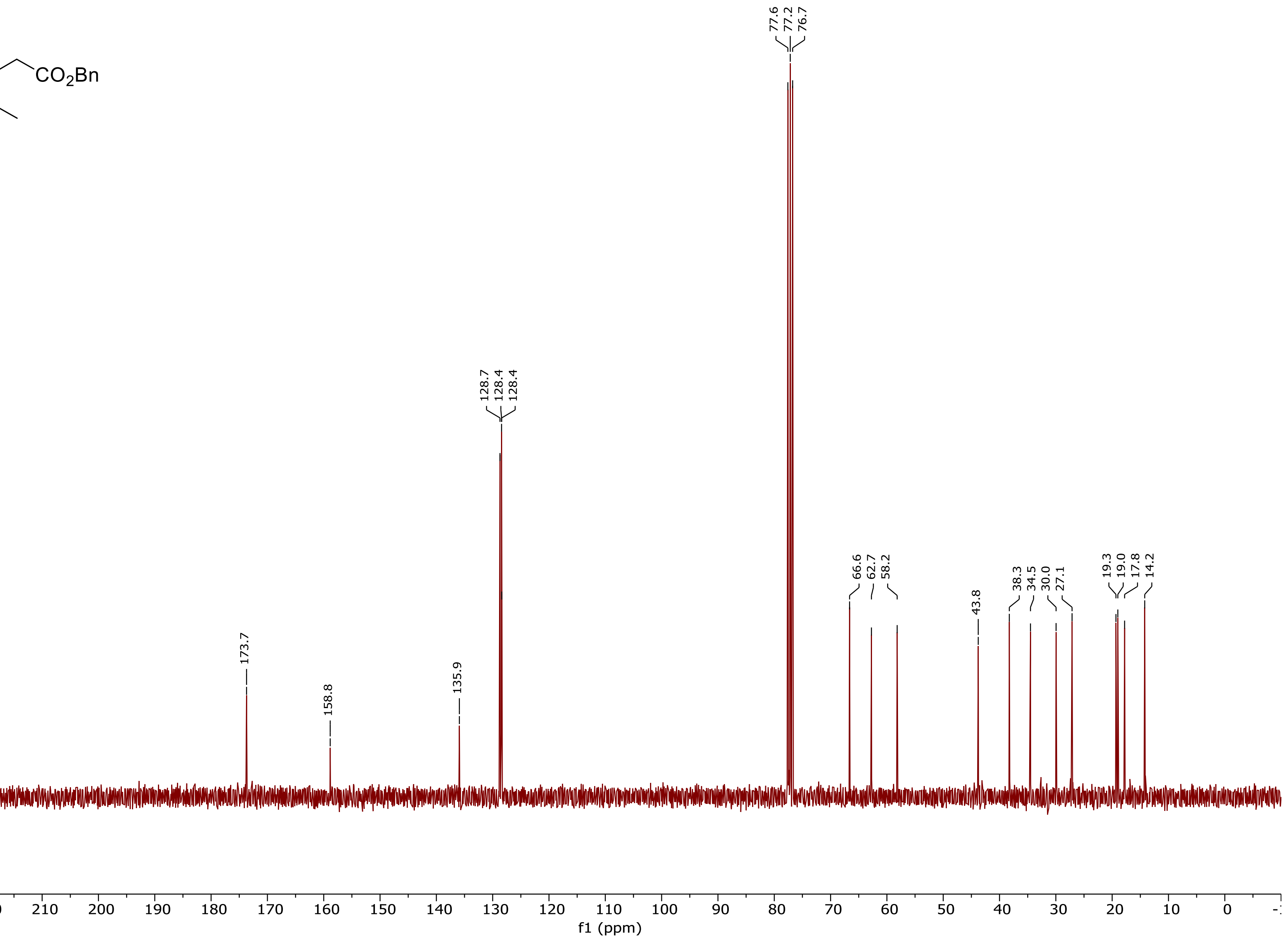

Ethyl (*R*)-3-(((4*S*,5*R*)-2-oxo-4,5-diphenyloxazolidin-3-yl)methyl)-pentanoate (**70**)

^{1}H NMR (300 MHz, $CDCl_3$)

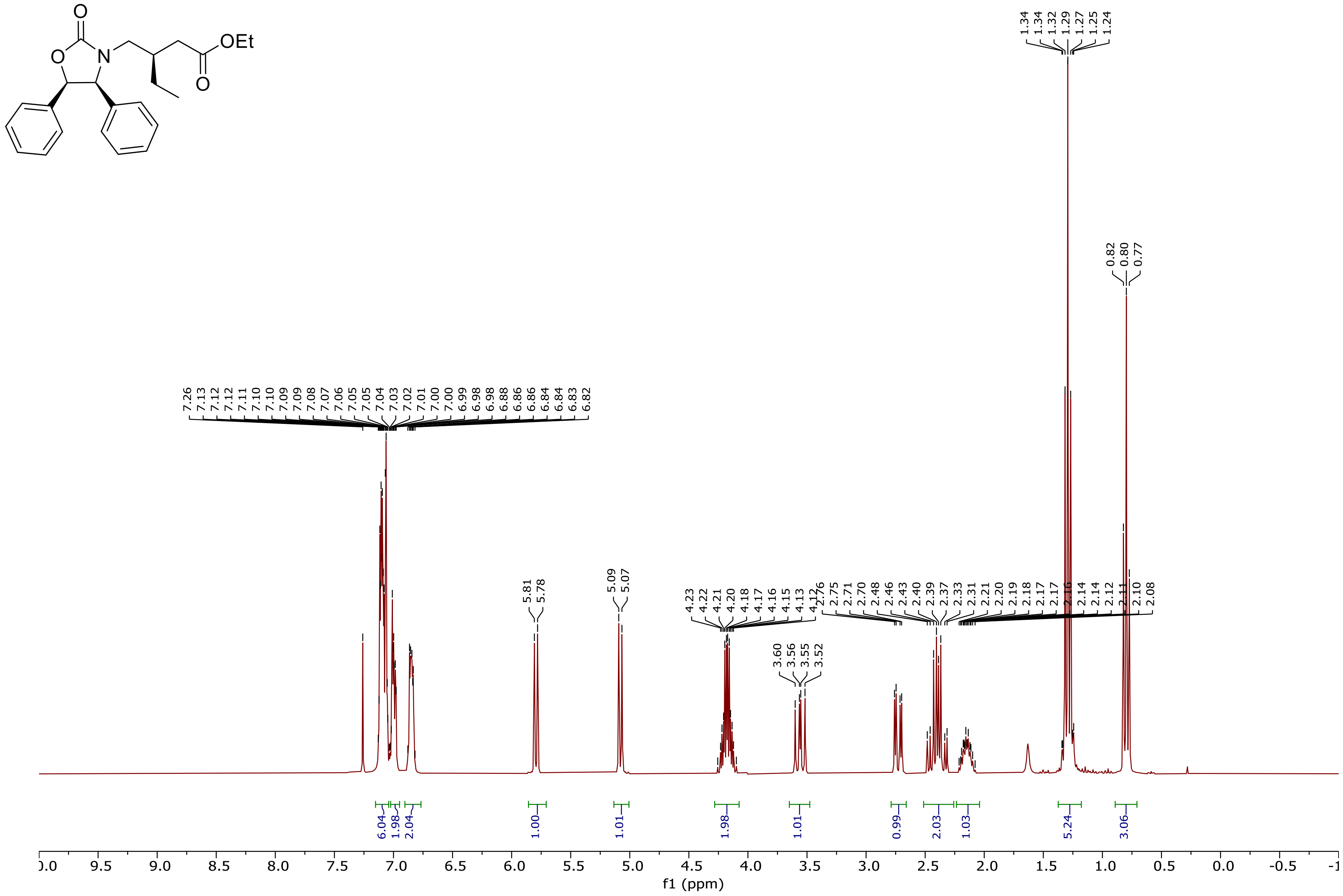

Ethyl (R)-3-((($4S$,$5R$)-2-oxo-4,5-diphenyloxazolidin-3-yl)methyl)-pentanoate (**70**)

^{13}C NMR (75 MHz, $CDCl_3$)

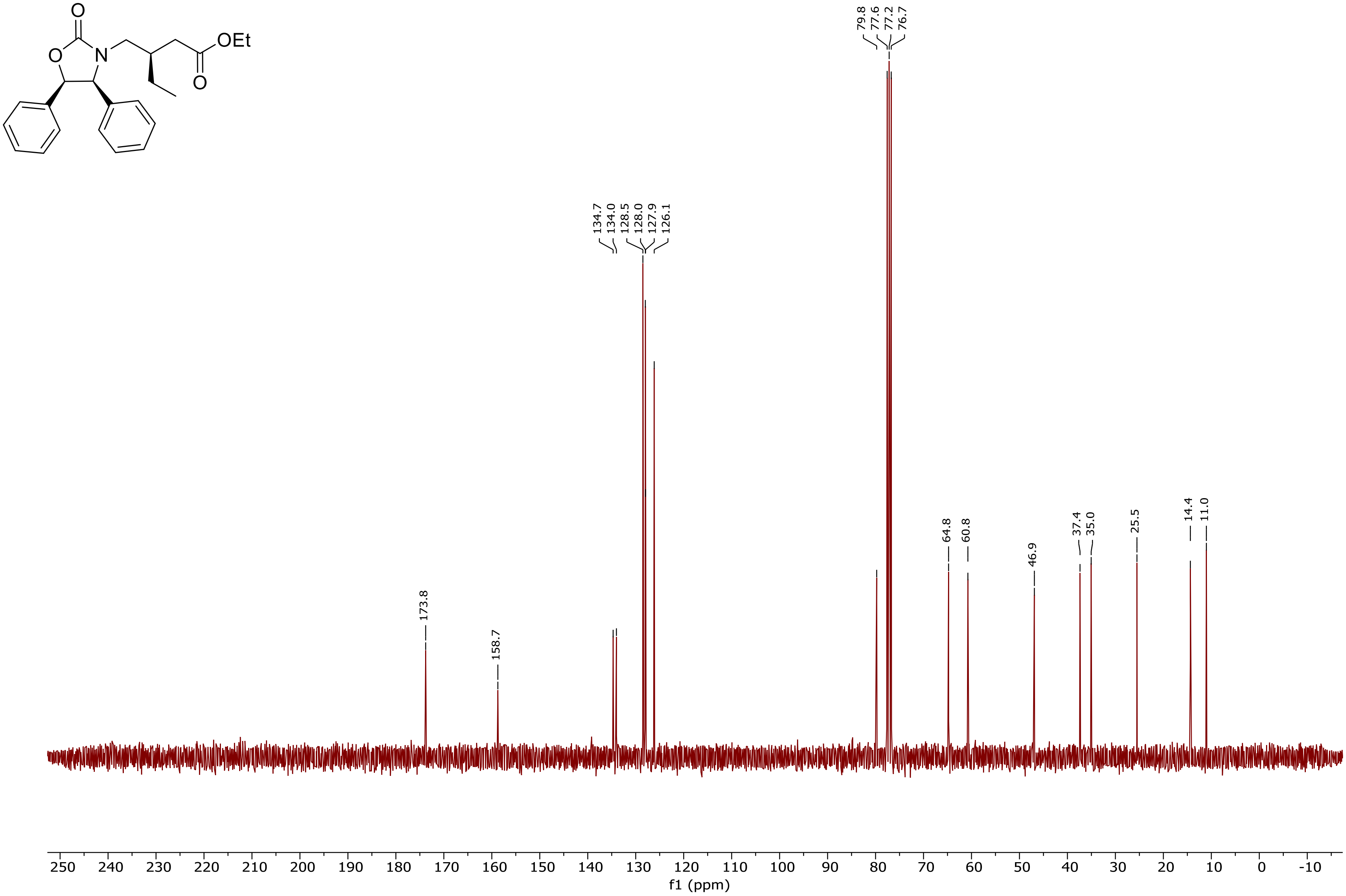

Benzyl 3-(((4*S*,5*R*)-2-oxo-4,5-diphenyloxazolidin-3-yl)methyl)pentanoate (**71**) ^{1}H NMR (300 MHz, $CDCl_3$)

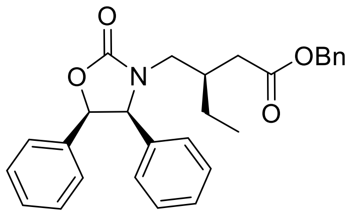

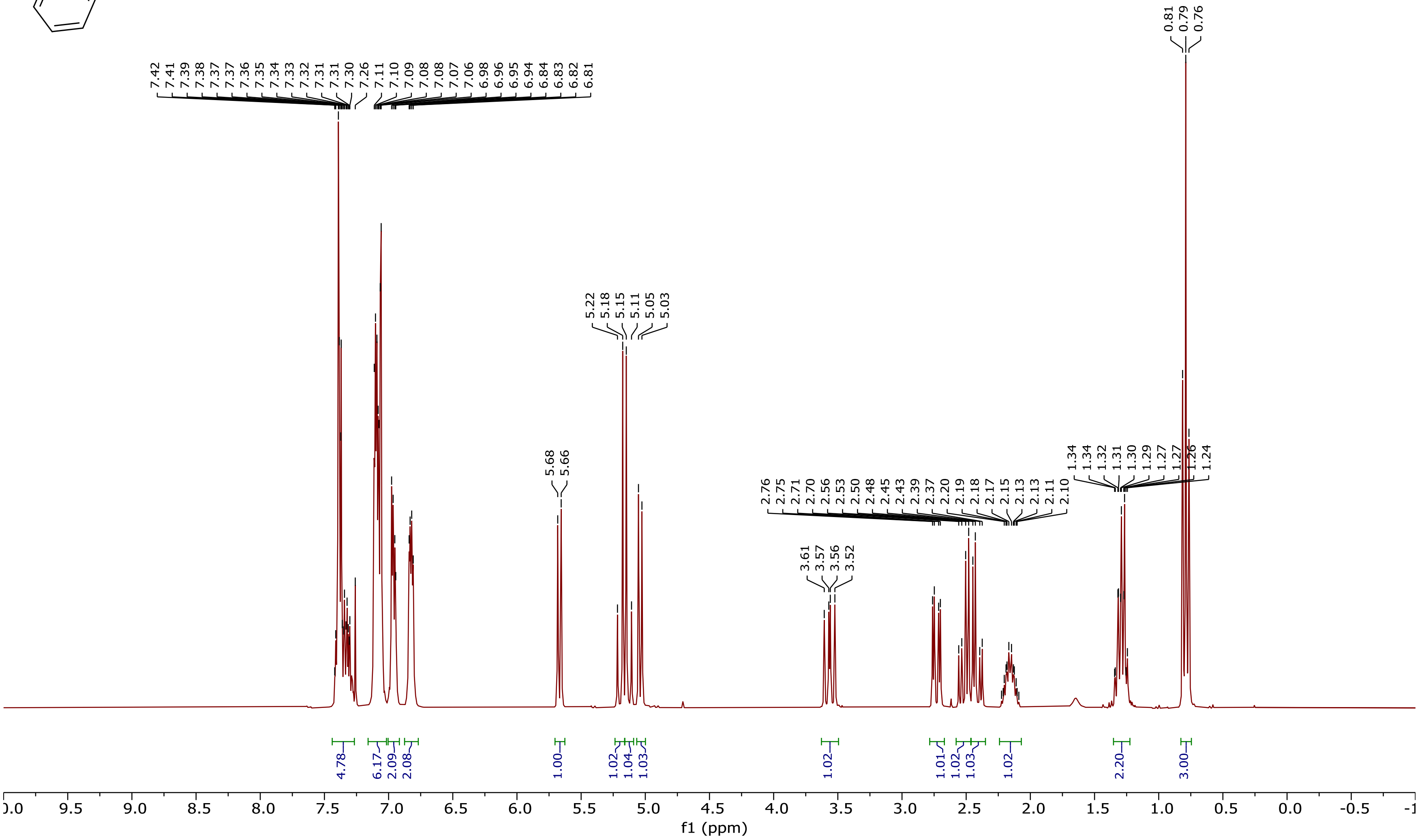

Benzyl 3-(((4*S*,5*R*)-2-oxo-4,5-diphenyloxazolidin-3-yl)methyl)pentanoate (**71**) ^{13}C NMR (75 MHz, $CDCl_3$)

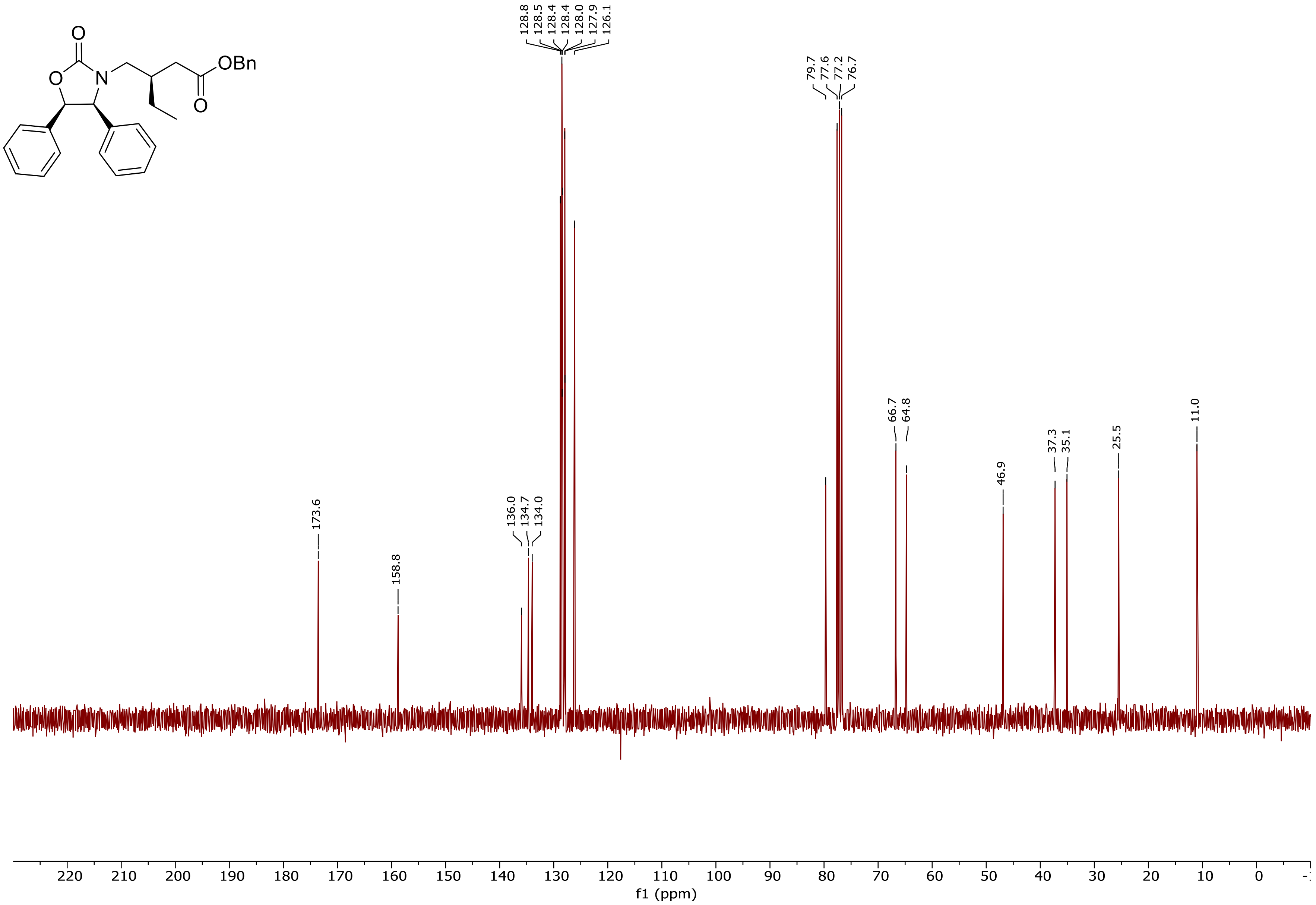

(3*S*,8*S*,9*S*,10*R*,13*S*,14*S*,17*S*)-10,13-Dimethyl-17-((*R*)-5-oxotetrahydrofuran-2-yl)-2,3,4,7,8,9,10,11,12,13,14,15,16,17-tetradecahydro-1*H*-cyclopenta[*a*]phenanthren-3-yl acetate (**42**)

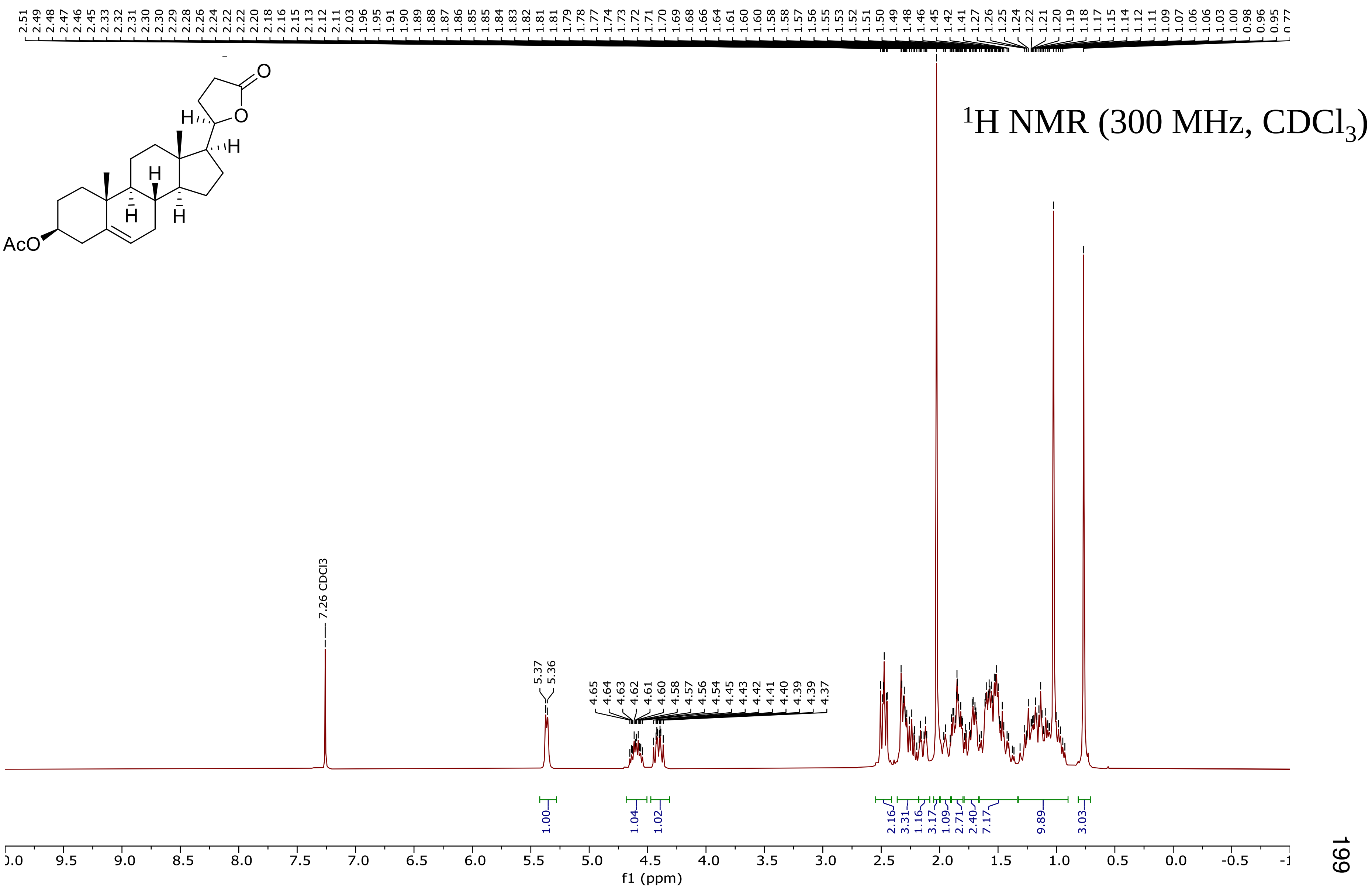

(3*S*,8*S*,9*S*,10*R*,13*S*,14*S*,17*S*)-10,13-Dimethyl-17-((*R*)-5-oxotetrahydrofuran-2-yl)-2,3,4,7,8,9,10,11,12,13,14,15,16,17-tetradecahydro-1*H*-cyclopenta[*a*]phenanthren-3-yl acetate (**42**)

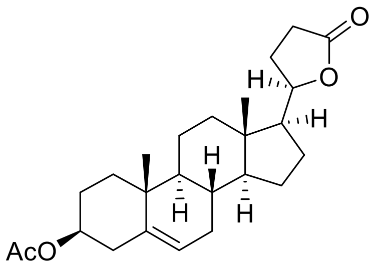

^{13}C NMR (75 MHz, $CDCl_3$)

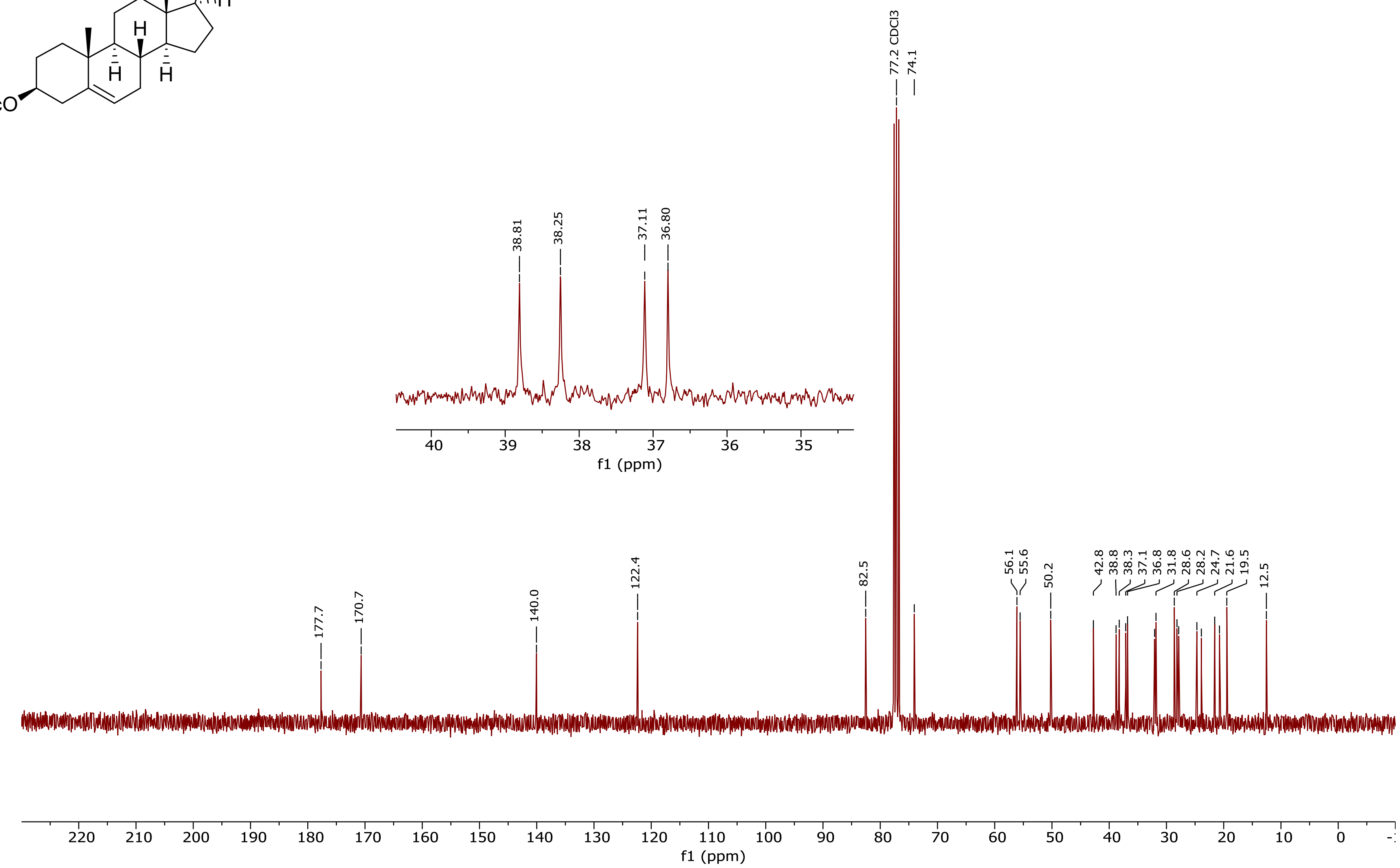

Ethyl 2-((*8R*,*9S*,*13S*,*14S*,*16S*,*17S*)-17-hydroxy-3-methoxy-13-methyl-7,8,9,11,12,13,14,15,16,17-decahydro-6*H*-cyclopenta[a]phenanthren-16-yl)acetate (**43**)

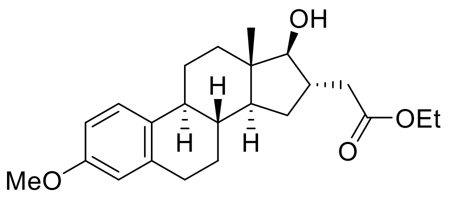

^{1}H NMR (300 MHz, $CDCl_3$)

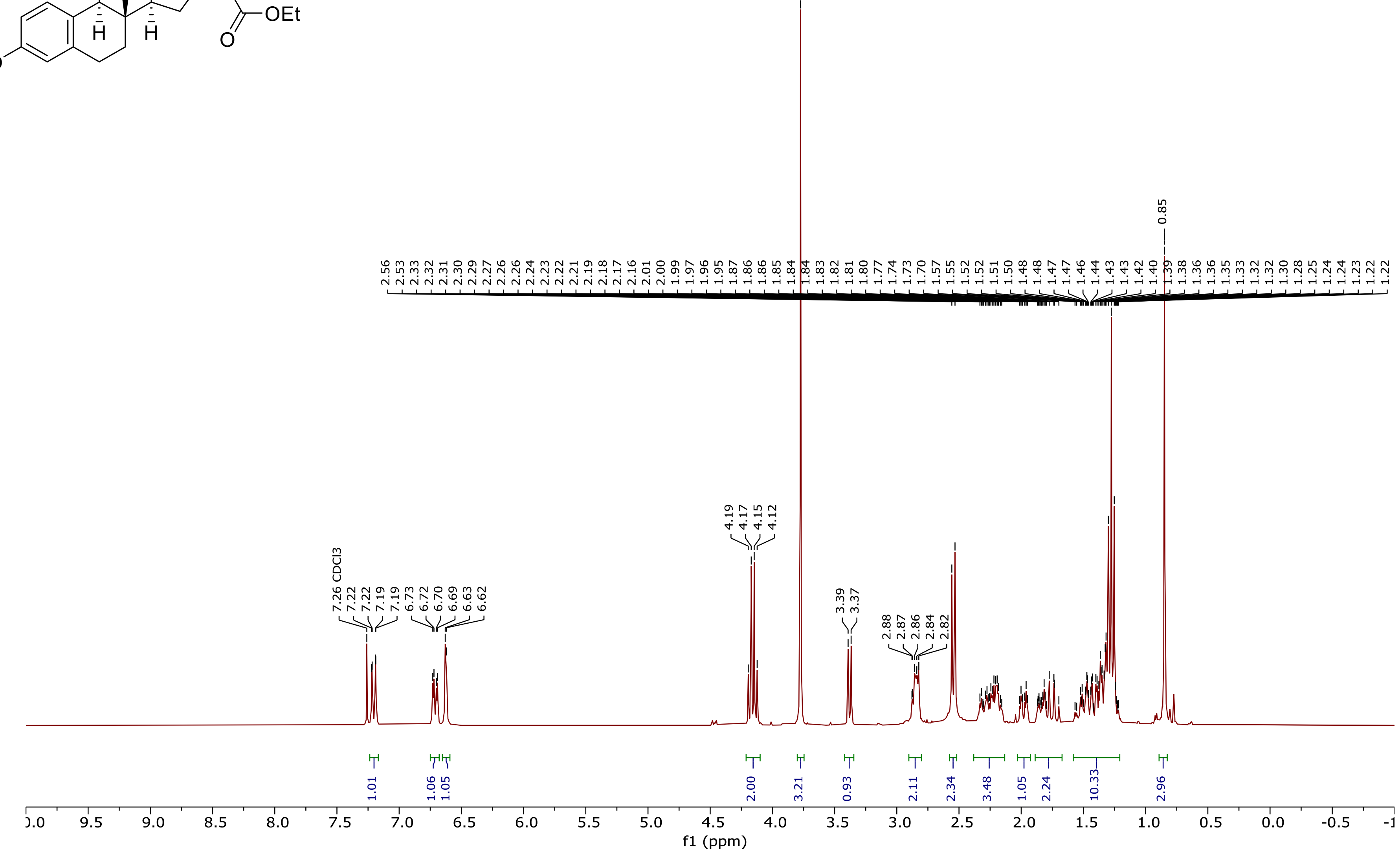

Ethyl 2-((*8R*,*9S*,*13S*,*14S*,*16S*,*17S*)-17-hydroxy-3-methoxy-13-methyl-7,8,9,11,12,13,14,15,16,17-decahydro-6*H*-cyclopenta[a]phenanthren-16-yl)acetate (**43**)

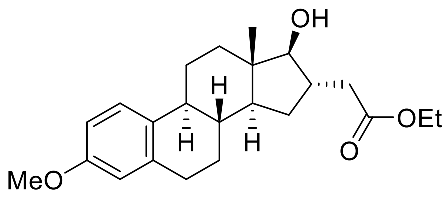

^{13}C NMR (75 MHz, $CDCl_3$)

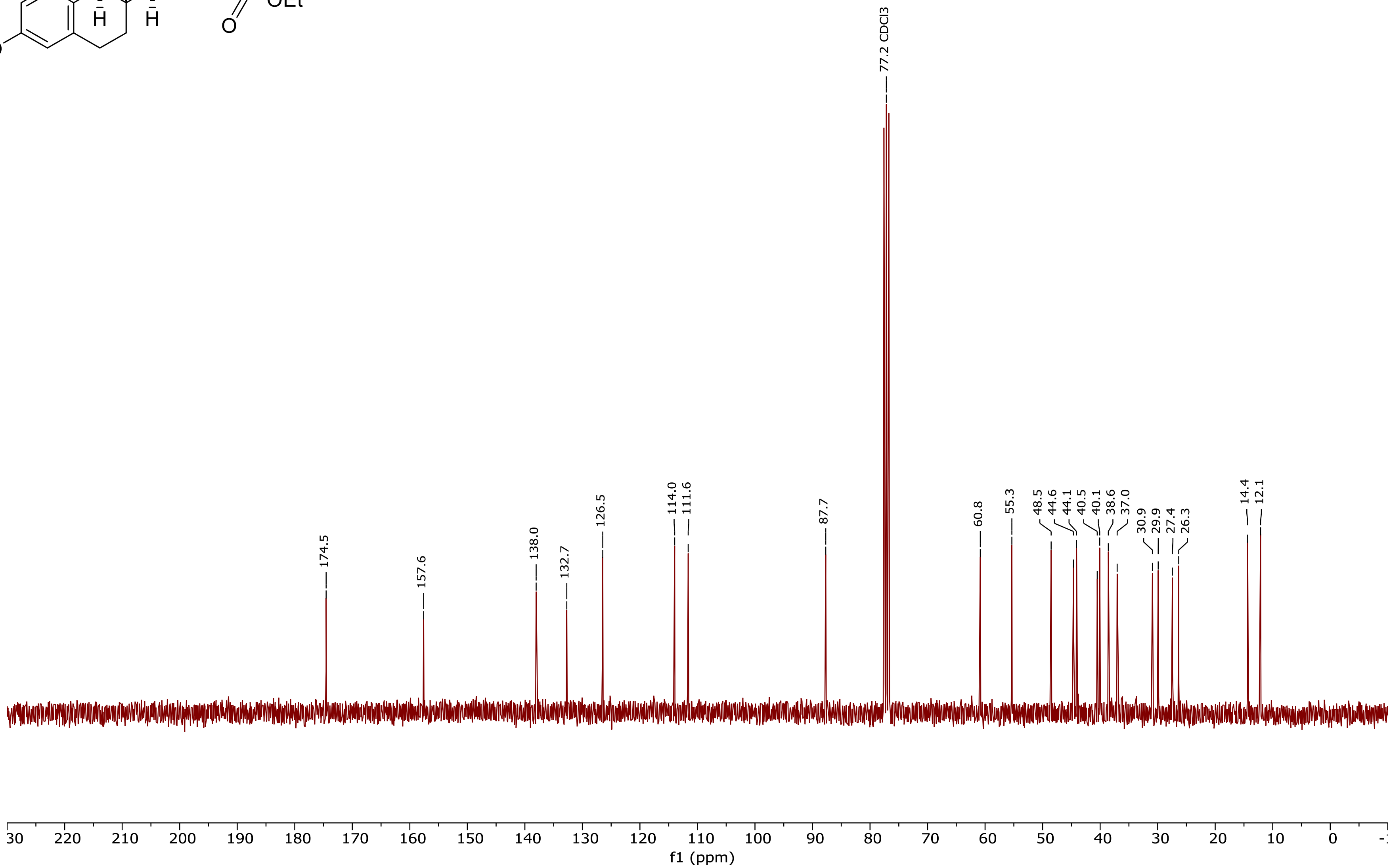

Ethyl (*R*)-3-(((tert-butoxycarbonyl)amino)methyl)pentanoate (**72**)

^{1}H NMR (300 MHz, $CDCl_3$)

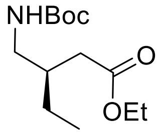

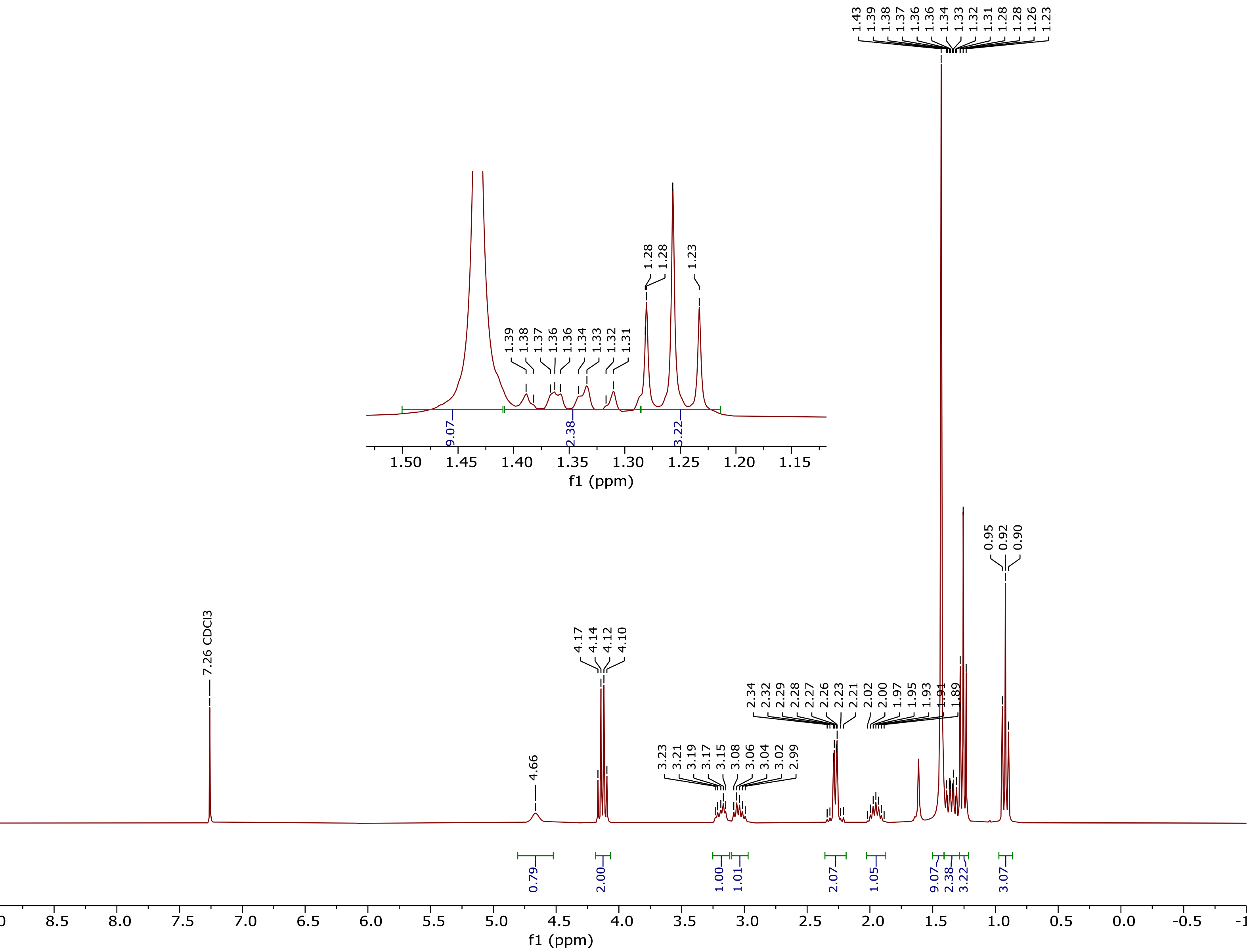

Ethyl (*R*)-3-(((tert-butoxycarbonyl)amino)methyl)pentanoate (**72**) ^{13}C NMR (101 MHz, $CDCl_3$)

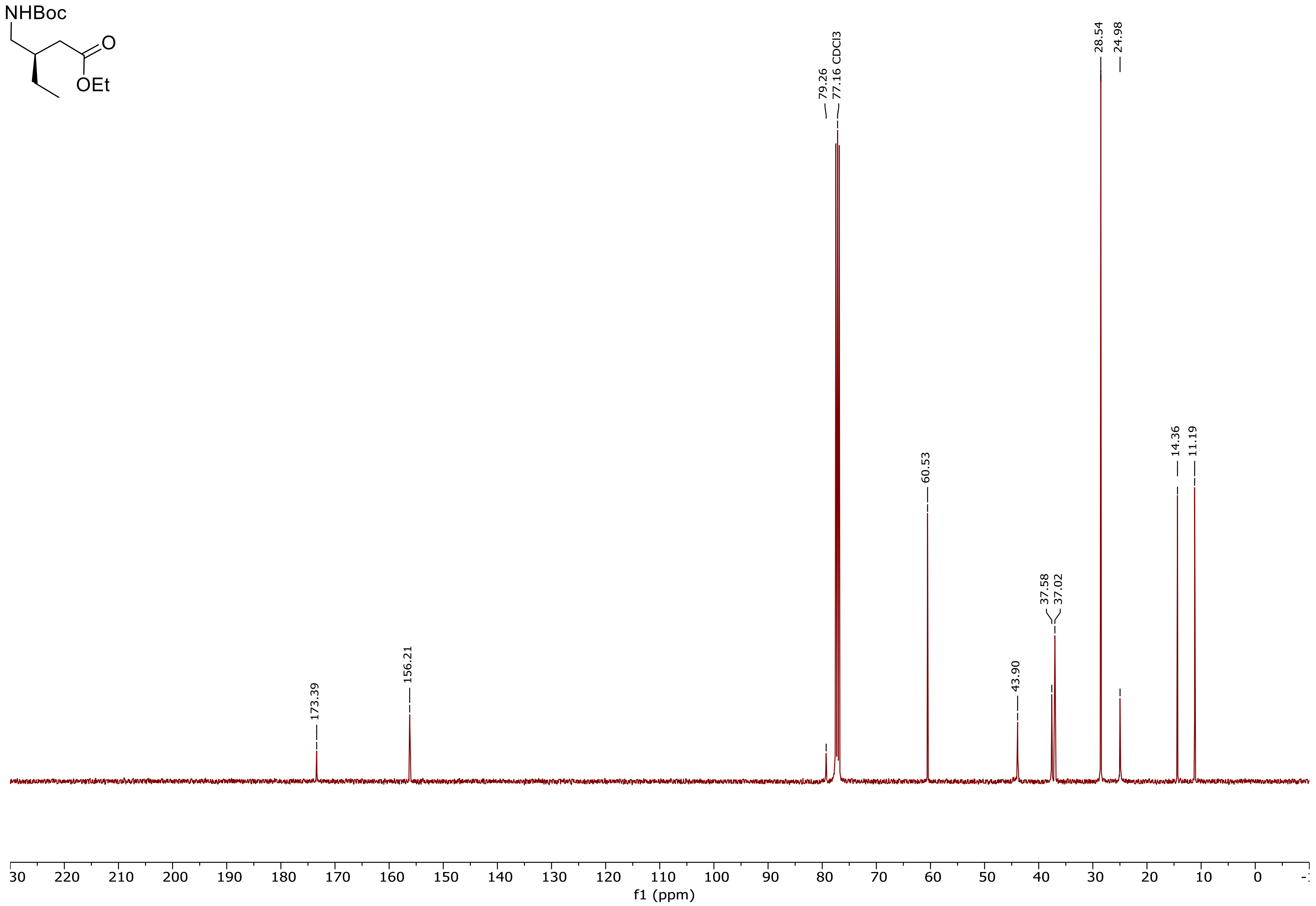

Supplement: SC-012-D0SC06341J-s001 [file SC-012-D0SC06341J-s001.pdf]
